# Synthesis of Aza‐indeno‐aza‐fluoranthene and Constitutional Isomers by Ring‐Size Selective C–H Activation

**DOI:** 10.1002/chem.202502960

**Published:** 2025-11-11

**Authors:** Christoph Keck, Frank Rominger, Sonali Garg, Marcus Elstner, Michael Mastalerz

**Affiliations:** ^1^ Organisch‐Chemisches Institut Ruprecht‐Karls‐Universität Heidelberg Im Neuenheimer Feld 272 Heidelberg 69120 Germany; ^2^ Institut für Physikalische Chemie und Theoretische Chemische Biologie Karlsruher Institut für Technologie (KIT) Kaiserstraße 12 Karlsruhe 76131 Germany

**Keywords:** aza‐fluoranthene, aza‐helicene, benzannelation, indeno‐annelation, ring‐size selectivity

## Abstract

The fluoranthene motif combines a planar geometry with electron‐deficient character, rendering fluoranthene and its larger congeners like indenofluoranthene suitable compounds for designing *n*‐semiconducting materials for applications in organic electronics. However, examples in which their opto‐ and electrochemical properties are tuned through selective five‐ or six‐membered ring annelation, as well as by isosteric nitrogen substitution of a C–H unit within the fluoranthene core, remain scarce. Here the structure–property‐relationships of a series of aza‐fluoranthene‐derived constitutional isomers as well as their syntheses by selective Pd‐catalyzed indeno‐ or benzannelation of the same precursor is described.

## Graphical abstract

A series of constitutional isomers of aza‐indeno‐aza‐fluoranthene with different ring sizes has been synthesized. Their product distribution was controlled by adjusting parameters such as base, temperature, solvent, or additive in the Pd‐catalyzed direct arylation step, thereby providing an overview about the options to control ring‐size selectivity in C–H activation reactions.

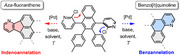

## 1 Introduction

Fluoranthene, a polycyclic aromatic hydrocarbon (PAH) composed of cyclopenta‐fused benzene and naphthalene units, has emerged as a versatile molecular scaffold in the design of advanced π‐conjugated systems.^[^ ^1^ ^]^ The presence of a five‐membered ring within the aromatic backbone creates a nonalternant π‐system with unique electronic and photophysical properties, such as small HOMO–LUMO bandgaps.^[^ ^2^, ^3^ ^]^ A benefit of many nonalternant hydrocarbons is the combination of high stability with low band gaps; being significantly more stable than for instance acenes with comparable band gaps.^[^ ^4^, ^5^, ^6^, ^7^, ^8^, ^9^ ^]^ Its planarity, caused by the rigid polycyclic core and electron‐deficient character due to the central five‐membered ring makes fluoranthene and its derivatives highly attractive for applications for organic semiconductors,^[^ ^10^ ^]^ optoelectronic materials,^[^ ^11^, ^12^ ^]^ or supramolecular chemistry.^[^ ^13^ ^]^

**Figure 1:**
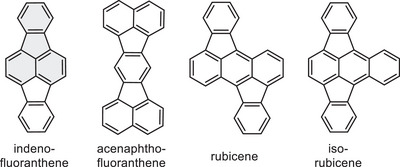
Molecular structures of fluoranthene‐containing motifs, which is highlighted in gray.

Typical fluoranthene‐containing structural motifs are indenofluoranthene,^[^ ^14^, ^15^, ^16^, ^17^ ^]^ acenaphthofluoranthene,^[^ ^18^ ^]^ rubicene, or isorubicene (Figure 1).^[^ ^16^, ^19^, ^20^, ^21^, ^22^ ^]^

Typical strategies to modulate the electronic properties of fluoranthene‐derived π‐systems are the functionalization with electron‐withdrawing groups like cyano‐ or imide groups,^[^ ^12^, ^23^ ^]^ or the incorporation of nitrogen atoms into the aromatic backbone.^[^ ^24^, ^25^, ^26^, ^27^ ^]^ The isosteric substitution of CH units by nitrogen leads to aza‐analogues that often show lower LUMO levels, and increased electron affinity compared to their all‐hydrocarbon analogues, as shown for example for *N*‐heteroacenes,^[^ ^7^, ^28^, ^29^, ^30^, ^31^ ^]^ aza‐perylenes, or aza‐coronenes.^[^ ^32^, ^33^ ^]^ However, aza‐analogues of fluoranthene are rare,^[^ ^24^, ^25^, ^26^, ^34^, ^35^ ^]^ and to the best of our knowledge no aza‐analogue of an indeno‐fluoranthene has been reported so far.

One challenge in expanding the chemical space of fluoranthene‐based compounds is regioselective functionalization. In 1992, Rice and Cai were the first to develop a multistep synthetic protocol for the preparation of substituted fluoranthenes by Kumada–Corriu coupling of two aryls, followed by triflation and Pd‐catalyzed indenoannelation with 1,8‐diazabicyclo[5.4.0]undec‐7‐ene (DBU).^[^ ^36^, ^37^ ^]^ About 10 years later, de Meijere and coworkers reported an approach to perform PAH indenoannelation in a single reaction step—a Suzuki–Miyaura–Heck‐type coupling cascade between *ortho*‐halo boronic acids and bromoaryls, using Pd_2_(dba)_3_ in the presence of PCy_3_ and DBU.^[^ ^16^, ^38^ ^]^ Following this strategy, fluoranthene, indeno‐fluoranthene, rubicene, and isorubicene were synthesized in yields ranging from 27% to 97%.^[^ ^16^ ^]^ In 2006, Scott and coworkers were able to quantitatively synthesize indenofluoranthene using further optimized reaction conditions with Pd(PCy_3_)_2_Cl_2_ and DBU in *N*,*N*‐dimethylacetamide (DMAc).^[^ ^38^ ^]^ As noted by Rice and Cai,^[^ ^37^ ^]^ the use of DBU promoted exclusively the formation of five‐membered rings, even in substrates where generation of six‐membered rings could also occur. However, no alternative bases to DBU were examined in their study.

While small molecules can still be designed to undergo a single, well‐defined mode of ring closure in Pd‐catalyzed direct arylations,^[^ ^39^ ^]^ this becomes increasingly challenging with larger PAHs.^[^ ^40^ ^]^ Therefore, understanding the parameters that govern ring‐size selectivity in C–H activation is of particular importance. In this respect, Würthner and coworkers where the first to shed light on the crucial role of the base in C–H activation, involving substrates capable of forming both five‐ or six‐membered rings.^[^ ^41^ ^]^ They reported a chemo‐selective synthesis of five‐ and six‐membered ring‐fused systems via intramolecular C–H activation depending on the choice of base. The chemo‐selectivity was assumed to occur from different mechanisms in the cyclopalladation step. Beyond the choice of base, the mechanism of carbopalladation can also be controlled by additives such as pivaloates or by variation of the reaction temperature.^[^ ^42^ ^]^

**Scheme 1:**
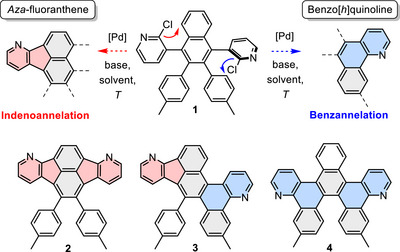
Molecular structures of chloropyridine precursor (1), aza‐indeno‐aza‐fluoranthene (2), aza‐fluorantheno‐aza‐tetrahelicene (2), di‐aza‐tribenzo‐pentahelicene (3).

Here, in this work, a chloropyridine precursor (**1**, Scheme 1) is subjected to selective Pd‐catalyzed indeno‐ or benzannelation to form either aza‐fluoranthene or benzo[*h*]quinoline units resulting in either a di‐aza‐analogue of indenofluoranthene (**2**) or one of the two constitutional isomers aza‐fluorantheno‐aza‐tetrahelicene (**3**) and di‐aza‐tribenzo‐pentahelicene (**4**). To achieve selectivity, the influence of base, additive, solvent, and temperature on annelation was investigated and the underlying cyclopalladation mechanisms were calculated by density‐functional theory (DFT).

## 2 Results and Discussion

### 2.1 Synthesis

**Scheme 2:**
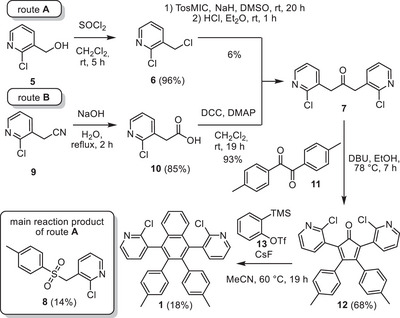
Synthesis of chloropyridine precursor **7**.

Chloropyridine precursor **1** was synthesized in four steps (Scheme 2) by two different routes with respect to the first two reaction steps. Route A started with the chlorination of alcohol **5** to give chloride **6** in 96% yield,^[^ ^43^ ^]^ followed by a coupling using the van Leusen reagent (toluenesulfonylmethyl isocyanide, TosMIC) and acidic hydrolysis to obtain ketone **7** in only 6% yield as a side‐product.^[^ ^44^, ^45^ ^]^ The main product was 2‐chloro‐3‐(tosylmethyl)pyridine (**8**) with 14% yield, which was clearly identified by single‐crystal X‐ray diffraction analysis (see Supporting Information).

Alternatively, ketone **7** was synthesized by route B, starting from nitrile **9**, that was transformed to carboxylic acid **10** in 85% yield,^[^ ^46^ ^]^ which was converted to ketone **7** in 93% under Steglich conditions using dicyclohexyl carbodiimide (DCC) and 4‐dimethylaminopyridine (DMAP). In the next step, ketone **7** was condensed with 4,4′‐dimethylbenzil (**11**) to give cyclopentadienone **12** in 57% yield. Finally, chloropyridine **1** was obtained in 18% yield, by reaction of **12** with benzyne (which is formed in‐situ from **13** using CsF).^[^ ^47^ ^]^

**Scheme 3:**
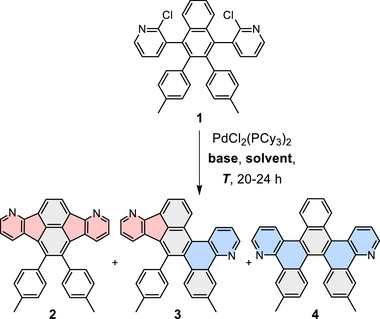
Synthesis of **2**–**4**. Conditions: PdCl_2_(PCy_3_)_2_ (20 mol%), base (10 equiv.), DMAc, or mesitylene, 160 °C (see Table 1 for details).

Chloropyridine **1** allows divergent reactivity in Pd‐catalyzed C–H activation reactions: the chloropyridine units can undergo either indenoannelation with the naphthyl core to give an *aza*‐fluoranthene moiety, or benzannelation with a tolyl group, forming a benzo[*h*]quinoline subunit and inducing helical distortion to the π‐system (Scheme 3 and Table 1). This leads to the possibility of the formation of three constitutional isomers **2**–**4**. For the C–H activation, PdCl_2_(PCy_3_)_2_ was used in combination with various organic (DBU, DIPEA, LiHMDS) or inorganic bases (K_3_PO_4_, K_2_CO_3_). Reactions were conducted in anhydrous dimethylacetamide (DMAc) or mesitylene at temperatures ranging from 140 to 200 °C. The use of potassium phosphate, lithium bis(trimethylsilyl)amide (LiHMDS), or potassium carbonate in DMAc led exclusively to the formation of the twofold benzannelated product **4** (Table 1, Entries 1–6). With K_2_CO_3_ (Entry 4) higher yields (45%), were achieved than with K_3_PO_4_ (16%, Entry 1), but similar yields to those with LiHMDS (44%, Entry 2). The yields with K_2_CO_3_ could be further improved by doubling either reaction time (51%, Entry 5) or catalyst loading (72%, Entry 6), whereas lowering the reaction temperature to 140 °C gave lower yields (26%, Entry 3). When the solvent was exchanged for mesitylene with otherwise unchanged reaction conditions, the yield could be further increased to 74% (Entry 7). In all of these reactions, the helicene **4** formed exclusively and no other annelated products were detected.

**Table 1:** Isolated yields and conditions of the palladium‐catalyzed C–H activation reaction in Scheme 3: PdCl_2_(PCy_3_)_2_ (20mol%), DMAc, *t* = 20–24 hours, 10 equiv. of base in total. Optimized reaction conditions for the synthesis of fluoranthene **2** and helicene **4** are highlighted in bold.

Entry	Base	*T* [°C]	Yield 2 [%]	Yield 3 [%]	Yield 4 [%]
1	K_3_PO_4_	160	0	0	16
2	LiHMDS	160	0	0	44
3	K_2_CO_3_	140	0	0	26
4	K_2_CO_3_	160	0	0	45
5^[^ ^a^ ^]^	K_2_CO_3_	160	0	0	51
6^[^ ^b^ ^]^	K_2_CO_3_	160	0	0	72
7^[^ ^c^ ^]^	K_2_CO_3_	160	0	0	**74**
8	DBU	140	32	14	10
9	DBU	160	**53**	10	3
10	DBU	180	22	14	10
11	DBU	200	14	15	10
12^[^ ^d^ ^]^	DBU	160	5	20	51
13^[^ ^c^ ^]^	DBU	160	42	29	23
14	DBU/ K_2_CO_3_	160	0	0	49
15	DIPEA	160	0	0	0

^[a]^ Increased reaction time of 48 hours.

^[b]^ Increased catalyst loading (40 mol% PdCl_2_(PCy_3_)_2_).

^[c]^ Solvent: mesitylene instead of DMAc.

^[d]^ Additive: PivOH (40 mol%).

In contrast, when DBU in DMAc was used, both five‐ and six‐membered ring annelation occurred, with a clear preference toward the twofold indenoannelated product **2** (Entries 8–9). At 140 °C, fluoranthene **2** was formed in 32% yield besides small amounts of compound **3** (14%) and helicene **4** (10%). At a higher temperature of 160 °C, the yield of fluoranthene **2** increased to 53%, while the formations of compound **3** (10%) and helicene **4** (3%) were reduced.

Almost identical reaction conditions were employed by Scott and co‐workers to synthesize indenofluoranthene by C–H activation of the respective aryl chloride in quantitative yield at 155 °C.^[^ ^38^ ^]^ At even higher temperatures (180 or 200 °C), both the selectivity toward fluoranthene **2** and the combined yields of all C–H activation products (**2**–**4**) decrease significantly (Entries 10–11). Adding PivOH to the reaction with DBU in DMAc (Entry 12) favored the formation of helicene **4** (51%) over fluoranthene **2** (5%), inverting the product distribution compared to the reaction without PivOH (Entry 9). Previous studies have already shown, that the ring‐size selectivity in C‐H activations may be switched by the variation of base, additive, or temperature and this is also the case here.^[^ ^35^, ^41^, ^42^ ^]^ However, the choice of solvent (DMAc or mesitylene) may also play a role for ring‐size selectivity, because when the solvent was exchanged for mesitylene, the selectivity for indenoannelation with DBU decreased (Entry 13) in addition the main product was formed in lower yields than with DMAc. An attempt to make use of the observed base‐selectivity for a preferential formation of the mixed indeno‐ and benzannelated compound **3** by mixing K_2_CO_3_ and DBU (Entry 14) produced helicene **4** exclusively. When diisopropylethylamine (DIPEA, Hünig's base) was used, no product formation was observed at all (Entry 15). It is worth mentioning that annulation by CH‐activation was only observed with PdCl_2_(PCy_3_)_2_ as a catalyst precursor. With (Pd(PPh_3_)_4_, PdCl_2_(PPh_3_)_2_) predominantly starting material was re‐isolated and the formation of traces of mono‐cyclodehydrochlorinated product were observed.

**Figure 2:**
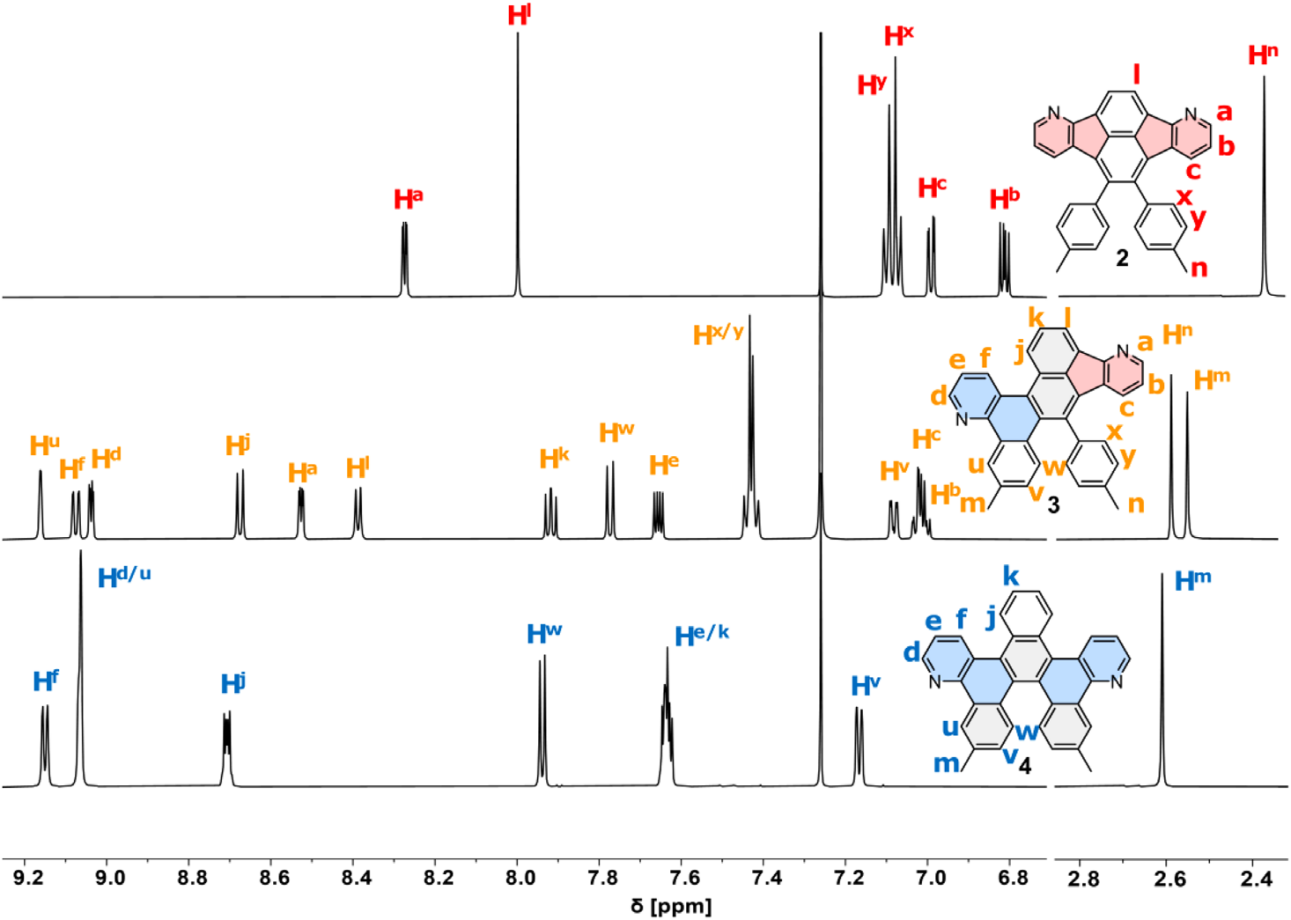
^1^H NMR spectra of **2**–**4** in CDCl_3_ (295 K, 600 MHz for **2**–**3**, 700 MHz for **4**).

Compounds **2**–**4** could be clearly distinguished by comparing their ^1^H NMR spectra (Figure 2). For the *C*_2v_‐symmetric fluoranthene **2**, both methyl groups are chemically equivalent and resonate as a singlet at *δ* = 2.37 ppm. Similarly, the methyl protons of pentahelicene **4** (point group *C*_2_) appear as one singlet at *δ* = 2.61 ppm. The ^1^H signals of the pyridine unit in helicene **4** (H^d‐f^) are shifted downfield by at least Δ*δ* = +0.8 ppm relative to the pyridine ^1^H signals in fluoranthene **2** (H^a‐c^). In contrast, the NMR spectrum of compound **3** is more complex due to lower symmetry (point group *C*_1_) and can be identified by two individual singlets for each methyl group, located at *δ* = 2.58 ppm (H^n^) and *δ* = 2.55 ppm (H^m^).

**Figure 3:**
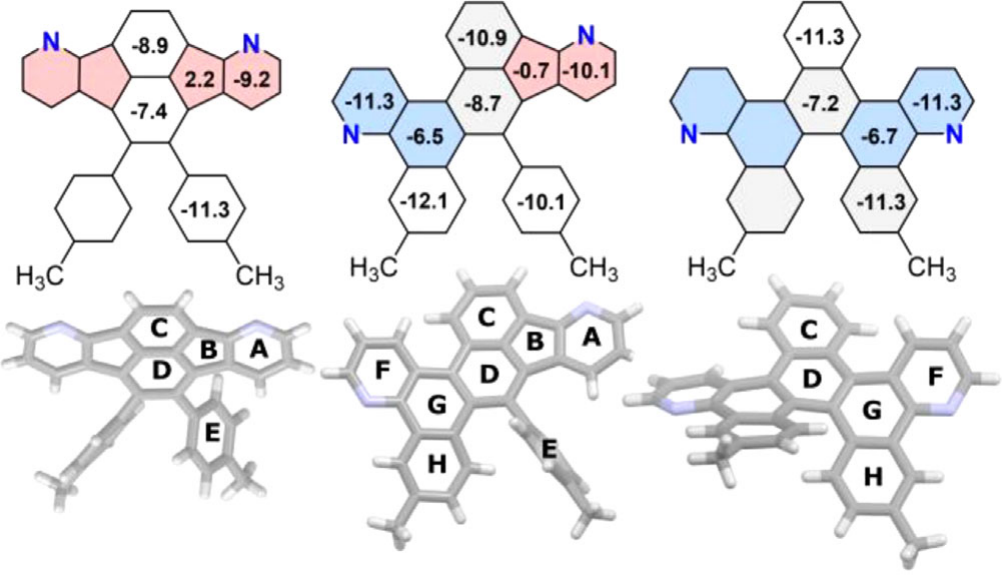
Calculated NICS(1)_av_‐values of **2**–**4** (Level of theory: HF‐GIAO/6–311g(d,p)).

Nucleus‐independent chemical shifts (NICS(1)_av_) were calculated (Figure 3, Supporting Information).^[^ ^48^, ^49^, ^50^, ^51^ ^]^ All hexagonal rings have negative values between ‐6.7 and ‐11.3 ppm indicating diamagnetic ring currents. The five‐membered ring shows nonaromatic character in fluoranthene **2** (+2.2 ppm) and compound **3** (‐0.7 ppm).^[^ ^52^ ^]^ NICS(1)_av_ calculations predict more negative values—and thus stronger diatropic ring currents—for the pyridine rings within the quinoline moieties than for those within the *aza*‐indene moieties by at least ‐1.2 ppm (rings A & F).^[^ ^53^ ^]^ This is supported by NMR measurements, where the quinoline ^1^H signals in helicene **4** are shifted downfield relative to the *aza*‐indene ^1^H signals in fluoranthene **2**.

### 2.2 Optical Properties and Electrochemistry

**Figure 4:**
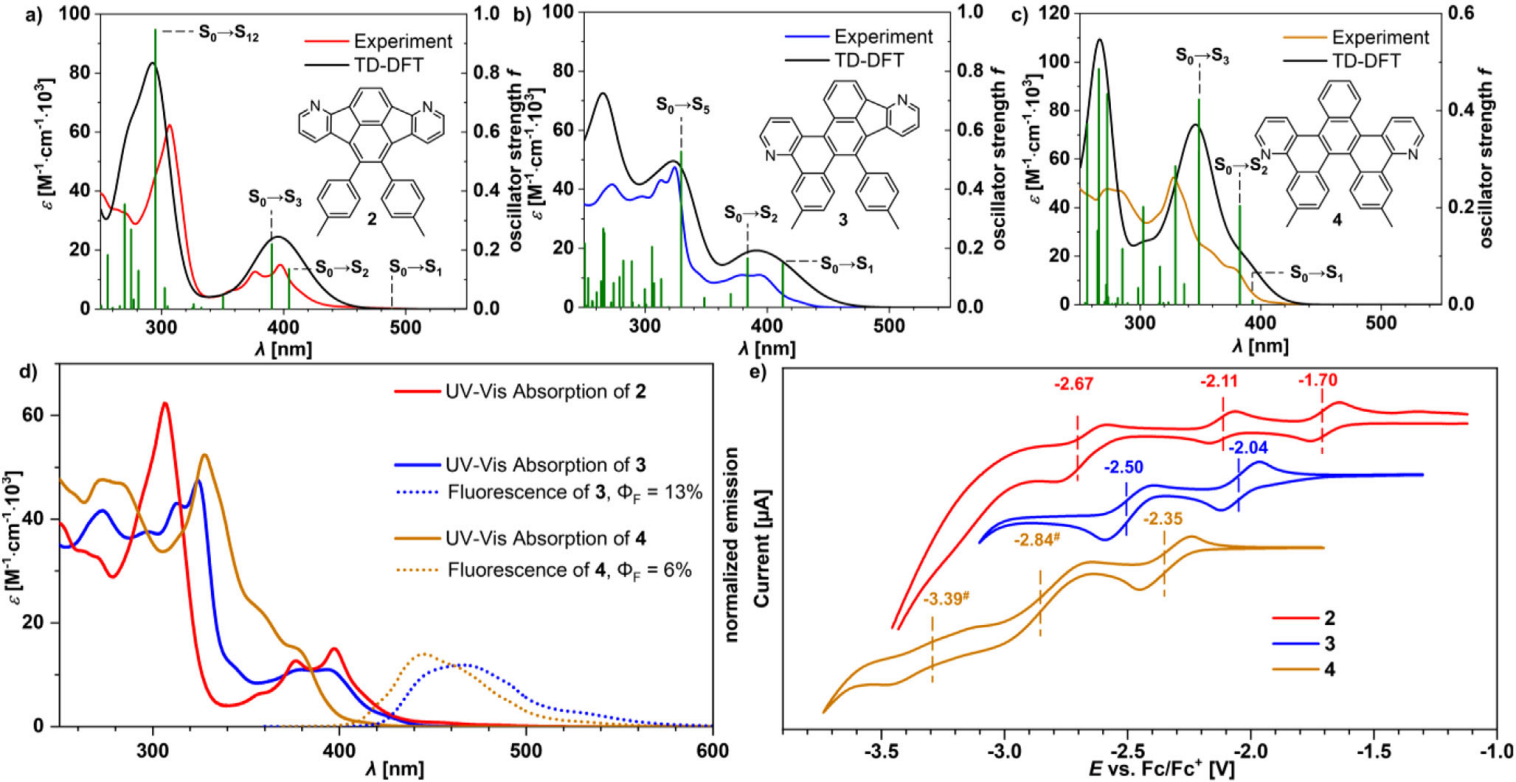
a‐c) Experimental (colored) and TD‐DFT‐simulated (black, B3LYP/6–311g(d,p) UV/vis spectra in CHCl_3_ at 23 °C. Oscillator strengths *f* (green) and dominant orbital contributions are marked accordingly. d) Experimental UV/Vis absorption and fluorescence spectra of **2**–**4** in CHCl_3_. e) Cyclic voltammograms of **2**–**4** measured in THF (0.05 m NBu_4_PF_6_, scan rate: 100 mV·s^−1^, working electrode: Pt, counter electrode: Pt, pseudo‐reference electrode: Ag/Ag^+^) versus Fc/Fc^+^ as internal reference. # reduction by differential pulse voltammetry (see Supporting Information).

Compounds **2**–**4** were investigated by UV/Vis absorption and fluorescence spectroscopy in chloroform solution and the spectra were analyzed using TD‐DFT calculations (Figure 4a‐d, Table 2 and Tables S7‐S9).

**Table 2:** Optical and electrochemical properties of compounds **2**–**4**.

Compound	*E*_HOMO_^[^ ^a^ ^]^ [eV]	*E*_LUMO_^[^ ^a^ ^]^ [eV]	*E*_g_^[^ ^a^ ^]^ [eV]	*λ*_abs_^[^ ^b^ ^],[^ ^c^ ^]^ [nm]	*λ*_onset_^[^ ^c^ ^]^ [nm]	*E*_g,opt_^[^ ^d^ ^]^ [eV]	*λ*_em_^[^ ^c^ ^]^ [nm]	Δ$\tilde{\nu }$_Stokes_ [cm^−1^]	*Φ*_fl_^[^ ^c^ ^]^ [%]	*E*_red._^[^ ^e^ ^]^ [V]	*EA* ^[^ ^f^ ^]^ [eV]
**2**	−5.9	−2.7	3.2	397	508	2.4	−	−	0	−1.7; −2.1; −2.7	−3.1
**3**	−5.8	−2.3	3.5	394	439	2.8	465	3875	13	−2.0; −2.5	−2.8
**4**	−5.8	−2.0	3.7	377	428	2.9	446	4103	6	−2.4; −2.8; −3.4	−2.5

^[a]^ Determined by DFT (level of theory: B3LYP/6–311g(d,p)).

^[b]^ Most red‐shifted absorption maximum.

^[c]^ Absolute photoluminescence quantum yields (solution in CHCl_3_ at 23 °C).

^[d]^ Estimated from the onset of the corresponding UV/Vis absorption spectrum (*E*_g,opt _= 1240/*λ*_onset_).

^[e]^ Determined by cyclic voltammetry measured in THF versus Fc/Fc^+^ with a Pt working electrode, Pt reference electrode, Ag/Ag^+^ pseudo‐reference electrode, and *n*Bu_4_NPF_6_ as electrolyte. Scanning speed: 100 mV· s^−1^.

^[f]^ Electron affinity determined via *EA* = ‐(*E*_red1 _+ 4.8) eV.

The UV/Vis spectrum of fluoranthene **2** shows two major absorption peaks in the near UV‐regime at *λ* = 397 nm (*ε* = 14,600 m ^−1^cm^−1^) and 377 nm (*ε* = 12,300 m ^−1^cm^−^ ^1^), which correspond to the S_0_→S_2_ (*λ* = 397 nm, *f *= 0.14) and the S_0_→S_3_ (*λ* = 377 nm, *f *= 0.22) transitions according to TD‐DFT (B3LYP/6‐311g(d,p), see Supporting Information). The spectrum is dominated by a third major absorption band in the mid‐UV regime at *λ* = 306 nm (*ε* = 62,400 m ^−1^cm^−1^), which corresponds to the S_0_→S_12_ (*λ* = 306 nm, *f *= 0.95) transition. Furthermore, fluoranthene **2** shows a broad low‐intensity absorption band in the onset regime at approx. *λ*_onset_ = 508 nm, that is associated with a weak S_0_→S_1_ transition (*f *= 0.006) at *λ* = 488 nm according to TD‐DFT and corresponds to an optical band gap of *E*_gap,opt_ = 2.4 eV. The spectral features are comparable to all‐carbon congeners of indenofluoranthene^[^ ^15^ ^]^ and other fluoranthene derivatives.^[^ ^41^, ^54^, ^55^, ^56^ ^]^

The UV/Vis absorption spectrum of compound **3** shows an absorption band in the near‐UV region with peaks at *λ* = 394 nm (*ε* = 10,900 m ^−1^cm^−1^) and *λ* = 379 nm (*ε* = 10,800 m ^−1^cm^−1^), which is similar to fluoranthene **2**, although the peaks are less pronounced in this case. According to TD‐DFT, the peaks in the near‐UV region correspond to the S_0_→S_1_ (*λ* = 394 nm, *f *= 0.15) and S_0_→S_2_ (*λ* = 379 nm, *f *= 0.17) transitions. The absorption onset is located near *λ*_onset_ = 439 nm, which corresponds to an optical band gap of *E*_gap,opt_ = 2.8 eV. A second absorption band in the mid‐UV region dominates the spectrum of compound **3**, similar to fluoranthene **2**, although compound **3** shows multiple well‐resolved peaks at *λ* = 324 nm (*ε* = 47,400 m ^−1^cm^−1^), 313 nm (*ε* = 42,800 m ^−1^cm^−1^) and 272 nm (*ε* = 41,300 m ^−1^cm^−1^), to which the S_0_→S_5_ transition makes the largest contribution (*f *= 0.53). The dominant mid‐UV absorption band in both fluoranthene **2** (S_0_→S_12_) and compound **3** (S_0_→S_5_) primarily originates from the HOMO→LUMO + 1 transition.

Helicene **4** shows the most bathochromically shifted absorption peak at *λ* = 377 nm (*ε* = 15,200 m ^−1^cm^−^ ^1^), which is a hypsochromic shift of at least 20 nm relative to the ones of compound **3** (*λ* = 397 nm) and fluoranthene **2** (*λ* = 394 nm). The absorption onset is dominated by the S_0_→S_2_ transition (HOMO→LUMO transition), because the S_0_→S_1_ transition is weak (*f *= 0.009). The onset is located near *λ*_onset_ = 428 nm and corresponds to an optical band gap of *E*_gap,opt_ = 2.9 eV, which is larger than for compound **3** (*λ*_onset_ = 439 nm, *E*_gap,opt_ = 2.8 eV) and fluoranthene **2** (*λ*_onset_ = 508 nm, *E*_gap,opt_ = 2.4 eV) and, thus, follows the trend of the DFT calculations.

Compound **3** and helicene **4** both show blue fluorescence at *λ*_em_ = 465 nm (**3**) and *λ*_em_ = 446 nm (**4**) with quantum yields of *Φ*_fl _= 13% (**3**) and *Φ*_fl _= 6% (**4**), but fluoranthene **2** is nonfluorescent. The weak fluorescence may be attributed to the low oscillator strengths of the S_0_→S_1_ transitions in fluoranthene **2** (*f *= 0.006) and helicene **4** (*f *= 0.009).^[^ ^41^ ^]^

The electrochemical properties of compounds **2**–**4** were investigated by cyclic voltammetry (CV, Figure 4e) and differential pulse voltammetry (DPV, see Supporting Information) versus ferrocene/ferrocenium^+^ in THF solution. Fluoranthene **2** shows the first reduction potential at *E*_red_ = ‑1.70 V, while the first half‐wave reduction potentials of compound **3** (‐2.04 V) and helicene **4** (‐2.35 V) are lower. From the first reduction potentials the electron affinities were estimated by the commonly used expression *EA* = ‐(*E*_red1 _+ 4.8) eV. The electron affinities are *EA* = ‐3.1 eV (fluoranthene **2**), ‐2.8 eV (compound **3**), and ‐2.5 eV (helicene **4**), which are in good correlation to the DFT‐calculated LUMO levels (*E*_LUMO_ = ‐2.7 eV (**2**), ‐2.3 eV (**3**), ‐2.0 eV (**4**)). The trend of their electron affinities demonstrates that an *aza*‐fluoranthene motif is more electron‐deficient than a benzoquinoline unit.

**Figure 5:**
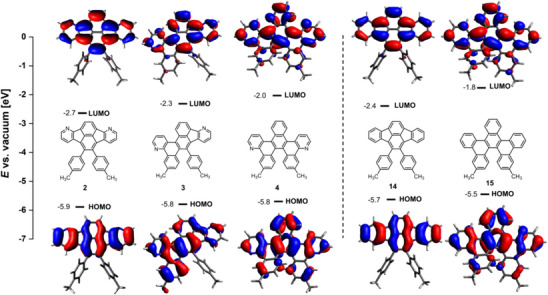
HOMO and LUMO energies of **2**–**4** and all‐carbon analogues **14** and **15** (DFT: B3LYP/6–311g(d,p)). Isovalue = 0.026. See Supporting Information for the illustrations of orbitals HOMO‐2 to LUMO + 1 of **2**–**4** and **14**–**15**.

The electron affinity of fluoranthene **2**, which is the most electron deficient compound in this series, is comparable to naphthalimide‐substituted fluoranthene (‐3.1 eV),^[^ ^23^ ^]^ nitrogen‐doped dibenzo‐acenaphtho‐fluoranthenes (DBAFs, ‐3.0 – ‐3.2 eV),^[^ ^7^ ^]^ quinoxalino‐phenanthrophenazines (QPPs, ‐2.8 – ‐3.4 eV),^[^ ^57^, ^58^, ^59^ ^]^ and other fluoranthene‐based electron‐poor molecules that are of potential interest as organic semiconductors in organic electronics.^[^ ^12^, ^60^ ^]^ DFT calculations of the all‐carbon analogues of fluoranthene **2** (**14**) and helicene **4** (**15**) revealed that nitrogen substitution leads to a lowering of the frontier orbital energies by up to ‐0.3 eV (Figure 5 and Supporting Information).

### 2.3 Single‐Crystal X‐Ray Structure Analysis

**Figure 6:**
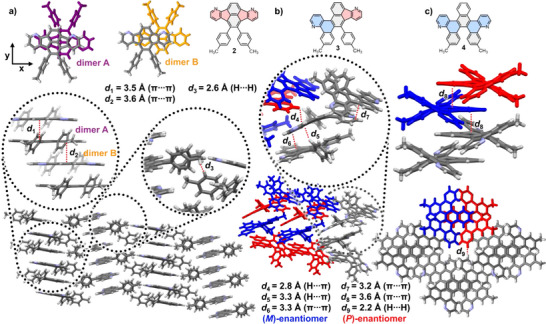
Single crystal X‐ray structures of compounds **2** a), **3** b), and **4** c). The dimers composing the π‐stacks in **2** are shown in purple (dimer A, x_offset,A_ = 1.3 Å and y_offset,A_ = 0.2 Å) and yellow (dimer B, x_offset,B_ = 3.1 Å and y_offset,B_ = 0.1 Å), respectively. Enantiomers of **3** and **4** are shown in red (P‐enantiomer) and blue (M‐enantiomer). Relevant short contacts are highlighted in the insets. Color code C: gray, H: white, N: violet.

Single crystals suitable for X‐ray diffraction of compounds **2**–**4** were obtained by liquid–liquid diffusion of *n*‐hexane into saturated solutions of dichloromethane (for **3** and **4**) or dichloromethane/EtOAc (**2**) (see Figure 6 and Tables S4‐S6 for more information). Single crystals of fluoranthene **2** were also obtained by vacuum sublimation (*T* = 270 °C, *p* = 9 × 10^−3 ^mbar) having the same unit cell as found with the other method.

Fluoranthene **2** forms two types of slipped π‐stacked dimers. In each dimer, the molecules adopt an antiparallel arrangement. These dimers have similar stacking distances of *d*_1_ = 3.5 Å (dimer A) and *d*_2_ = 3.6 Å (dimer B), but different 1D offsets of x_A _= 1.3 Å (dimer A) and x_B _= 3.1 Å (dimer B). In contrast, the offsets along the y‐direction are comparably small in both dimers (y_A _= 0.2 Å, y_B _= 0.1 Å). Dimers A and B assemble in an alternating pattern, resulting in slipped π‐stacked molecular columns propagating along the crystallographic *a*‐axis.

Adjacent columns interact with each other by dispersion interactions among their tolyl‐group hydrogens (*d*_3_ = 2.6 Å), resulting in a γ‐coronene‐type packing motif that incorporates both herringbone and sandwich‐type stacking modes.^[^ ^61^, ^62^ ^]^ Crystals of compound **3** contain both *M*‐ and *P*‐enantiomers, which assemble to enantiopure, 1D threads along the crystallographic *a*‐axis, driven by edge‐to‐face π‐stacking (*d*_4 _= 2.8 Å). Along the crystallographic *b*‐axis, these enantiopure threads interact with the opposite enantiomer by edge‐to‐face π‐stacking (*d*_5_ = 3.3 Å), as well as by face‐to‐face π‐stacking among fluoranthene units (*d*_6_ = 3.3 Å) or between fluoranthene and benzoquinoline units (*d*_7_ = 3.2 Å). The crystal packing of helicene **4** is primarily driven by face‐to‐face π‐stacking (*d*_8_ = 3.6 Å) between the naphthalene units of neighboring pentahelicene molecules. The π‐stacks propagate along the crystallographic *c*‐axis and contain *P*‐ and *M*‐enantiomers in an alternating pattern. Adjacent π‐stacks are formed by dispersion interaction of hydrogen atoms of the quinoline units (*d*_9_ = 2.2 Å).

By DFTB calculations, the charge transfer (CT) integrals were calculated for compounds **2**–**4** in their crystalline forms (see Supporting Information). Dimer A (fluoranthene **2**) shows preferential n‐type semiconducting properties, with CTs for hole transport of *t*_h_ = 54 ± 78 meV, and *t*_e_ = 110 ± 54 meV for electron transport. The latter is in the same regime as *t*_e_‐values of benchmark n‐type semiconducting materials like rubrene (*t*_e_ = 83 meV) or pentacene (*t*_e_ = 75 meV).^[^ ^63^, ^64^ ^]^ In contrast, dimer B of fluoranthene **2** shows preferential p‐type semiconducting properties. CT for electron transport is with *t*_e_ = 19 ± 23 meV relatively low, but CT for hole transport is with *t*_h_ = 102 ± 71 meV relatively high; comparable to that of a p‐type semiconducting material like DBAF (*t*_h_ = 70 ± 24 meV).^[^ ^7^ ^]^ However, CV of fluoranthene **2** revealed multiple reduction potentials but no low‐lying oxidation potential, indicating preferential electron over hole transport. Fluoranthene **2** shows significantly lower CT integrals along direction *d*_3_ (*t*_e,h_ < 1 meV) compared to *d*_1_ or *d*_2_. Thus, charge transport can be assumed to take place only along the π‐stacks. The CT integrals between two aza‐fluoranthenes (*d*_6_) in compound **3** are *t*_e_ = 13 ± 11 meV for electron transport and *t*_h_ = 26 ± 17 meV for hole transport. In comparison, the CT‐integral values among aza‐fluoranthene and benzoquinoline units (*d*_7_) in compound **3** are slightly higher (*t*_e_ = 23 ± 22 meV, *t*_h_ = 36 ± 16 meV), while for both cases hole transport is preferred over electron transport. In contrast, pentahelicene **4** shows similar CT‐values for electron transport (*t*_e_ = 21 ± 22 meV) and hole transport (*t*_h_ = 14 ± 32 meV) along direction *d*_8_.

### Reaction Mechanism

Würthner and coworkers made similar observations of base‐selective five‐ or six‐membered ring formation by using either organic (DBU) or inorganic bases (e.g., carbonates, phosphates) for a palladium‐catalyzed synthesis of polycyclic aromatic dicarboximides (PADIs) by Suzuki–Miyaura and Heck‐type cascade‐reaction.^[^ ^41^ ^]^ They proposed that the switch of selectivity is due to a switch of reaction mechanisms for the carbopalladation step. For Pd‐catalyzed PADI synthesis, carbonate bases led to benzannelation at the position of higher C═C double bond character. Therefore, the carbopalladation with carbonate base was assumed to occur by a Heck‐type reaction pathway, followed by base‐assisted E_2_ or anti‐β‐H elimination in the final catalytic step. In contrast, DBU promoted indenoannelation at the more aromatic position, likely via a concerted metalation deprotonation (CMD‐) or S_E_Ar‐type pathway, followed by reductive elimination in the last catalytic step.^[^ ^41^ ^]^ From a phenomenological point of view, we have made the same observations with respect to base‐selective ring annelation, but in contrast to the Würthner group, we observed indenoannelation at the position of higher double‐bond character (5‐ and 8‐position of naphthalene unit) with DBU.^[^ ^65^ ^]^

**Scheme 4:**
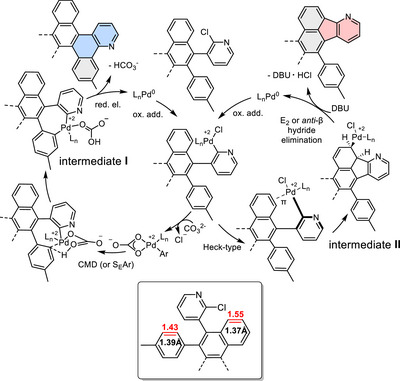
Proposed catalytic cycle of the palladium‐catalyzed C–H activation with DBU. Inset shows Wiberg bond order (red) according to DFT (B3LYP/6–311g(d,p)) and C–C bond lengths (black) according to the single‐crystal structure of chloropyridine **1**.

With K_2_CO_3_, benzannelation occurs at the more aromatic tolyl groups, which is the opposite situation than in the case described by Würthner et al. In our case, the benzannelation pathway with K_2_CO_3_ is assumed to proceed by a CMD mechanism, in which the base that is coordinated to the Pd‐center abstracts a proton, while the Pd–carbon bond is formed in the same step. Thus, aromatic conjugation in the CMD‐transition state is preserved, leading to a seven‐membered palladacycle intermediate (intermediate **I**, Scheme 4).^[^ ^66^ ^]^ This assumption is experimentally supported by the observation that carbo‐palladation is not only controlled by the choice of base but also by adding PivOH (Table 1, Entry 12),^[^ ^39^, ^42^ ^]^ which is known to support the CMD carbo‐palladation pathway by acting as a proton shuttle via complexation to the palladium.^[^ ^42^, ^67^, ^68^ ^]^ Given the similar coordination properties of carbonate and carboxylate ions, both acting as *κ* ^2^‐ligands,^[^ ^69^ ^]^ the benzannelation pathway is assumed to proceed by a CMD mechanism in both cases.

Since the addition of PivOH promotes the CMD mechanism in the presence of DBU while disfavoring indenoannelation, the latter is assumed to proceed via a different carbopalladation pathway, such as a Heck‐type coupling. According to Würthner and coworkers, the Heck‐type carbo‐palladation is more likely to proceed with increasing double bond character.^[^ ^70^ ^]^ They reported that the double bond character may be estimated by the Wiberg bond order, which should exceed a value of 1.5 for the Heck‐type coupling to be feasible. Single‐crystal X‐ray diffraction analysis and DFT calculations of compound **1** (see Supporting information) reveal a more pronounced double bond character in the naphthyl unit compared to the tolyl group, as evidenced by a higher Wiberg bond order (1.546 vs. 1.433, level of theory: B3LYP/6–311g(d,p)) and a shorter C═C bond length in the naphthyl moiety (1.37 Å vs. 1.39 Å, Scheme 4). Consequently, the carbo‐palladation at the naphthyl unit of **1** is assumed to proceed via a Heck‐type coupling mechanism (intermediate **II**, Scheme 4).

In contrast to the mechanisms proposed by Würthner for PADI synthesis, where indenoannelation proceeds via CMD (or S_E_Ar) and benzannelation via Heck‐type coupling, the situation here is the opposite: benzannelation of **1** occurs through CMD (or S_E_Ar) and indenoannelation via a Heck‐type coupling.

**Figure 7:**
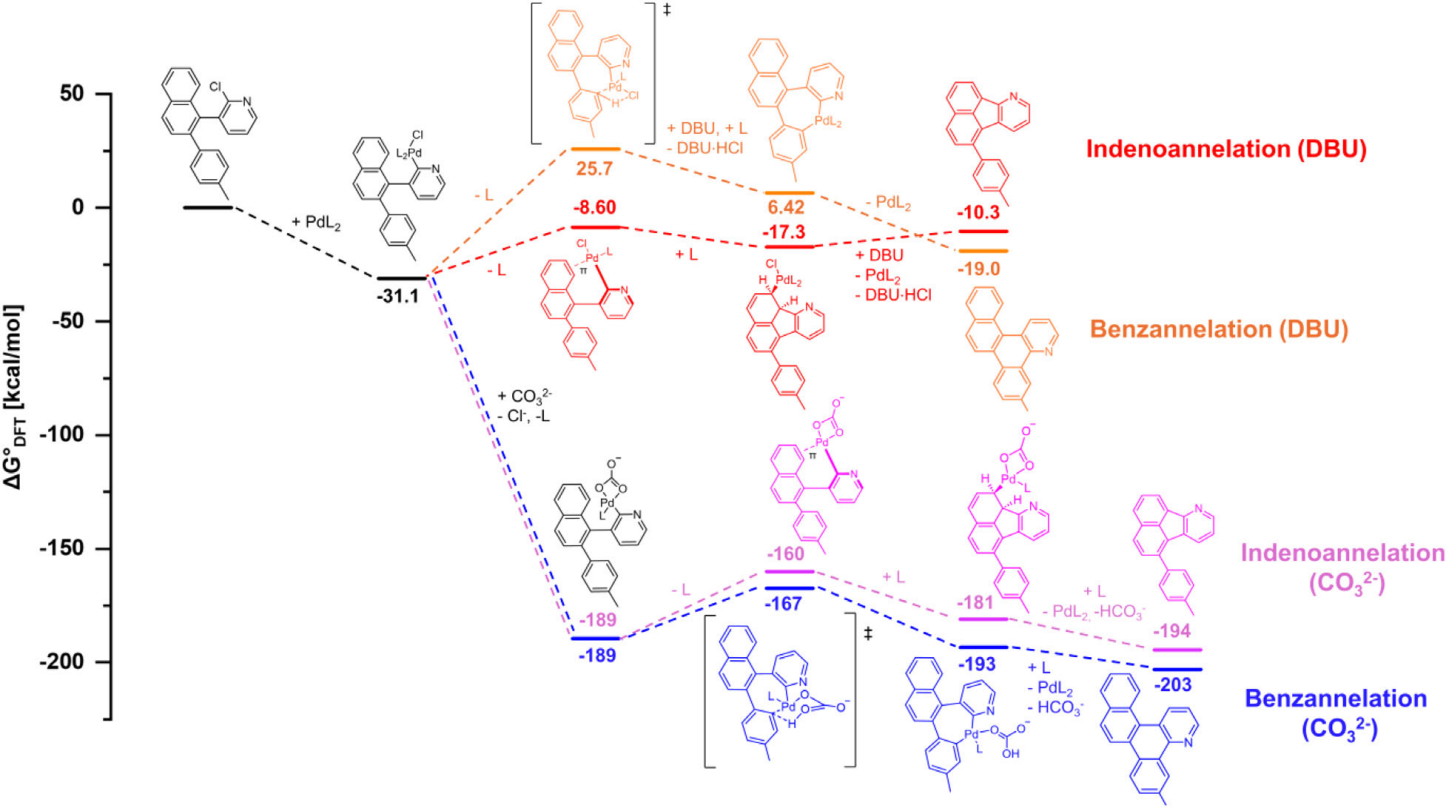
DFT‐calculated mechanisms (B3LYP/6–311g(d)/LANL2DZ/GD3BJ, *T* = 298 K) for the Pd‐catalyzed benzannelation and indenoannelation pathways with DBU and carbonate bases, respectively. L = PMe_3_.

To understand the observed base‐selectivity, both reaction pathways to the indeno‐ and benzannelation were calculated by DFT on the B3LYP/6‐311g(d)/LANL2DZ level of theory with a GD3BJ dispersion correction (Figure 7, see Supporting Information for more information).^[^ ^71^, ^72^, ^73^, ^74^, ^75^ ^]^ Both pathways were calculated with DBU or carbonate as base, respectively. Carbonate was treated as *κ* ^2^‐ligand, while DBU was treated as noncoordinating base in solution that neutralizes the HCl formed in the catalytic cycle. Irrespective of the base, the benzannelated product is favored by Δ*G*° = ‐9 kcal/mol over the indenoannelated product, which corroborates to our findings.

When DBU is used, the CMD transition state of the benzannelation pathway (Δ*G*° = 25.7 kcal/mol) is disfavored by Δ*G*° = 34.3 kcal/mol over the Pd‐π complex (Δ*G*° = ‐8.60 kcal/mol) that leads to indenoannelation. Thus, indenoannelation is kinetically favored in the DBU reaction pathway, whereas benzannelation is thermodynamically favored. In contrast, when carbonate is used instead of DBU, the CMD transition state (Δ*G*° = ‐167 kcal/mol) is energetically favored by Δ*G*° = ‐7 kcal/mol over the Pd‐π complex (Δ*G*° = ‐160 kcal/mol). Therefore, when a carbonate base is used, benzannelation is both kinetically and thermodynamically favored over indenoannelation. Moreover, formation of the *κ* ^2^‐Pd‐O chelate complex (Δ*G*° = ‐189 kcal/mol) with carbonate is strongly favored over the Pd‐Cl complex (Δ*G*° = ‐31.1 kcal/mol) by Δ*G*°= ‐158 kcal/mol. Thus, in the presence of both DBU and carbonate, the reaction mixture can be expected to form predominantly benzannelated product (Table 1, Entry 14).

## 3 Conclusion

In summary, conditions were elaborated for selective pent‐ or hexaannulation of a chloropyridine precursor to an *aza*‐indeno‐*aza*‐fluoranthene or benzo[*h*]quinoline. This was achieved by controlling the Pd‐catalyzed C–H activation reaction through various parameters, such as base, solvent, additive, and temperature. With K_2_CO_3_ pentahelicene **4** was formed in 74% yield exclusively, while with DBU as base the *aza*‐indenofluoranthene **2** was the main product in 53% yield. Thus, the underlying annulation was controlled by exchanging the base. It is worth to mention that the mechanistic pathways are the opposite of those proposed for PADI synthesis, where it is suggested that indenoannelation proceeds via CMD (or S_E_Ar) and benzannelation via Heck‐type coupling, because the situation is here the opposite: benzannelation of **1** most likely occurs through CMD (or S_E_Ar) and indenoannelation via a Heck‐type coupling.

All products show interesting optoelectronic properties as electron acceptors. The crystal packing of fluoranthene **2**, in combination with their high charge‐transfer integrals for electron transport, suggests their potential application as an n‐type semiconductor material in organic electronics.

## Supporting Information

The authors have cited additional references within the Supporting Information.^[^ ^48^, ^49^, ^50^, ^51^, ^76^, ^77^, ^78^, ^79^, ^80^, ^81^, ^82^, ^83^, ^84^, ^85^, ^86^, ^87^, ^88^, ^89^, ^90^, ^91^, ^92^, ^93^, ^94^, ^95^, ^96^, ^97^, ^98^, ^99^, ^100^, ^101^, ^102^, ^103^, ^104^ ^]^ Deposition numbers that contain supplementary crystallographic data can be found at the end of the document.^[^ ^105^ ^]^

## Conflict of Interest

The authors declare no conflict of interest.

## Supporting information



Supporting Information

Supporting Information

## Data Availability

The data that support the findings of this study are available from the corresponding author upon reasonable request.
