# Supplementary material for: Synthesis of Aza‐indeno‐aza‐fluoranthene and Constitutional Isomers by Ring‐Size Selective C–H Activation
Source: Chemistry. 2025 Nov 11;31(72):e02960. doi: 10.1002/chem.202502960 (PMC12731527; doi:10.1002/chem.202502960)
Supplement: Supplementary file 1 — Supporting Information [file CHEM-31-e02960-s001.docx]

**Supporting Information**

Synthesis of *Aza*-Indeno-*Aza*-Fluoranthene and Constitutional Isomers by Ring-Size-Selective C–H Activation

Christoph Keck,^[a]^ Frank Rominger,^[a]^ Sonali Garg,^[b]^ Marcus Elstner^[b]^ and Michael Mastalerz*^[a]^

[a] Christoph Keck, Dr. Frank Rominger, Prof. Dr. Michael Mastalerz
Organisch-Chemisches Institut, Ruprecht-Karls-Universität Heidelberg, Im Neuenheimer Feld 270, 69120 Heidelberg (Germany)
E-mail: [michael.mastalerz@oci.uni-heidelberg.de](mailto:michael.mastalerz@oci.uni-heidelberg.de)

[b] Sonali Garg, Prof. Dr. Marcus Elstner

Institut für Physikalische Chemie und Theoretische Chemische Biologie, Karlsruher Institut für Technologie (KIT), Kaiserstraße 12, 76131 Karlsruhe (Germany)

**Table of Contents**

[General Remarks 1](#_Toc212014858)

[1 Synthetic Procedures 2](#_Toc212014859)

[2-Chloro-3-(tosylmethyl)pyridine (**8**) and 1,3-Bis(2-chloropyridin-3-yl)propan-2-one (**7**) (Route A) 2](#_Toc212014860)

[2-(2-Chloropyridin-3-yl)acetic acid (**10**) 3](#_Toc212014861)

[1,3-Bis(2-chloropyridin-3-yl)propan-2-one (**7**) 3](#_Toc212014862)

[2,5-Bis(2-chloropyridin-3-yl)-3,4-di-*p*-tolyl-cyclopentadienone (**12**) 4](#_Toc212014863)

[3,3'-(2,3-Di-*p*-tolylnaphthalene-1,4-diyl)bis(2-chloropyridine) (**1**) 4](#_Toc212014864)

[Screening Experiments of the Pd-Catalyzed Direct Arylation 5](#_Toc212014865)

[Optimized Reaction Conditions for the Pd-Catalyzed Indenoannelation of **1** 6](#_Toc212014866)

[Optimized Reaction Conditions for the Pd-Catalyzed Benzannelation of **1** 7](#_Toc212014867)

[2 Spectra 8](#_Toc212014868)

[^1^H and ^13^C NMR Spectra 8](#_Toc212014869)

[HR-MS Spectra 32](#_Toc212014870)

[IR Spectra 35](#_Toc212014871)

[UV/Vis Spectra 37](#_Toc212014872)

[UV/Vis Spectra of Fluoranthene **2** in Different Solvents 39](#_Toc212014873)

[CV and DPV 39](#_Toc212014874)

[Crystal Structure Data 41](#_Toc212014875)

[3 Calculations 46](#_Toc212014876)

[Kohn-Sham Molecular Orbitals 46](#_Toc212014877)

[UV/Vis Absorption Spectra (TD-DFT) 47](#_Toc212014878)

[NICS(0) & NICS(1) 50](#_Toc212014879)

[Charge-Transfer Integrals 51](#_Toc212014880)

[Calculation of One-Dimensional Offsets in Crystal-Packing of Fluoranthene **2** 51](#_Toc212014881)

[Reaction Mechanism 52](#_Toc212014882)

[4 References 68](#_Toc212014883)

## General Remarks

**Materials:** All reagents and solvents were obtained from abcr, Acros Organics, Alfa Aesar, Fluorochem, Merck, SigmaAldrich or VWR International and were used without further purification. Anhydrous solvents were obtained commercially or taken from a solvent purification system (MBraun SPS-800). For thin layer chromatography *TLC silica gel 60 F_254_* plates from Merck were used and examined under UV-light irradiation (254 nm and 365 nm). Flash column chromatography was performed on silica gel from Macherey Nagel (particle size: 0.04-0.063 mm) using ethyl acetate (EA), dichloromethane (DCM) and light petroleum ether (PE). **Melting points (m.p.):** Melting points were measured on a Büchi m-565 melting point instrument. **Nuclear magnetic resonance spectroscopy (NMR):** NMR spectra were recorded on a Bruker Avance 300 (300 MHz), a Bruker Avance III 600 (600 MHz) or a Bruker Avance Neo 700 (700 MHz) spectrometer. Chemical shifts (δ) are reported in parts per million (ppm) relative to traces of CHCl_3_ or CHDCl_2_ in the corresponding deuterated solvent (CDCl_3_: *δ*_H_ = 7.26 ppm, *δ*_C_ = 77.16 ppm; CD_2_Cl_2_: *δ*_H_ = 5.32 ppm, *δ*_C_ = 53.84 ppm).^[S1]^ Signals were assigned by 2D NMR Experiments (^1^H-^13^C-HSQC, ^1^H-^13^C-HMBC, ^1^H-^1^H-COSY, ^1^H-^1^H-NOESY)**.**  **Infrared spectroscopy (IR):** Infrared spectroscopy was conducted using a Bruker Fourier transform infrared spectrometer Tensor 27 either with a ZnSe crystal (ATR) or freshly prepared KBr discs. **UV/Vis absorption spectroscopy (UV/Vis) and fluorescence spectroscopy:** Absorption spectra were recorded on a Jasco V-730-ST UV/vis spectrometer and emission spectra were recorded on a Jasco FP-8300 fluorescence spectrometer. Absolute photoluminescence quantum yields (Φ_fl_) were measured in chloroform solution (OD < 0.1) at room temperature using a JASCO FP-8500 fluorescence spectrometer with a JASCO ILF-835 (100 mm) integrating sphere. **Electrochemistry:** Cyclic voltammetry (CV) and differential pulse voltammetry (DPV) spectra were recorded on a Metrohm Autolab PGSTAT101 potentiostat using a Pt working electrode (0.78 mm^2^), a Pt counter electrode and an Ag/Ag^+^ pseudo reference electrode in degassed HPLC-grade THF. Ferrocene was used as an internal standard for calibration. CV spectra were obtained at a scan rate of 0.1 Vs^-1^ and DPV spectra were obtained with a step size of 0.005 V, a modulation amplitude of 0.025 V a modulation time of 0.05 s and an interval time of 0.5 s. **Mass spectrometry (MS):** Mass spectrometry was performed on a Bruker AutoFlex Speed time-of-flight spectrometer (MALDI-TOF) or Bruker timsTOFfleX spectrometer using DCTB (trans-2-[3-(4-tert-Butylphenyl)-2-methylpropenylidene)malononitrile) as matrix. **Elemental analysis:** The elemental analyses were performed by the microanalytical laboratory of University of Heidelberg using a vario MICRO cube device purchased from *Elementar*. **Computational details:** Kohn-Sham molecular orbitals were computed by DFT-geometry-optimized crystal-structure geometries using the Gaussian16 program package.^[S2]^ The theoretical approach is based on Kohn-Sham density functional methodologies,^[S3-6]^ using the B3LYP-functional^[S7-10]^ in combination with the 6-311g(d,p)-basis set.^[S11-12]^ Molecular orbitals were visualized using Avogadro^[S13]^ (Version 1.2) at an isosurface value of 0.026. Spectra were computed by TD-DFT^[S14-20]^ in CHCl_3_ based on DFT-geometry-optimized crystal-structure geometries on the same level of theory (B3LYP/6-311g(d,p)). The TD-DFT calculated data was processed with GaussSum^[S21]^ and the respective output data was used for plotting the simulated UV/Vis absorption spectra. NICS(0) and NICS(1) values were calculated from the optimized geometries by adding a ghost atom in the center or 1 Å above/below of the corresponding ring and performing a single-point calculation based on Hartree-Fock^[S22]^ with the GIAO-method.^[S23-27]^ Wiberg bond-orders were calculated by NBO analysis (Gaussian 16, theory level: B3LYP/6-311g(d,p)) using DFT-optimized geometries at the same level of theory. Some grammatical and spelling corrections were performed with the assistance of ChatGPT.^[S28]^ **X-ray crystal structure analysis:** X-ray crystal diffractograms were recorded with a Bruker APEX-II Quazar diffractometer using Mo-Kα radiation (λ = 0.71073 Å) or a STOE Stadivari diffractometer using Cu-Kα radiation (λ = 1.54178 Å). Intensities were corrected for Lorentz and polarization effects, an empirical scaling and absorption correction was applied using SADABS^[S29]^ based on the Laue symmetry of the reciprocal space (*μ*, *T*_min_, *T*_max_). The structures were solved with SHELXT-2014/2 (Sheldrick 2015)^[S30]^ and refined with a full-matrix least-squares algorithm using the SHELXL-2019/2 (Sheldrick, 2019) software.^[S31]^

# 1 Synthetic Procedures

Chloromethyl-chloropyridine (**6**) was synthesized according to a literature-known procedure.^[S32]^

### **2-Chloro-3-(tosylmethyl)pyridine (8) and** 1,3-Bis(2-chloropyridin-3-yl)propan-2-one (7) (Route A)

NaH (592 mg (60w% dispersion in mineral oil), 14.8 mmol) was suspended in dry DMSO (10 mL) under argon atmosphere at 0 °C. A solution of chloropyridine **6** (2.00 g, 12.3 mmol) and TosMIC (1.16 g, 5.94 mmol) in dry DMSO (10 mL) was slowly added within 30 min. After the addition was completed, the reaction mixture was stirred at room temperature for 20 h. The reaction was cooled to 0 ^o^C and water (60 mL) and DCM (30 mL) added. The phases were separated and the aqueous phase was extracted with DCM (2 x 30 mL). The combined organic phases were washed with sat. NaHCO_3_-solution (2 x 50 mL) and dried over Na_2_SO_4_. The solvent was removed in vacuo to give a brown oil that was dissolved in Et_2_O (30 mL). Concentrated HCl_aq_ (3 mL) was added and the mixture stirred at room temperature for 1 h. Water (30 mL) and DCM (30 mL) were added and mixture was neutralized using solid Na_2_CO_3_. The phases were separated and the aqueous phase was extracted with DCM (2 x 30 mL). The combined organic phases were dried over Na_2_SO_4_ and the solvent was removed *in vacuo*. The residue was purified by column chromatography (SiO_2_, PE/EA 1:1, *R*_f_ = 0.30, 0.05) to give tosylmethyl-chloro-pyridine **8** (*R*_f_ = 0.30) as a colorless solid (484 mg, 14%) and ketone **7** (*R*_f_ = 0.05) as a colorless solid (198 mg, 6%).

Analytical data of 2-chloro-3-(tosylmethyl)pyridine (compound **8**): ***R*_f_** (SiO_2_, PE/EA 1:1) = 0.30. **m.p.** 120–121.5 °C. **^1^H NMR** (600 MHz, CD_2_Cl_2_, 295 K, ppm): *δ* = 8.32 (dd, J = 4.8, 1.9 Hz, 1*H*, *H*-4), 7.78 (dd, J = 7.6, 1.9 Hz, 1*H*, *H*-6), 7.52–7.47 (m, 2*H*, *H*-2’), 7.31–7.25 (m, 3*H, H*-3’ & *H*-5), 4.49 (s, 2*H, H*-1), 2.41 (s, 3*H*, C*H*_3_).**^13^C NMR** (151 MHz, CDCl_3_, 295 K, ppm): *δ* = 152.5 (*C*-3), 150.3 (*C*-4), 146.0 (*C*-4’), 141.9 (*C*-6), 135.4 (*C*-1’), 130.2 (*C*-3’), 128.9 (*C*-2’), 124.4 (*C*-2), 123.1 (*C*-5), 59.3 (*C*-1), 21.8 (*C*H_3_). **FT-IR** (ATR, cm^-1^): $\tilde{\text{ν}}$ = 3070 (w), 2989 (vw), 2920 (w), 1294 (s), 1412 (s), 1306 (s). 1294 (s), 1134 (s), 1121 (s), 1157 (s), 1088 (s), 1065 (s), 876 (m), 812 (s), 733 (vs), 689 (m). **HR-MS** (EI): *m/z*calcd for C_13_H_12_ClNO_2_S^+^: 281.0272 [*M*]^+^; found (%): 281.0271 (10) [*M*]^+^, 126.0106 (100) [*M*-C_7_H_7_SO_2_]^+^. **Elemental analysis** calcd for C_13_H_12_ClNO_2_S: C 55.42, H 4.29, N 4.97, found: C 55.45, H 4.45, N 4.94.

Analytical data of 1,3-Bis(2-chloropyridin-3-yl)propan-2-one (compound **7**):***R*_f_** (SiO_2_, PE/EA 1:1) = 0.05. **m.p.** 164–165 °C. **^1^H NMR** (600 MHz, CDCl_3_, 295 K, ppm): *δ* = 8.31 (dd, *J* = 4.8, 1.9 Hz, 2*H, H*-5), 7.59 (dd, *J* = 7.5, 1.9 Hz, 2*H*, *H*-7), 7.26 (dd, *J* = 7.5, 4.7 Hz, 2*H*, *H*-6), 4.00 (s, 2*H*, *H*-2). **^13^C NMR** (151 MHz, CDCl_3_, 295 K, ppm): *δ* = 201.4 (*C*-1), 151.9 (*C*-4), 149.0 (*C*-5), 140.9 (*C*-7), 129.5 (*C*-3), 123.1 (*C*-6), 47.2 (*C*-2). **FT-IR** (ATR, cm^-1^): $\tilde{\text{ν}}$ = 3323 (vw), 3042 (vw), 2930 (w), 2905 (w), 2851 (w), 1728 (m), 1582 (m), 1566 (s), 1414 (s), 1335 (m), 1313 (m), 1070 (s), 829 (m), 808 (vs), 760 (m), 739 (m), 700 (m), 671 (s). **HR-MS** (APCI): *m/z* calcd for C_13_H_10_Cl_2_N_2_O^+^: 281.0243 [*M*]^+^; found: 281.0233. **Elemental analysis** calcd for C_13_H_10_Cl_2_N_2_O: C 55.54, H 3.59, N 9.96, found: C 55.79, H 3.84, N 10.08.

### 2-(2-Chloropyridin-3-yl)acetic acid (10)

2-(2-Chloropyridin-3-yl)acetonitrile (5.00 g, 32.8 mmol) was suspended in an aqueous sodium hydroxide solution (75 mL, 15 wt%) and stirred under reflux for 2 h. The solution was cooled to 0 °C and acidified to pH = 1 by dropwise addition of concentrated hydrochloric acid. The resulting white suspension was left to stand for 1 h in an ice-water bath. The precipitate was collected by filtration and washed with cold water to give the product as a colorless solid (4.81 g, 85%).

**m.p.** 198–200 °C. **^1^H NMR** (600 MHz, DMSO-*d*_6_, 295 K, ppm): *δ* = 12.64 (s, 1H, COO*H*), 8.32 (dd, *J* = 4.8, 2.0 Hz, 1*H*, *H*-5), 7.86 (dd, *J* = 7.6, 2.0 Hz, 1*H*, *H*-7), 7.41 (dd, *J* = 7.5, 4.7 Hz, 1*H*, *H*-6), 3.75 (s, 2*H*). **^13^C NMR** (151 MHz, CDCl_3_, 295 K, ppm): *δ* = 171.1 (*C*-1), 150.9 (*C*-4), 148.3 (*C*-5), 141.2 (*C*-7), 130.2 (*C*-3), 123.3 (*C*-6), 38.2 (*C*-2). **FT-IR** (KBr disc, cm^‑1^): $\tilde{\text{ν}}$ = 3651 (w), 2931 (w), 1698 (m), 1407 (s), 1170 (vs), 1093 (vs), 756 (s). **HR-MS** (EI): *m/z* calcd for C_7_H_6_ClNO_2_^+^: 171.0082 [*M*]^+^; found (%): 171.0095 (20) [*M*]^+^, 136.0414 (90) [*M*-Cl]^+^, 91.0425 (100) [*M*-Cl-COOH]^+^. **Elemental analysis** calcd. for C_7_H_6_ClNO_2_: C 49.00, H 3.52, N 8.16, found: C 48.67, H 3.72, N 8.18.

### 1,3-Bis(2-chloropyridin-3-yl)propan-2-one (7)

*N*,*N*′-Dicyclohexylcarbodiimide (628 mg, 3.05 mmol) and 4-(dimethylamino)-pyridine (84.6 mg, 692 µmol) were dissolved in dry DCM (2.5 mL) under argon atmosphere. A solution of carboxylic acid **10** (475 mg, 2.77 mmol) in dry DCM (9 mL) was added at room temperature, forming first a yellow solution and then a colorless precipitate. The reaction mixture was stirred at room temperature for 19 h, and the precipitate collected by filtration and washed with DCM (120 mL). The solvent was removed in vacuo, giving a crude yellow product, that was purified by column chromatography (SiO_2_, PE/EA 1:2, *R*_f_ = 0.38) to give ketone **7** as a colorless solid (363 mg, 93%). **m.p.** 164–165 °C. **^1^H NMR** (600 MHz, CDCl_3_, 295 K, ppm): *δ* = 8.31 (dd, *J* = 4.8, 1.9 Hz, 2*H, H*-5), 7.59 (dd, *J* = 7.5, 1.9 Hz, 2*H*, *H*-7), 7.26 (dd, *J* = 7.5, 4.7 Hz, 2*H*, *H*-6), 4.00 (s, 2*H*, *H*-2). **^13^C NMR** (151 MHz, CDCl_3_, 295 K, ppm): *δ* = 201.4 (*C*-1), 151.9 (*C*-4), 149.0 (*C*-5), 140.9 (*C*-7), 129.5 (*C*-3), 123.1 (*C*-6), 47.2 (*C*-2). **FT-IR** (ATR, cm^-1^): $\tilde{\text{ν}}$ = 3323 (vw), 3042 (vw), 2930 (w), 2905 (w), 2851 (w), 1728 (m), 1582 (m), 1566 (s), 1414 (s), 1335 (m), 1313 (m), 1070 (s), 829 (m), 808 (vs), 760 (m), 739 (m), 700 (m), 671 (s). **HR-MS** (APCI): *m/z* calcd for C_13_H_10_Cl_2_N_2_O^+^: 281.0243 [*M*]^+^; found: 281.0233. **Elemental analysis** calcd. for C_13_H_10_Cl_2_N_2_O: C 55.54, H 3.59, N 9.96, found: C 55.79, H 3.84, N 10.08.

### 2,5-Bis(2-chloropyridin-3-yl)-3,4-di-*p*-tolyl-cyclopentadienone (12)

Ketone **7** (800 mg, 2.85 mmol) and diketone **11** (880 mg, 3.69 mmol) were suspended in dry ethanol (6 mL) under argon atmosphere. DBU (0.45 mL, 3.0 mmol) was added and the reaction mixture was stirred at 78 °C for 7 hours. The solvent was removed under reduced pressure and the residue was purified by column chromatography (SiO_2_, PE/EA 1:1, *R*_f_ = 0.5) to give cyclopentadienone **12** as a red solid (930 mg, 68%). The compound was used without further purification in the next step. **m.p.** 125-127 °C. **^1^H NMR** (600 MHz, DMSO-*d*_6_, 295 K, ppm): *δ* = 8.40 (dd, *J* = 5.0, 1.9 Hz, 2H, *H*-6), 7.76 (dd, *J* = 7.0, *H*-8), 7.45 – 7.43 (m, 2H, *H*-7), 7.06 (d, *J* = 8.0 Hz, 4H, *H*-11), 6.78 (d, *J* = 8.0 Hz, 4H, *H*-10), 2.24 (s, 6H, *H*-13). **^13^C NMR** (151 MHz, DMSO-*d*_6_, 295 K, ppm): *δ* =195.7, (*C*-1), 156.3 (*C*-9), 150.2 (*C*-5), 149.5 (*C*-6), 148.1 (*C*-3), 142.0 (*C*-4), 141.7 (*C*-8), 139.5 (*C*-12), 128.8 (*C*-11), 128.6 (*C*-10), 124.2 (*C*-2), 123.0 (*C*-7), 20.9 (*C*-13). **FT-IR** (ATR, cm^-1^): $\tilde{\text{ν}}$ = 2919 (vw), 1704 (s), 1555 (m), 1390 (vs), 1062 (s), 768 (vs). **HR-MS** (EI): *m/z* calcd for C_29_H_20_Cl_2_N_2_O^+^: 482.0947 [*M*]^+^; found (%): 482.0935 (100) [*M*]^+^, 447.1242 (60) [M-Cl]^+^.

### 3,3'-(2,3-Di-*p*-tolylnaphthalene-1,4-diyl)bis(2-chloropyridine) (1)

Cyclopentadienone **12** (620 mg, 1.28 mmol) and CsF (900 mg, 5.93 mmol) were suspended in dry acetonitrile (7.0 mL). TMS-triflate **13** (470 µL, 578 mg, 277 mmol) was added and the reaction mixture was stirred for 19 hours at 60 °C. The black solution was quenched by adding water (30 mL) and DCM (30 mL). The phases were separated, and the aqueous phase was extracted with DCM (2 x 30 mL). The combined organic extracts were dried over anhydrous Na_2_SO_4_ and the solvents were removed under reduced pressure. The crude product was purified by column chromatography (SiO_2_, gradient of DCM/EA 100:0 to 98:2, *R*_f_ = 0.2) to give **1** as an off-white solid (120 mg) in 18% yield. ***R*_f_** (SiO_2_, DCM) = 0.2. **m.p.** 149–151 °C. **^1^H NMR** (600 MHz, CDCl_3_, 295 K, ppm): *δ* = 8.30 (dd, *J* = 4.8, 2.0 Hz, 2H, *H*-10), 7.55 (dd, *J* = 7.5, 2.0 Hz, 2H, *H*-8), 7.49–7.44 (m, 2H, *H*-4), 7.38–7.34 (m, 2H, *H*-5), 7.16 (dd, *J* = 7.5, 4.8 Hz, 2H, *H*-9), 6.95–6.93 (m, 2H, H-12), 6.73–6.71 (m, 2H, *H*-13), 6.71–6.70 (m, 4H, *H*-13’ & *H*-12’), 2.09 (s, 6H, C*H*_3_). **^13^C NMR** (151 MHz, CDCl_3_, 295 K, ppm): *δ* = 151.6 (*C*-6), 148.7 (*C*-10), 141.5 (*C*-8), 140.0 (*C*-11), 136.3 (*C*-1), 135.7 (*C*-14), 135.2 (*C*-2), 134.8 (*C*-7), 131.1 (*C*-3), 130.5 (*C*-13’/*C*-12’), 129.9 (*C*-12), 128.4 (*C*-13), 127.4 (*C*-13’/*C*-12’), 126.9 (*C*-4), 126.2 (*C*-5), 121.9 (*C*-9), 21.2 (*C*H_3_). **FT-IR** (ATR, cm^-1^): $\tilde{\text{ν}}$ = 2952 (w), 2921 (w), 2852 (w), 1557 (m), 1394 (s), 1366 (m), 1093 (m), 1070 (s), 766 (vs), 760 (s). **UV/Vis** (CHCl_3_, nm (M^-1^cm^-1^$\cdot$10^3^), 295 K): *λ*_max_ (*ε*) = 278 (sh, 18.3), 307 (sh, 8690), 306 (62.4). **Fluorescence** (CHCl_3_, 295 K, nm): *λ*_exc_ = 303, *λ*_em_ = 364. **HR-MS** (EI): *m/z* calcd for C_34_H_24_Cl_2_N_2_^+^: 530.1311 [*M*]^+^; found (%): 530.1316 (100) [M]^+^, 495.1660 (80) [M-Cl]^+^. **Elemental analysis** calcd. for C_34_H_24_Cl_2_N_2_⋅¼ ethyl acetate: C 75.95, H 4.73, N 5.06, found: C 76.13, H 5.15, N 5.10.

### Screening Experiments of the Pd-Catalyzed Direct Arylation

In a typical experiment, chloropyridine **1** (25 mg, 47 µmol), bis-(tricyclohexylphosphino)-palladium(II)-dichlorid (7.3 mg, 9.9 µmol, 21 mol%) and a base (0.46 mmol) were dissolved in dry mesitylene or DMAc (2.0 mL) under argon atmosphere and the reaction mixture was stirred at the indicated temperature for 20-24 hours. The reaction mixture was allowed to cool to room temperature, before water (20 mL) and DCM (40 mL) were added. The phases were separated and the organic phase was washed with water (2 x 20 mL) and brine (50 mL). The organic phase was dried over anhydrous Na_2_SO_4_ and the solvents were removed under reduced pressure. The crude product was purified by column chromatography (SiO_2_, gradient of PE/EA 4:1 to 1:2, *R*_f_ (PE/EA 1:1) = 0.15, 0.41, 0.74) to give pentahelicene **4** (*R*_f_ = 0.74) as a yellow solid, compound **3** (*R*_f_ = 0.41) as a yellow solid and fluoranthene **2** (*R*_f_ = 0.15) as an orange solid in the indicated yields (Table S1). All products were analyzed by 1H NMR spectroscopy.

**Table S1**. Screening parameters and isolated yields of the Pd-catalyzed direct arylation of chloropyridine **1**.

| Entry | Base | *T* [°C] | Yield **2** [%] | Yield **3** [%] | Yield **4** [%] |
| --- | --- | --- | --- | --- | --- |
| 1 | K_3_PO_4_ | 160 | 0 | 0 | 16 |
| 2 | LiHMDS | 160 | 0 | 0 | 44 |
| 3 | K_2_CO_3_ | 140 | 0 | 0 | 26 |
| 4 | K_2_CO_3_ | 160 | 0 | 0 | 45 |
| 5^[a]^ | K_2_CO_3_ | 160 | 0 | 0 | 51 |
| 6^[b]^ | K_2_CO_3_ | 160 | 0 | 0 | 72 |
| 7^[c]^ | K_2_CO_3_ | 160 | 0 | 0 | **74** |
| 8 | DBU | 140 | 32 | 14 | 10 |
| 9 | DBU | 160 | **53** | 10 | 3 |
| 10 | DBU | 180 | 22 | 14 | 10 |
| 11 | DBU | 200 | 14 | 15 | 10 |
| 12^[d]^ | DBU | 160 | 5 | 20 | 51 |
| 13^[c]^ | DBU | 160 | 42 | 29 | 23 |
| 14 | DBU/ K_2_CO_3_ | 160 | 0 | 0 | 49 |
| 15 | DIPEA | 160 | 0 | 0 | 0 |

[a] Increased reaction time of 48 h. [b] Increased catalyst loading (40 mol% PdCl_2_(PCy_3_)_2_). [c] Solvent: mesitylene instead of DMAc. [d] Additive: PivOH (40 mol%).

### Optimized Reaction Conditions for the Pd-Catalyzed Indenoannelation of 1

Chloropyridine **1** (33.0 mg, 62.1 µmol) and bis-(tricyclohexylphosphino)-palladium(II)-dichloride (9.5 mg, 12.9 µmol, 20 mol%) were dissolved in dry DMAc (2.0 mL) under argon atmosphere. DBU (90 µL, 0.60 mmol) was added and the reaction mixture was stirred at 160 °C for 24 hours. The reaction mixture was cooled to room temperature, water (20 mL) and DCM (40 mL) were added and the phases were separated. The organic phase was washed with water (2 x 20 mL) and brine (50 mL). The organic phase was dried over anhydrous Na_2_SO_4_ and the solvents were removed under reduced pressure. The crude product was purified by column chromatography (SiO_2_, gradient of PE/EA 4:1 to 1:2, *R*_f_ (PE/EA 1:1) = 0.15, 0.41, 0.74) to give pentahelicene **4** (*R*_f_ = 0.74) as a yellow solid (0.7 mg, 3%), compound **3** (*R*_f_ = 0.41) as a yellow solid (2.8 mg, 10%) and fluoranthene **2** (*R*_f_ = 0.15) as an orange solid (15 mg, 53%).

*Aza*-indeno-*aza*-fluoranthene (**2**): ***R*_f_** (SiO_2_, PE/EA 1:1) = 0.15 (yellow). **m.p.** 298-300 °C. **^1^H NMR** (600 MHz, CDCl_3_, 295 K, ppm): *δ* = 8.27 (dd, *J* = 5.0, 1.5 Hz, 2*H, H*-6), 8.00 (s, 2*H, H*-8), 7.11–7.09 (m, 4*H, H*-11), 7.08–7.06 (m, 4*H, H*-10), 6.99 (dd, *J* = 7.7, 1.5 Hz, 2*H, H*-4), 6.81 (dd, *J* = 7.7, 5.0 Hz, 2*H, H*-5), 2.37 (s, 6H, C*H*_3_). **^13^C NMR** (151 MHz, CDCl_3_, 295 K, ppm): *δ* = 162.3 (*C*-7), 147.9 (*C*-6), 141.1 (*C*-9), 137.5 (C-2a’), 137.4 (*C*-2a), 137.3 (*C*-12), 134.7 (*C*-1/*C*-2), 133.0 (*C*-1/*C*-2), 131.2 (C-7a), 130.8 (*C*-4), 130.0 (*C*-10), 128.9 (*C*-11), 123.6 (*C*-8), 121.9 (*C*-5), 21.5 (*C*H_3_). **FT-IR** (ATR, cm^-1^): $\tilde{\text{ν}}$ = 2955 (m), 2924 (s), 2853 (s), 1460 (m), 1364 (s), 1186 (s), 1082 (s), 966 (vs), 768 (vs). **UV/Vis** (CHCl_3_, nm (M^-1^cm^-1^$\cdot$10^3^) 295 K): *λ*_max_ (*ε*) = 397.2 (14.6), 376.8 (12.3), 306 (62.4). **CV** (THF, Bu_4_NPF_6,_ *E* vs. Fc/Fc^+^): *E*_red1_= -1.70 V, *E*_red2_ = -2.11 V, *E*_red3_ = -2.67 V. **HR-MS** (ESI): *m/z* calcd for C_34_H_22_N_2_+H^+^: 459.1856 [*M*+H]^+^; found: 459.1855, 939.3470 [2*M*+Na]^+^, 1397.5268 [3*M*+Na]^+^. **Elemental analysis** calcd for C_34_H_22_N_2_ ⋅ ¾ H_2_O: C 86.51, H 5.02, N 5.93, found: C 86.48, H 5.00, N 6.16.

*Aza*-fluorantheno-*aza*-tetrahelicene (**3**): ***R*_f_** (SiO_2_, PE/EA 1:1) = 0.41. **m.p.** 226–228 °C. **^1^H NMR** (600 MHz, CDCl_3_, 295 K, ppm): *δ* = 9.16 (s, 1H, *H*-5), 9.08 (dd, *J* = 8.3, 1.6 Hz, 1H, *H*-1), 9.04 (dd, *J* = 4.4, 1.6 Hz, 1H, *H*-3), 8.67 (d, *J* = 8.3 Hz, 1H, *H*-16), 8.53 (dd, *J* = 4.6, 1.8 Hz, 1H, *H*-12), 8.39 (d, *J* = 6.9 Hz, 1H, H-14), 7.92 (dd, *J* = 8.3, 6.9 Hz, 1H, *H*-15), 7.77 (d, *J* = 8.6 Hz, 1H, *H*-8), 7.66 (dd, *J* = 8.3, 4.3 Hz, 1H, *H*-2), 7.47–7.39 (m, 4H, *H*-2’ & *H*-3’), 7.08 (dd, *J* = 8.6, 2.1 Hz, 1H, *H*-7), 7.03 (dd, J = 7.8, 1.8 Hz, 1H, *H*-10) 7.00 (dd, J = 7.8, 4.6 Hz, 1H, *H*-11), 2.58 (s, 3H, C*H*_3_’’), 2.55 (s, 3H, C*H*_3_’). **^13^C NMR** (151 MHz, CDCl_3_, 295 K, ppm): *δ* = 159.8 (*C*-13a), 148.6 (*C*-3), 148.0 (*C*-12), 147.6 (*C*-8a), 138.9 (*C*-1), 138.6 (*C*-9), 138.3 (*C*-1’), 137.5 (*C*-6), 136.9 (*C*-4b), 135.5 (*C*-16a/*C*-13b), 133.9 (*C*-9b), 132.0 (*C*-9a’), 132.0 (*C-*16b), 131.9 (*C*-9a/*C*-8b), 131.3 (*C*-10), 130.5 (*C*-4’), 130.4 (*C*-2’/*C*-3’), 129.9 (*C*-2’/*C*-3’), 129.1 (*C*-4a), 129.0 (*C*-7), 128.8 (*C*-16), 128.6 (*C*-15), 128.3 (*C*-8), 126.2 (*C*-16a/*C*-13b), 124.8 (*C*-5), 124.6 (*C*-16c), 121.6 (*C*-2), 121.5 (*C*-11), 121.0 (*C*-14), 21.7 (*C*-1’’), 21.5 (*C-*1’). **FT-IR** (KBr disc, cm^-1^): $\tilde{\text{ν}}$ = 3036 (w), 2920 (w), 2852 (2), 1584 (m), 1563 (m), 1440 (s), 810 (s), 767 (vs). **UV/Vis** (CHCl_3_, nm (M^‑1^cm^‑1^$\cdot$10^3^), 295 K): *λ*_max_ (*ε*) = 394 (10.9), 378.6 (10.8), 324 (47.4), 313 (42.8), 272.4 (41.3). **Fluorescence** (CHCl_3_, nm, 295 K): *λ*_exc_ *=* 345, *λ*_em_ = 465. **CV** (THF, Bu_4_NPF_6,_ *E* vs. Fc/Fc^+^): *E*_red1_= -2.04 V, *E*_red2_= -2.50 V. **HR-MS** (ESI): *m/z* calcd for C_34_H_22_N_2_+H^+^: 459.1842; found: 459.1856. **Elemental analysis** calcd. for C_34_H_22_N_2_⋅ H_2_O: C 86.51, H 5.02, N 5.93, found: C 86.42, H 5.43, N 5.90.

Di-*aza*-tri-benzo-pentahelicene (**4**): ***R*_f_** (SiO_2_, PE/EA 1:1) = 0.74. **m.p.** 199–201 °C. **^1^H NMR** (700 MHz, CDCl_3_, 295 K, ppm): *δ* = 9.15 (dd, *J* = 8.2, 1.7 Hz, 2H, *H*-1), 9.07–9.06 (m, 4H, *H*-5 & *H*-3), 8.71–8.70 (m, 2H, *H*-9), 7.94 (d, *J* = 8.4 Hz, 2H, *H*-8), 7.65–7.62 (m, 4H, *H*-2 & *H*-10), 7.17 (dd, *J* = 8.5, 2.0 Hz, 2H, *H*-7), 2.61 (s, 6H, C*H*_3_). **^13^C NMR** (176 MHz, CDCl_3_, 295 K, ppm): *δ* = 148.5 (*C*-3/*C*-5), 147.8 (*C*-4a), 137.5 (*C*-6), 136.4 (*C*-1), 130.9 (*C*-8b), 130.4 (*C*-8d), 129.9 (*C*-8), 129.7, 128.7 (*C*-7), 128.4 (*C*-9), 127.7 (*C*-8c), 126.6 (*C*-2/*C*-10), 126.4 (*C*-8a), 124.3 (*C*-3/*C*-5), 123.7 (C-4b), 120.9 (*C*-2/*C*-10), 21.8 (*C*H_3_). **FT-IR** (KBr disc, cm^-1^): $\tilde{\text{ν}}$ = 3026 (w), 2918 (m), 2852 (w), 1568 (m), 1536 (m), 1479 (m), 1392 (m), 816 (vs), 793 (s), 762 (s), 742 (s). **UV/Vis** (CHCl_3_, nm (M^‑1^cm^‑1^$\cdot$10^3^), 295 K): *λ*_max_ (*ε*) = 377 (sh, 15.2), 328 (52.4), 272.6 (47.9). **Fluorescence** (CHCl_3_, nm, 295 K): *λ*_exc_ = 346, *λ*_em_ = 446. **DPV** (THF, Bu_4_NPF_6,_ *E* vs. Fc/Fc^+^): *E*_red1_= -2.35 V, *E*_red2_= -2.84 V, *E*_red3_= -3.39 V. **HR-MS** (ESI): *m/z* calcd for C_34_H_22_N_2_+H^+^: 459.1842 [*M*+H]^+^; found: 459.1850, 939.3462 [2*M*+Na]^+^. **Elemental analysis** calcd for C_34_H_22_N_22_: C 89.05, H 4.84, N 6.11, found: C 89.05, H 4.94, N 6.21.

### Optimized Reaction Conditions for the Pd-Catalyzed Benzannelation of 1

Chloropyridine **1** (25.0 mg, 47.0 µmol), bis-(tricyclohexylphosphino)-palladium(II)-dichlorid (7.3 mg, 9.9 µmol, 21 mol%) and potassium bicarbonate (65 mg, 0.46 mmol) were dissolved in dry mesitylene (2.0 mL) under argon atmosphere and the reaction mixture was stirred at 160 °C for 20 hours. The reaction mixture was allowed to cool to room temperature and water (20 mL) and DCM (40 mL) were added. The phases were separated and the organic phase was washed with water (2 x 20 mL) and brine (50 mL). The organic phase was dried over anhydrous Na_2_SO_4_ and the solvents were removed under reduced pressure. The crude product was purified by column chromatography (SiO_2_, PE/EA 1:1, *R*_f_ = 0.74) to give bis-pyridino-benzo-pentahelicene (**4**) as a yellow solid (15.9 mg, 74%).

***R*_f_** (SiO_2_, PE/EA 1:1) = 0.74. **m.p.** 199–201 °C. **^1^H NMR** (700 MHz, CDCl_3_, 295 K, ppm): *δ* = 9.15 (dd, *J* = 8.2, 1.7 Hz, 2H, *H*-1), 9.07–9.06 (m, 4H, *H*-5 & *H*-3), 8.71–8.70 (m, 2H, *H*-9), 7.94 (d, *J* = 8.4 Hz, 2H, *H*-8), 7.65–7.62 (m, 4H, *H*-2 & *H*-10), 7.17 (dd, *J* = 8.5, 2.0 Hz, 2H, *H*-7), 2.61 (s, 6H, C*H*_3_). **^13^C NMR** (176 MHz, CDCl_3_, 295 K, ppm): *δ* = 148.5 (*C*-3/*C*-5), 147.8 (*C*-4a), 137.5 (*C*-6), 136.4 (*C*-1), 130.9 (*C*-8b), 130.4 (*C*-8d), 129.9 (*C*-8), 129.7, 128.7 (*C*-7), 128.4 (*C*-9), 127.7 (*C*-8c), 126.6 (*C*-2/*C*-10), 126.4 (*C*-8a), 124.3 (*C*-3/*C*-5), 123.7 (C-4b), 120.9 (*C*-2/*C*-10), 21.8 (*C*H_3_). **FT-IR** (KBr disc, cm^-1^): $\tilde{\text{ν}}$ = 3026 (w), 2918 (m), 2852 (w), 1568 (m), 1536 (m), 1479 (m), 1392 (m), 816 (vs), 793 (s), 762 (s), 742 (s). **UV/Vis** (CHCl_3_, nm (M^‑1^cm^‑1^$\cdot$10^3^), 295 K): *λ*_max_ (*ε*) = 377 (sh, 15.2), 328 (52.4), 272.6 (47.9). **Fluorescence** (CHCl_3_, nm, 295 K): *λ*_exc_ = 346, *λ*_em_ = 446. **DPV** (THF, Bu_4_NPF_6,_ *E* vs. Fc/Fc^+^): *E*_red1_= -2.35 V, *E*_red2_= -2.84 V, *E*_red3_= -3.39 V. **HR-MS** (ESI): *m/z* calcd for C_34_H_22_N_2_+H^+^: 459.1842 [*M*+H]^+^; found: 459.1850, 939.3462 [2*M*+Na]^+^. **Elemental analysis** calcd for C_34_H_22_N_22_: C 89.05, H 4.84, N 6.11, found: C 89.05, H 4.94, N 6.21.

# 2 Spectra

## ^1^H and ^13^C NMR Spectra


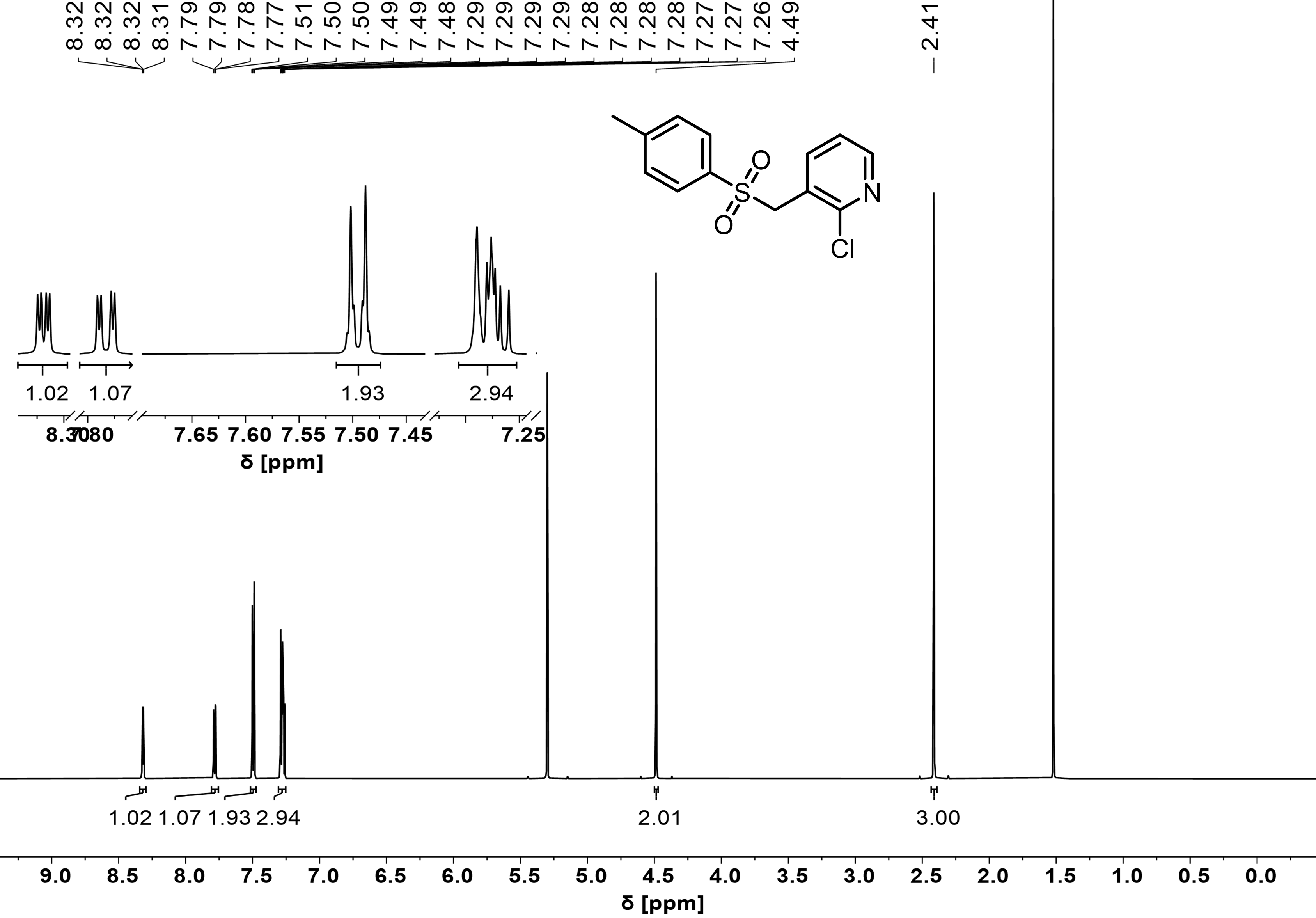


**Figure S1.** ^1^H NMR spectrum (600 MHz, 295 K) of 2-chloro-3-(tosylmethyl)pyridine (**8**) in CD_2_Cl_2_.


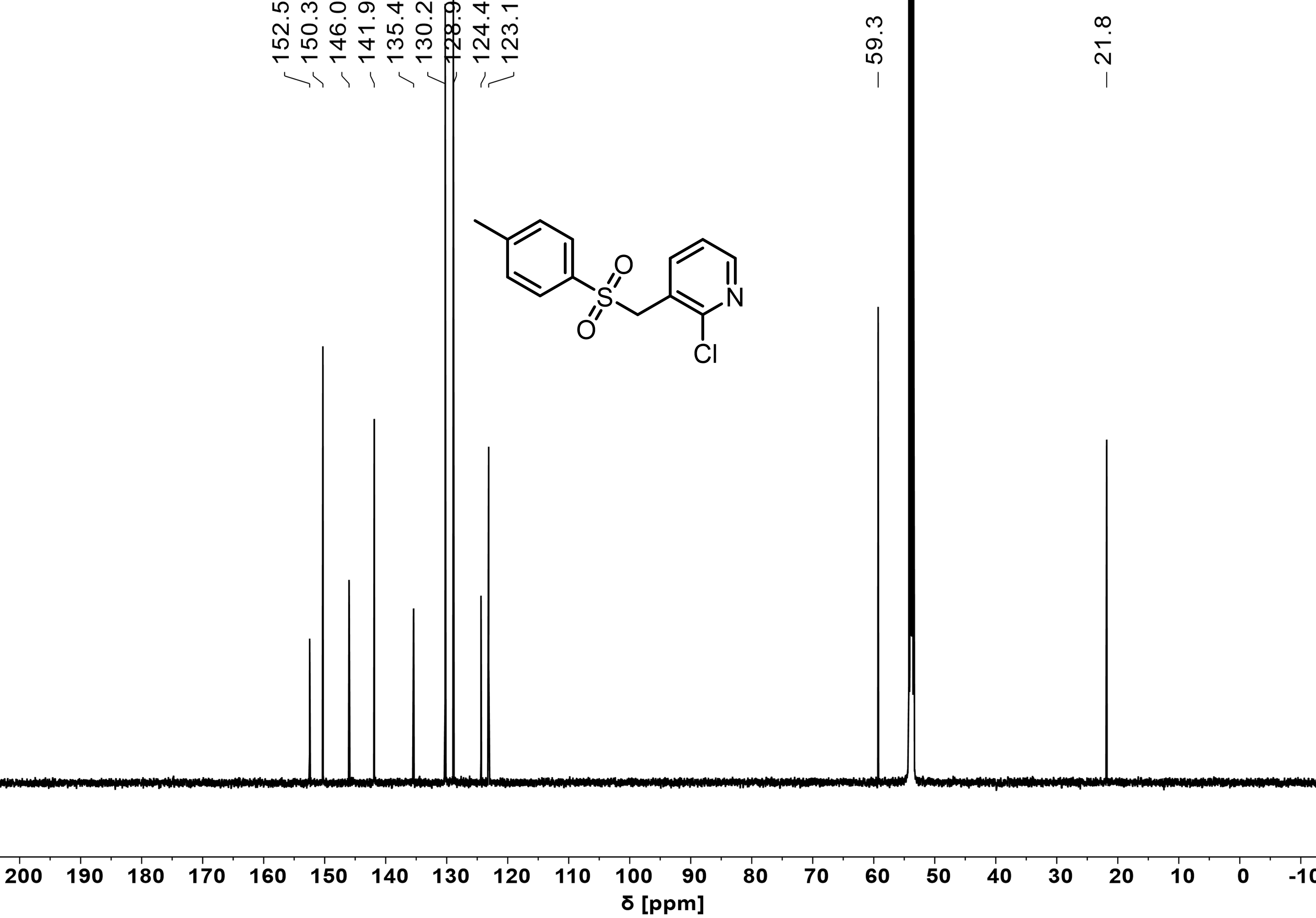


**Figure S2.** ^13^C NMR spectrum (151 MHz, 295 K) of 2-chloro-3-(tosylmethyl)pyridine (**8**) in CD_2_Cl_2_.


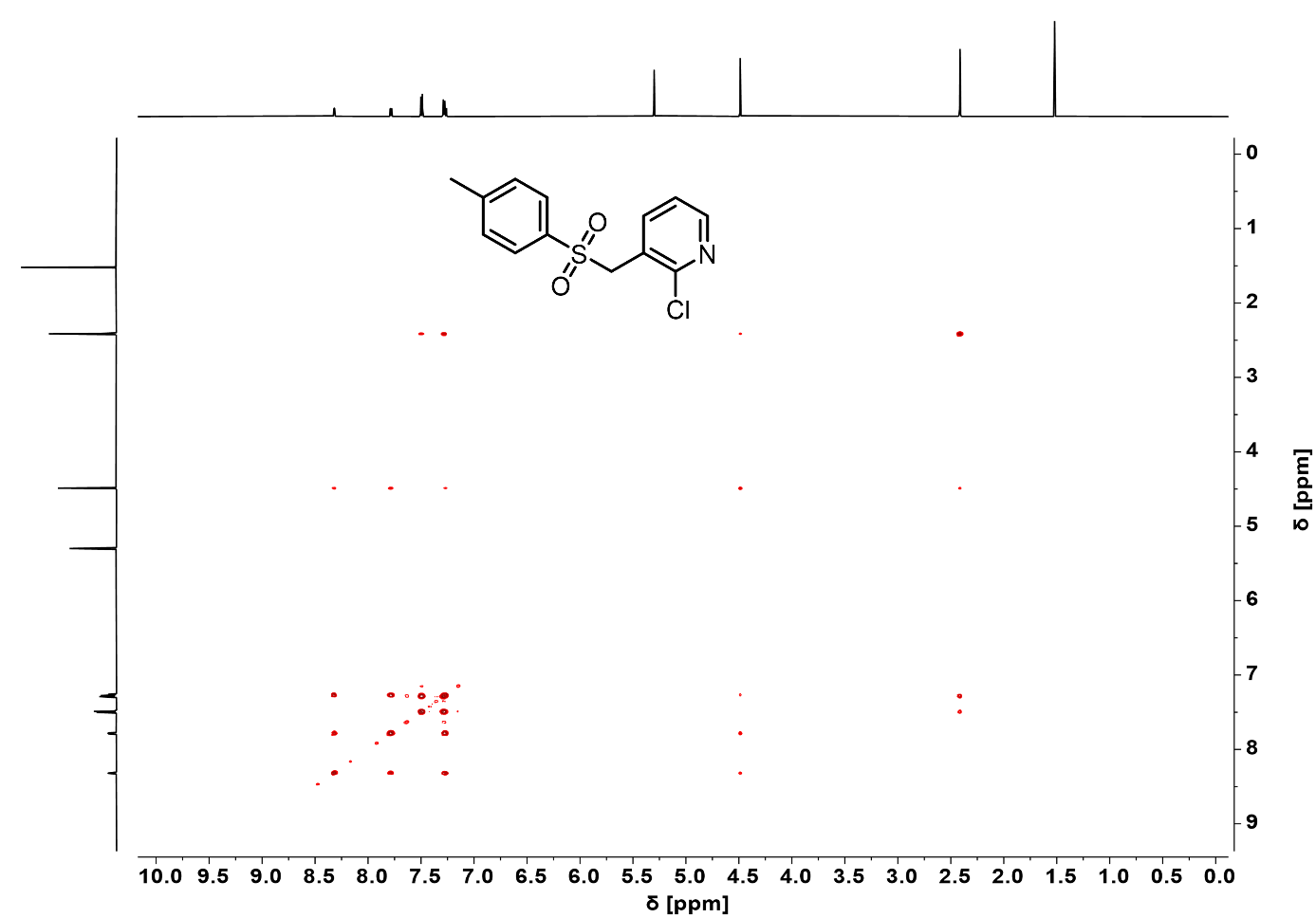


**Figure S3**. ^1^H-^1^H-COSY NMR spectrum (600 MHz, 295 K) of 2-chloro-3-(tosylmethyl)pyridine (**8**) in CD_2_Cl_2_.


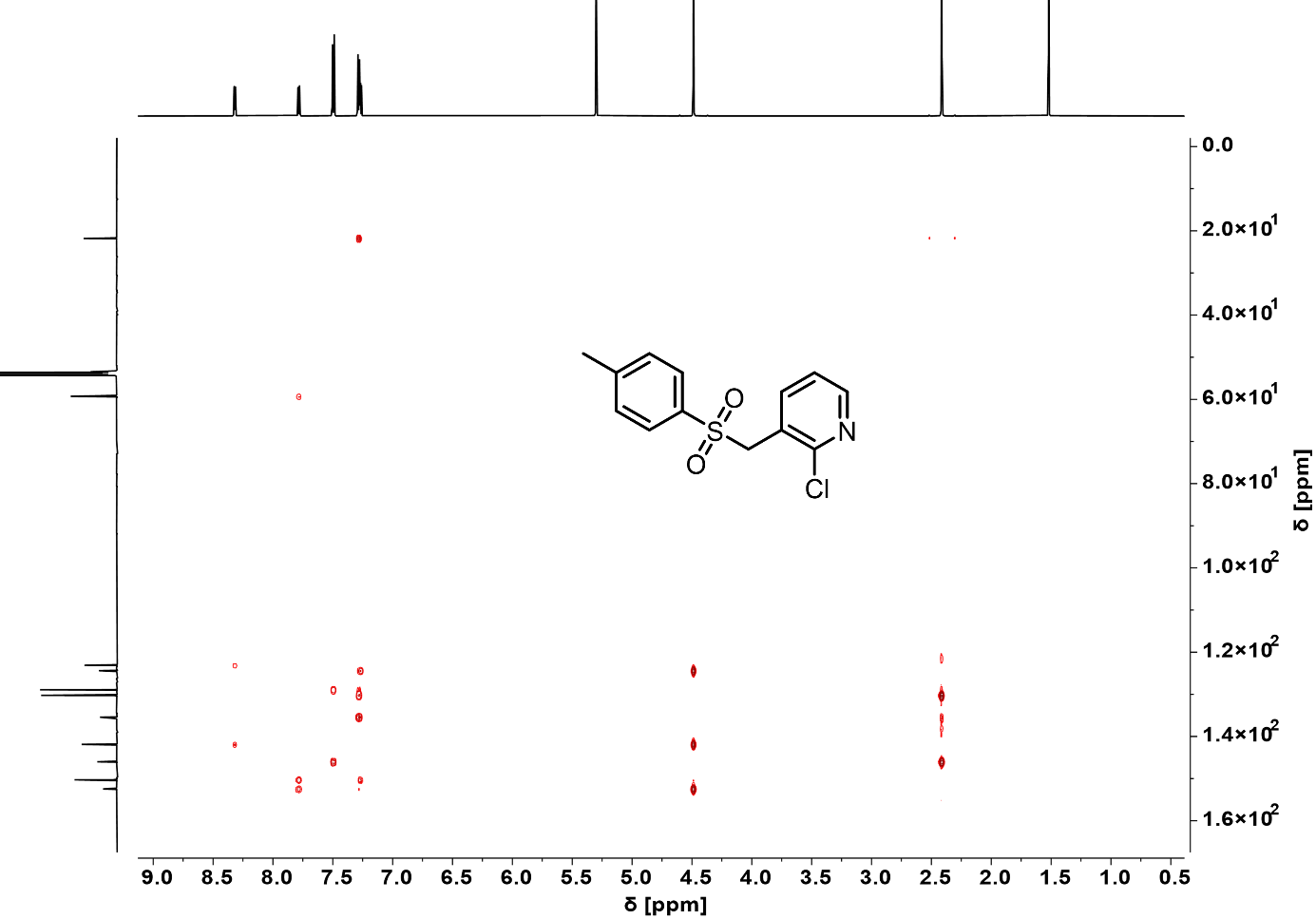


**Figure S4.** ^1^H-^13^C-HMBC NMR spectrum (600 MHz/151 MHz, 295 K) of 2-chloro-3-(tosylmethyl)pyridine (**8**) in CD_2_Cl_2_.


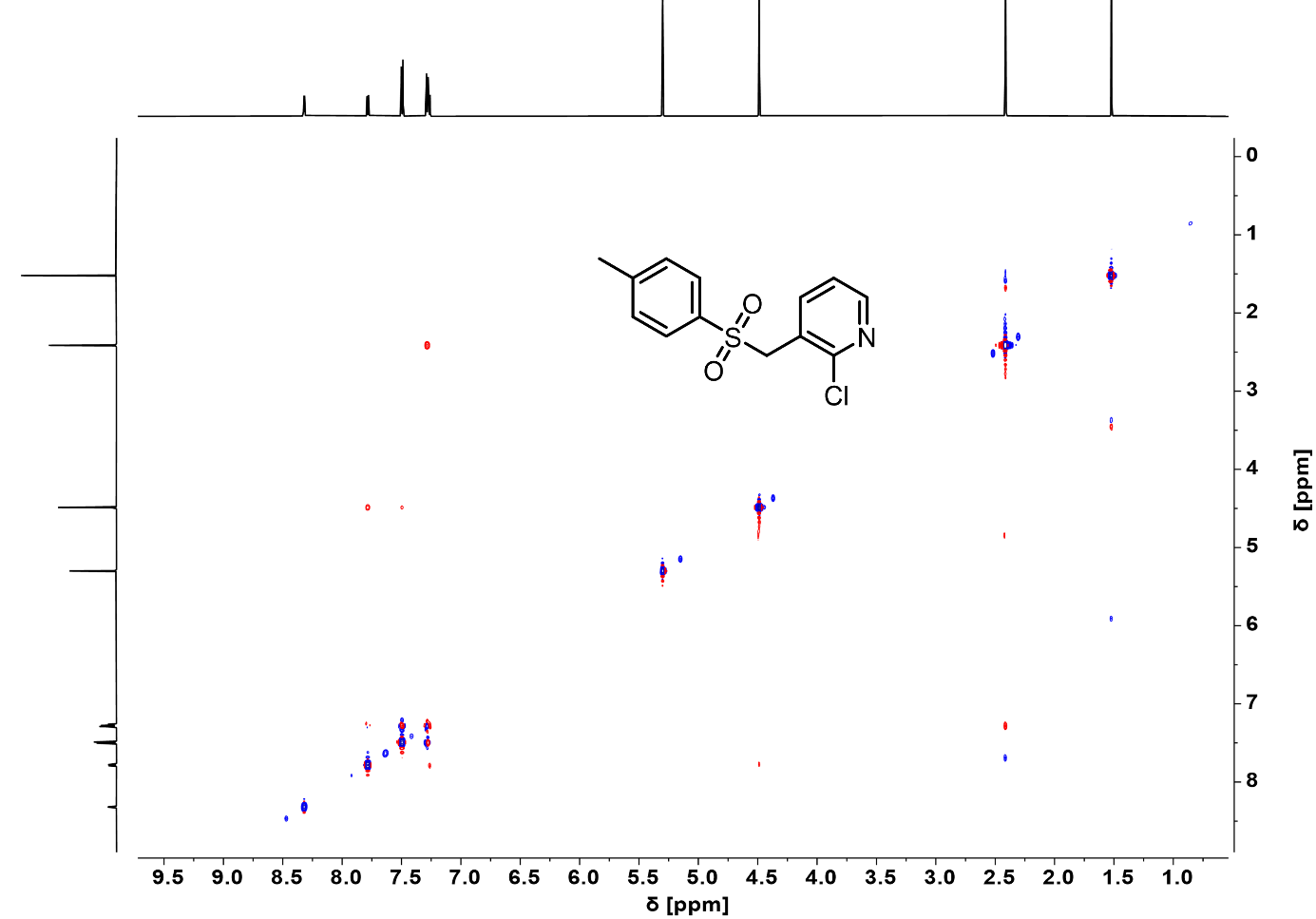


**Figure S5**. ^1^H-^1^H-ROESY NMR spectrum (600 MHz, 295 K) of 2-chloro-3-(tosylmethyl)pyridine (**8**) in CD_2_Cl_2_.


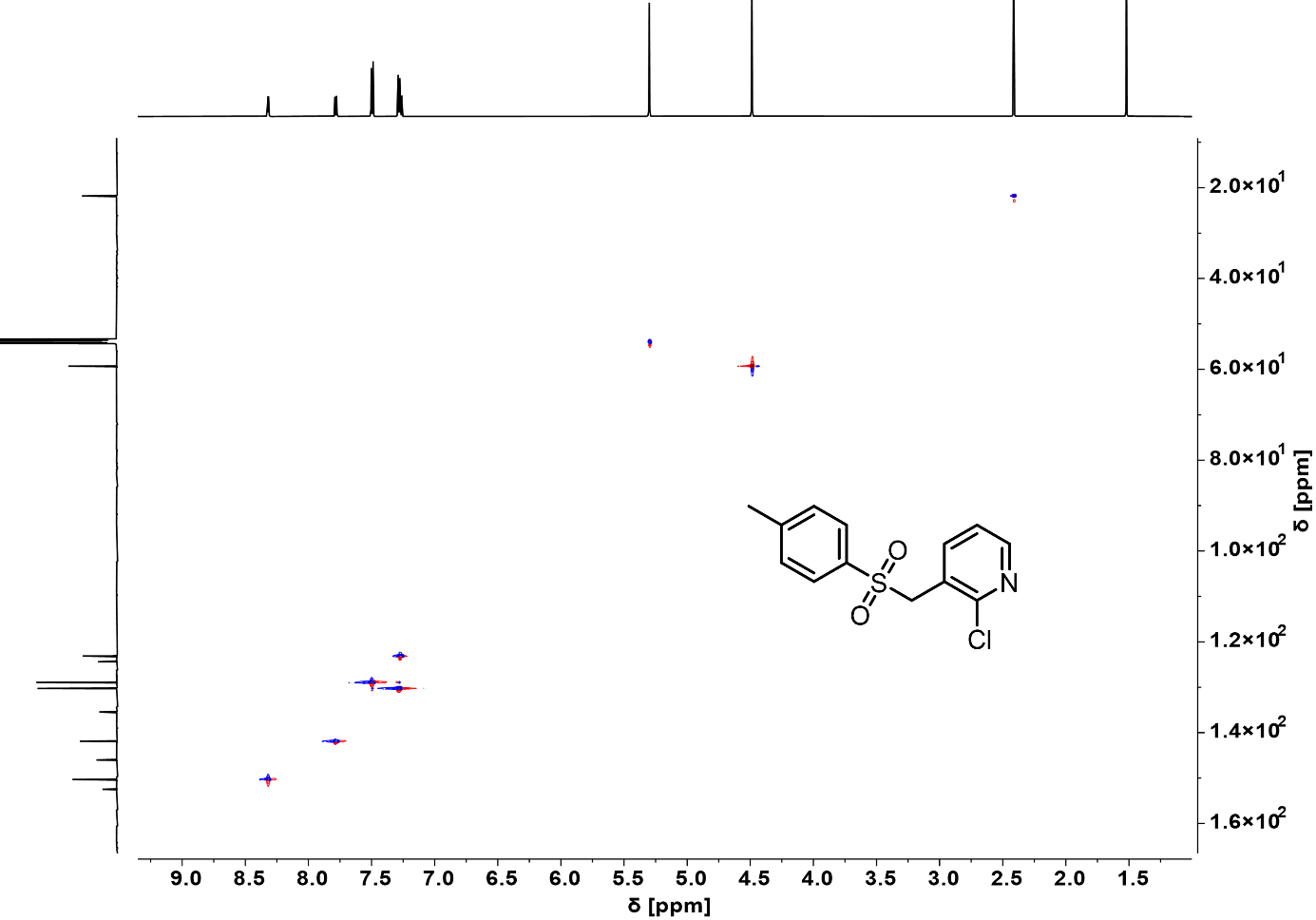


**Figure S6**. ^1^H-^13^C HSQC NMR spectrum (600 MHz, 295 K) of 2-chloro-3-(tosylmethyl)pyridine (**8**) in CD_2_Cl_2_.


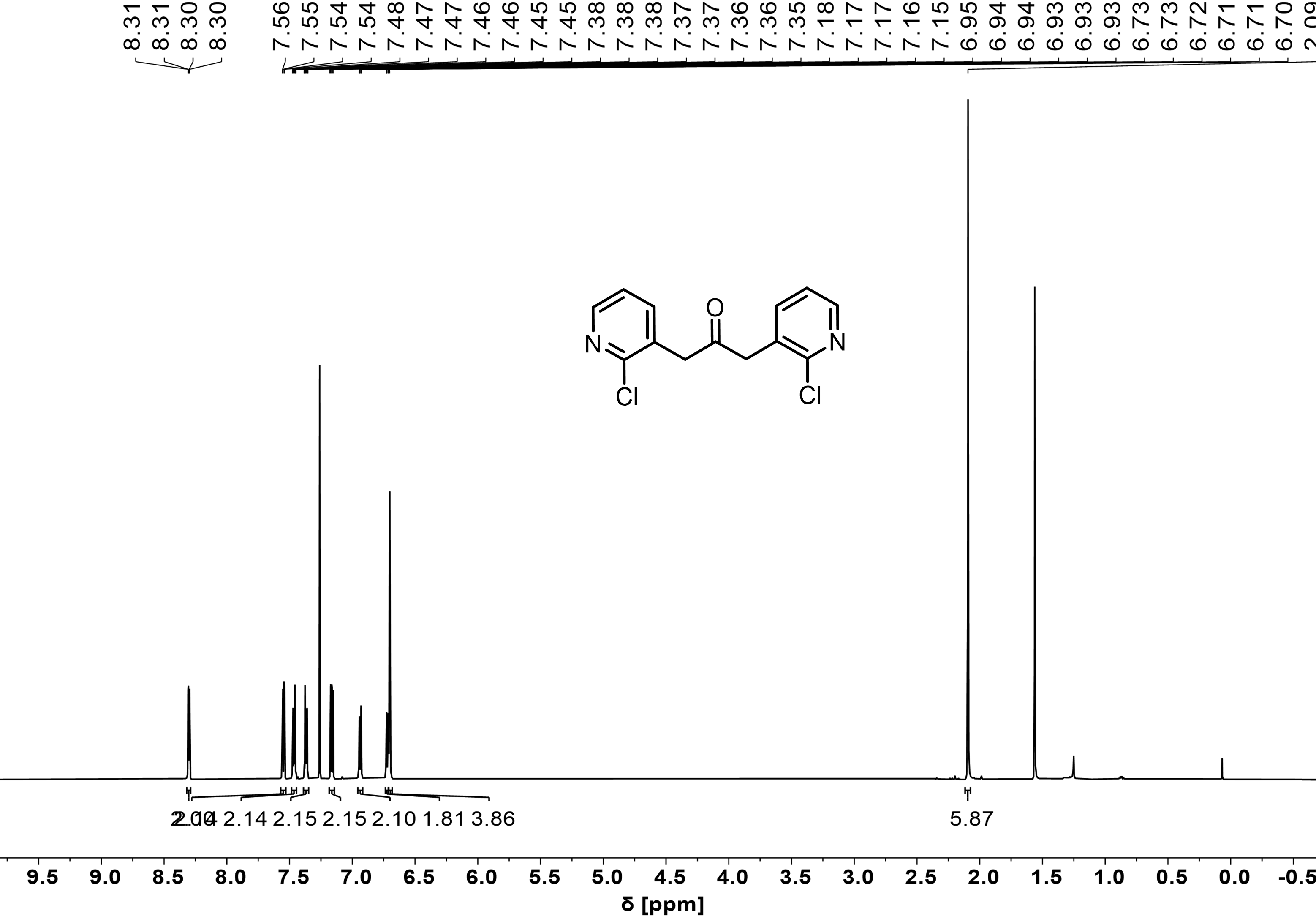


**Figure S7**. ^1^H NMR spectrum (600 MHz, 295 K) of 1,3-bis(2-chloropyridin-3-yl)propan-2-one (**7**) in CDCl_3_.


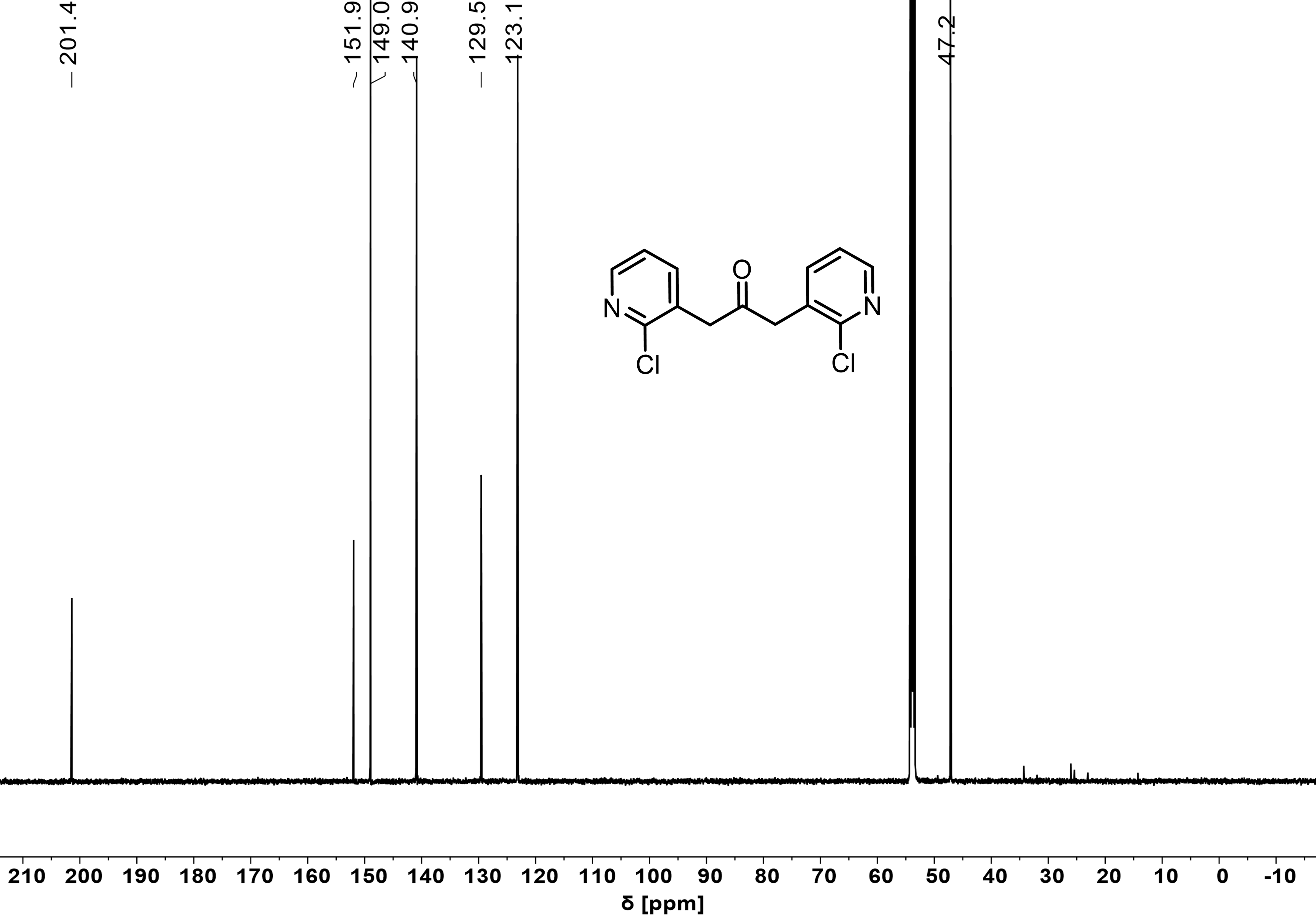


**Figure S8**. ^13^C NMR spectrum (151 MHz, 295 K) of 1,3-bis(2-chloropyridin-3-yl)propan-2-one (**7**) in CDCl_3_.


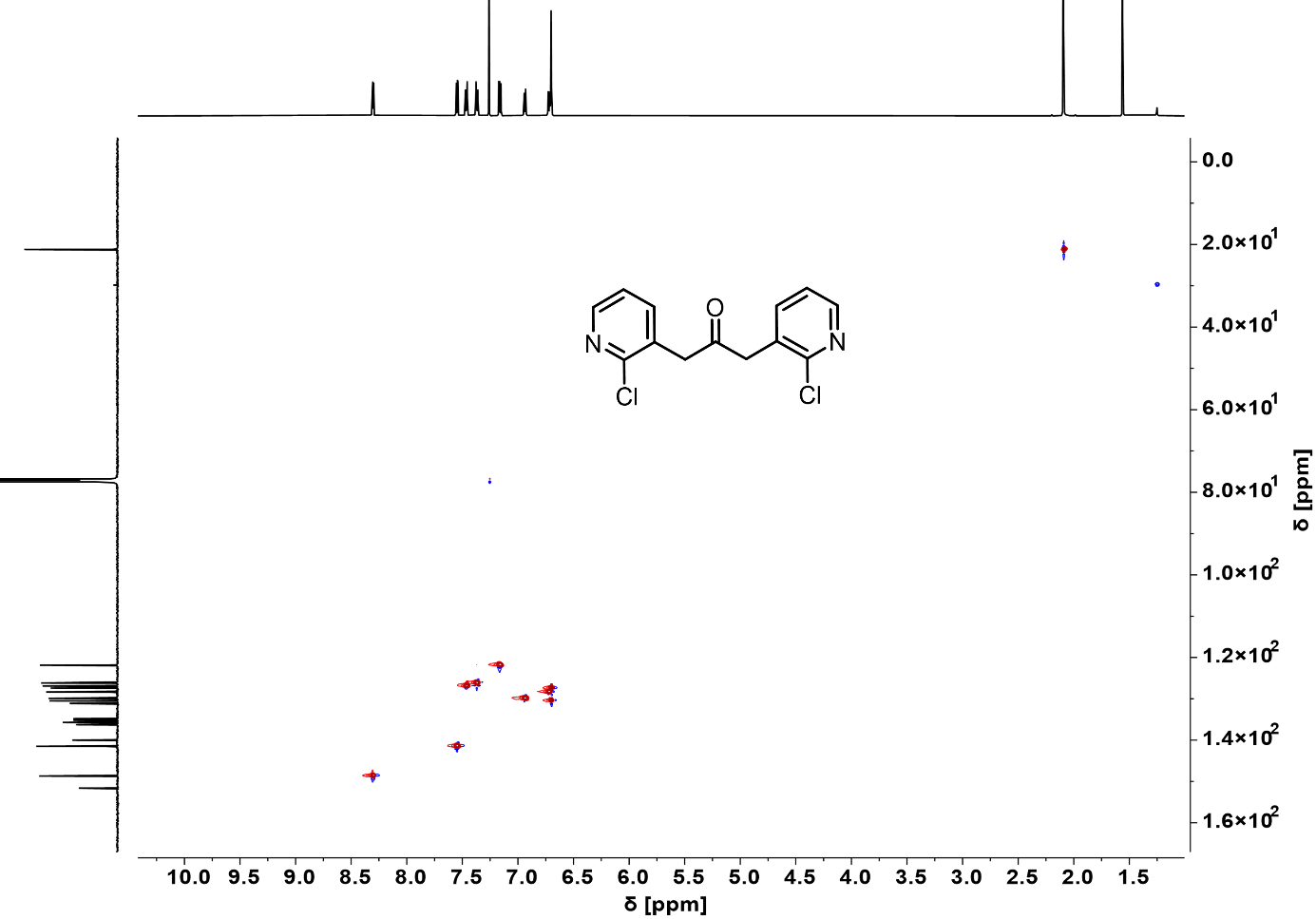


**Figure S9**. ^1^H-^13^C-HSQC NMR spectrum (600 MHz, 151 MHz, 295 K) of 1,3-bis(2-chloropyridin-3-yl)propan-2-one (**7**) in CDCl_3_.


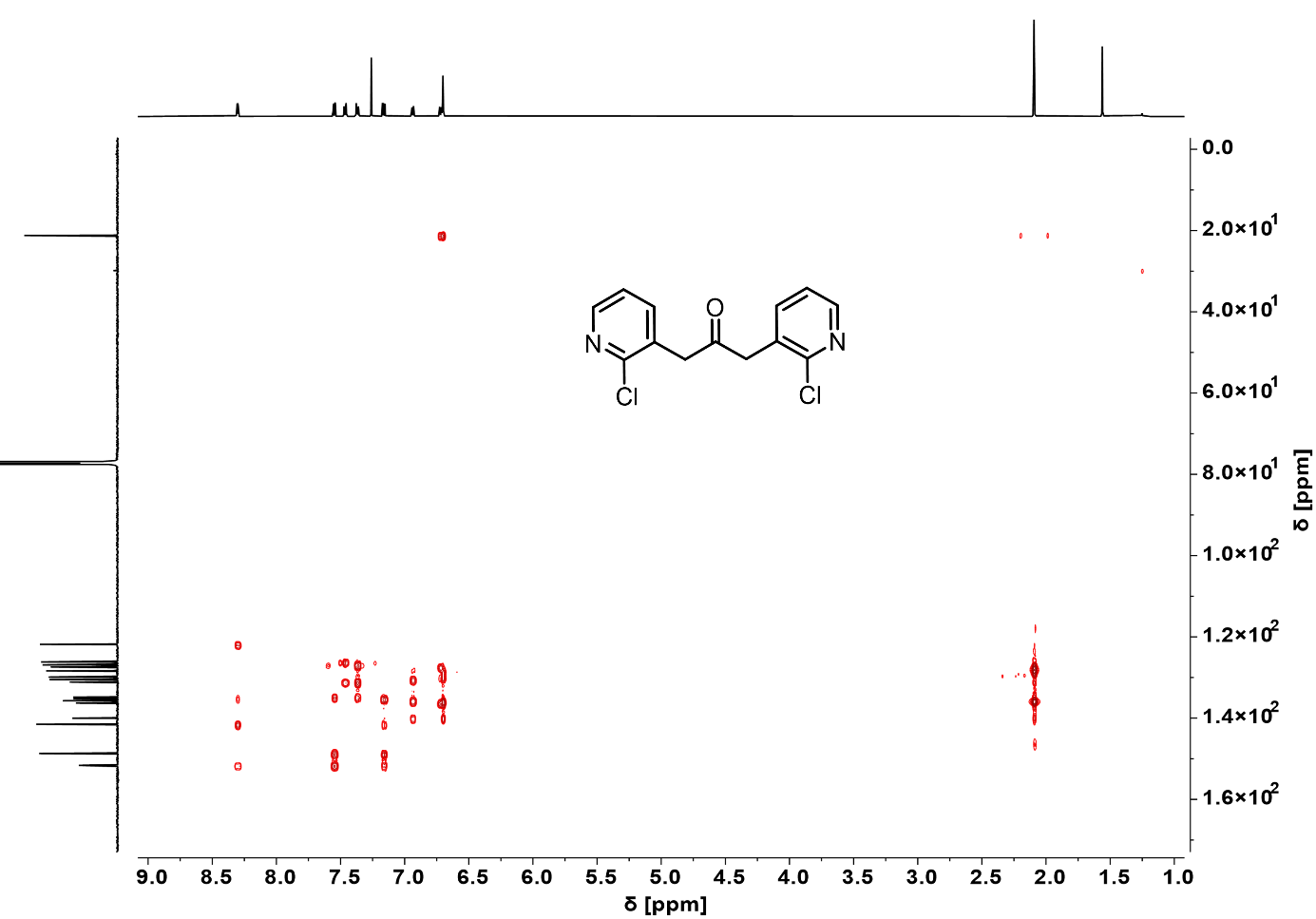


**Figure S10**. ^1^H-^13^C-HMBC NMR spectrum (600 MHz, 151 MHz, 295 K) of 1,3-bis(2-chloropyridin-3-yl)propan-2-one (**7**) in CDCl_3_.


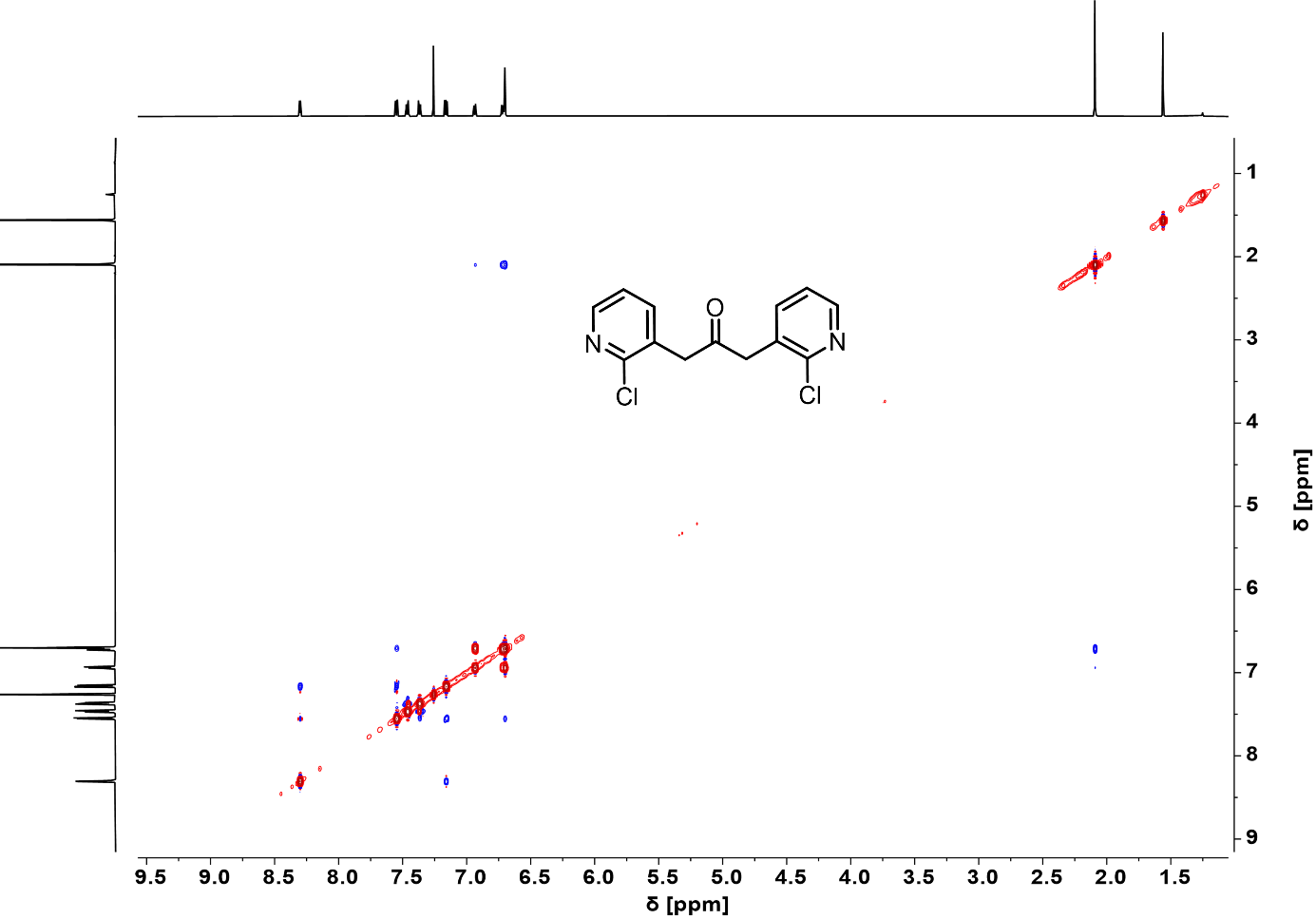


**Figure S11**. ^1^H-^1^H-NOESY NMR spectrum (600 MHz, 295 K) of 1,3-bis(2-chloropyridin-3-yl)propan-2-one (**7**) in CDCl_3_.


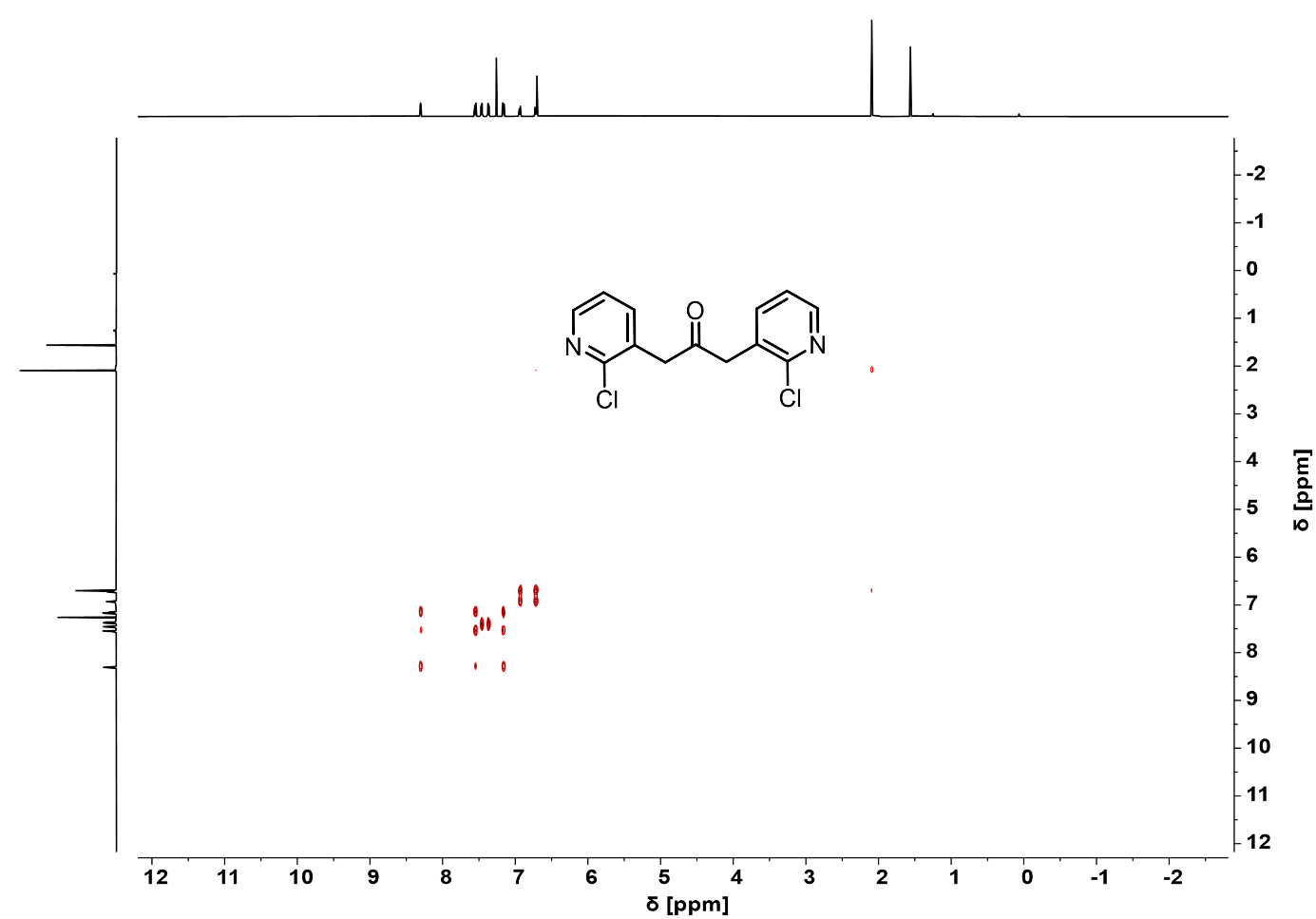


**Figure S12**. ^1^H-^1^H-COSY NMR spectrum (600 MHz, 295 K) of 1,3-bis(2-chloropyridin-3-yl)propan-2-one (**7**) in CDCl_3_.


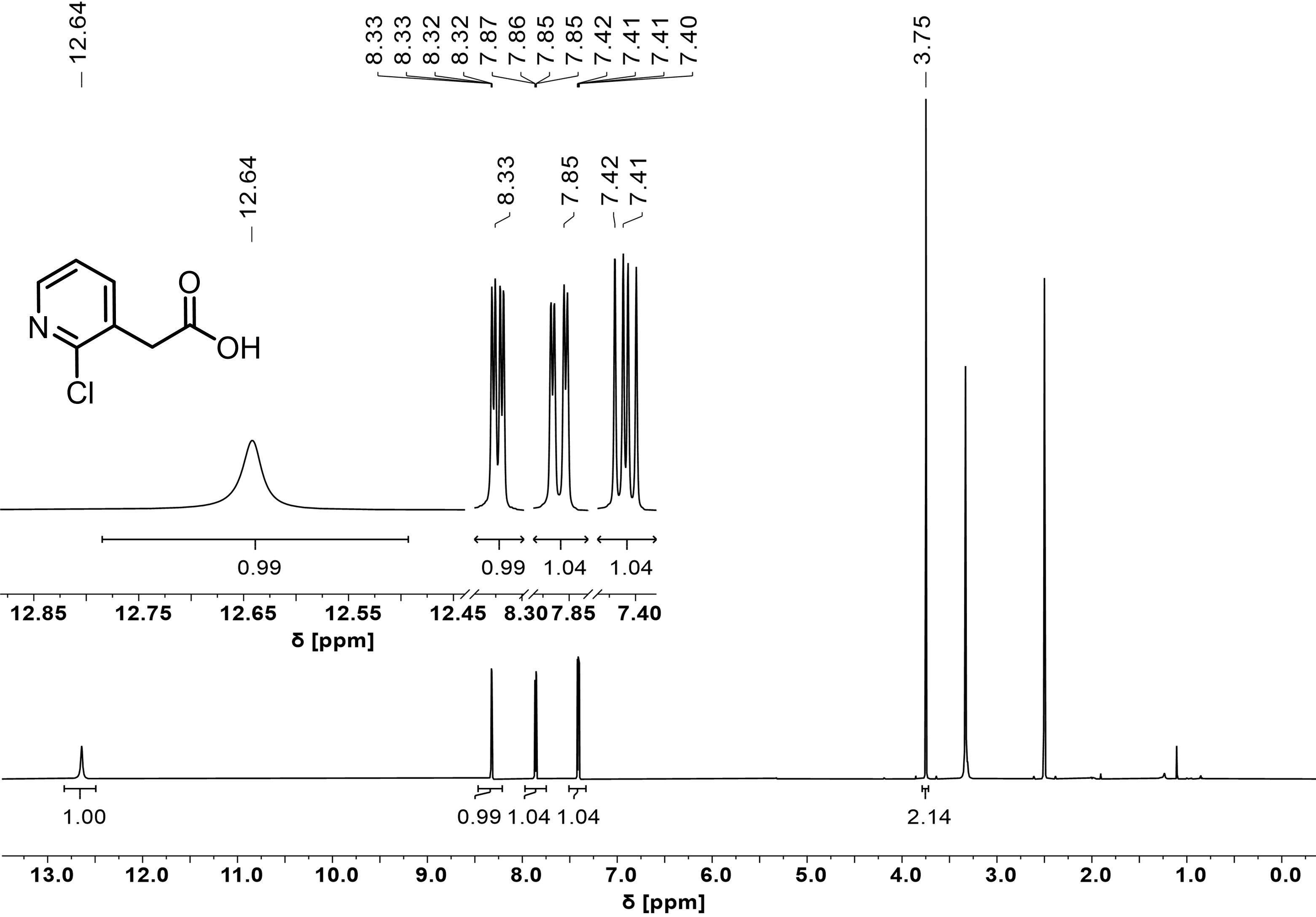


**Figure S13**. ^1^H NMR spectrum (600 MHz, 295 K) of 2-(2-chloropyridin-3-yl)acetic acid (**10**) in DMSO-d_6_.


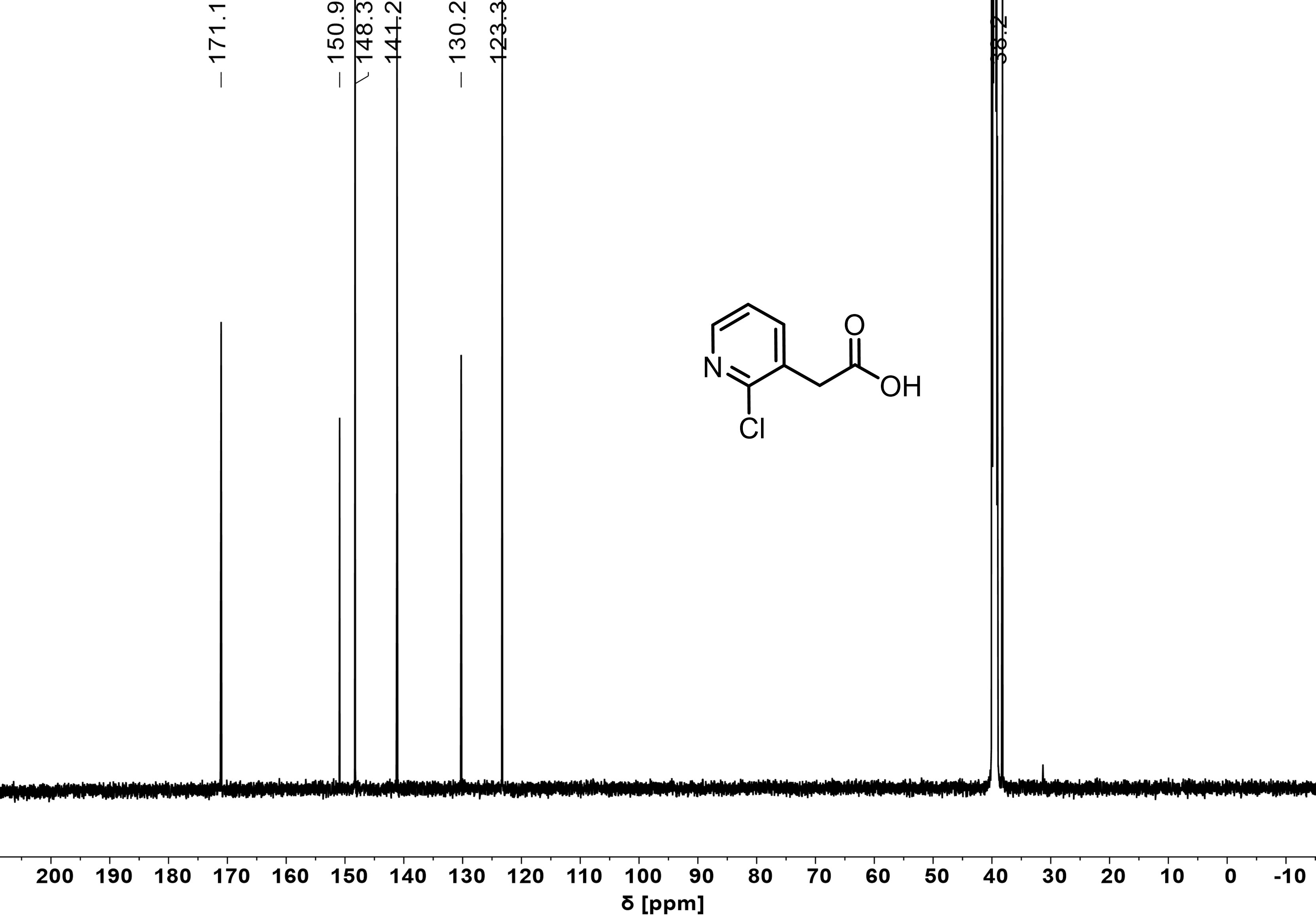


**Figure S14**. ^13^C NMR spectrum (151 MHz, 295 K) of 2-(2-chloropyridin-3-yl)acetic acid (**10**) in DMSO-d_6_.


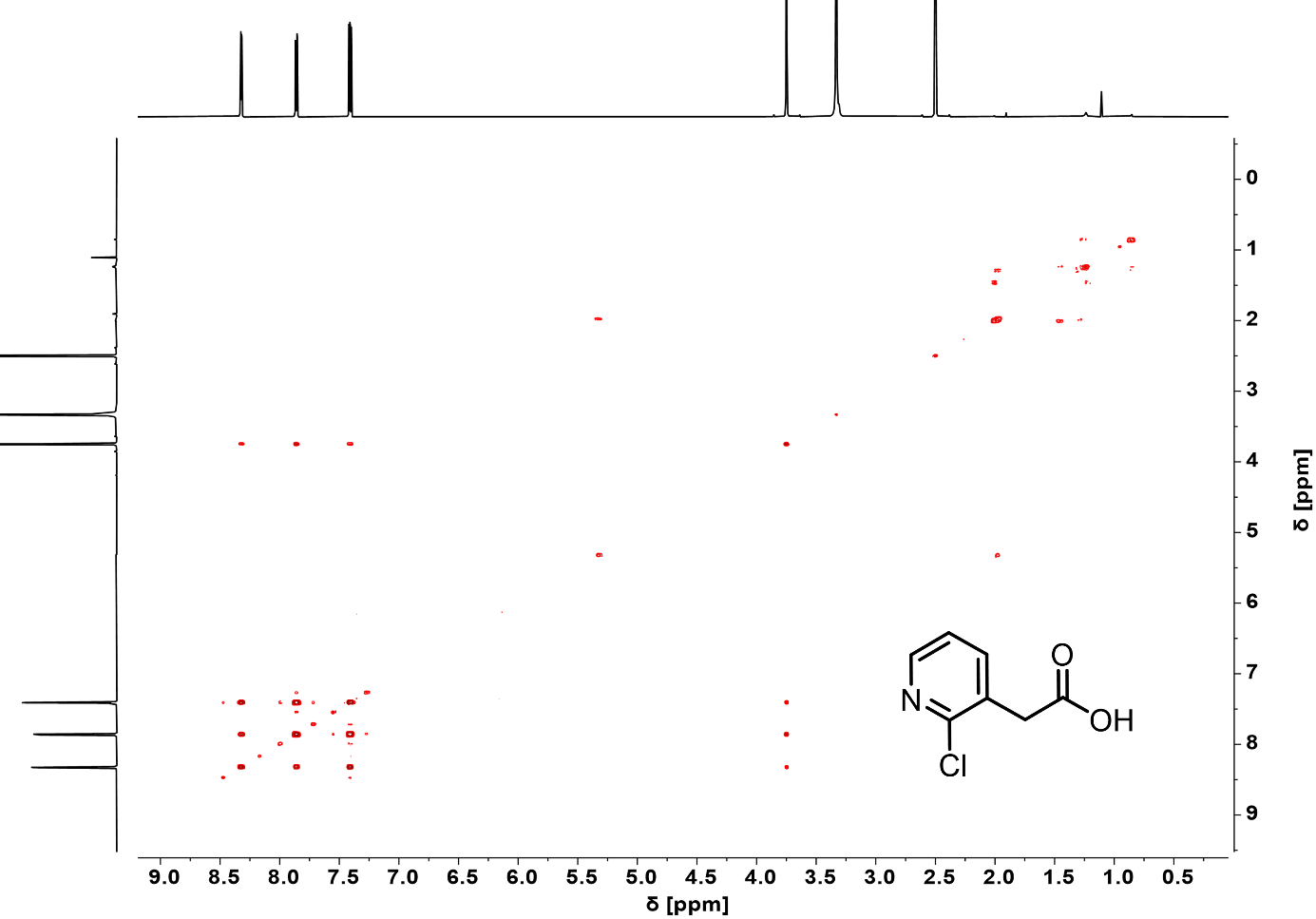


**Figure S15**. ^1^H-^1^H COSY NMR spectrum (600 MHz, 295 K) of 2-(2-chloropyridin-3-yl)acetic acid (**10**) in DMSO-d_6_.


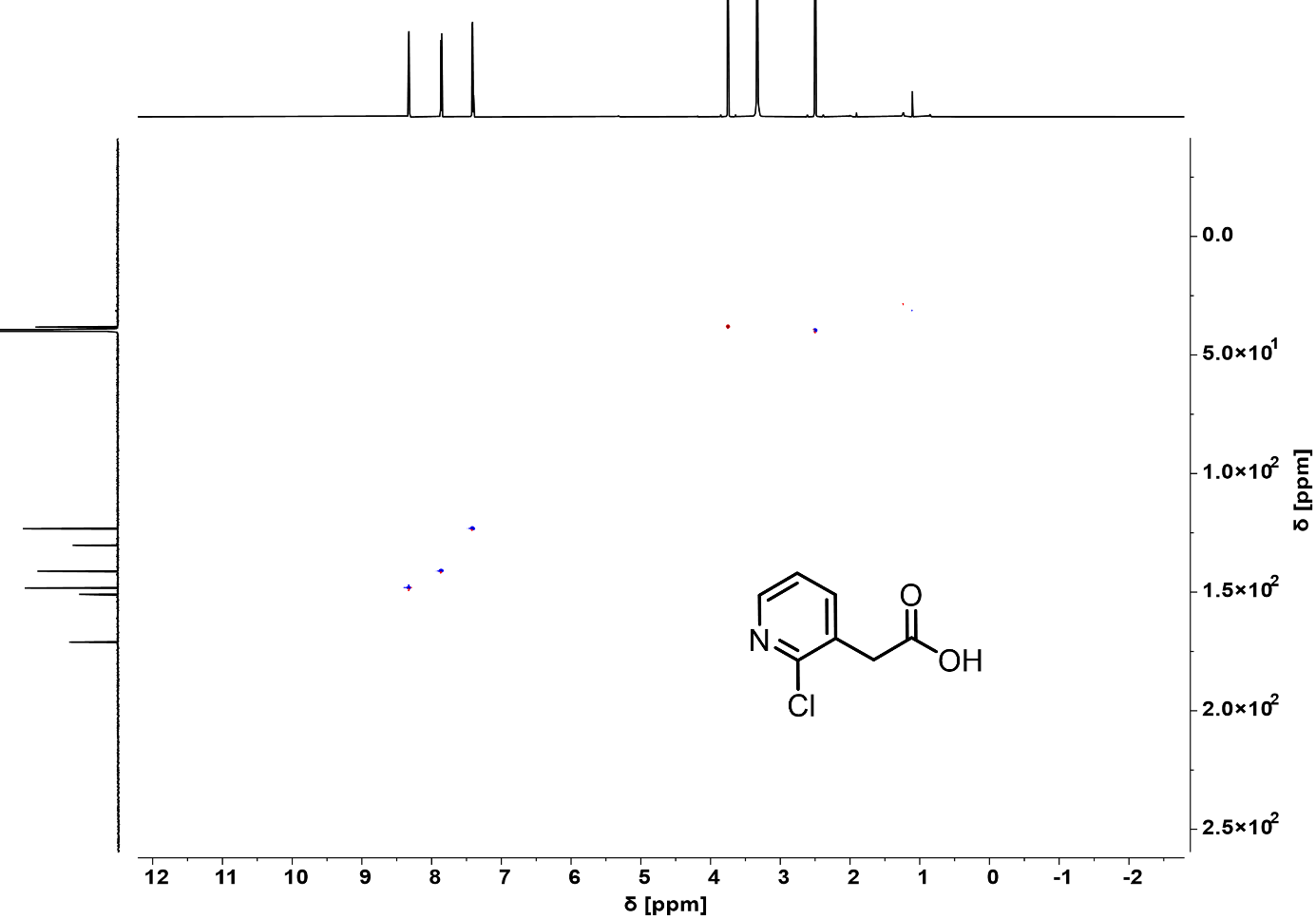


**Figure S16**. ^1^H-^13^C HSQC NMR spectrum (600 MHz, 151 MHz, 295 K) of 2-(2-chloropyridin-3-yl)acetic acid (**10**) in DMSO-d_6_.


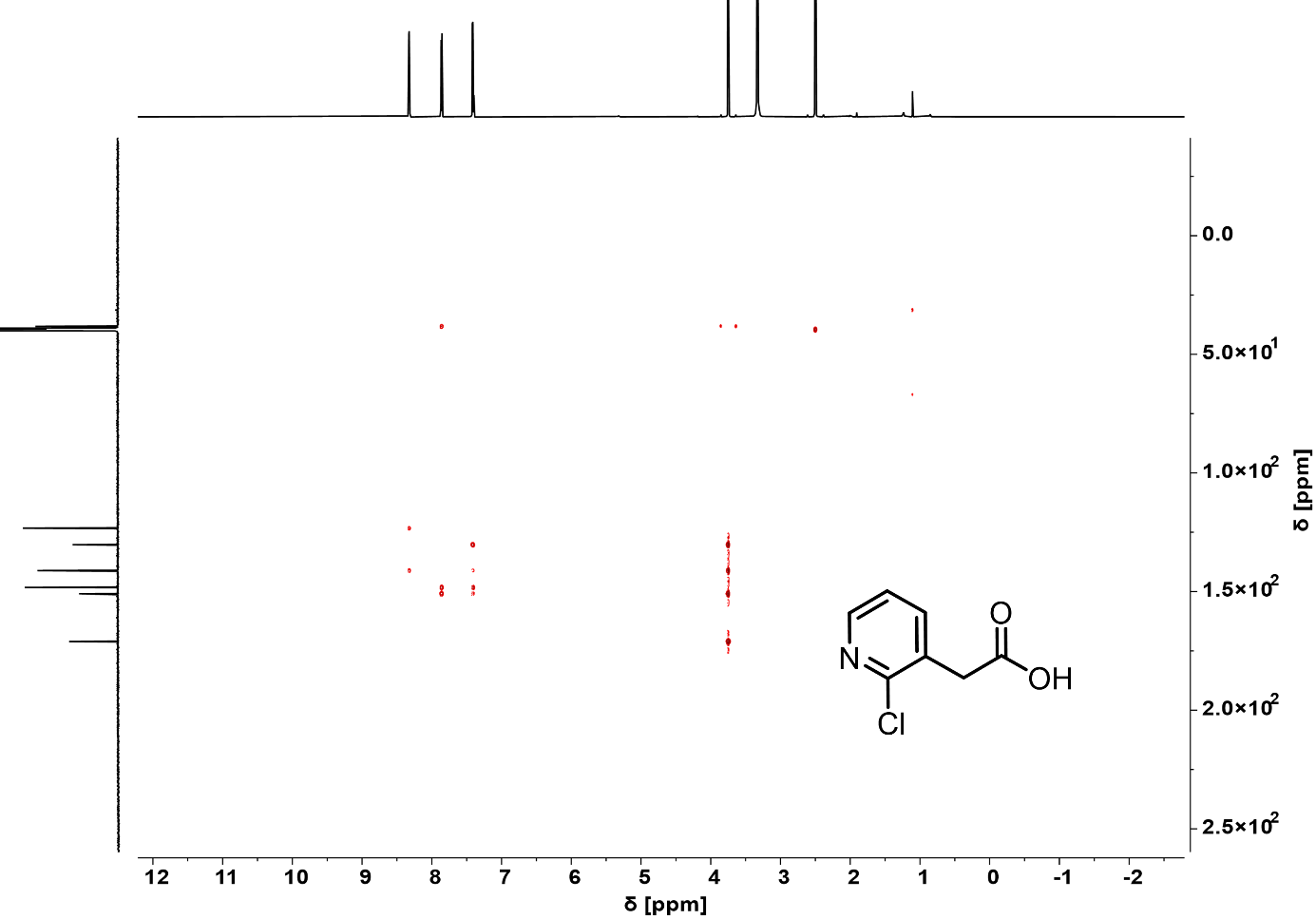


**Figure S17**. ^1^H-^13^C HMBC NMR spectrum (600 MHz, 151 MHz, 295 K) of 2-(2-chloropyridin-3-yl)acetic acid (**10**) in DMSO-d_6_.


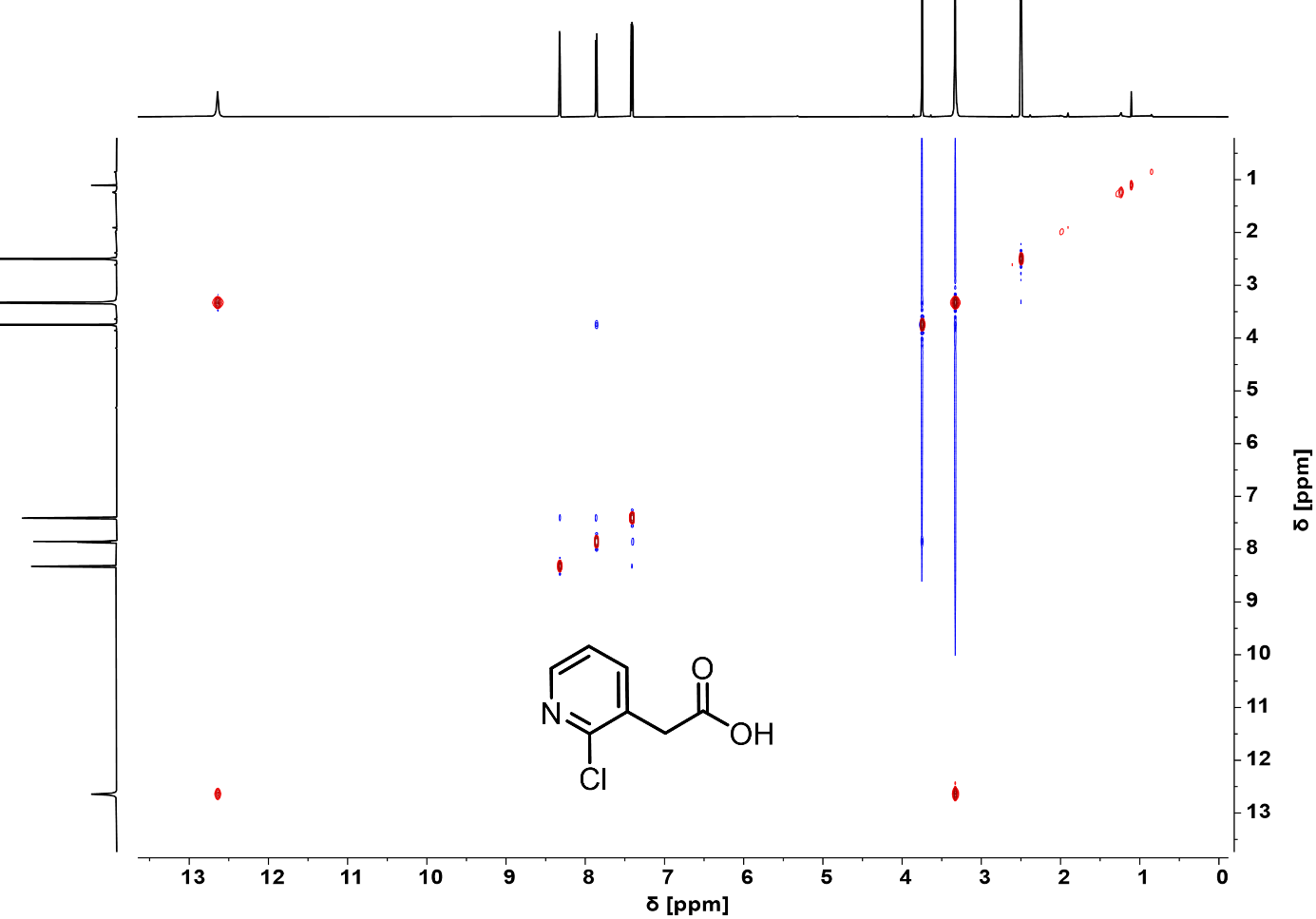


**Figure S18**. ^1^H-^1^H NOESY NMR spectrum (600 MHz, 295 K) of 2-(2-chloropyridin-3-yl)acetic acid (**10**) in DMSO-d_6_.


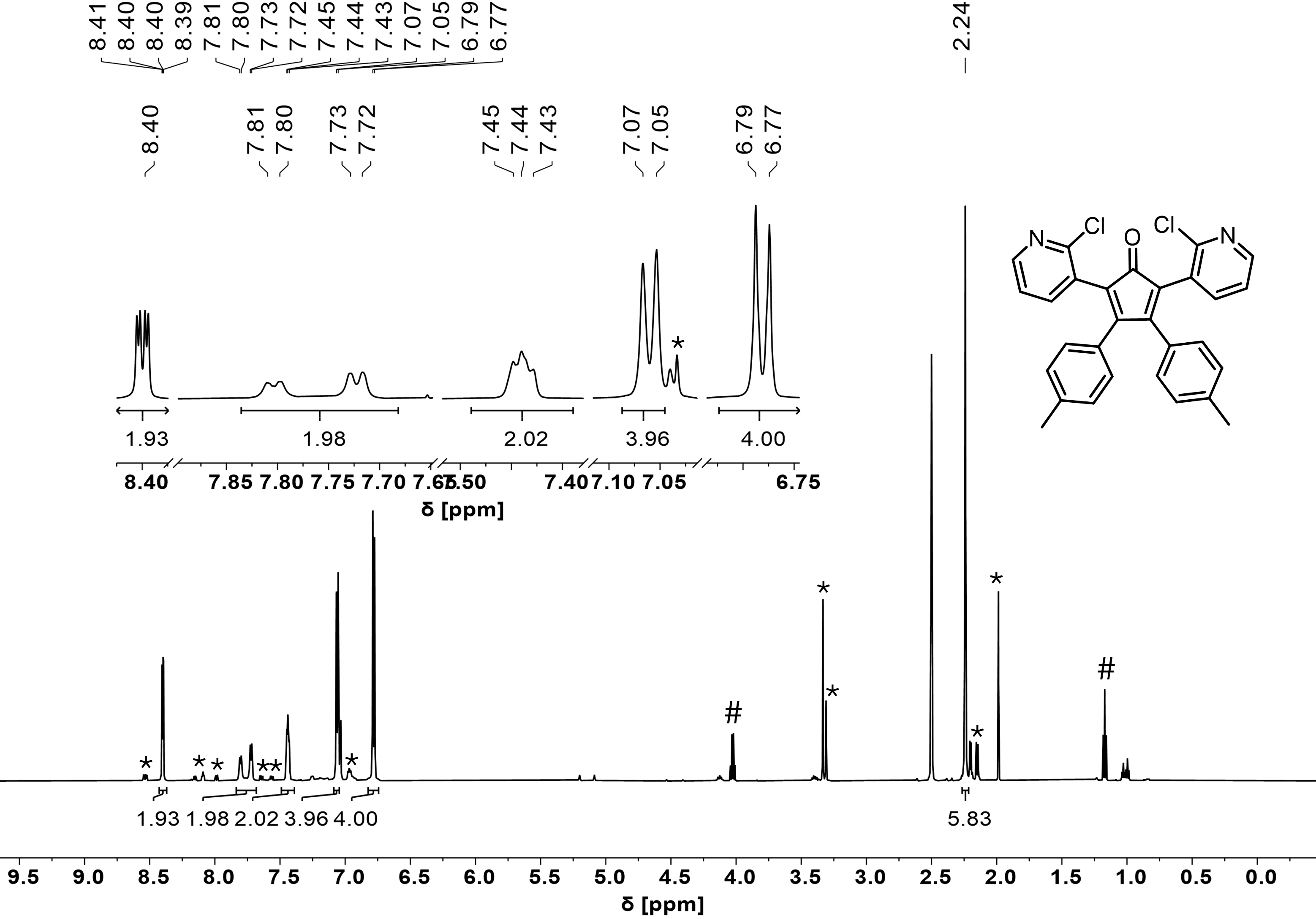


**Figure S19** ^1^H NMR spectrum (600 MHz, 295 K) of cyclopentadienone **12** in DMSO-d_6_.*: impurity. #: ethanol


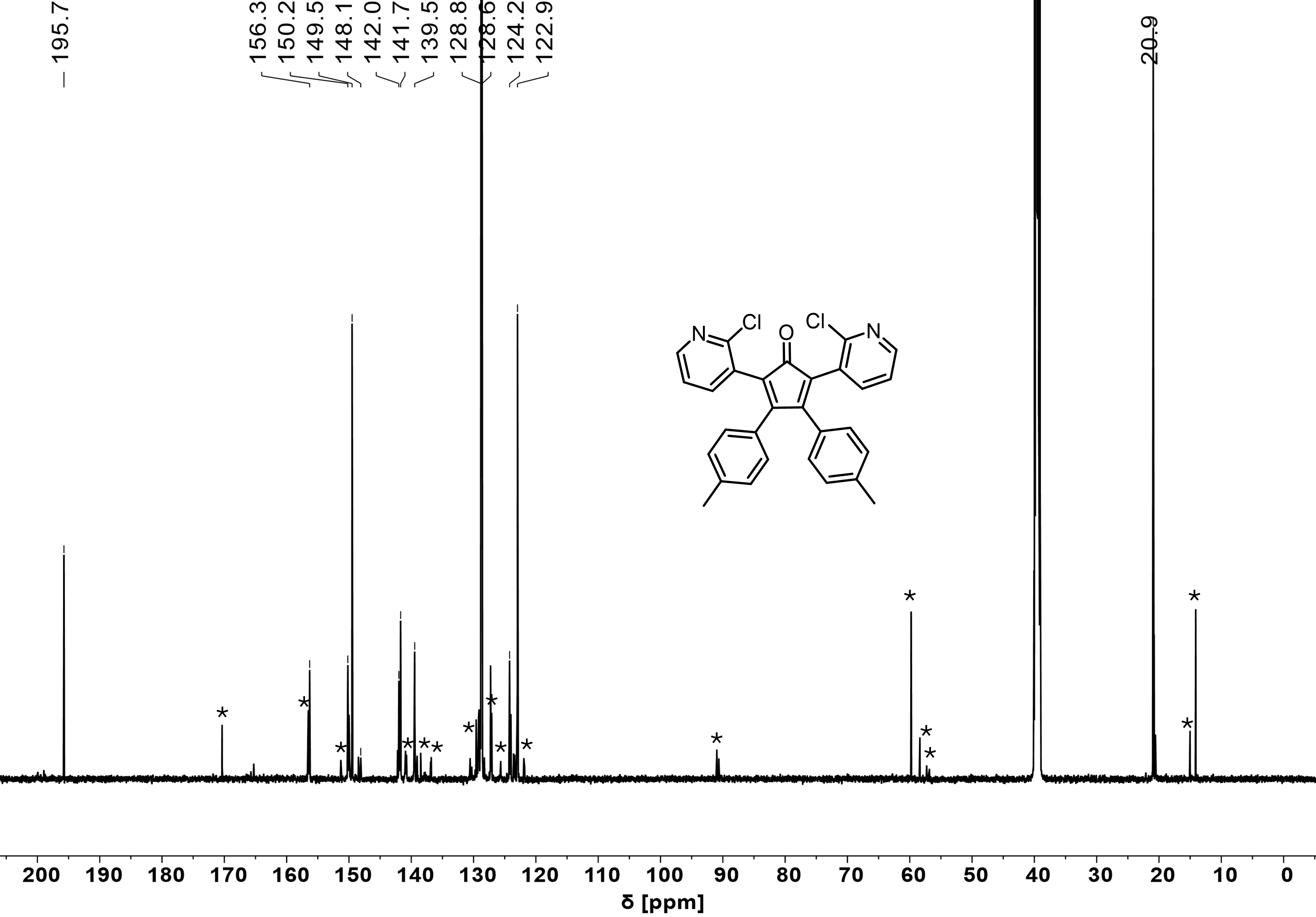


**Figure S20** ^13^C NMR spectrum (151 MHz, 295 K) of cyclopentadienone **12** in DMSO-d_6_.*impurity


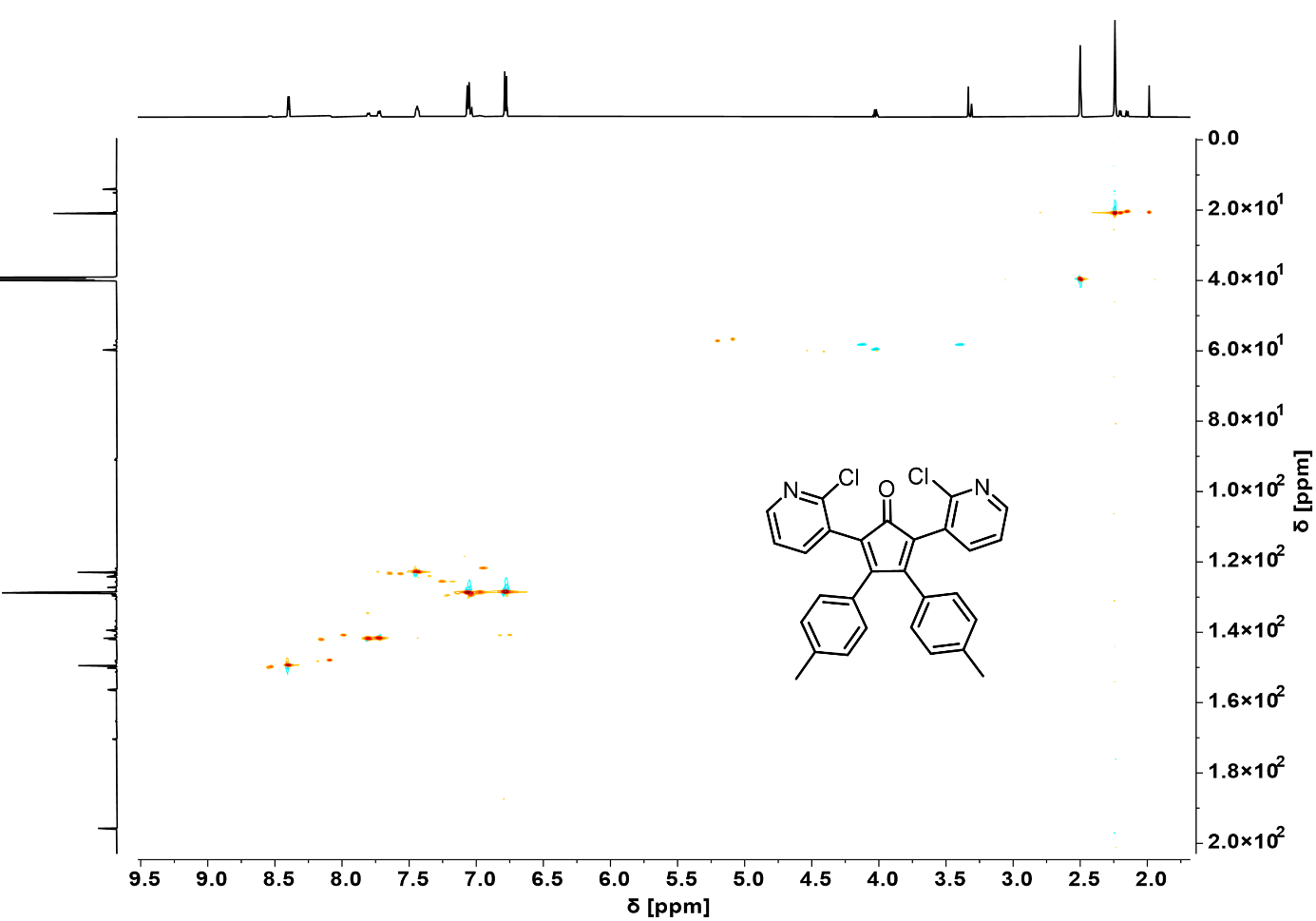


**Figure S21** ^1^H-^13^C-HSQC NMR spectrum (600 MHz, 151 MHz, 295 K) of cyclopentadienone **12** in DMSO-d_6_.


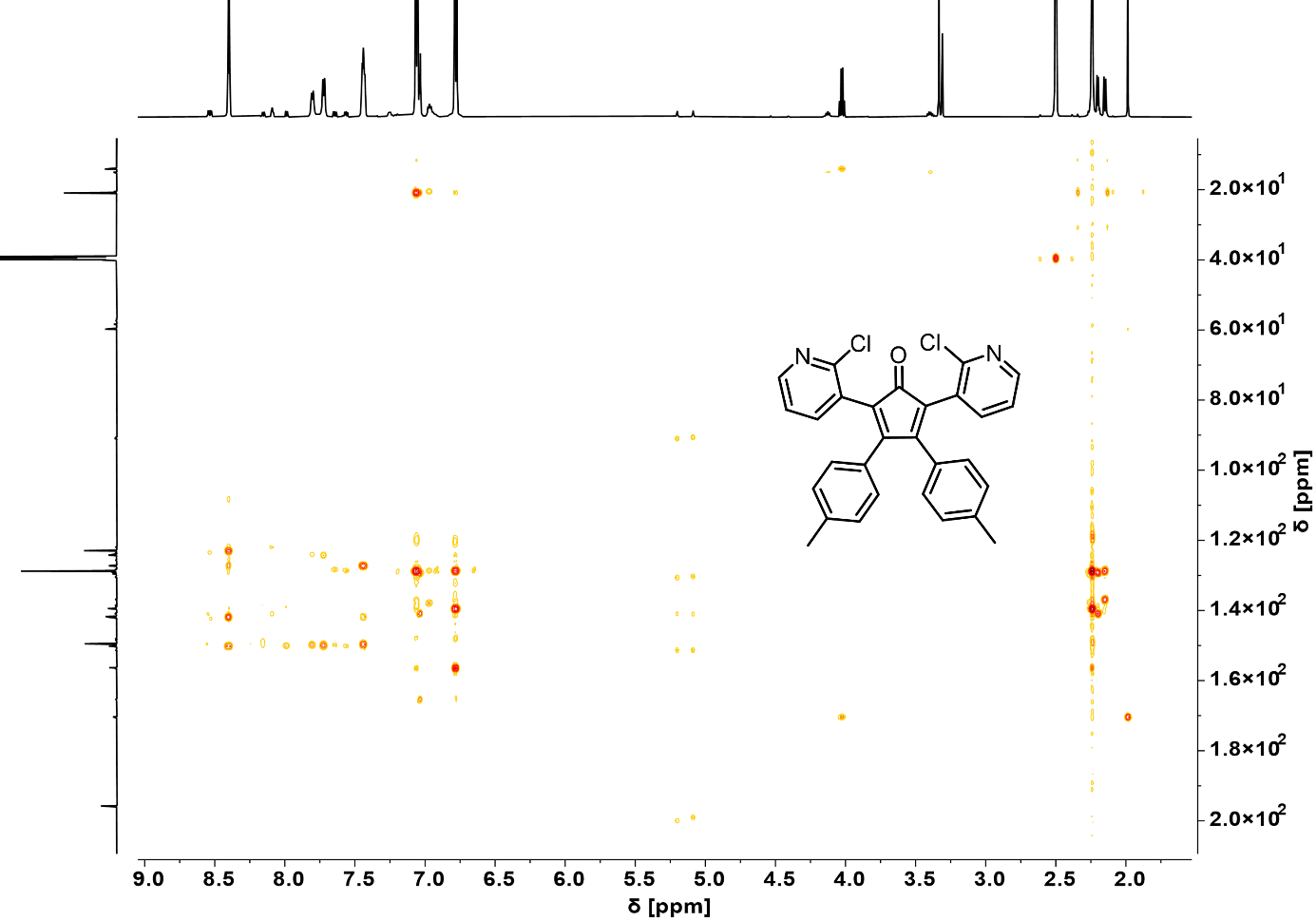


**Figure S22** ^1^H-^13^C-HMBC NMR spectrum (600 MHz, 151 MHz, 295 K) of cyclopentadienone **12** in DMSO-d_6_.


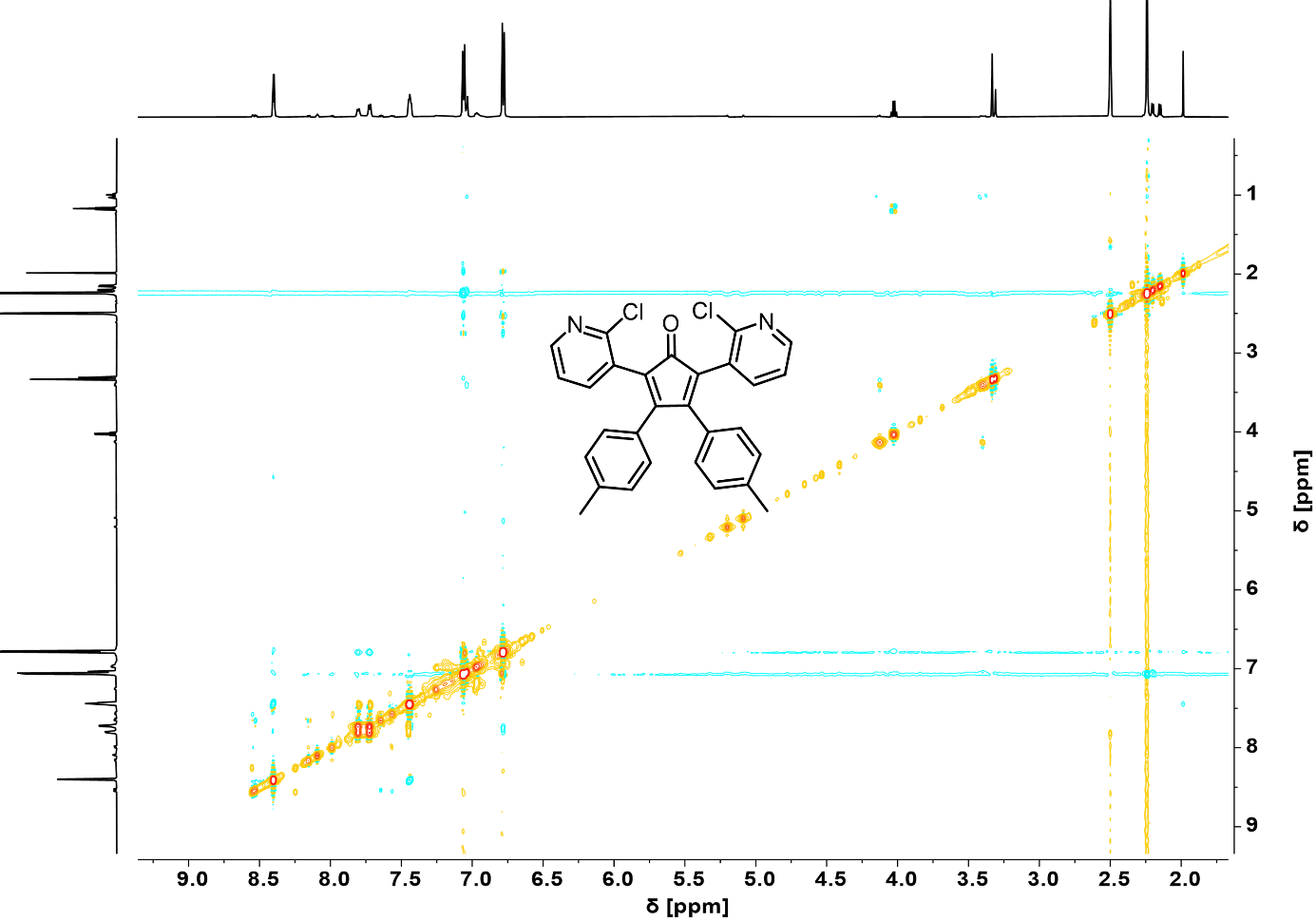


**Figure S23** ^1^H-^1^H-ROESY NMR spectrum (600 MHz, 295 K) of cyclopentadienone **12** in DMSO-d_6_.


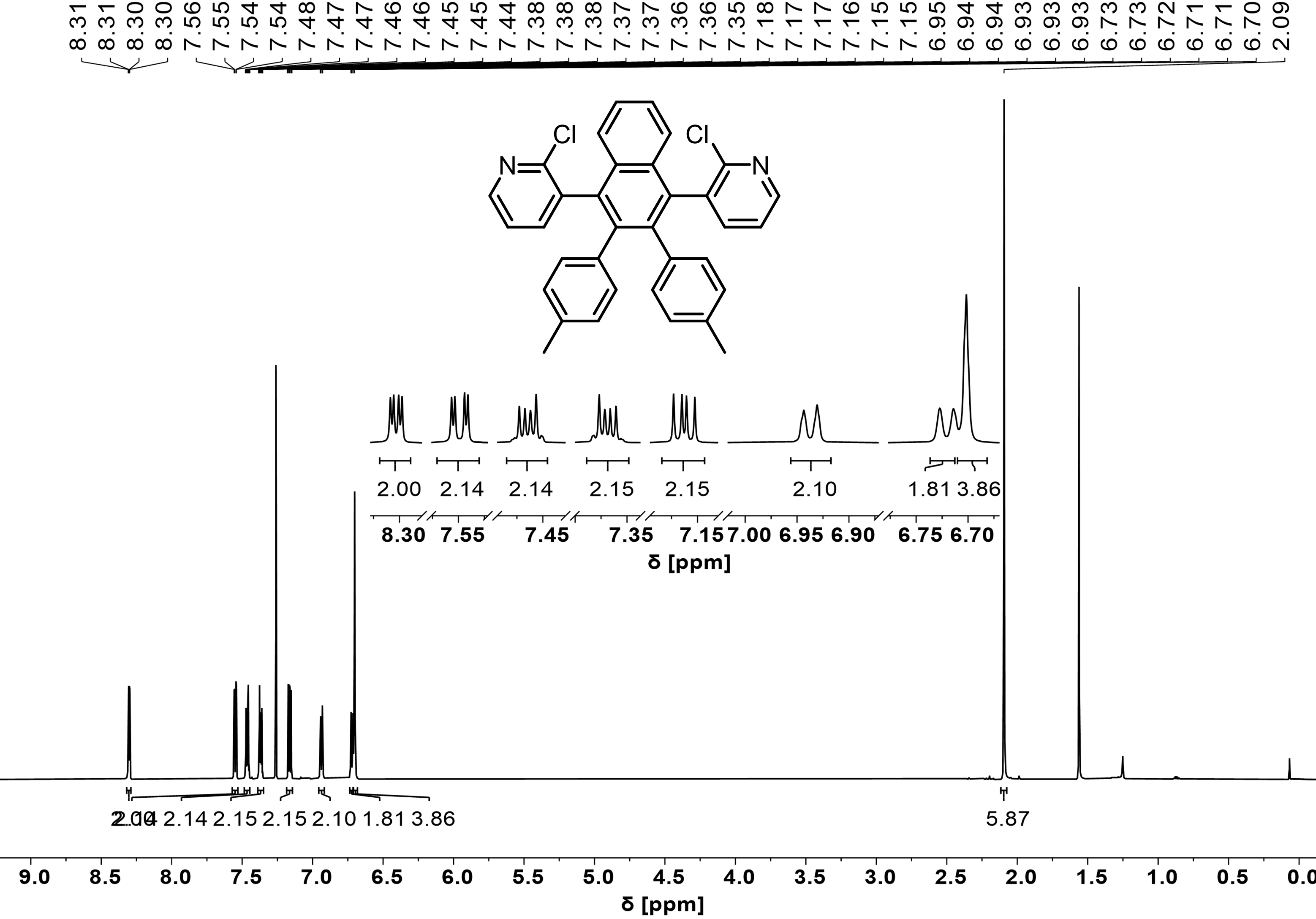


**Figure S24**. ^1^H NMR spectrum (600 MHz, 295 K) of chloropyridine **1** in CDCl_3_.


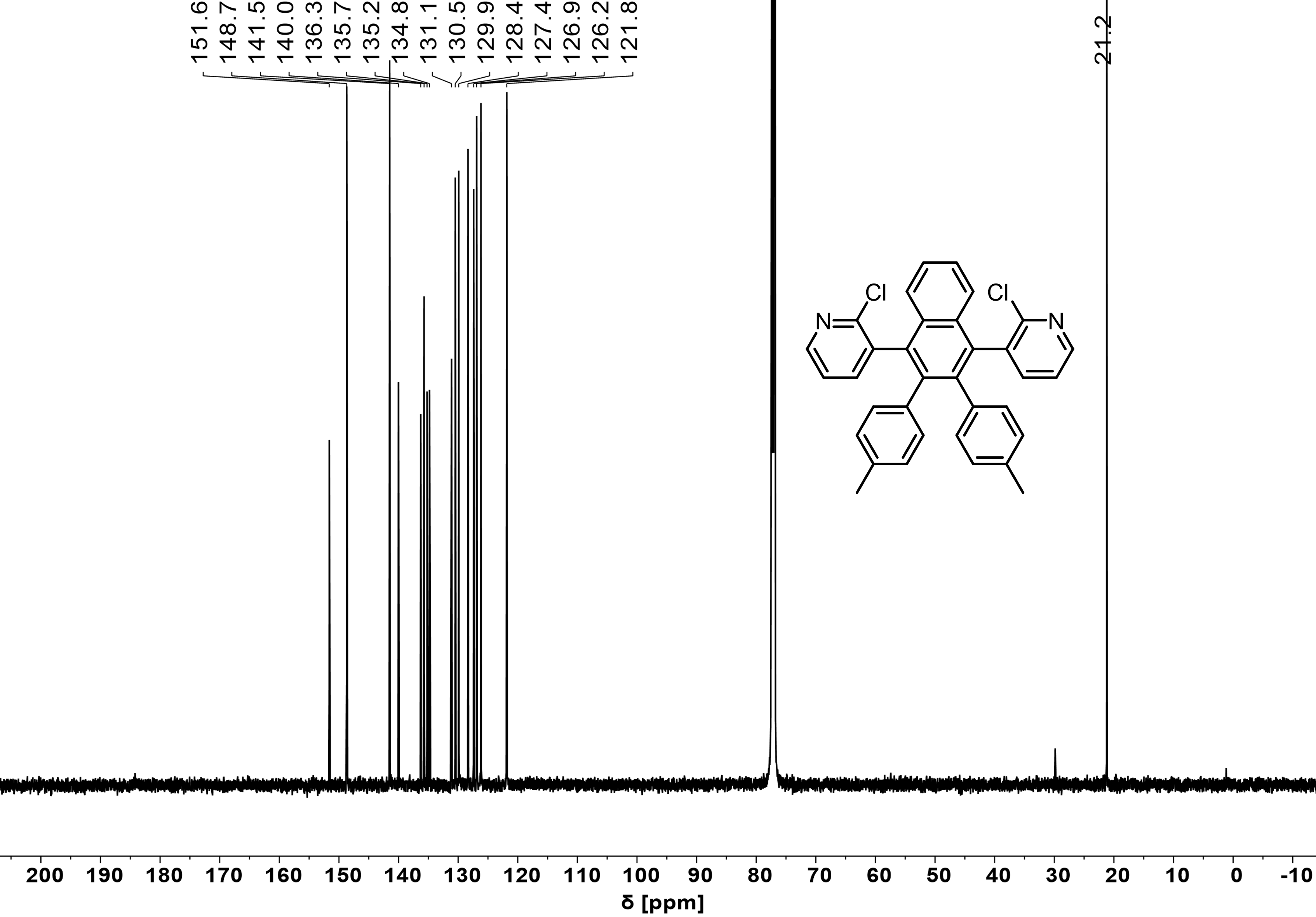


**Figure S25**. ^13^C NMR (151 MHz, 295 K) of chloropyridine **1** in CDCl_3_.


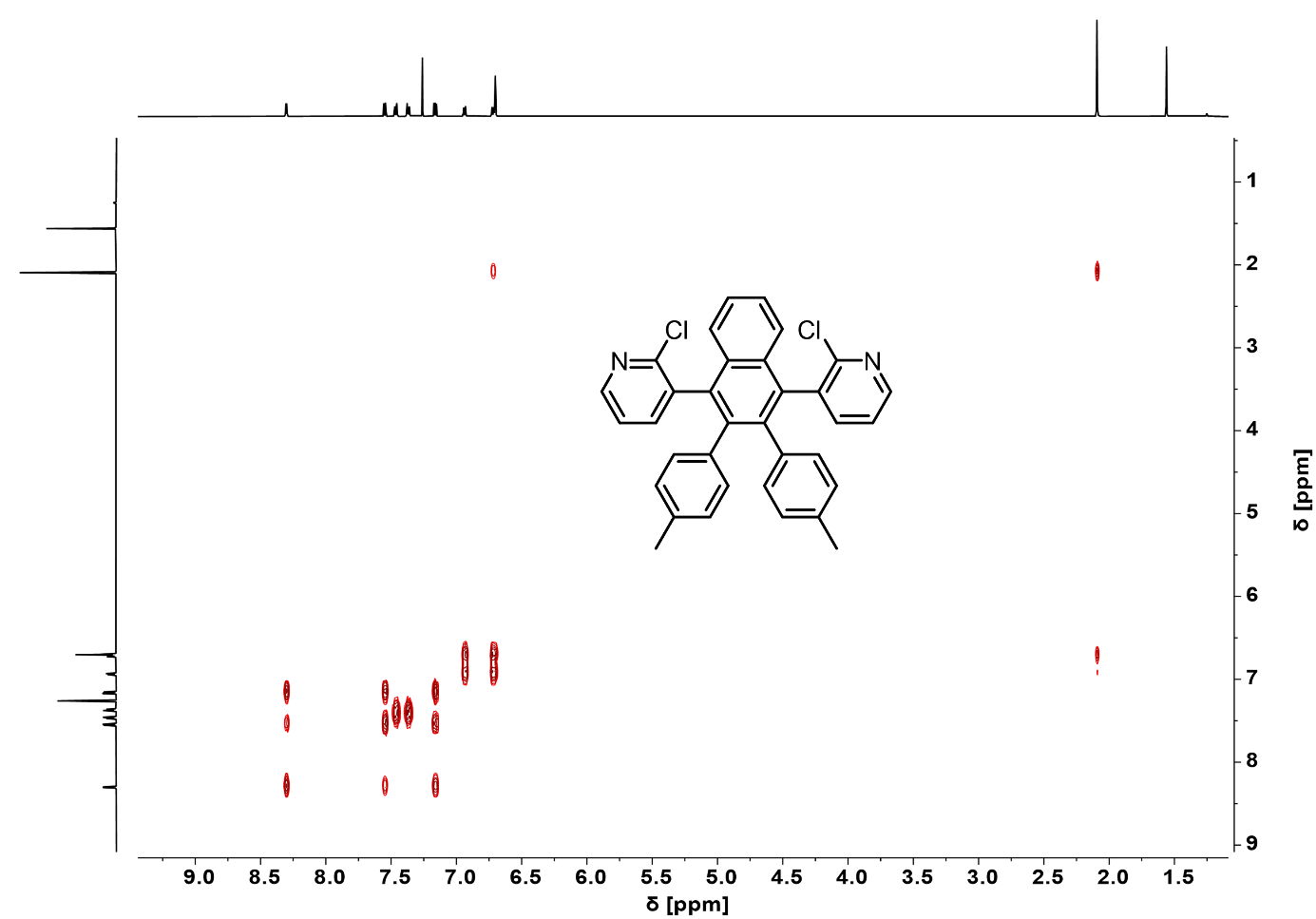


**Figure S26**. ^1^H-^1^H-COSY NMR (600 MHz, 295 K) of chloropyridine **1** in CDCl_3_.


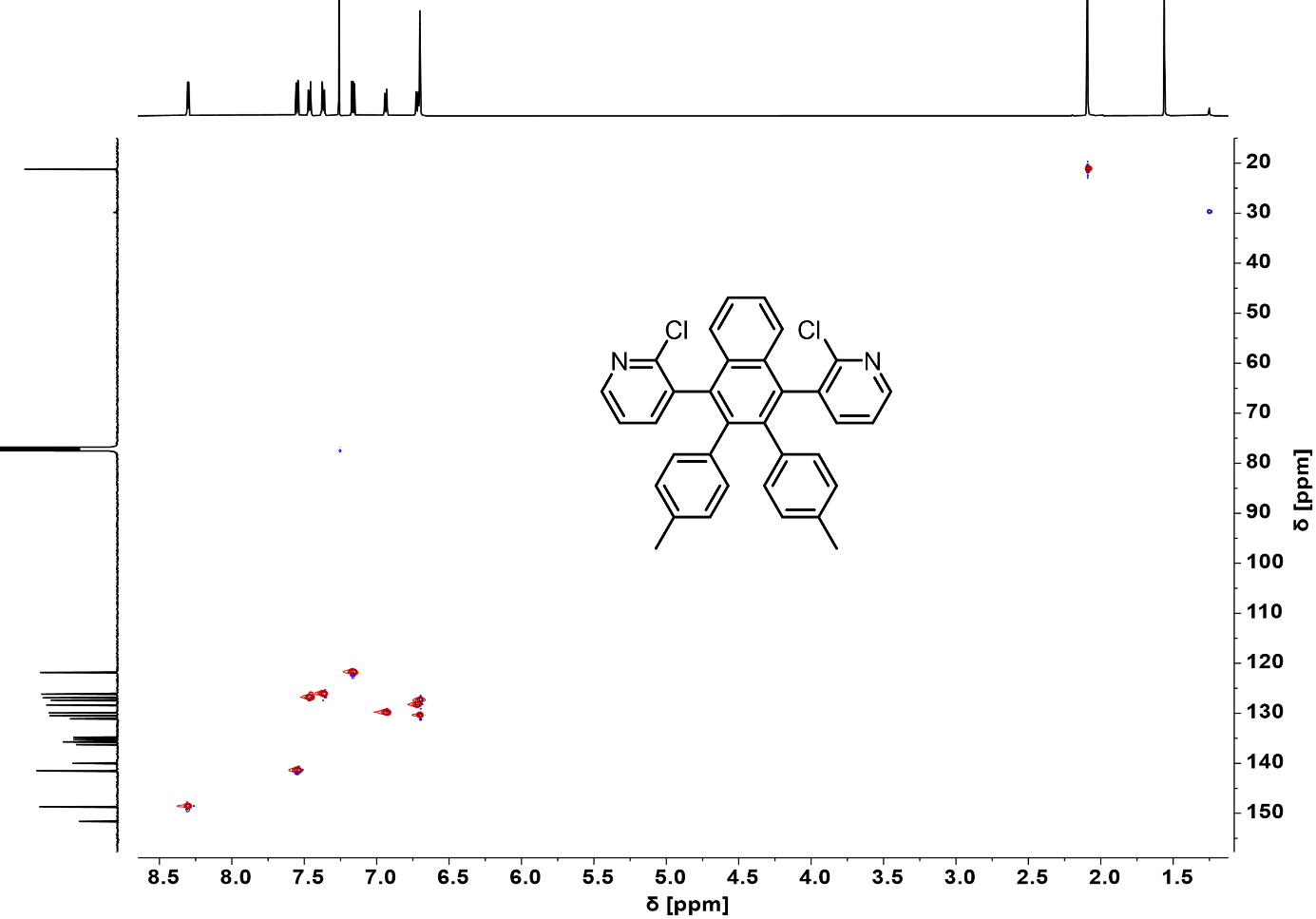


**Figure S27**. ^1^H-^13^C-HSQC (600 MHz, 151 MHz, 295 K) of chloropyridine **1** in CDCl_3_.


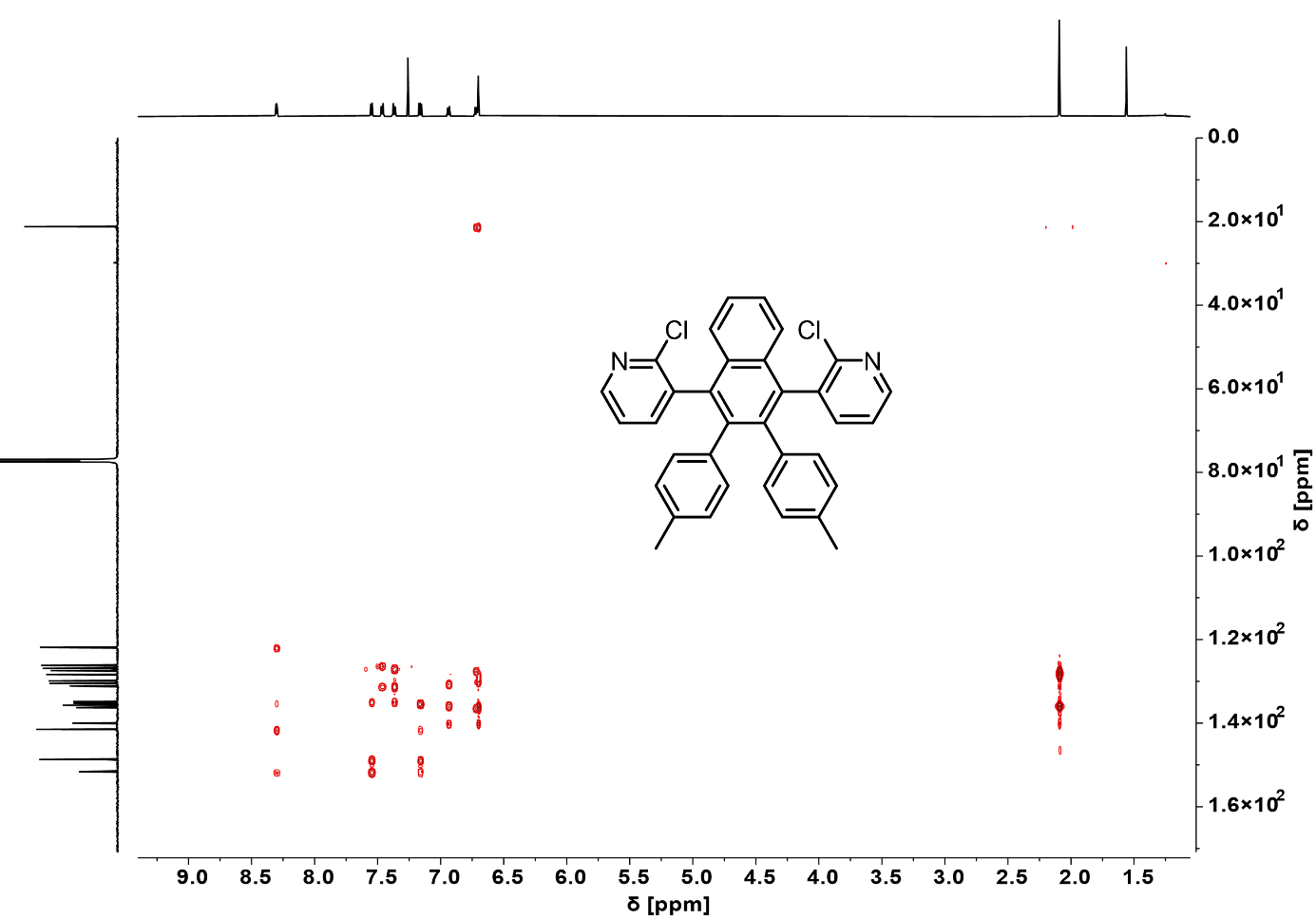


**Figure S28**. ^1^H-^13^C-HMBC NMR spectrum (600 MHz, 151 MHz, 295 K) of chloropyridine **1** in CDCl_3_.


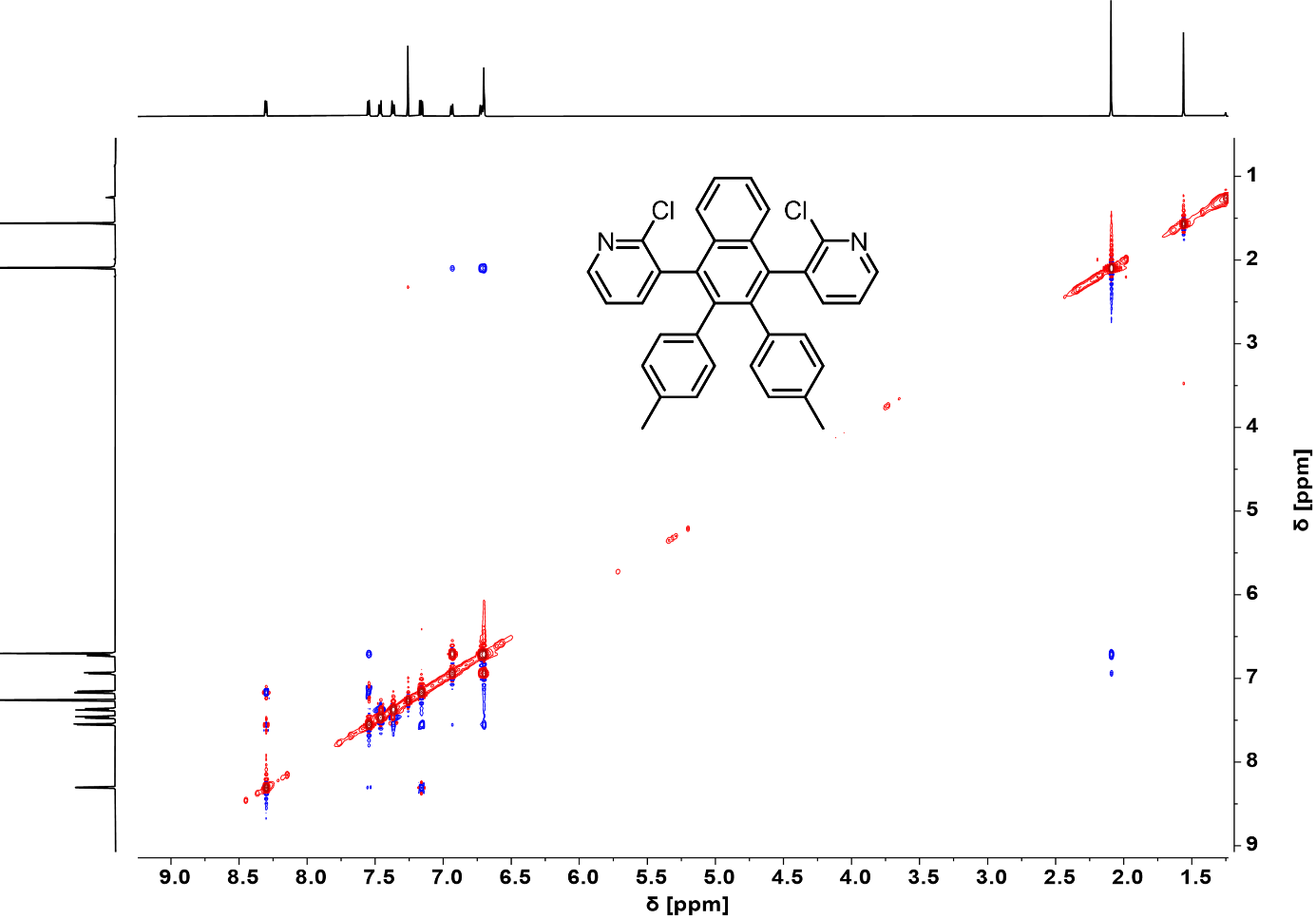


**Figure S29**. ^1^H-^1^H-NOESY NMR spectrum (600 MHz, 295 K) of chloropyridine **1** in CDCl_3_.


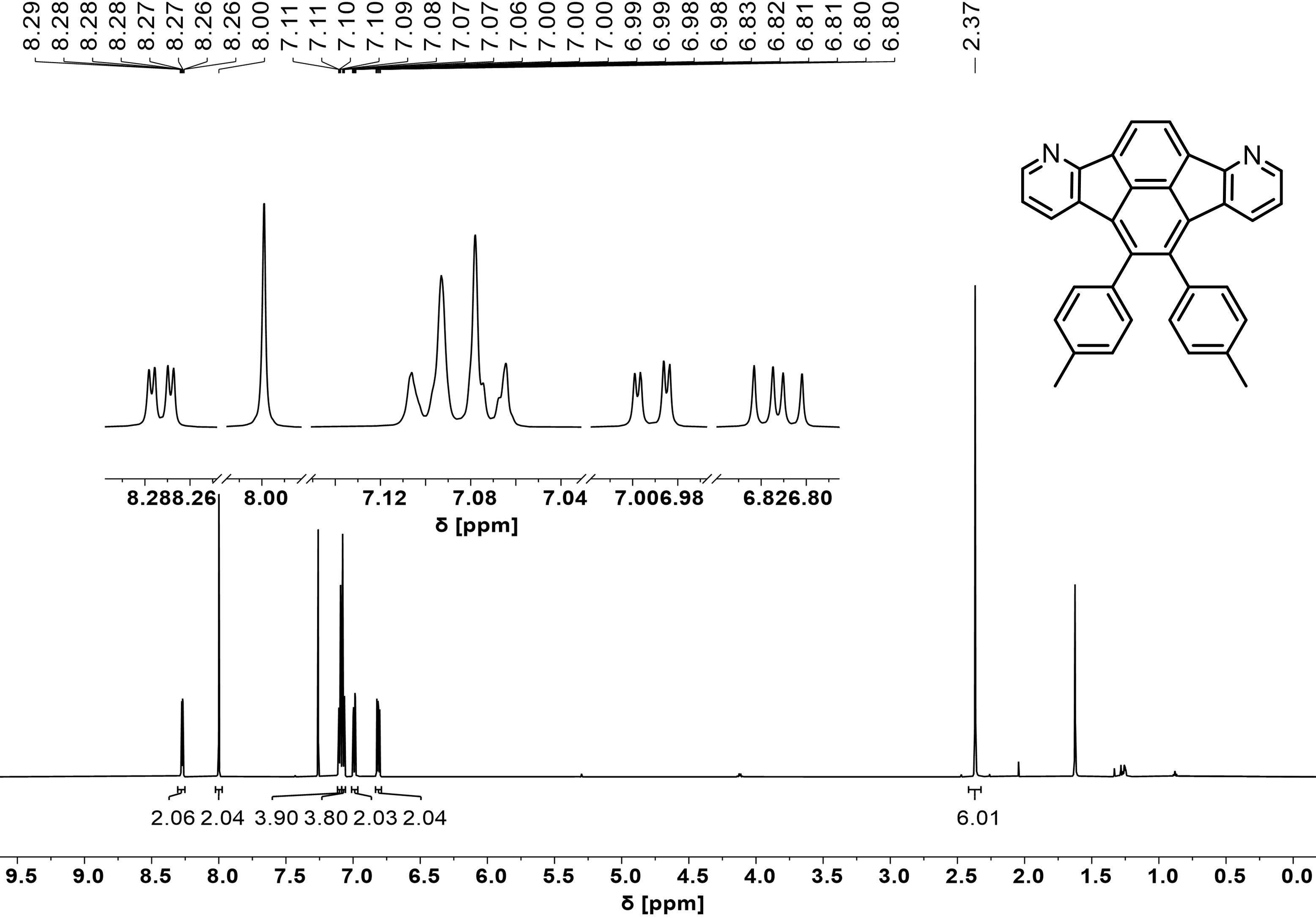


**Figure S30** ^1^H NMR spectrum (600 MHz, 295 K) of fluoranthene **2** in CDCl_3_.


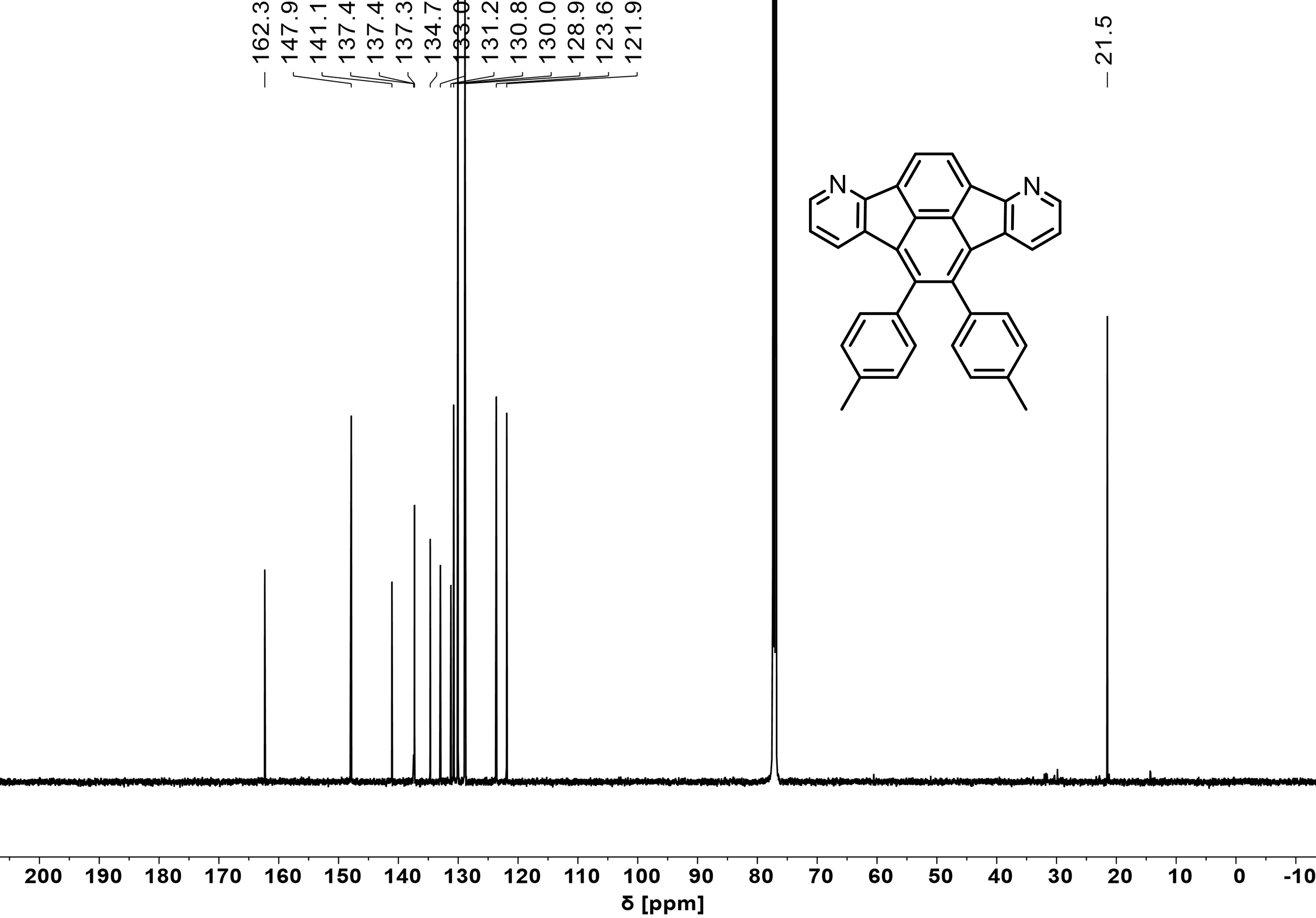


**Figure S31** ^13^C NMR spectrum (151 MHz, 295 K) of fluoranthene **2** in CDCl_3_.


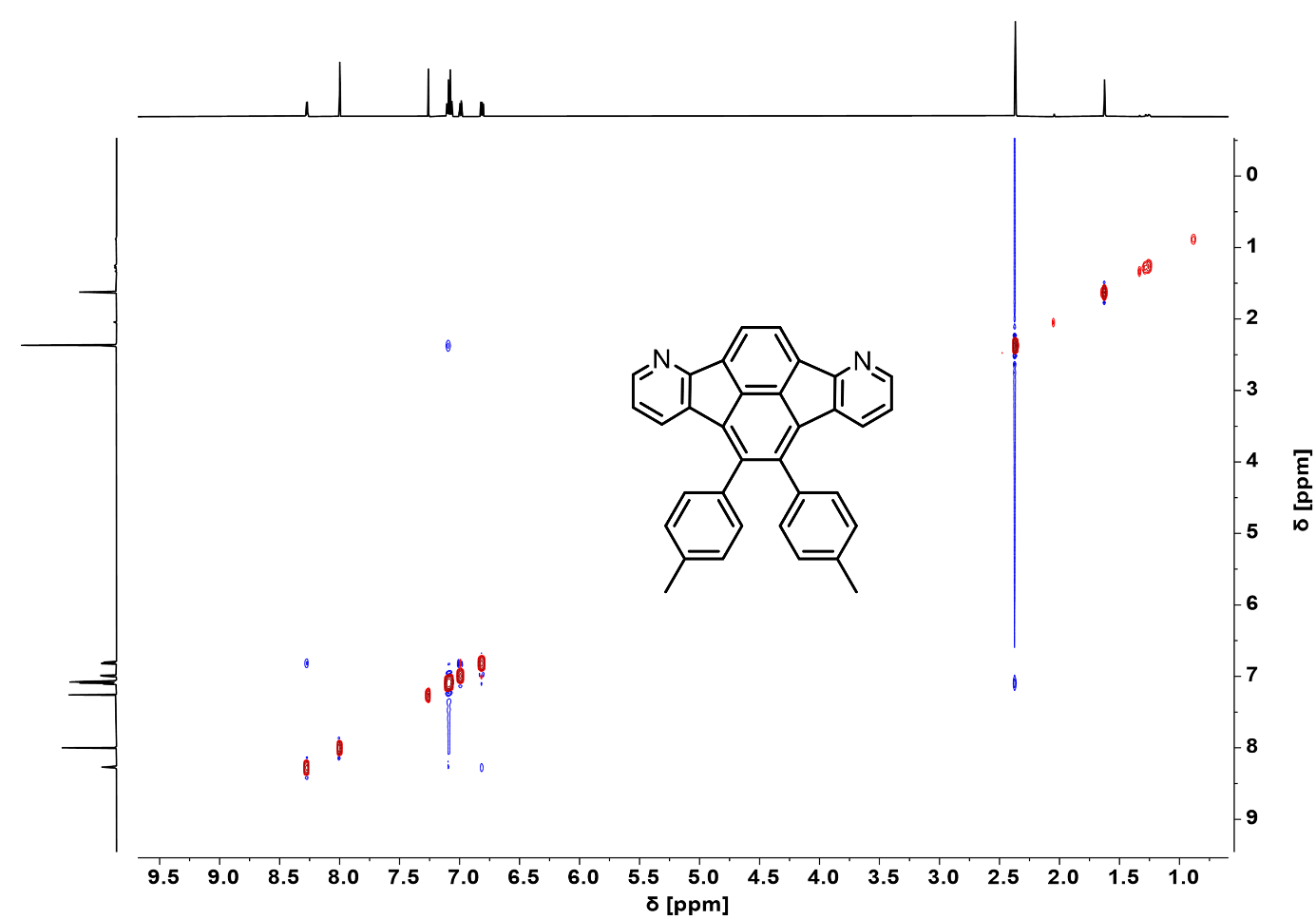


**Figure S32** ^1^H-^1^H NOESY NMR spectrum (600 MHz, 295 K) of fluoranthene **2** in CDCl_3_.


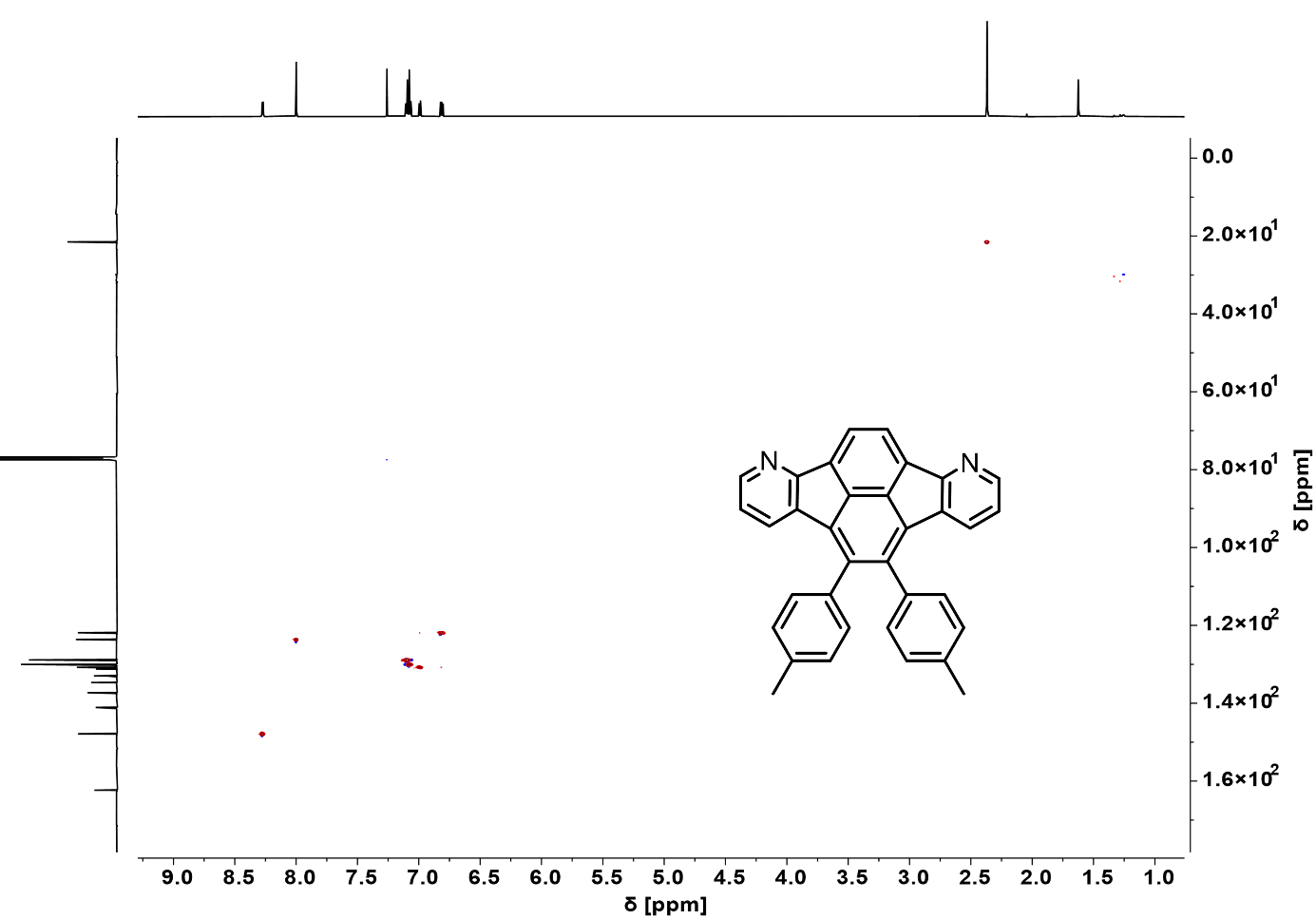


**Figure S33** ^1^H-^13^C HSQC NMR spectrum (600 MHz, 151 MHz, 295 K) of fluoranthene **2** in CDCl_3_.


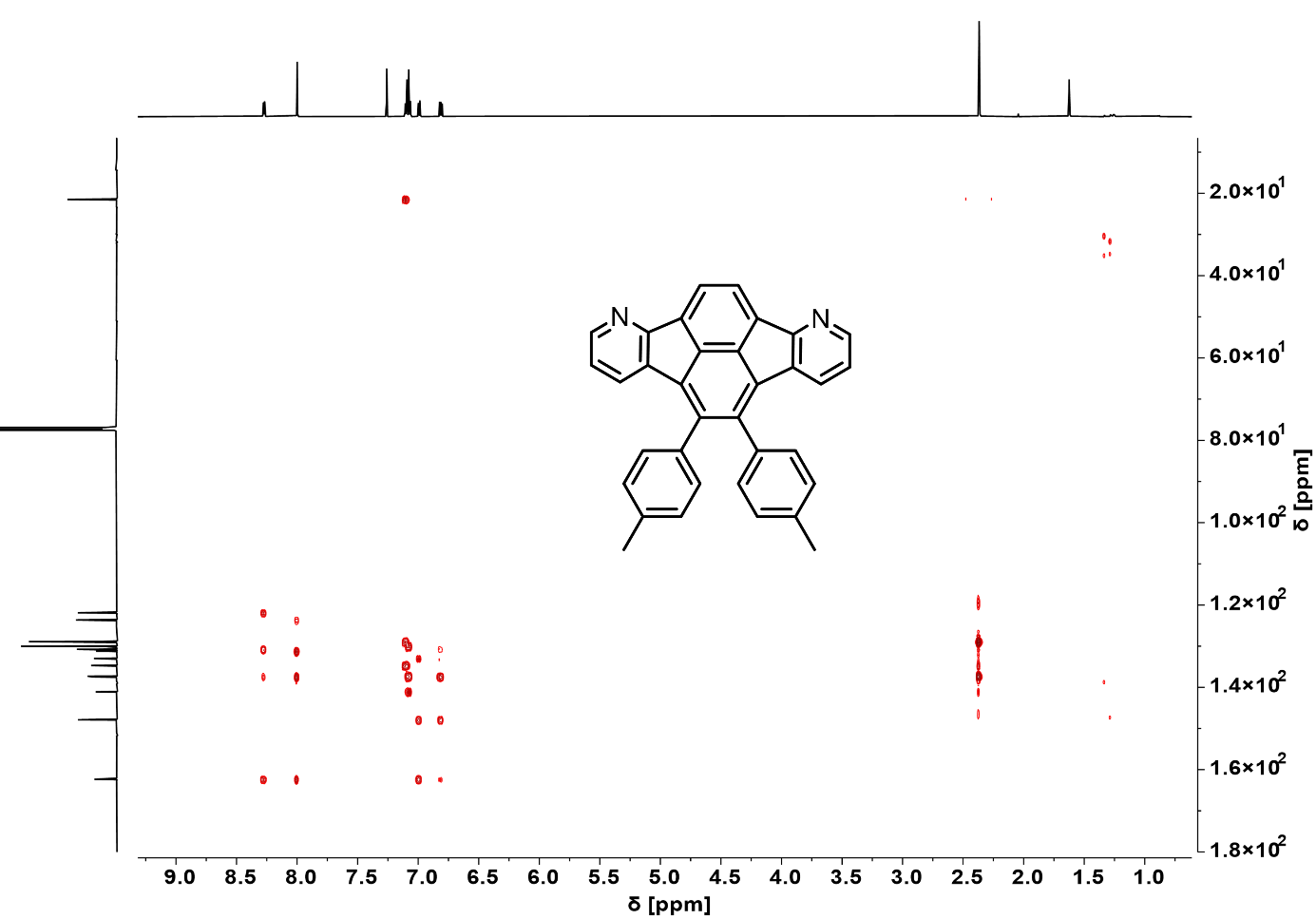


**Figure S34** ^1^H-^13^C HMBC NMR spectrum (600 MHz, 151 MHz, 295 K) of fluoranthene **2** in CDCl_3_.


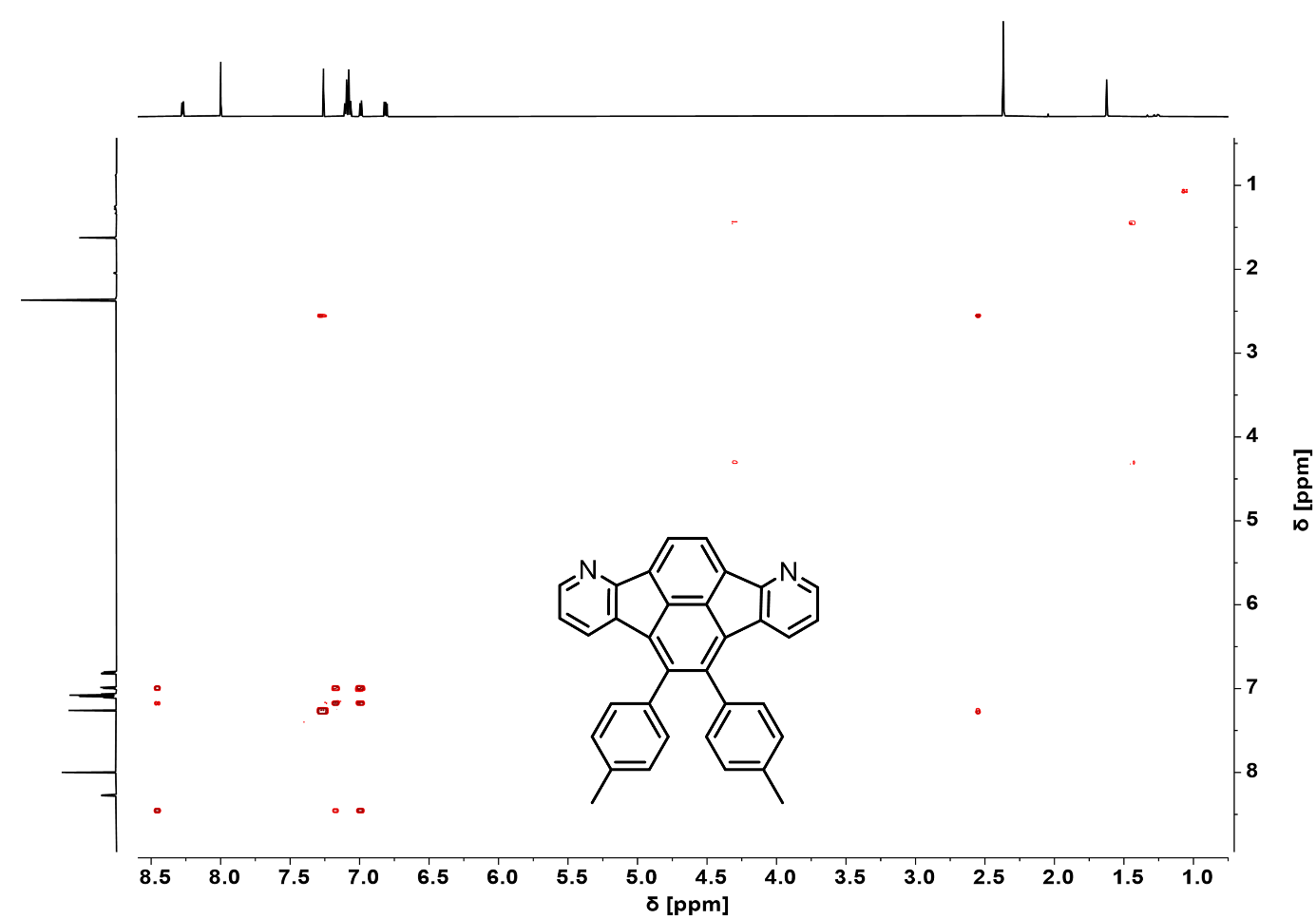


**Figure S35** ^1^H-^1^H COSY NMR spectrum (600 MHz, 295 K) of fluoranthene **2** in CDCl_3_.


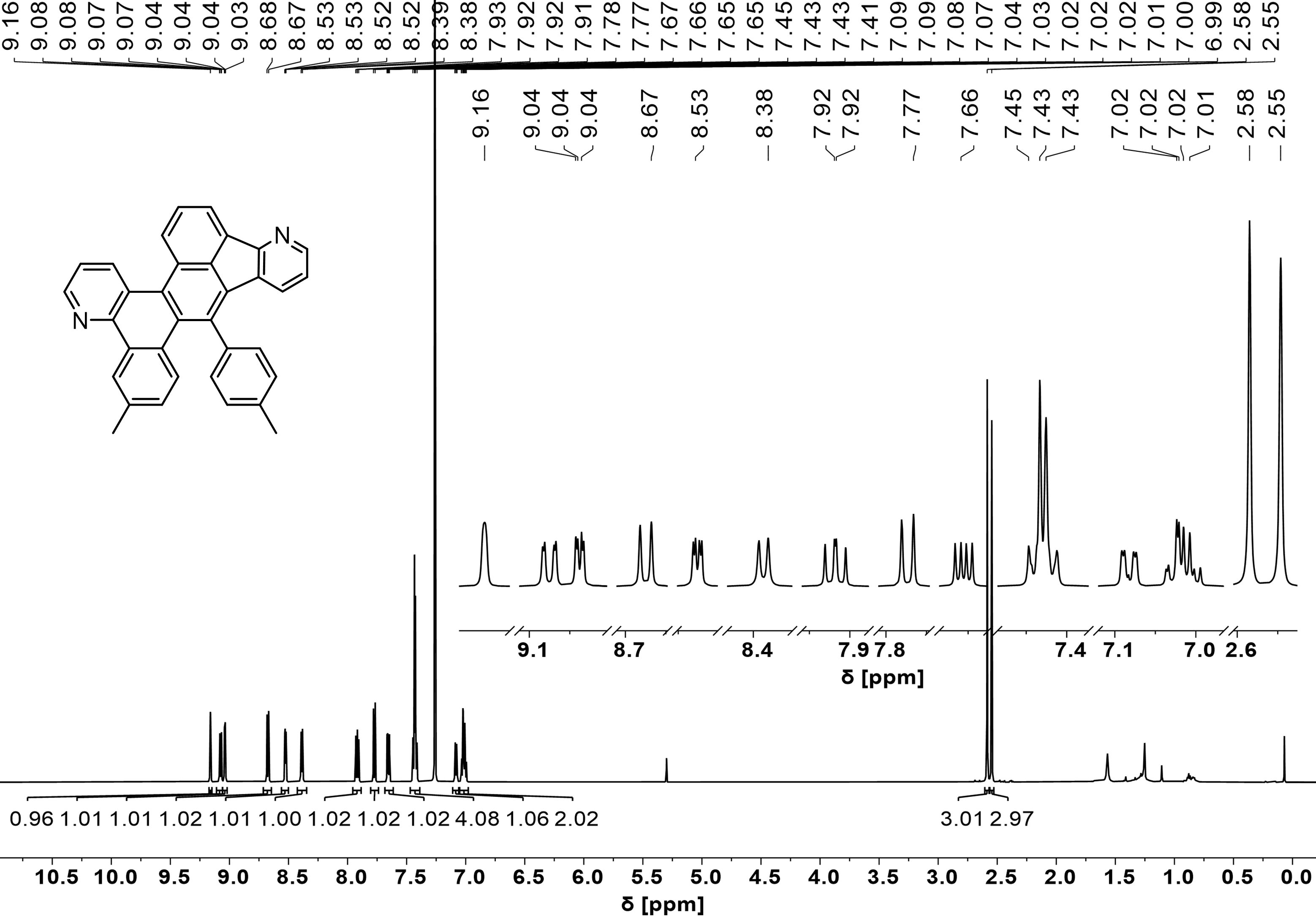


**Figure S36** ^1^H NMR spectrum (600 MHz, 295 K) of compound **3** in CDCl_3_.


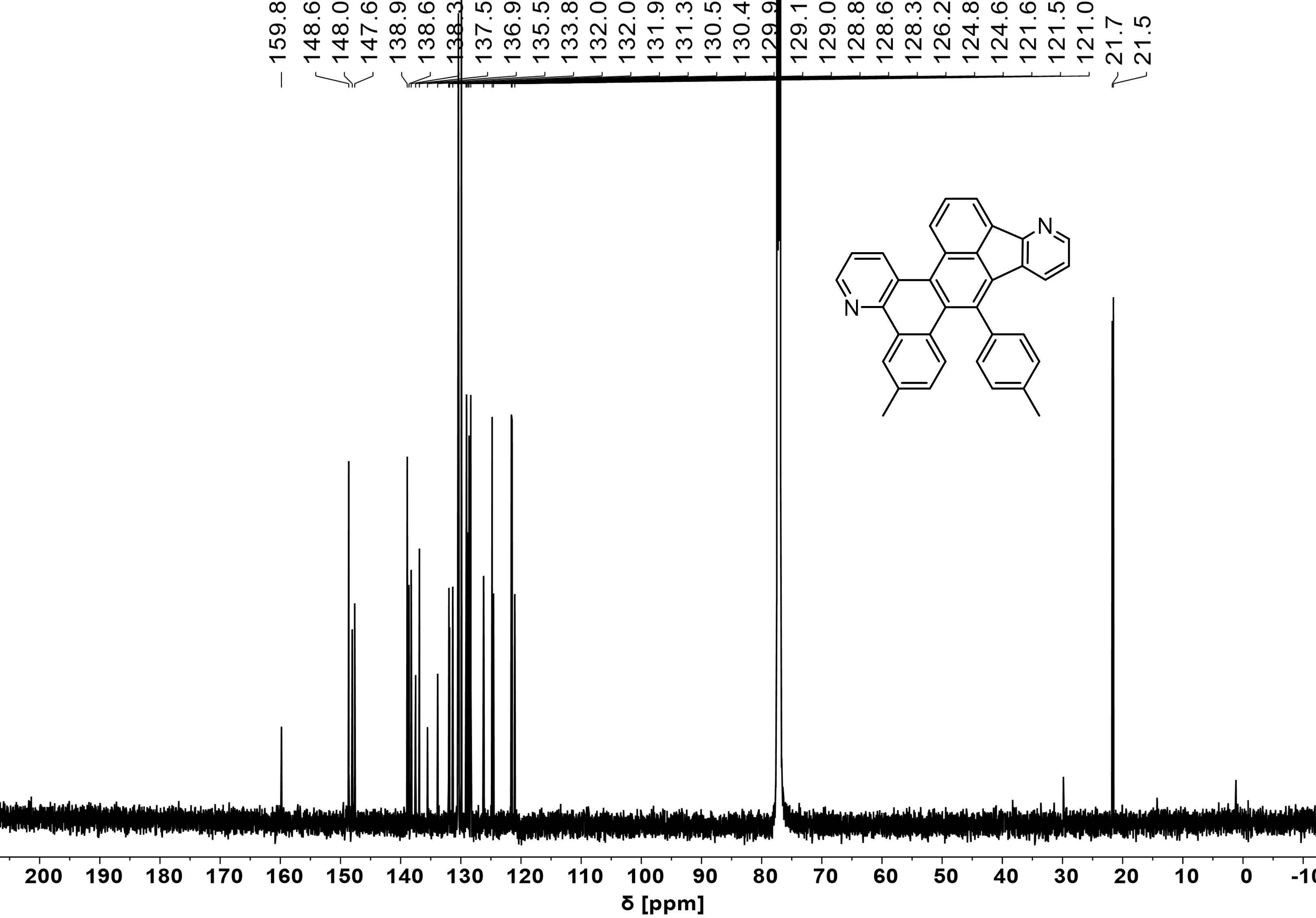


**Figure S37** ^13^C NMR spectrum (600 MHz, 295 K) of compound **3** in CDCl_3_.


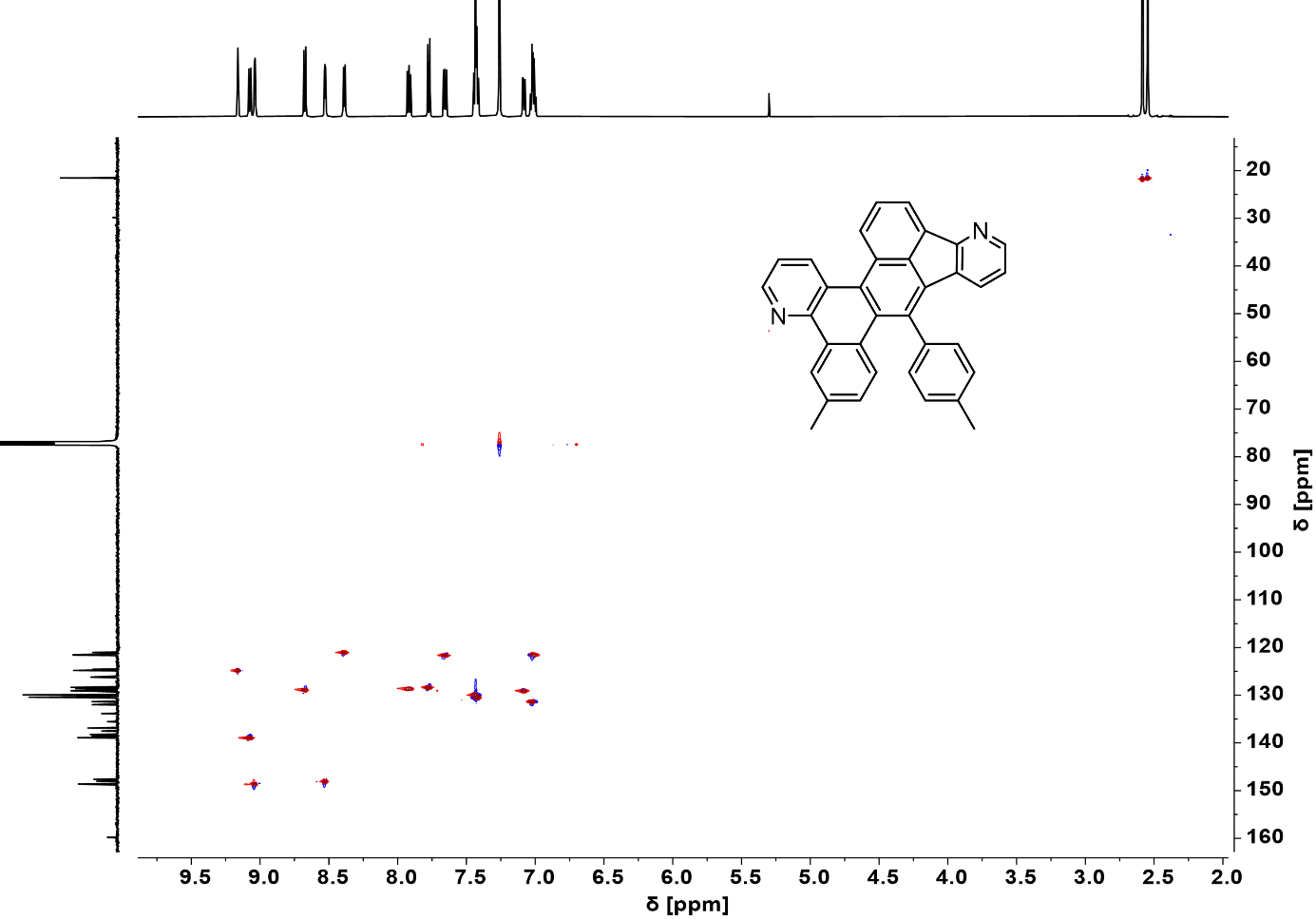


**Figure S38** ^1^H-^13^C HSQC NMR spectrum (600 MHz, 151 MHz, 295 K) of compound **3** in CDCl_3_.


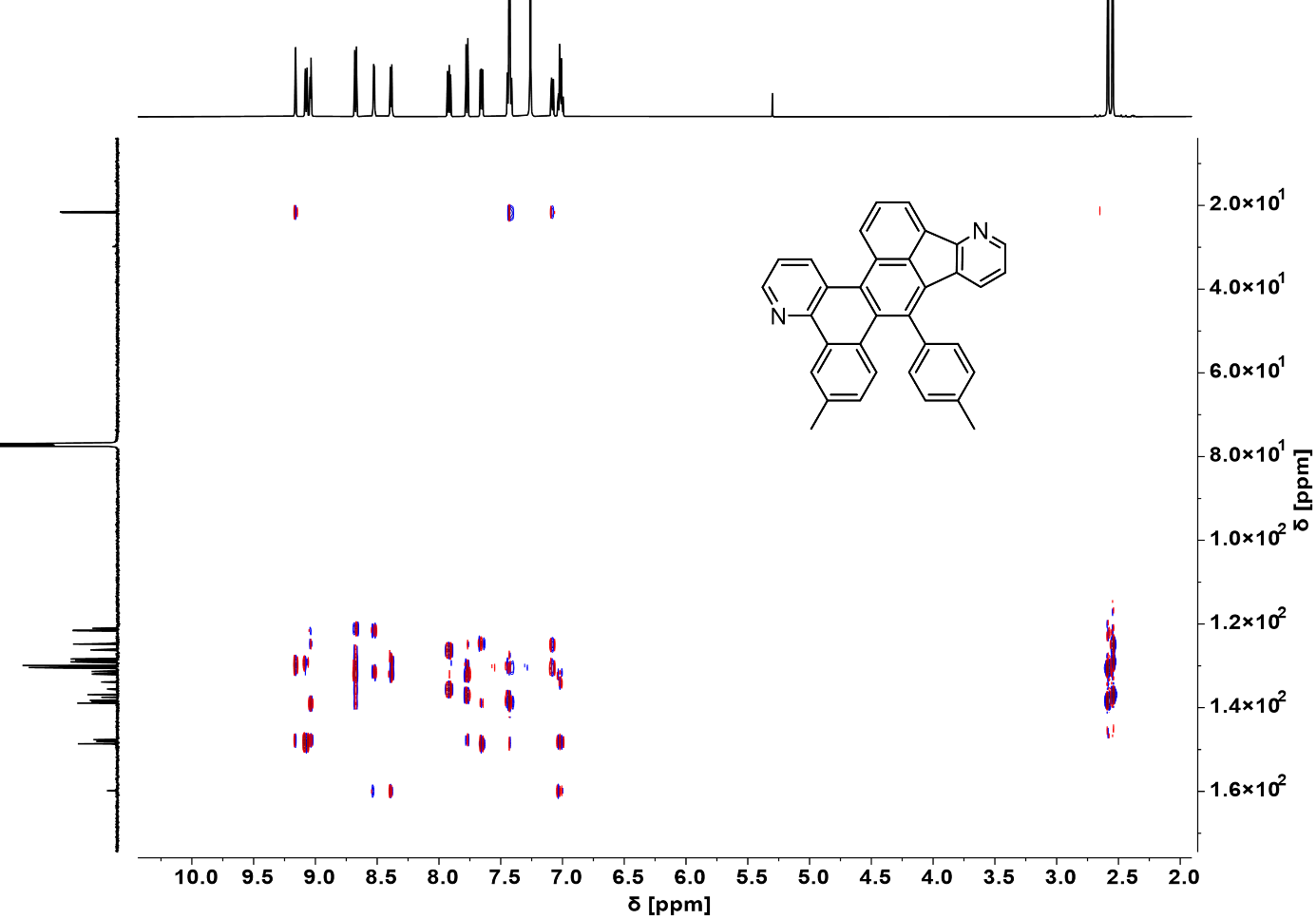


**Figure S39** ^1^H-^13^C HMBC NMR spectrum (600 MHz, 151 MHz, 295 K) of compound **3** in CDCl_3_.


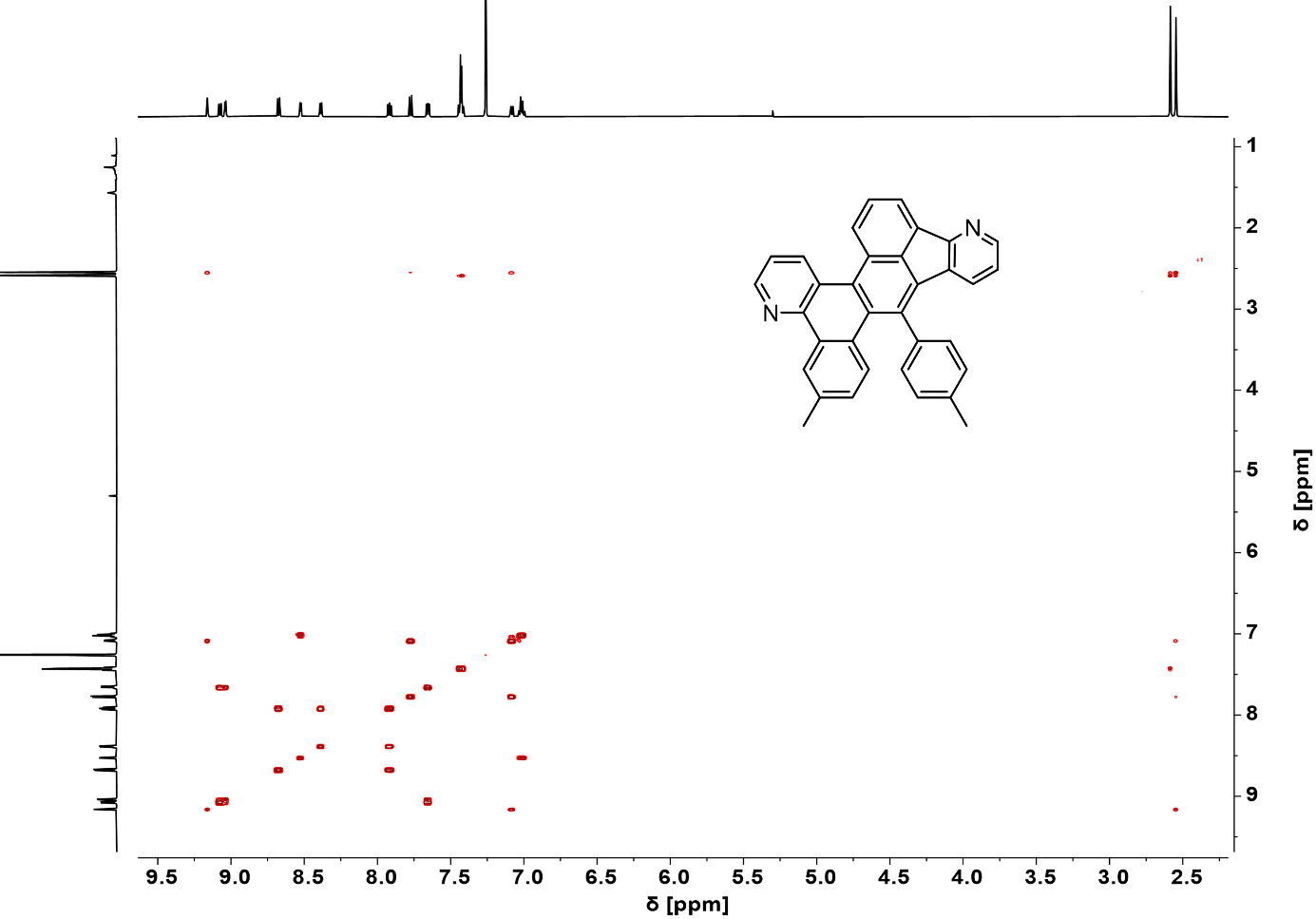


**Figure S40** ^1^H-^1^H COSY NMR spectrum (600 MHz, 295 K) of compound **3** in CDCl_3_.


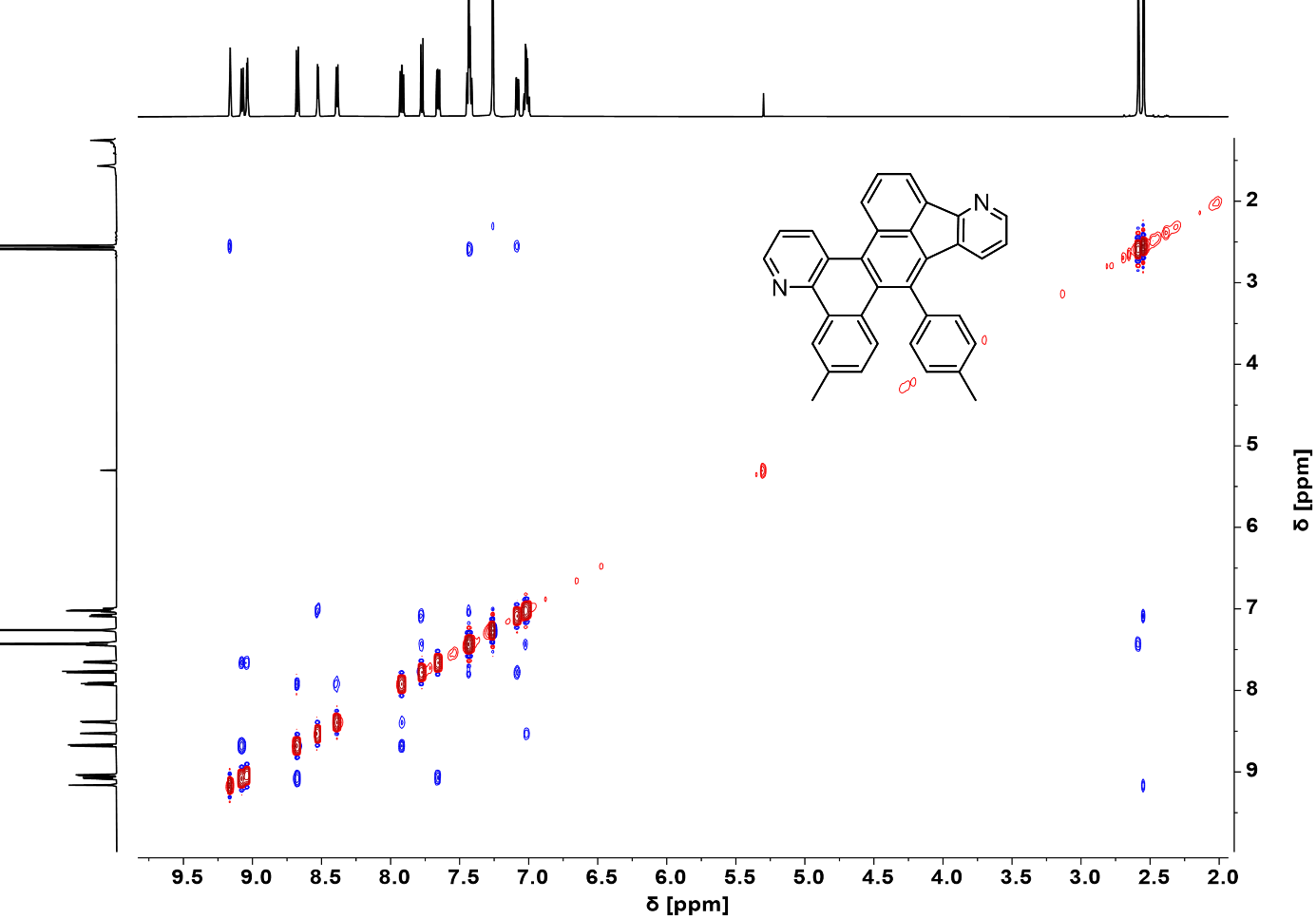


**Figure S41** ^1^H-^1^H NOESY NMR spectrum (600 MHz, 295 K) of compound **3** in CDCl_3_.


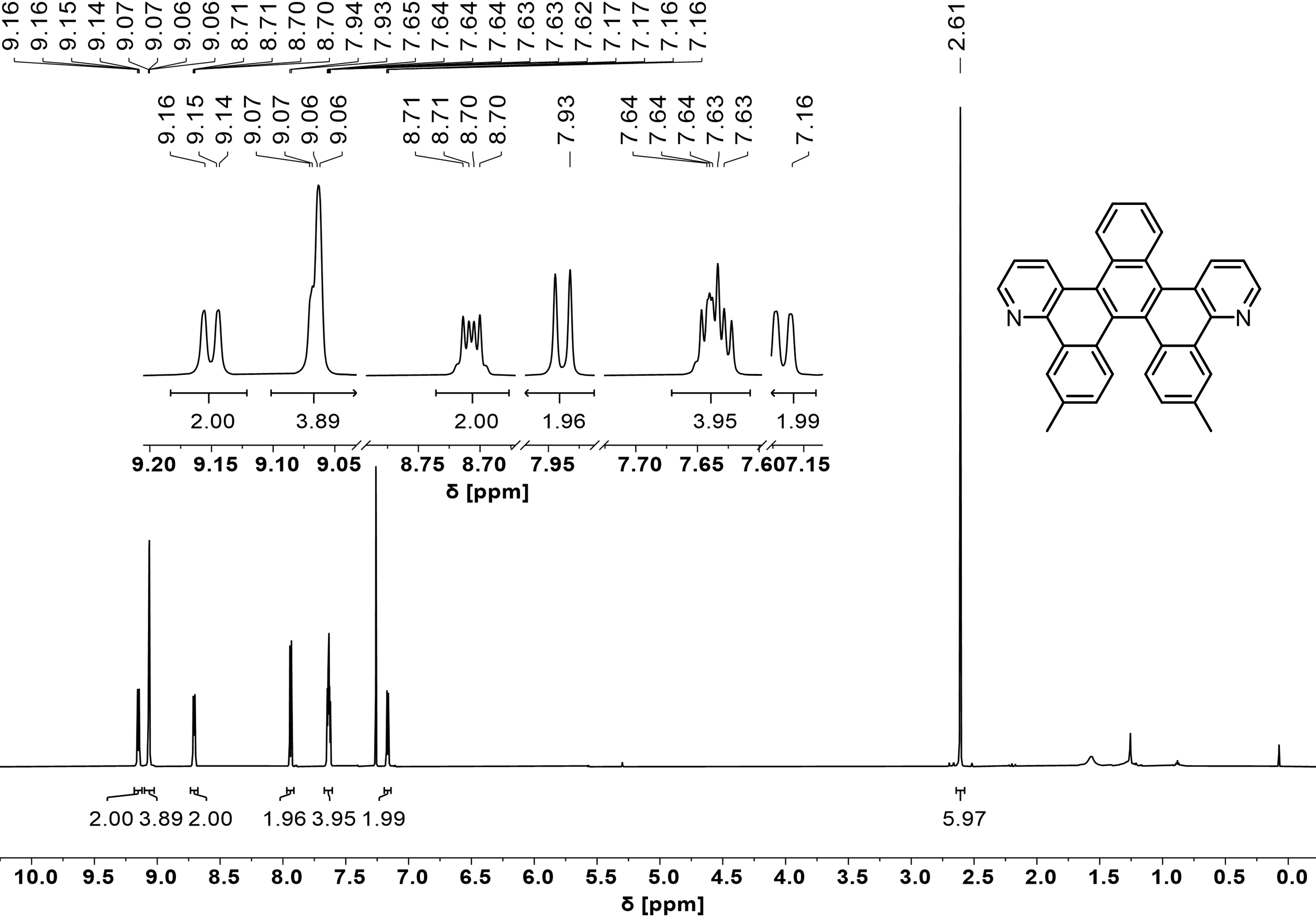


**Figure S42** ^1^H NMR spectrum (700 MHz, 295 K) of pentahelicene **4** in CDCl_3_.


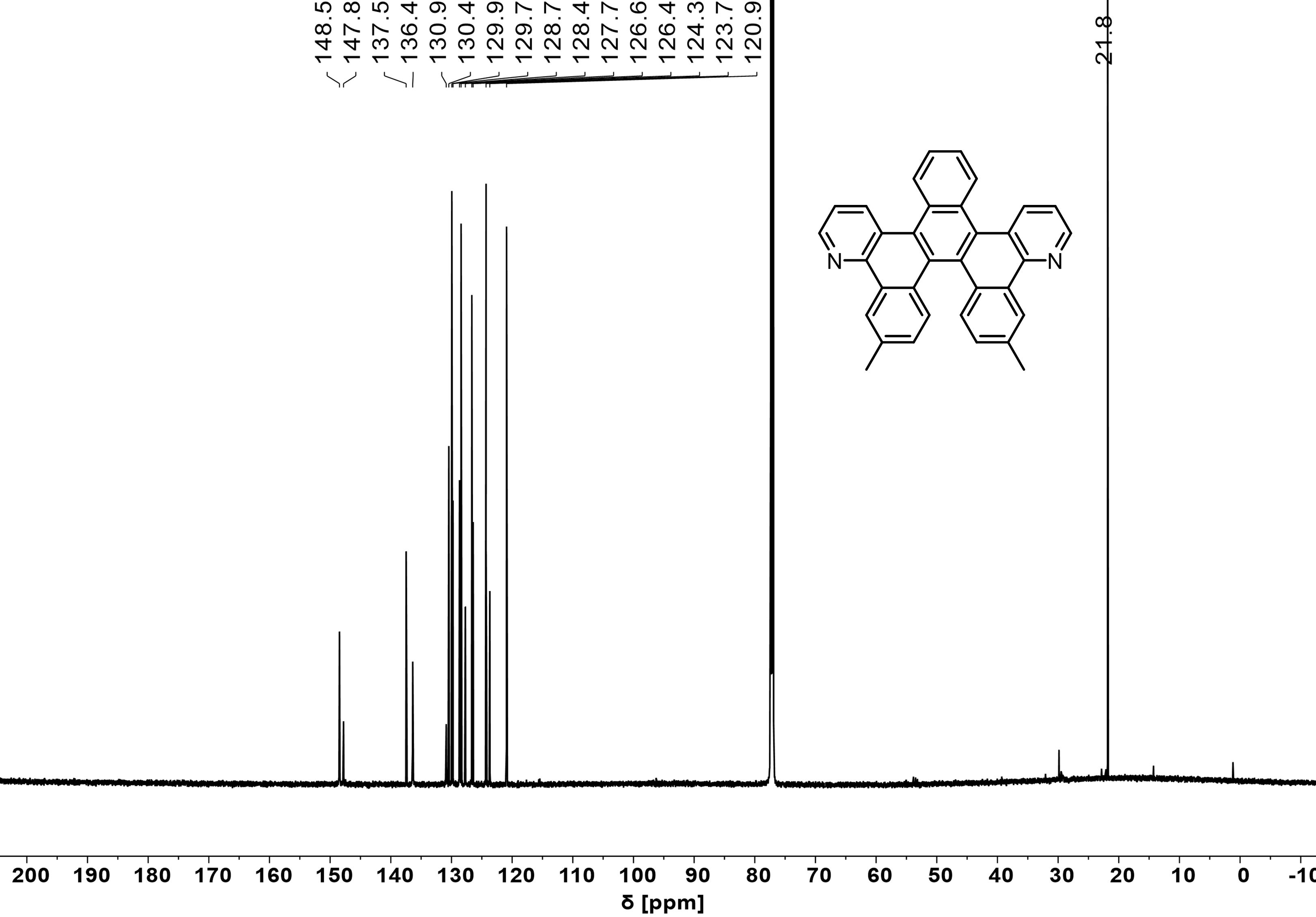


**Figure S43** ^13^C NMR spectrum (176 MHz, 295 K) of pentahelicene **4** in CDCl_3_.


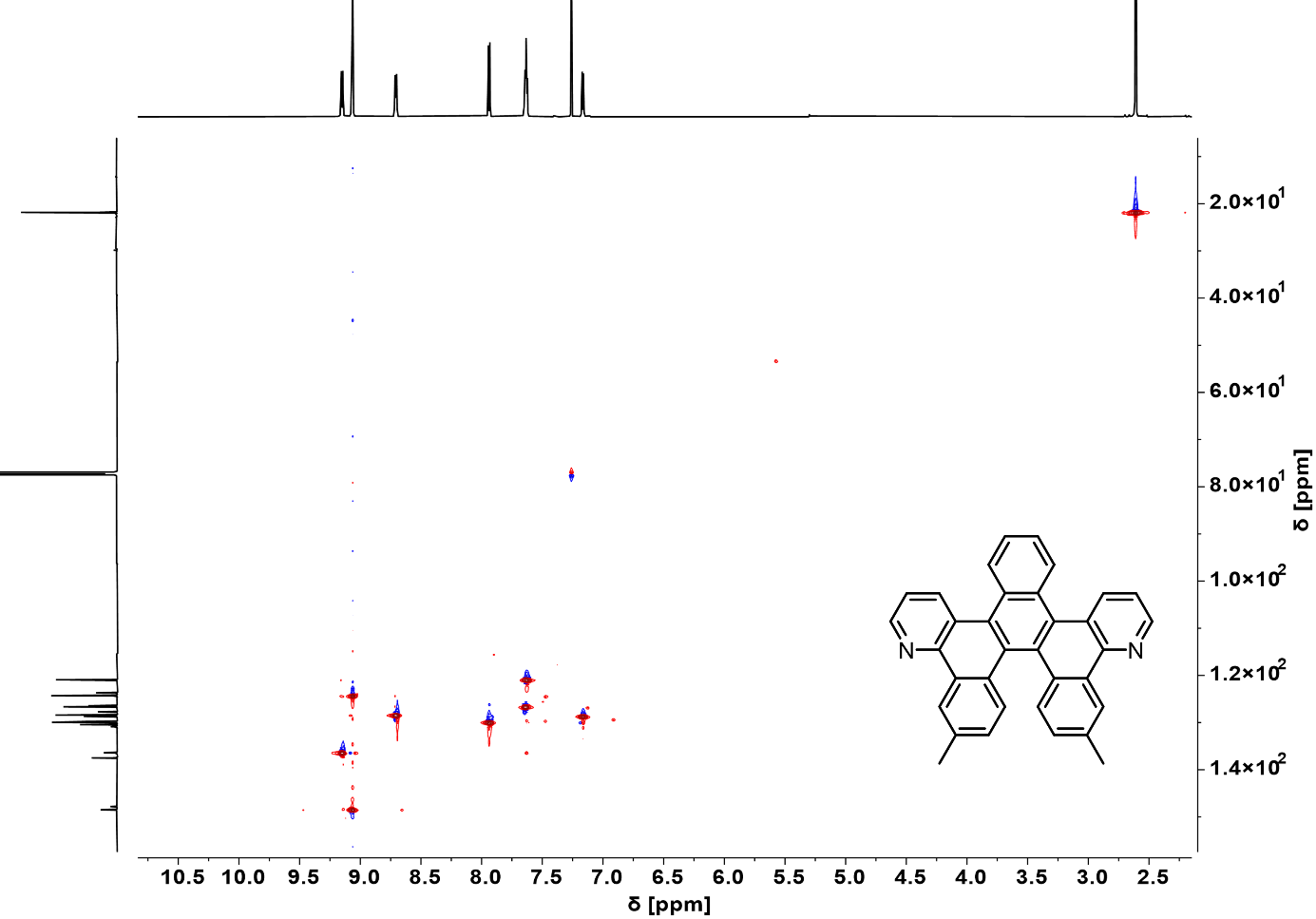


**Figure S44** ^1^H-^13^C HSQC NMR spectrum (700 MHz, 176 MHz, 295 K) of pentahelicene **4** in CDCl_3_.


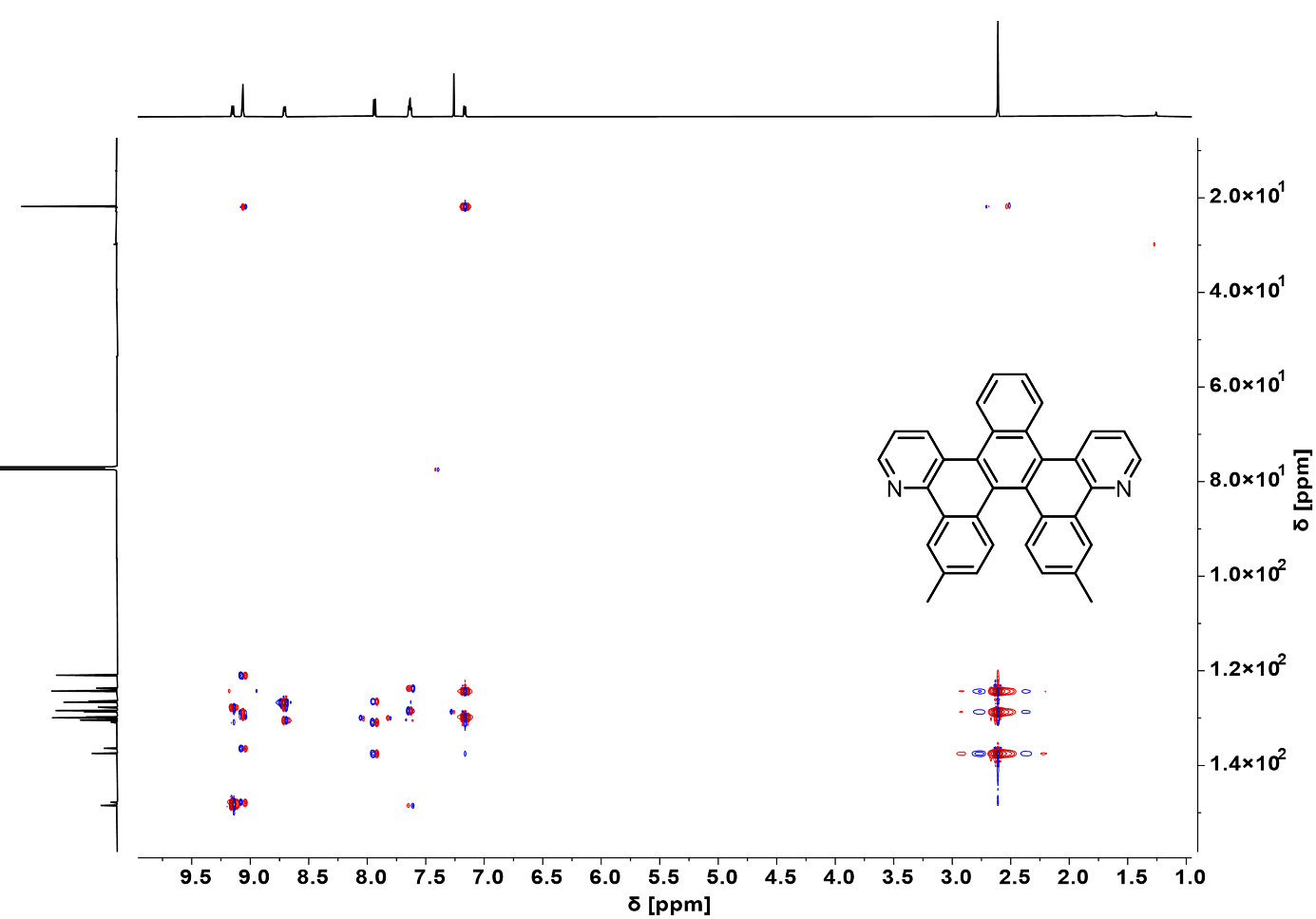


**Figure S45** ^1^H-^13^C HMBC NMR spectrum (700 MHz, 176 MHz, 295 K) of pentahelicene **4** in CDCl_3_.


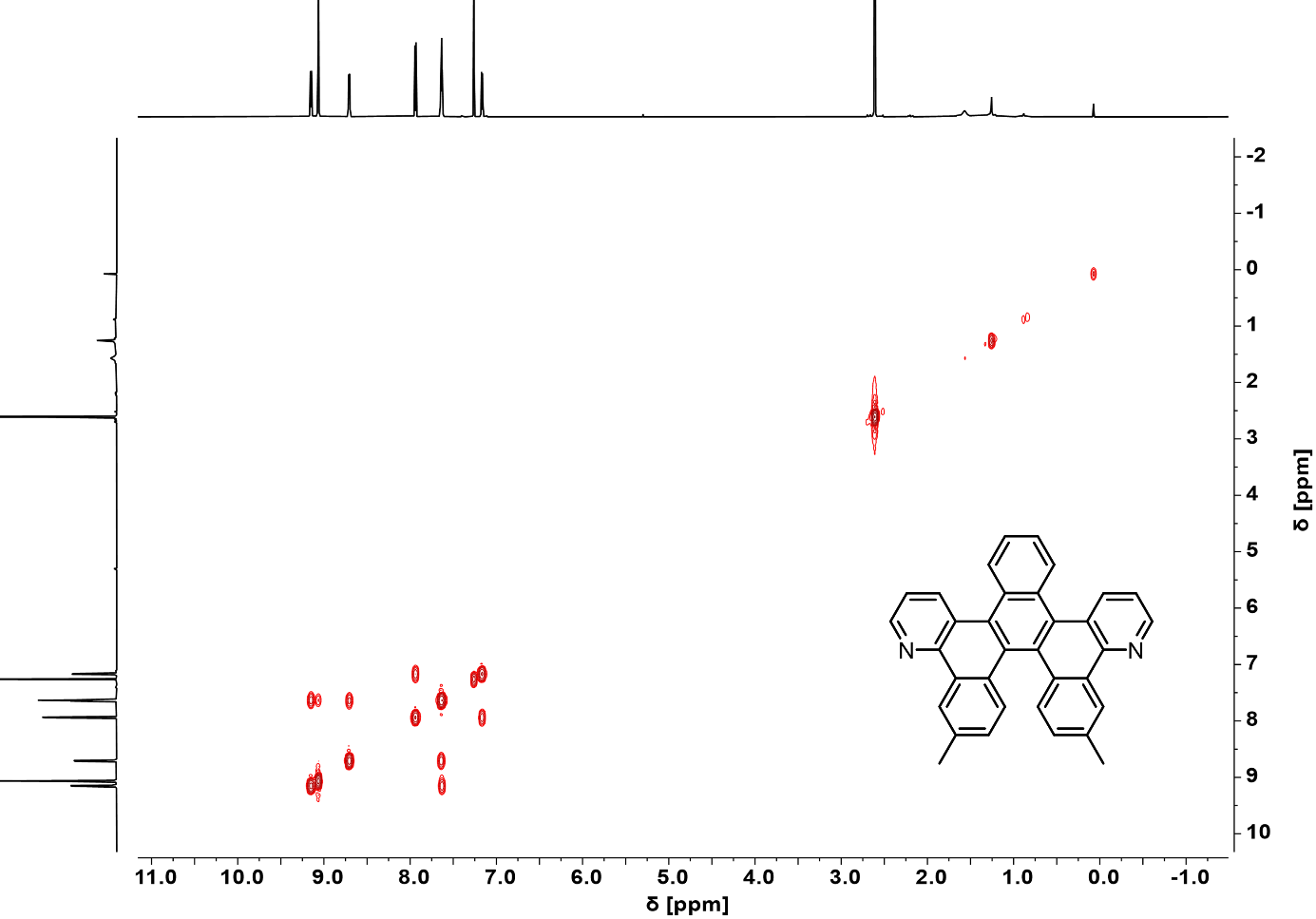


**Figure S46** ^1^H-^1^H COSY NMR spectrum (700 MHz, 295 K) of pentahelicene **4** in CDCl_3_.


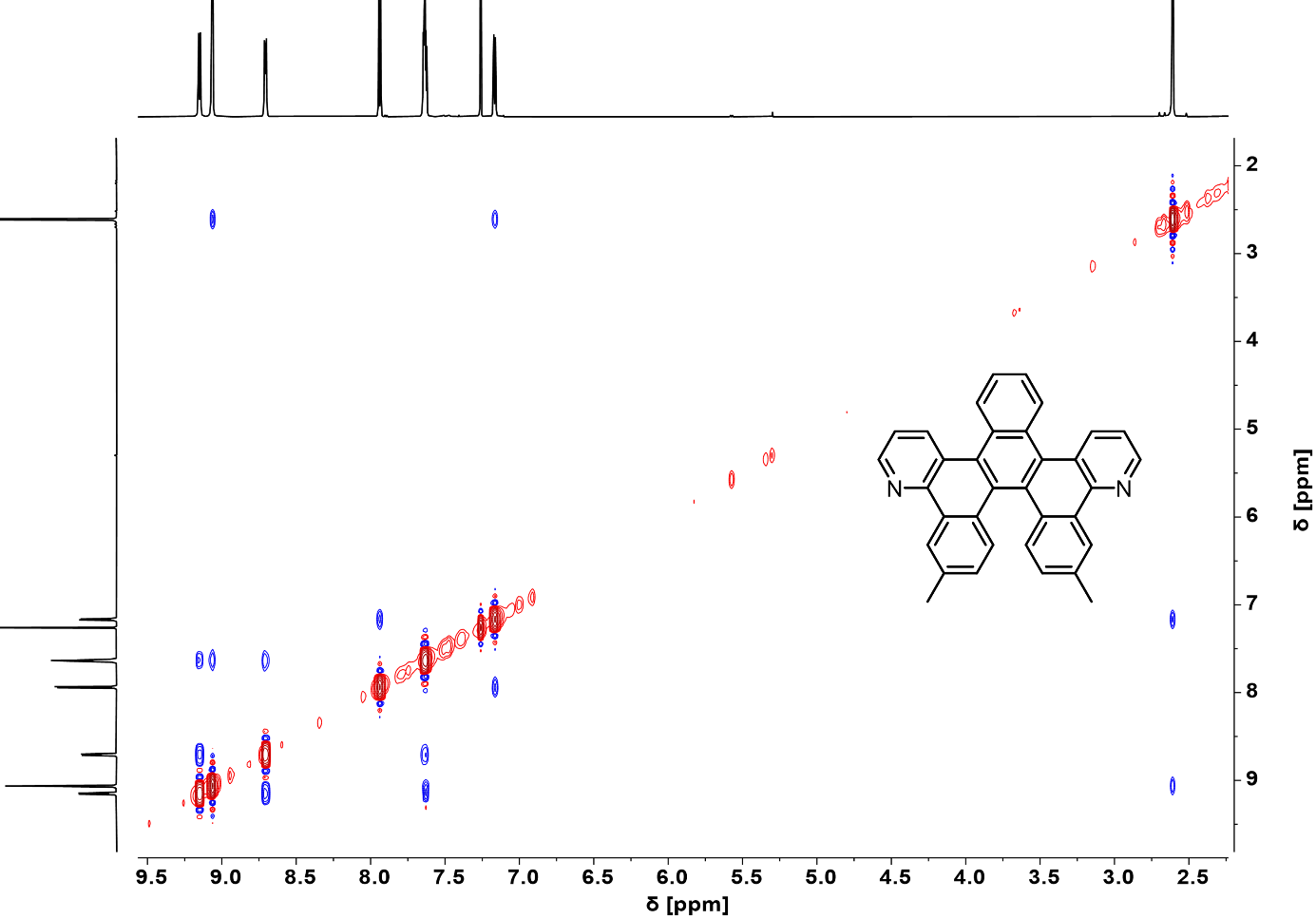


**Figure S47** ^1^H-^1^H NOESY NMR spectrum (700 MHz, 295 K) of pentahelicene **4** in CDCl_3_.

## HR-MS Spectra


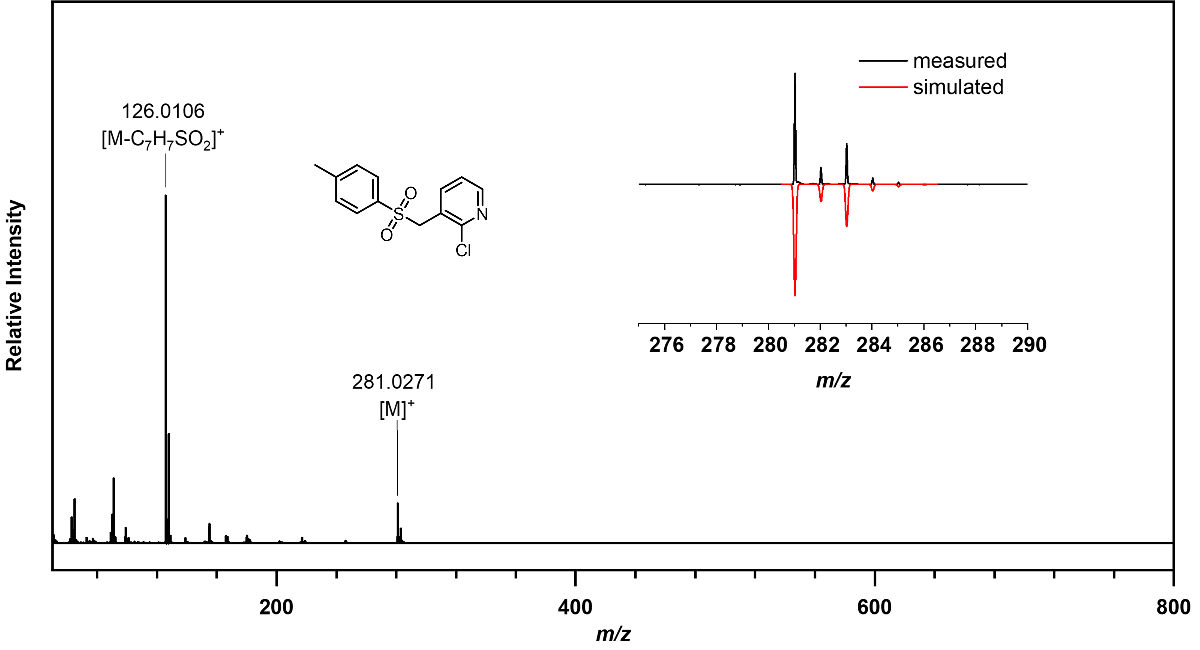


**Figure S48**. HR-MS (EI) of 2-Chloro-3-(tosylmethyl)pyridine (**8**).


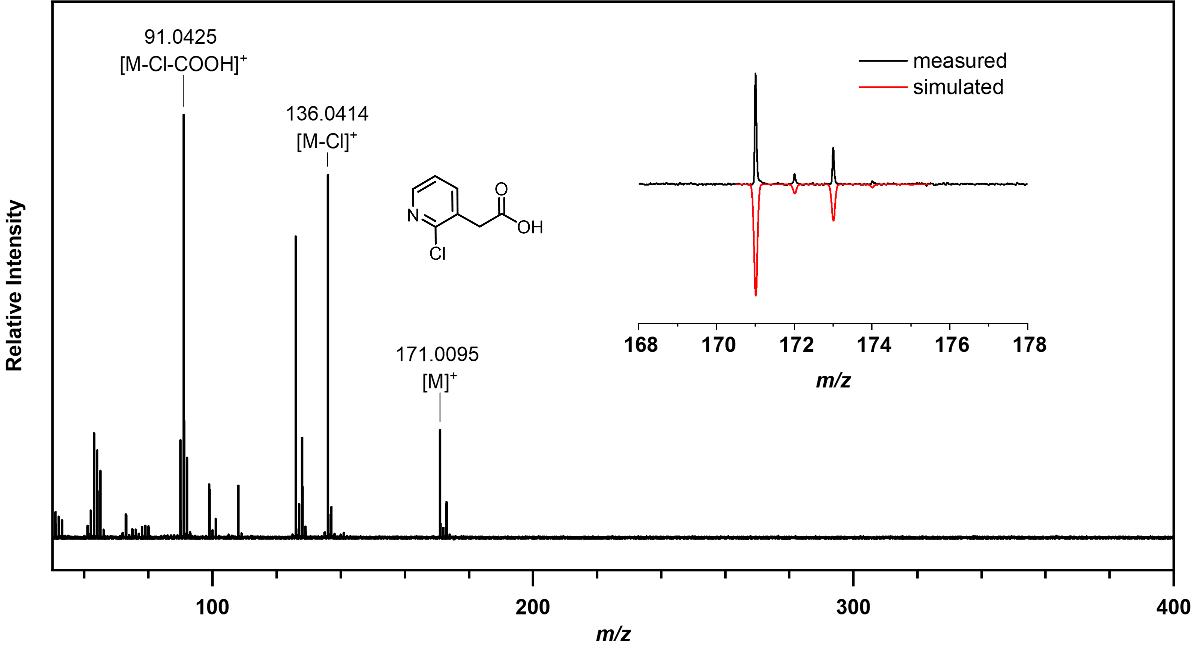


**Figure S49** HR-MS (EI) of 2-(2-chloropyridin-3-yl)acetic acid (**10**).


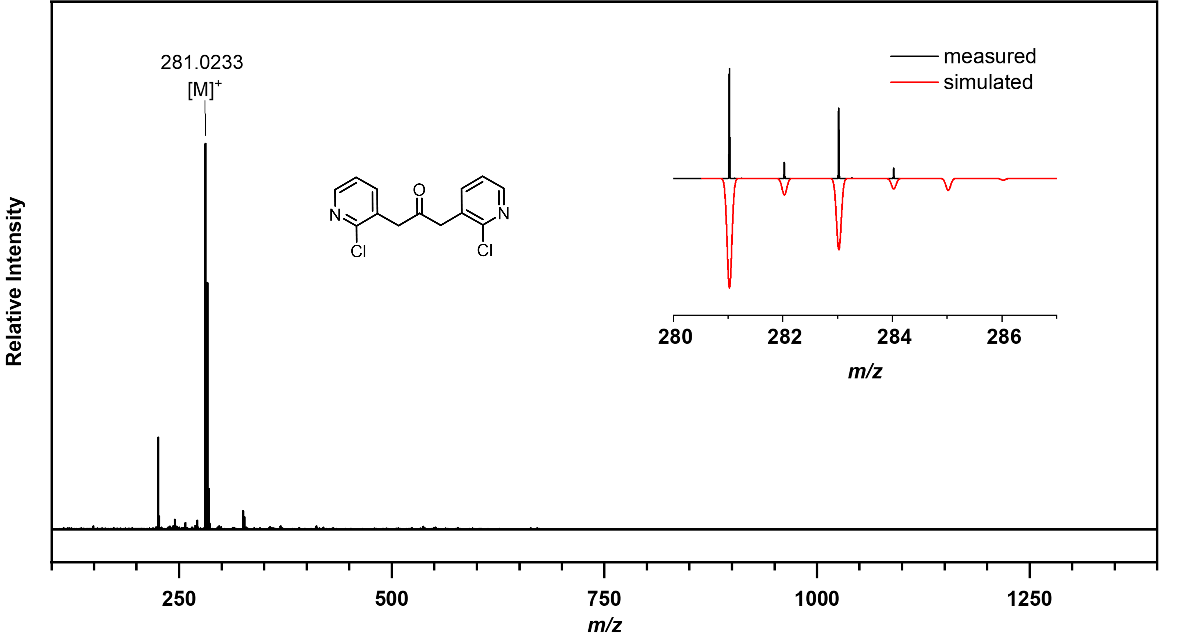


**Figure S50**. HR-MS (APCI) of 1,3-bis(2-chloropyridin-3-yl)propan-2-one (**7**).


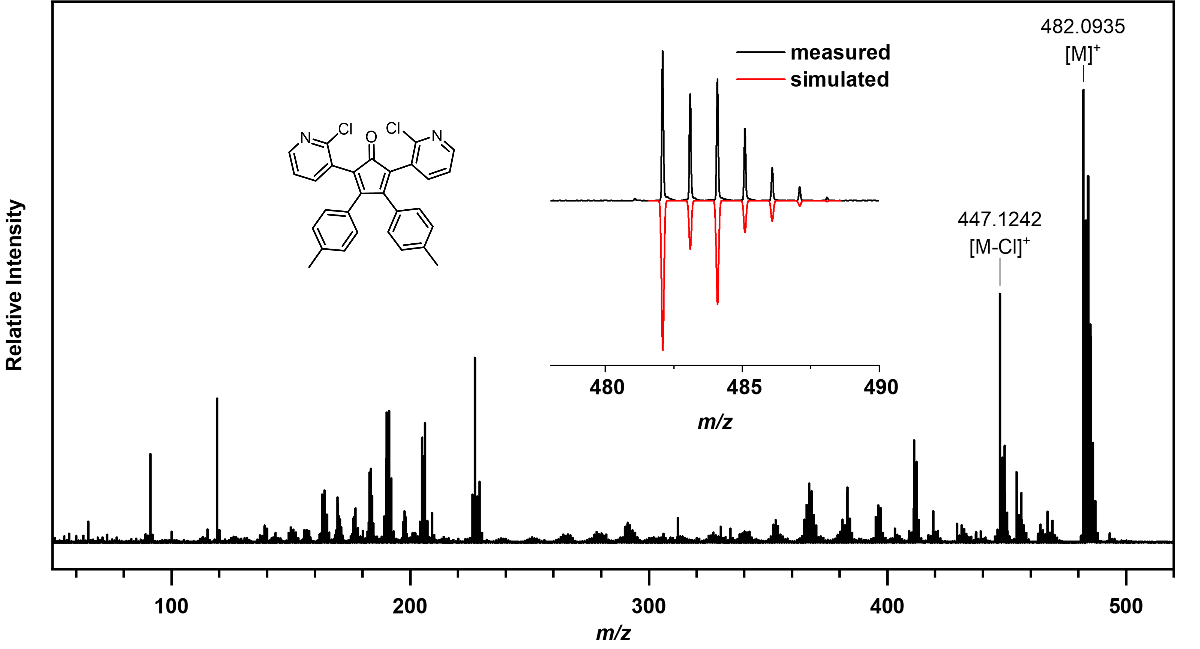


**Figure S51** HR-MS (EI) of 1,3-bis(2-chloropyridin-3-yl)propan-2-one (**12**).


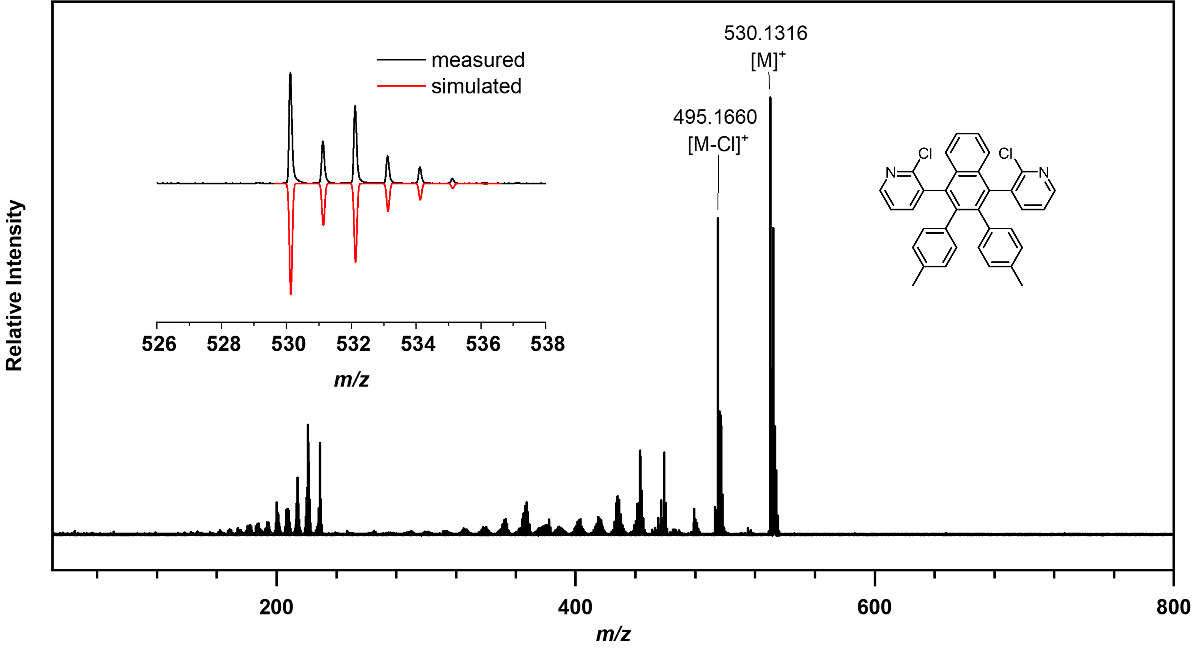


**Figure S52** HR-MS (EI) of chloropyridine **1**.


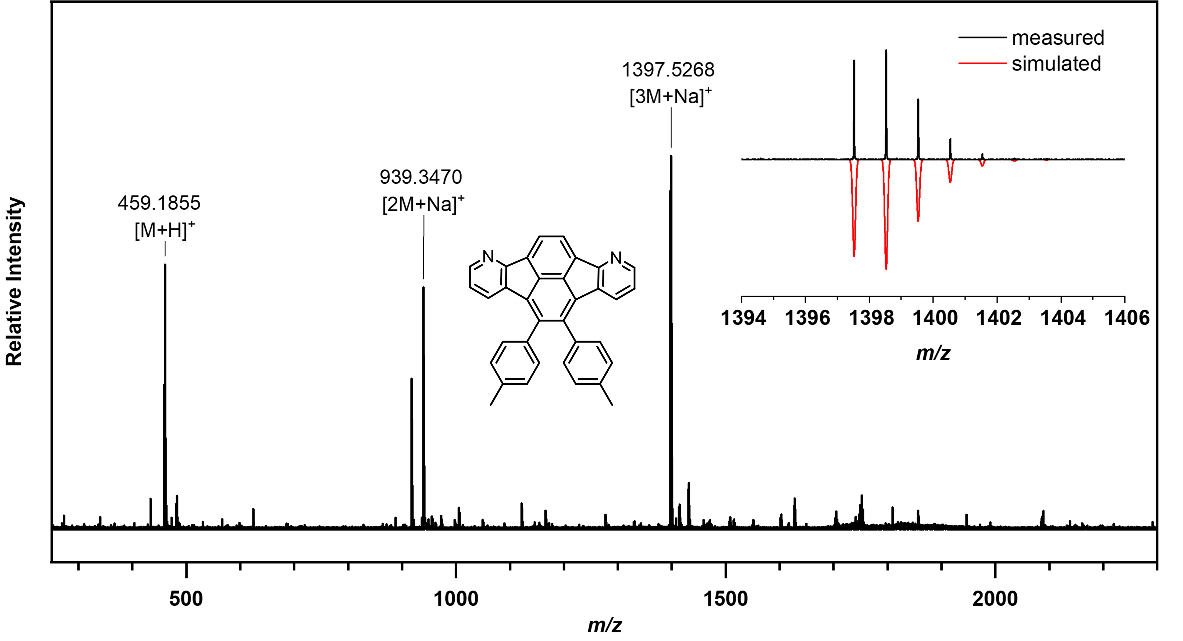


**Figure S53**. HR-MS (ESI, pos. mode) of fluoranthene **2**.


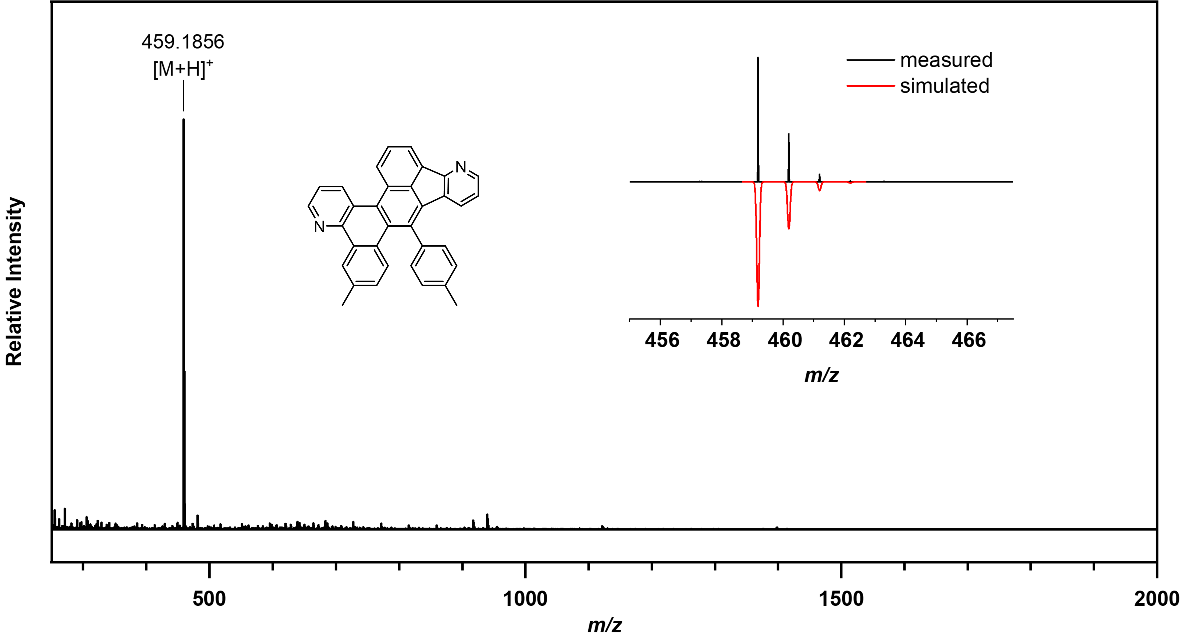


**Figure S54**. HR-MS (ESI, pos. mode) of compound **3**.


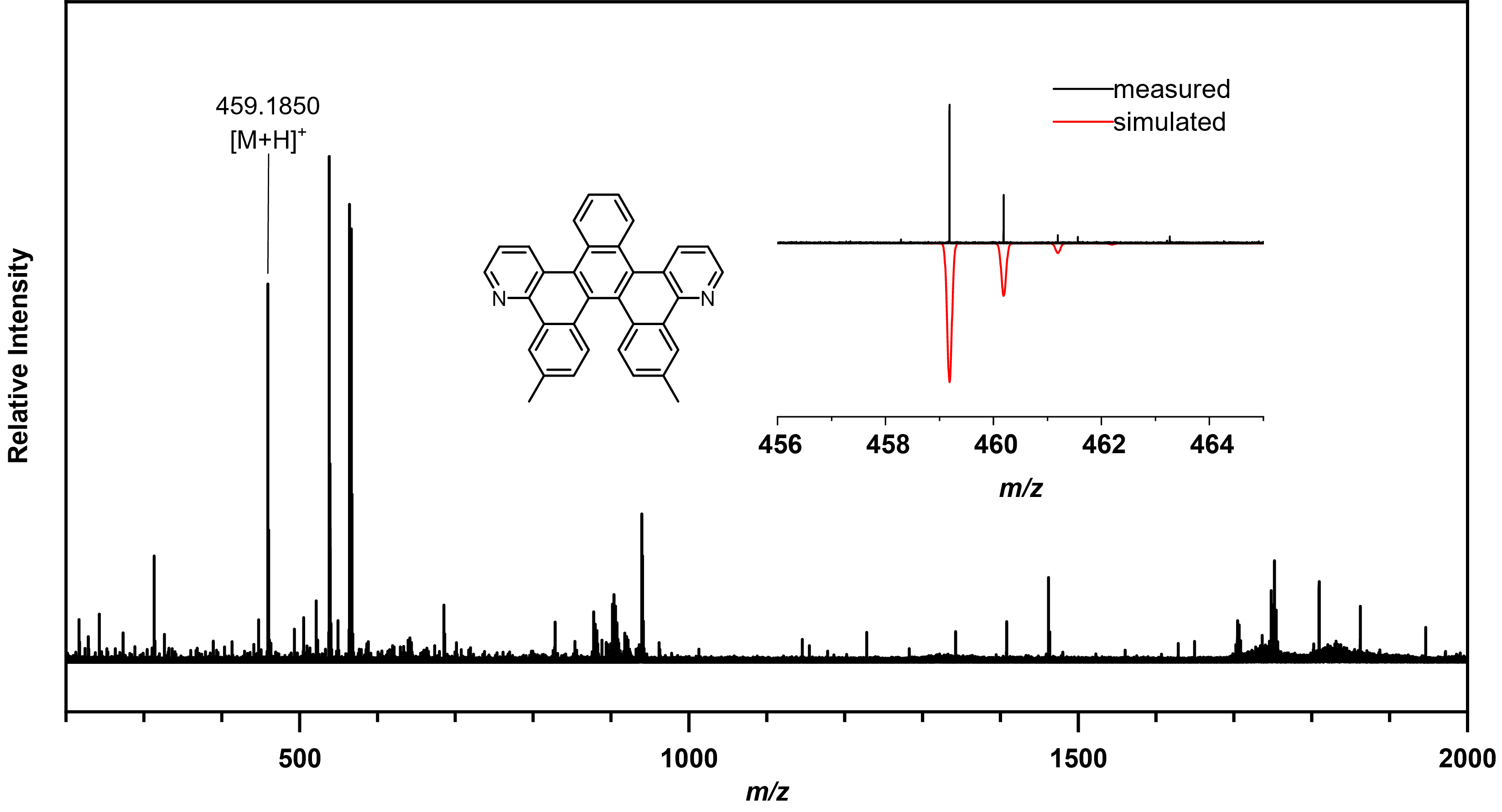


**Figure S55** HR-MS (ESI, pos. mode) of pentahelicene **4**.

## IR Spectra


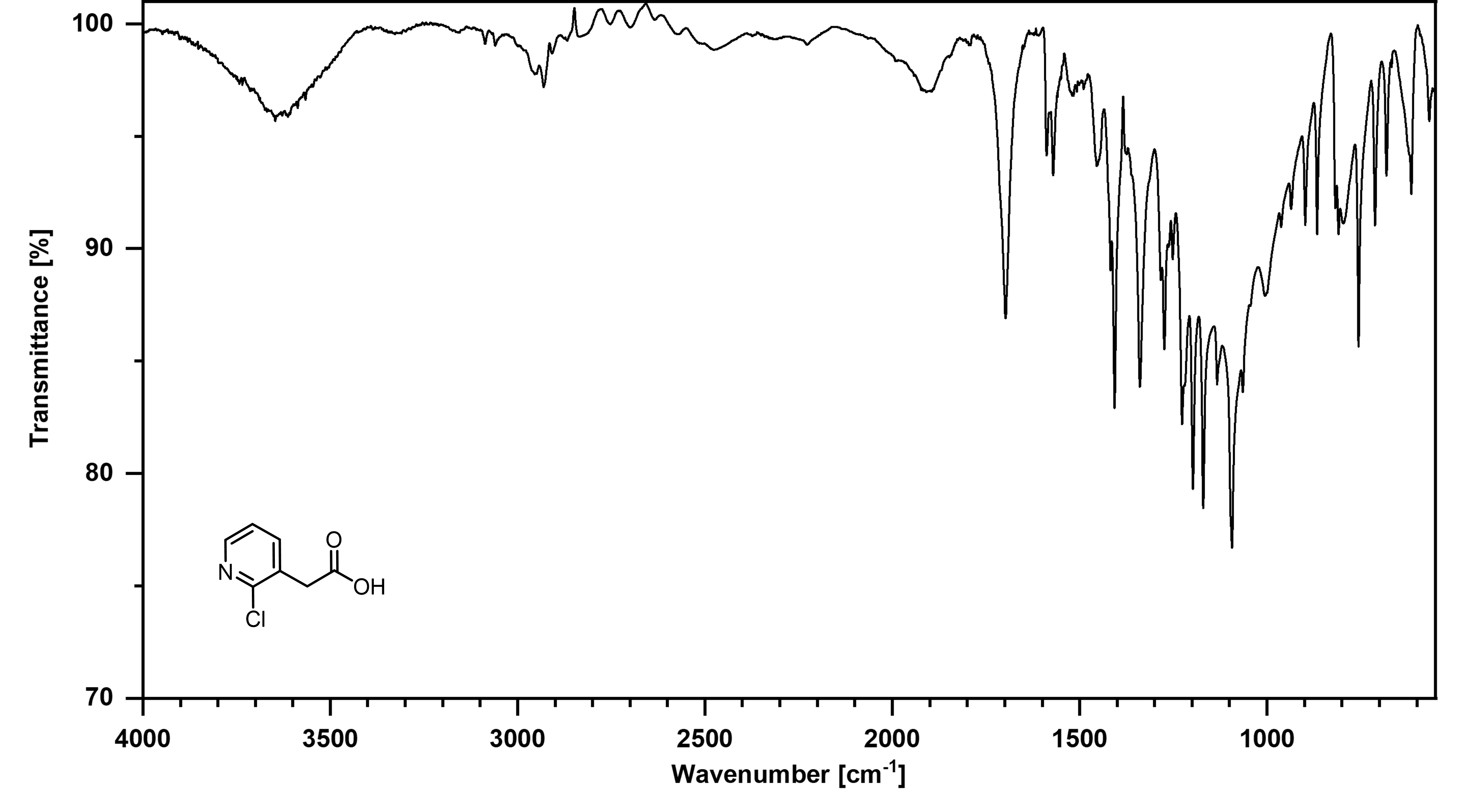


**Figure S56**. IR spectrum (KBr disc) of 2-(2-chloropyridin-3-yl)acetic acid (**10**).


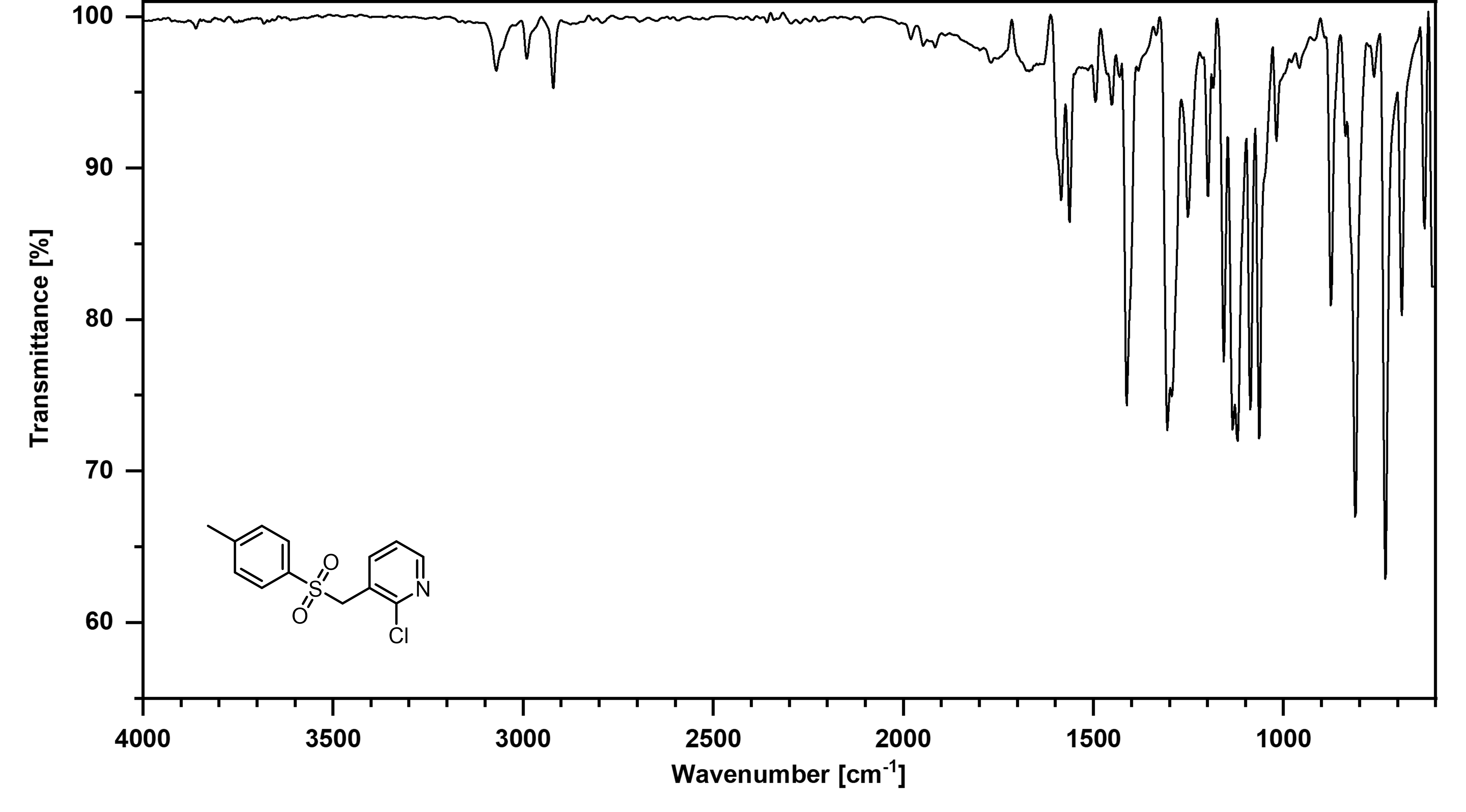


**Figure S57**. IR spectrum (ATR) of 2-chloro-3-(tosylmethyl)pyridine (**8**).


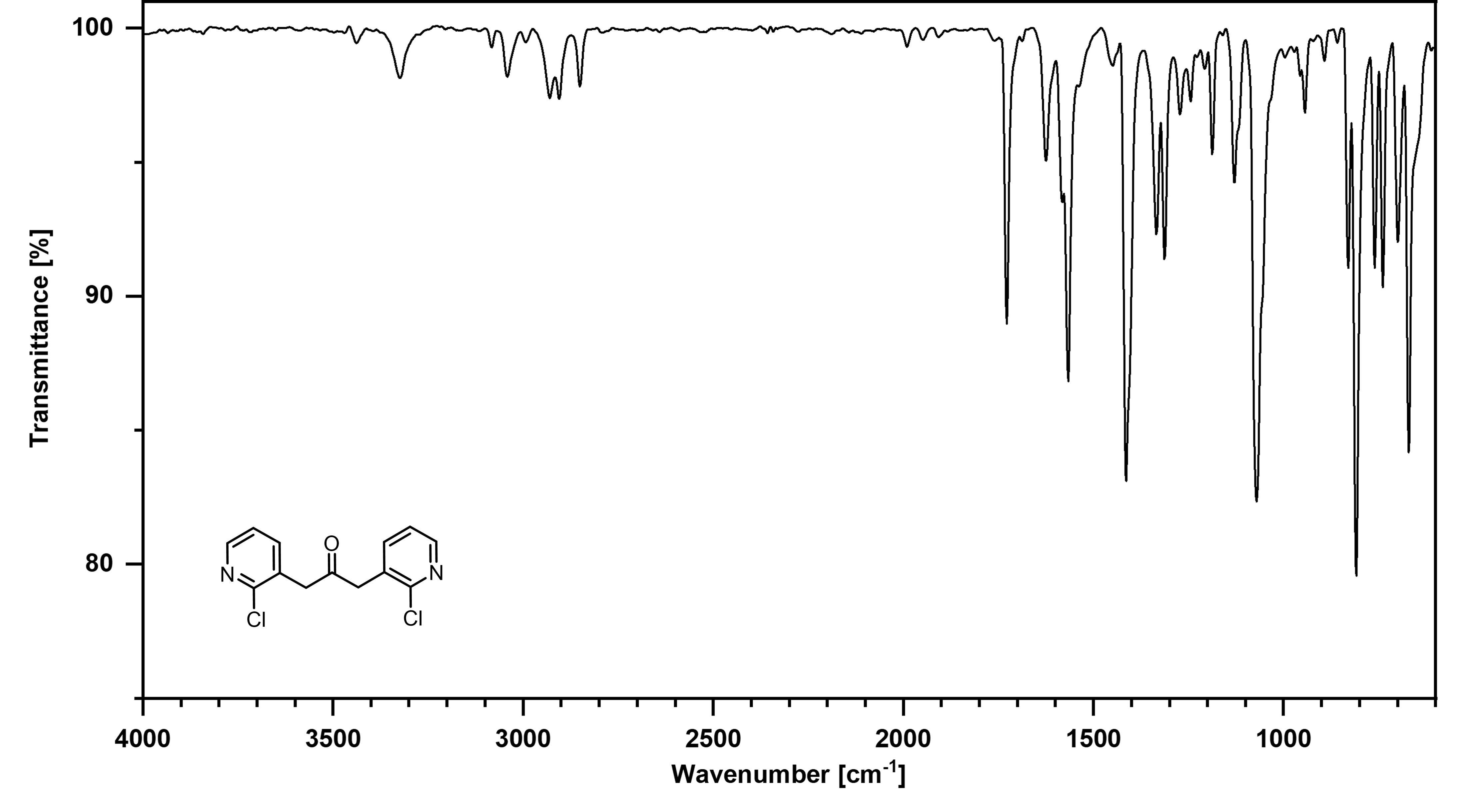


**Figure S58**. IR spectrum (ATR) of 1,3-Bis(2-chloropyridin-3-yl)propan-2-one (**7**).


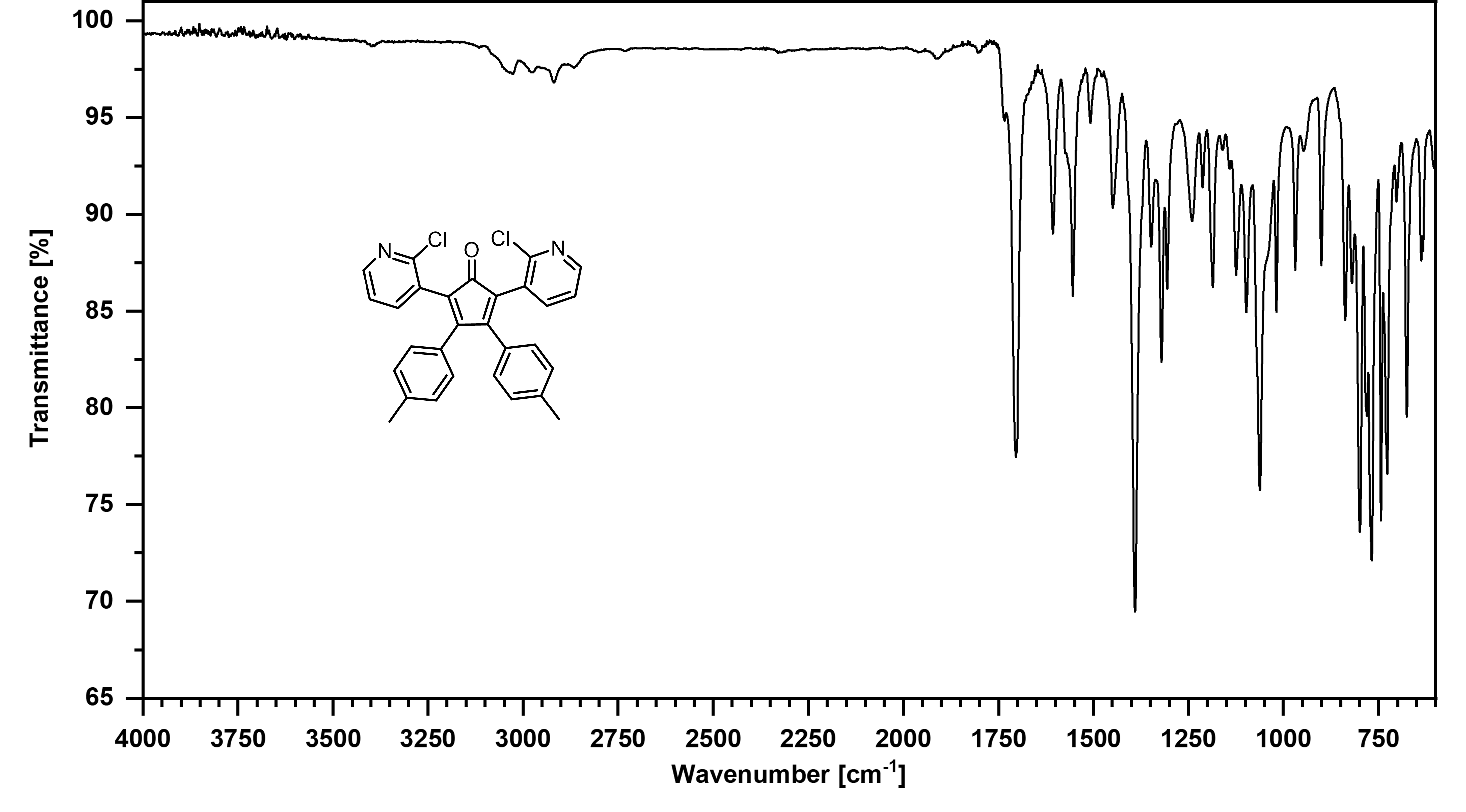


**Figure S59** IR spectrum (ATR) of cyclopentadienone **12**.


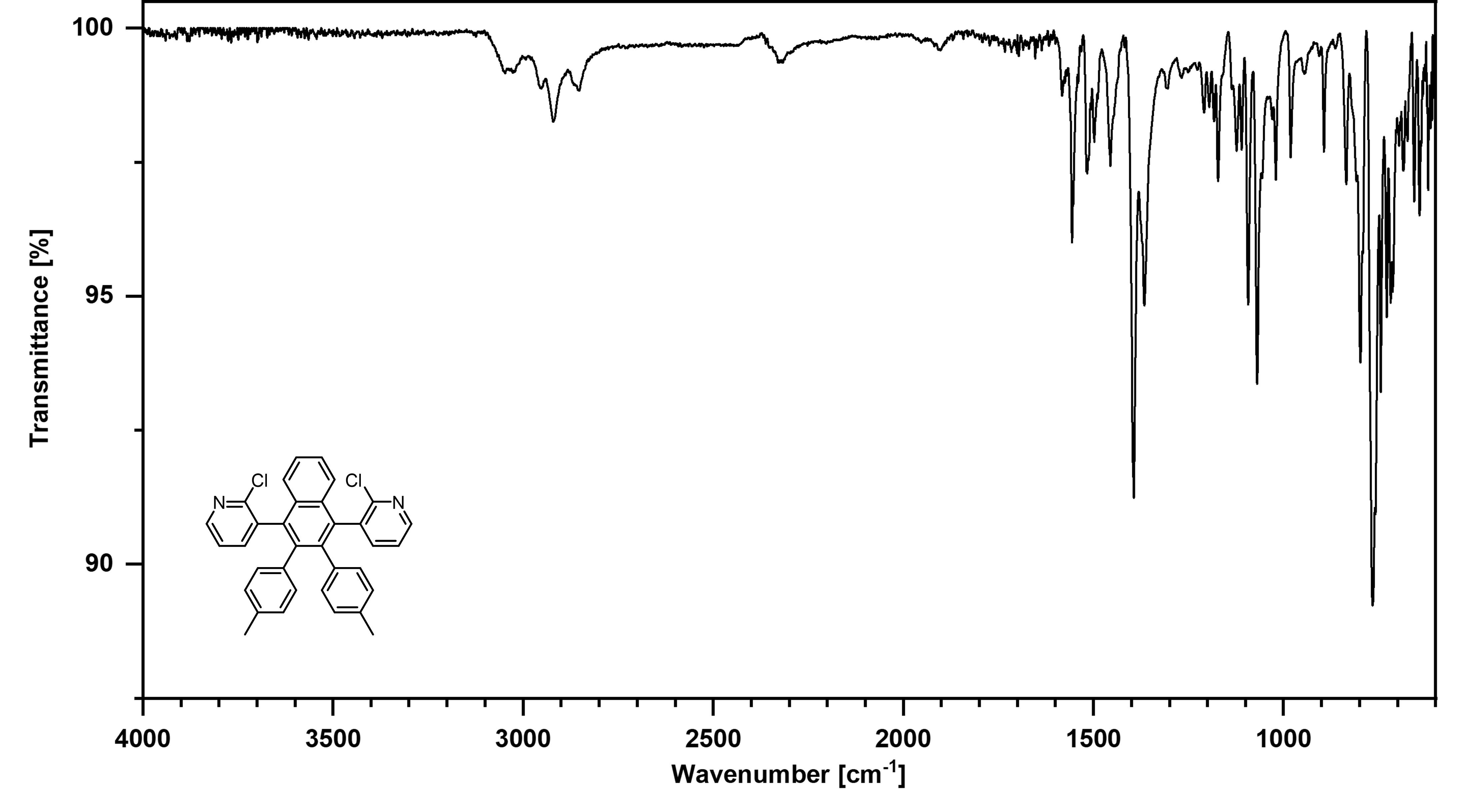


**Figure S60**. IR spectrum (ATR) of chloropyridine **1**.


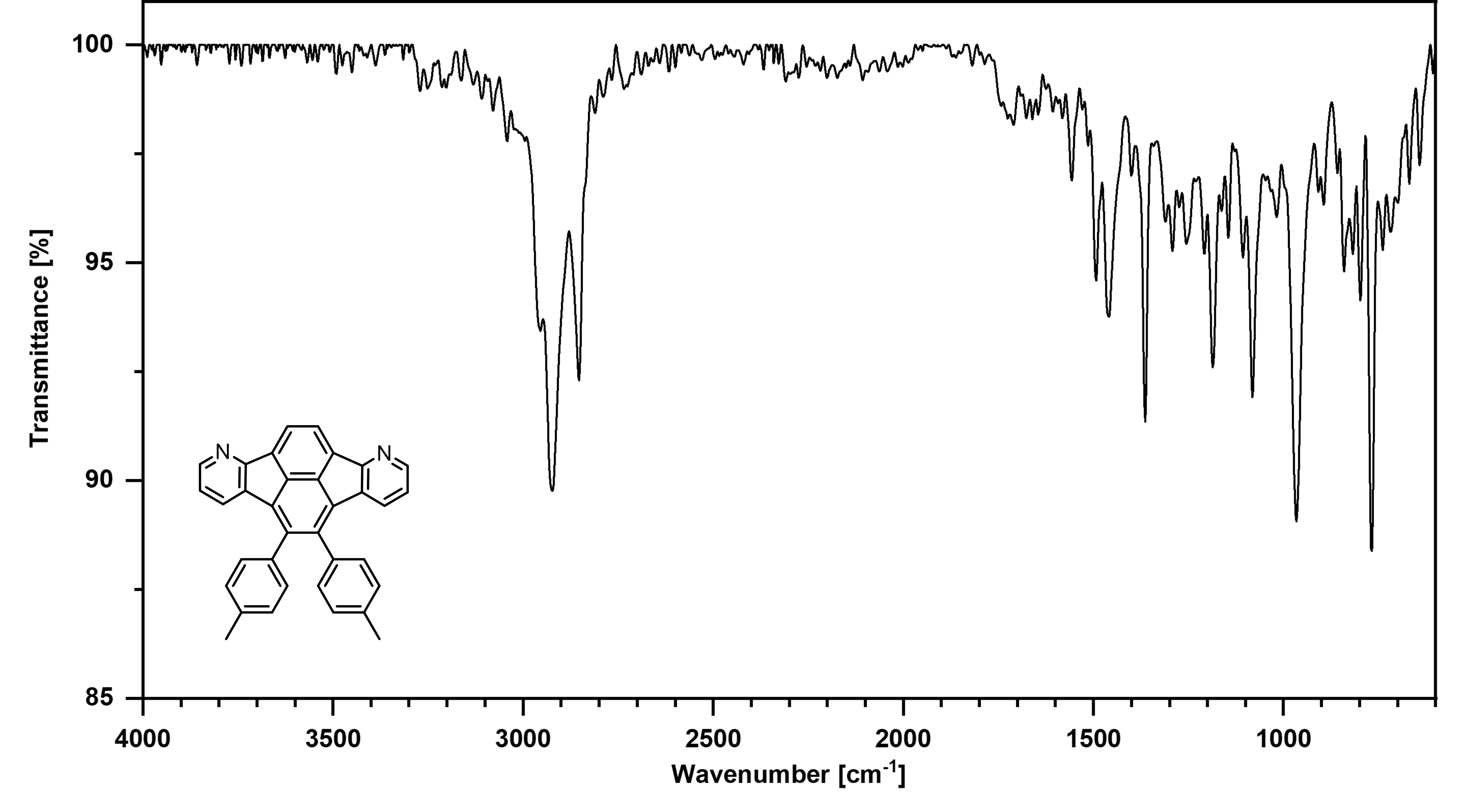


**Figure S61** IR spectrum (ATR) of fluoranthene **2**.


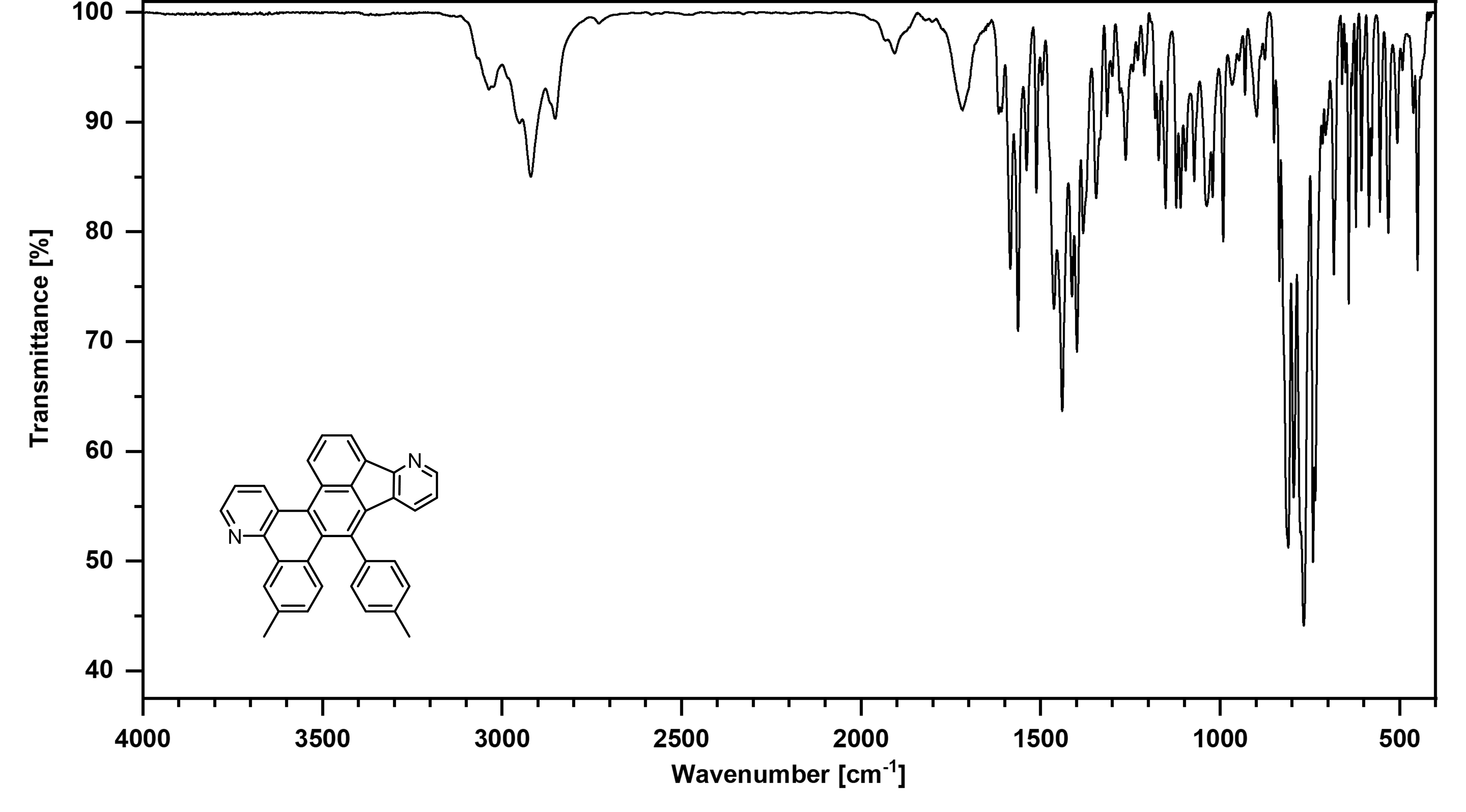


**Figure S62** IR spectrum (KBr disc) of compound **3**.


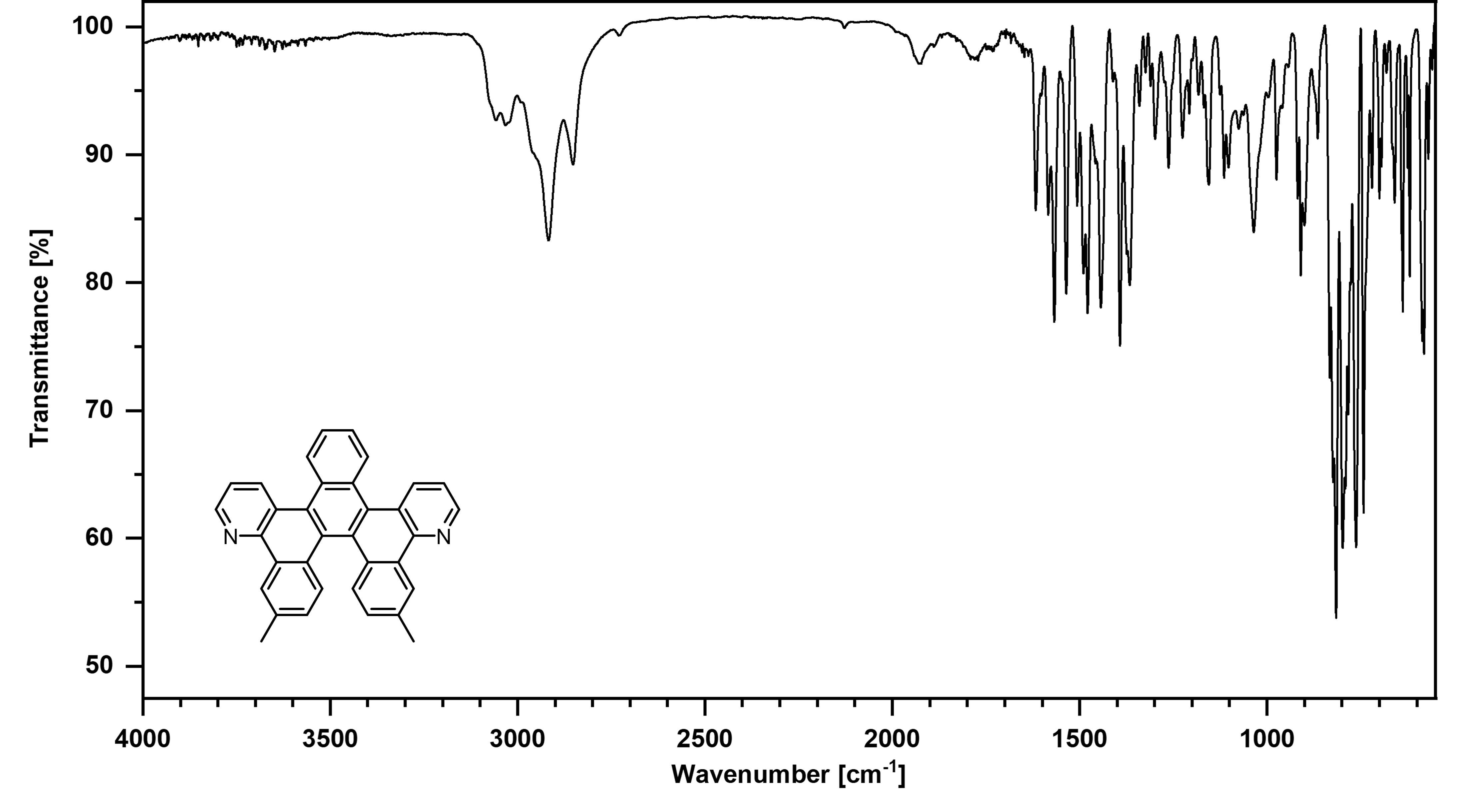


**Figure S63** IR spectrum (KBr disc) of pentahelicene **4**.

## UV/Vis Spectra


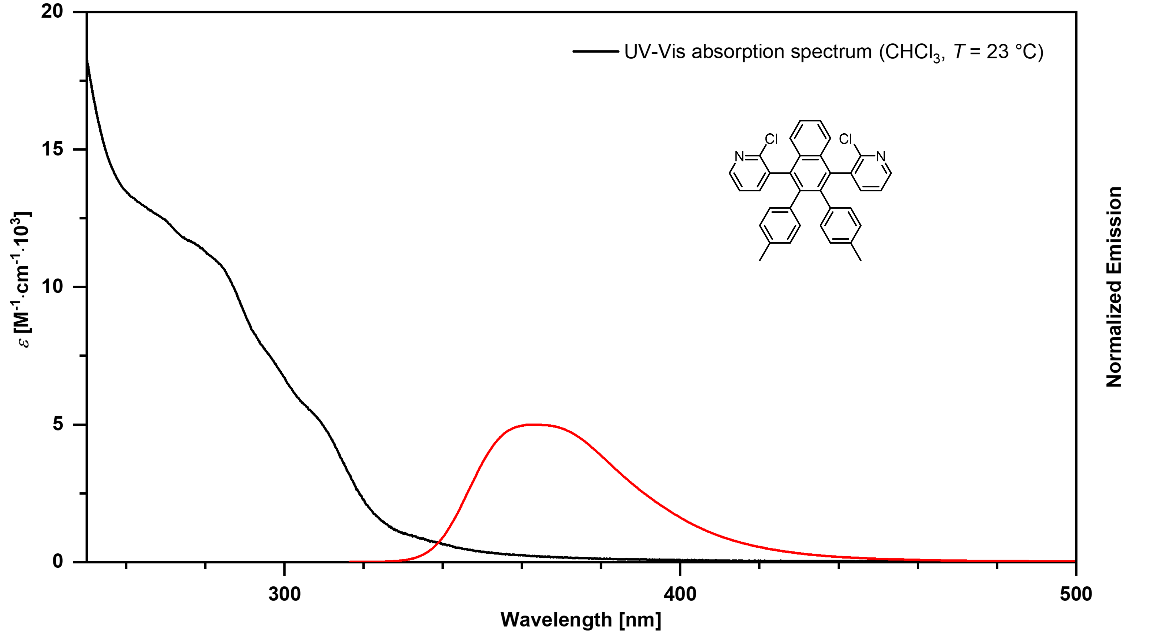


**Figure S64**. UV/Vis absorption (black) and fluorescence spectra (red, λ_exc_ = 303 nm) of chloropyridine **1**.


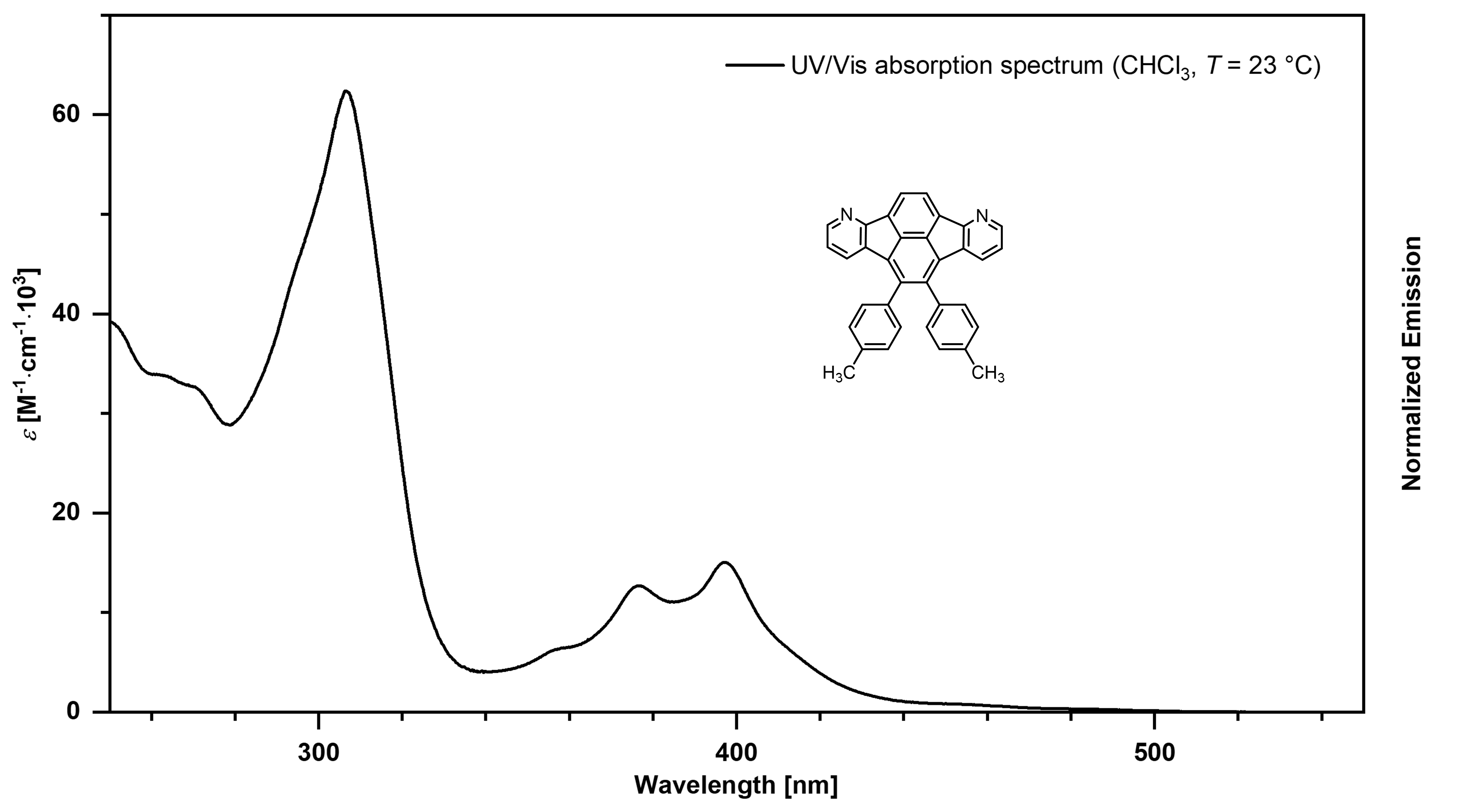


**Figure S65**. UV/Vis absorption spectrum of fluoranthene **2** (CHCl_3_, 296 K). No fluorescence was detected.


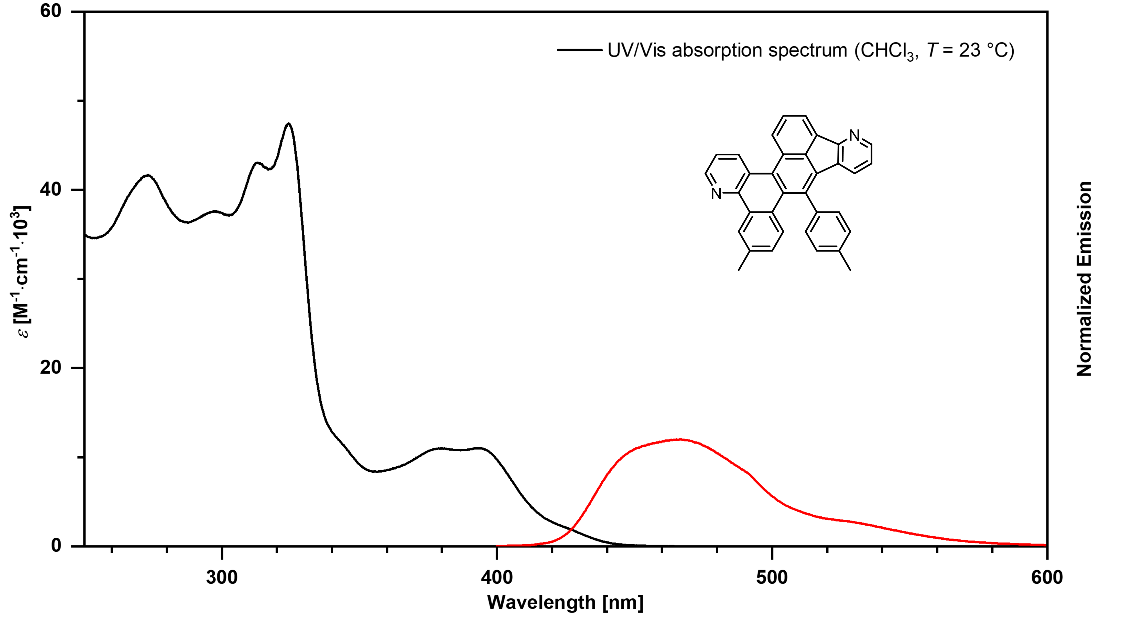


**Figure S66**. UV/Vis absorption (black) and fluorescence spectra (red, λ_exc_ = 345 nm) of **3** (CHCl_3_, 296 K, Φ_fl_= 13%).


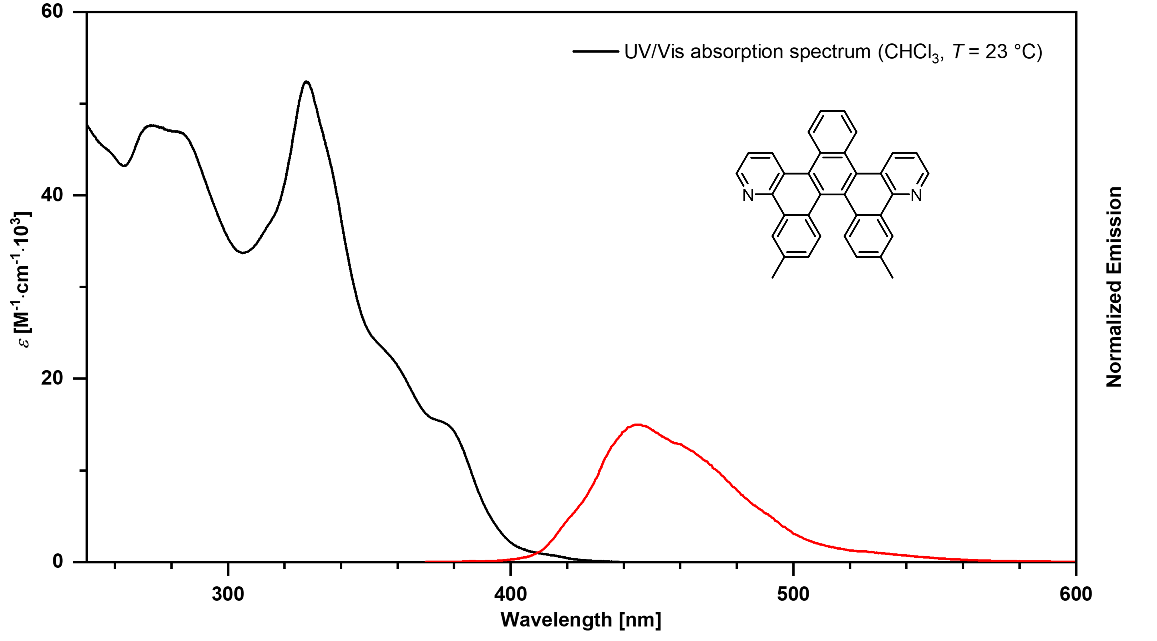


**Figure S67**. UV/Vis absorption (black) and fluorescence (red, λ_exc_ = 346 nm) spectra of helicene **4** (CHCl_3_, 296 K, Φ_fl_= 6%).

## UV/Vis Spectra of Fluoranthene 2 in Different Solvents


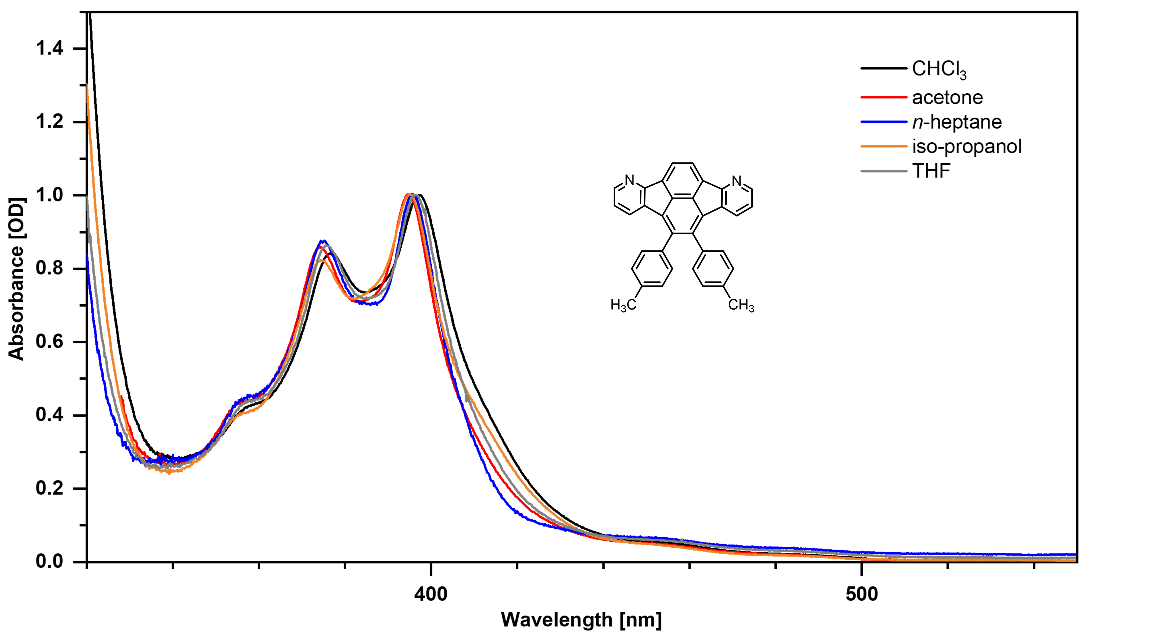


**Figure S68** UV/Vis absorption spectra of fluoranthene **2** recorded in solvents of different polarity.

## CV and DPV


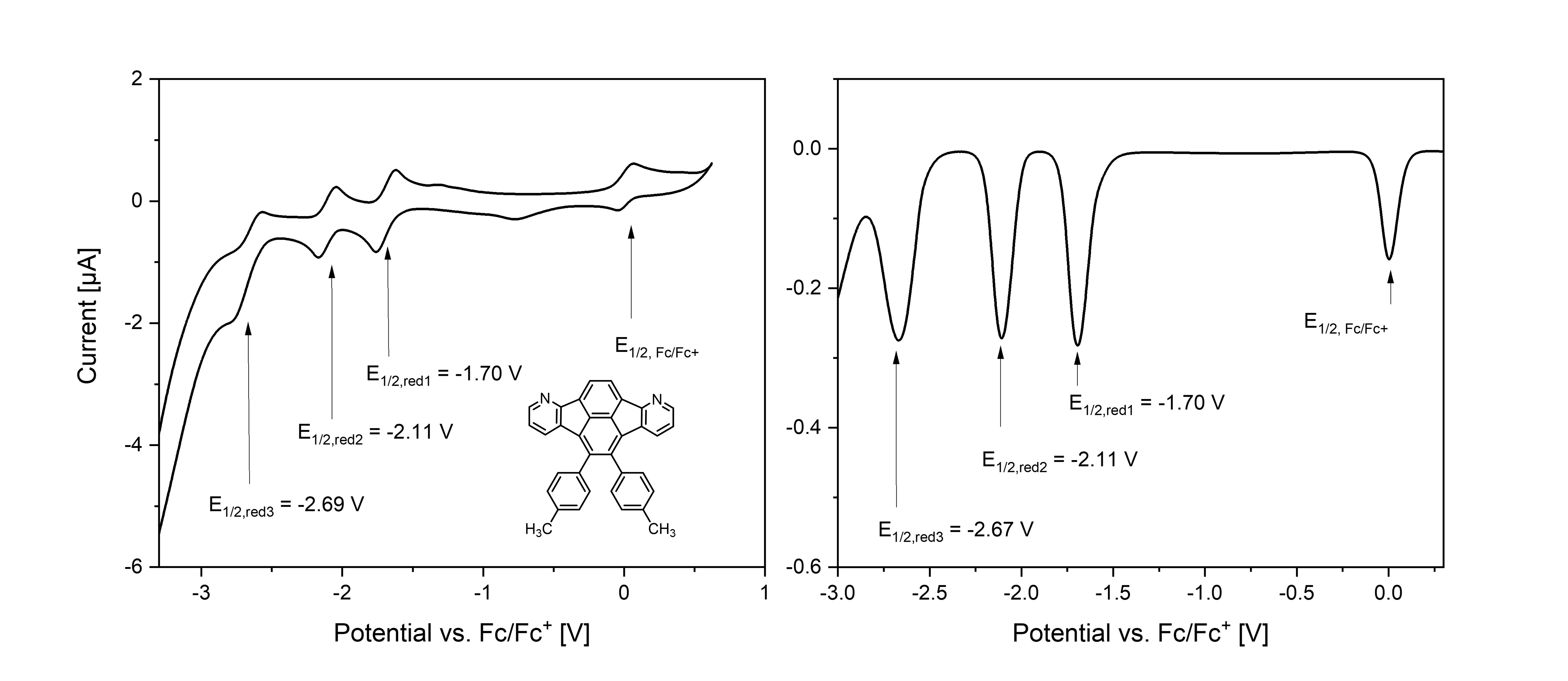


**Figure S69**. CV (left) and DPV (right) spectra of fluoranthene **2** in a 0.05 m NBu_4_PF_6_ solution in THF.


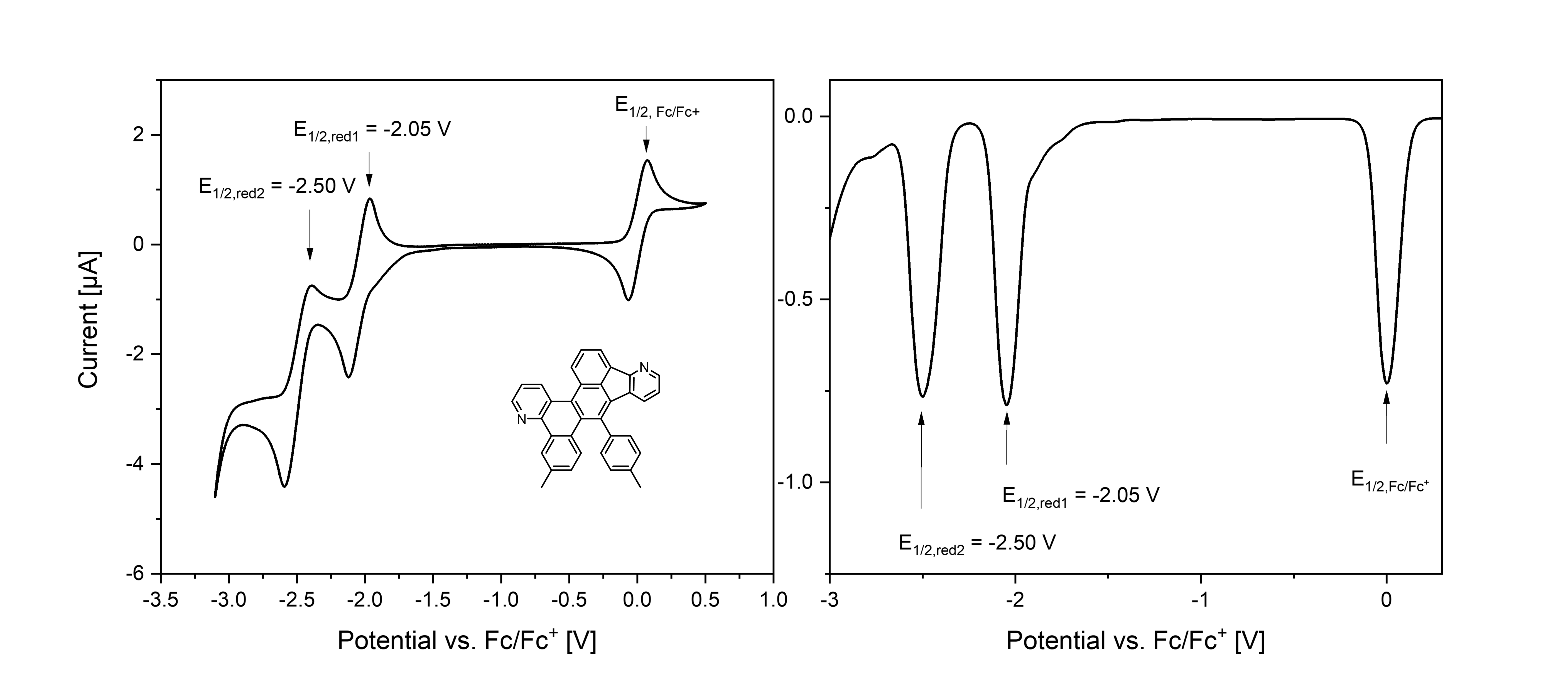
**Figure S70**. CV (left) and DPV (right) spectra of compound **3** in a 0.05 m NBu_4_PF_6_ solution in THF.


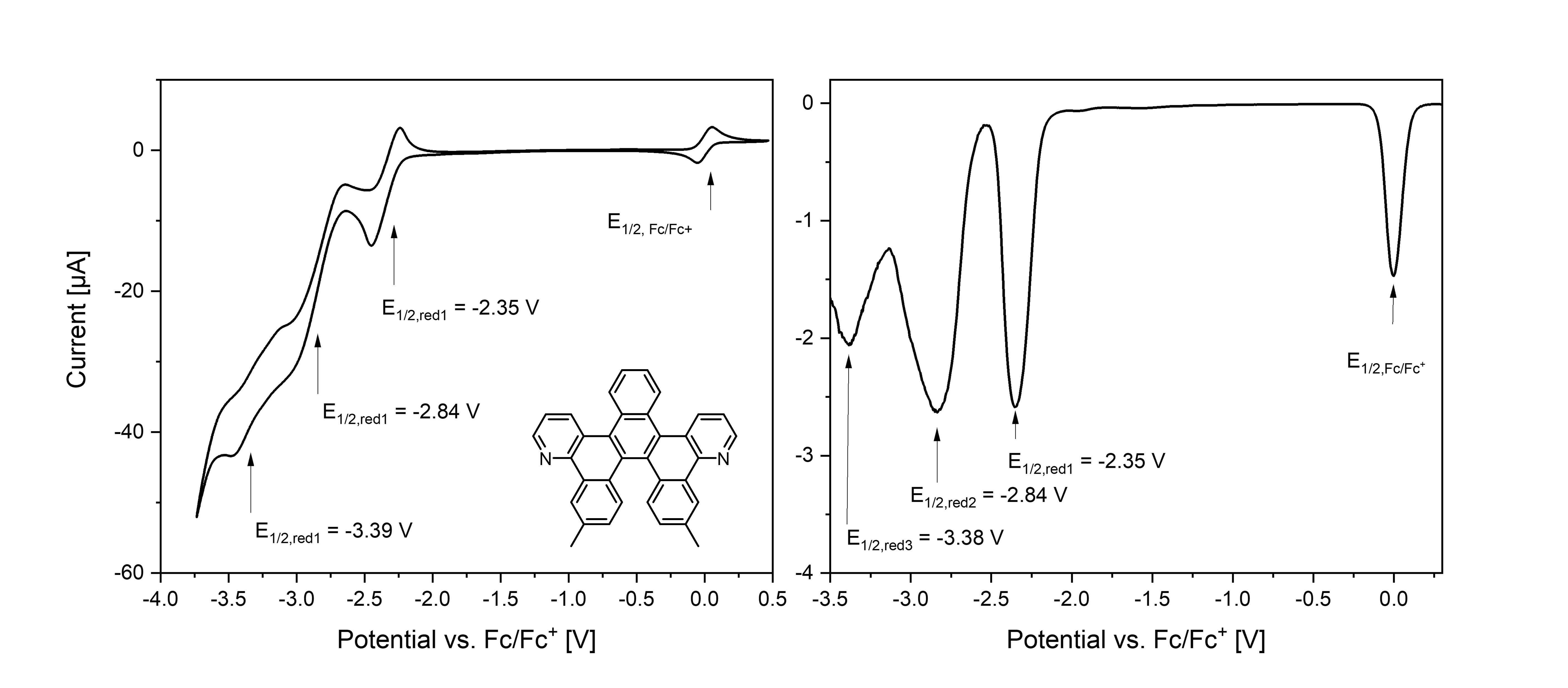


**Figure S71**. CV (left) and DPV (right) spectra of pentahelicene **4** in a 0.05 m NBu_4_PF_6_ solution in THF.

## Crystal Structure Data

Crystal structure data of 2-chloro-3-(tosylmethyl)pyridine

Crystallized by slow diffusion of *n*-hexane into an ethyl acetate solution.


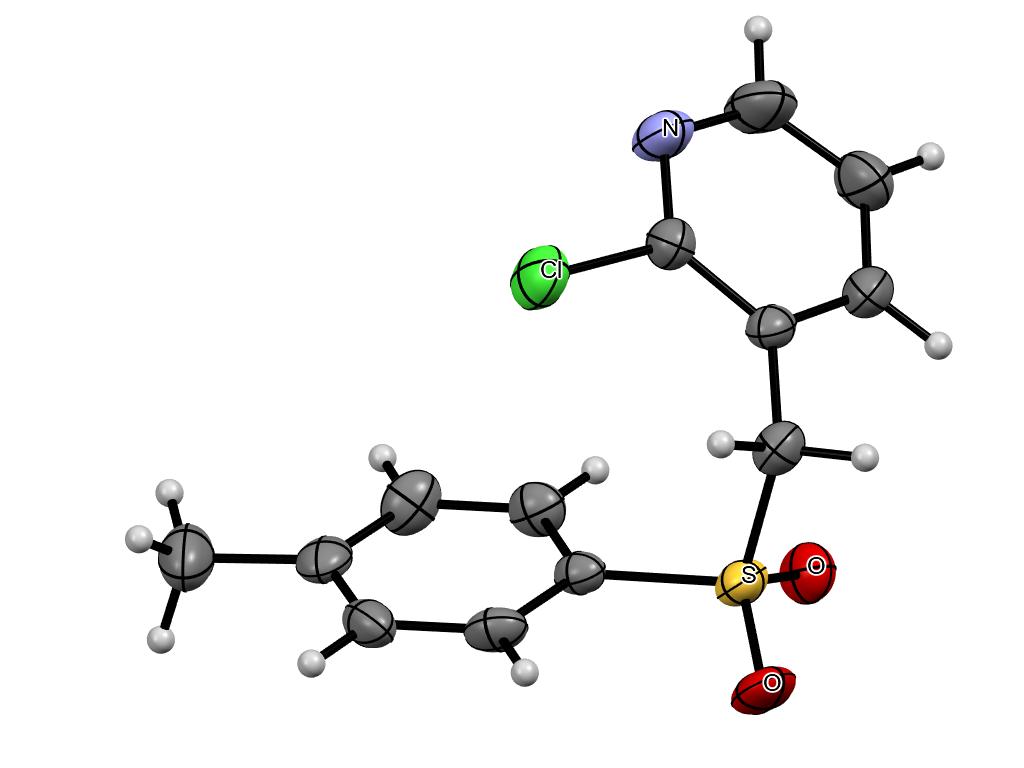


**Figure S72**. ORTEP representation of 2-chloro-3-(tosylmethyl)pyridine (**8**). Ellipsoid probability level: 50%.

**Table S2**. Crystal data and structure refinement for **8**.

CCDC-number 2486398

Empirical formula C_13_H_12_ClNO_2_S

Formula weight 281.75

Temperature 200(2) K

Wavelength 0.71073 Å

Crystal system monoclinic

Space group *P*21/c

Z 8

Unit cell dimensions *a* = 24.865(3) Å *α* = 90 deg.

*b* = 5.3408(6) Å *β* = 113.887(3) deg.

*c* = 21.503(3) Å *γ* = 90 deg.

Volume 2611.1(5) Å^3^

Density (calculated) 1.43 g/cm^3^

Absorption coefficient 0.44 mm^-1^

Crystal shape column

Crystal size 0.113 x 0.048 x 0.045 mm^3^

Crystal colour colourless

Theta range for data collection 0.9 to 26.3 deg.

Index ranges -30≤*h*≤31, -6≤*k*≤6, -26≤*l*≤26

Reflections collected 25705

Independent reflections 5307 (*R(int)* = 0.0958)

Observed reflections 3005 (*I* > 2*σ* (*I*))

Absorption correction Semi-empirical from equivalents

Max. and min. transmission 0.96 and 0.91

Refinement method Full-matrix least-squares on F^2^

Data/restraints/parameters 5307 / 0 / 327

Goodness-of-fit on F2 1.04

Final R indices (I>2sigma(I)) *R*1 = 0.058, *wR*2 = 0.105

Largest diff. peak and hole 0.31 and -0.32 eÅ^-3^

Crystal structure data of chloropyridine **1**.

Crystallized by slow diffusion of *n*-hexane into an ethyl acetate solution.


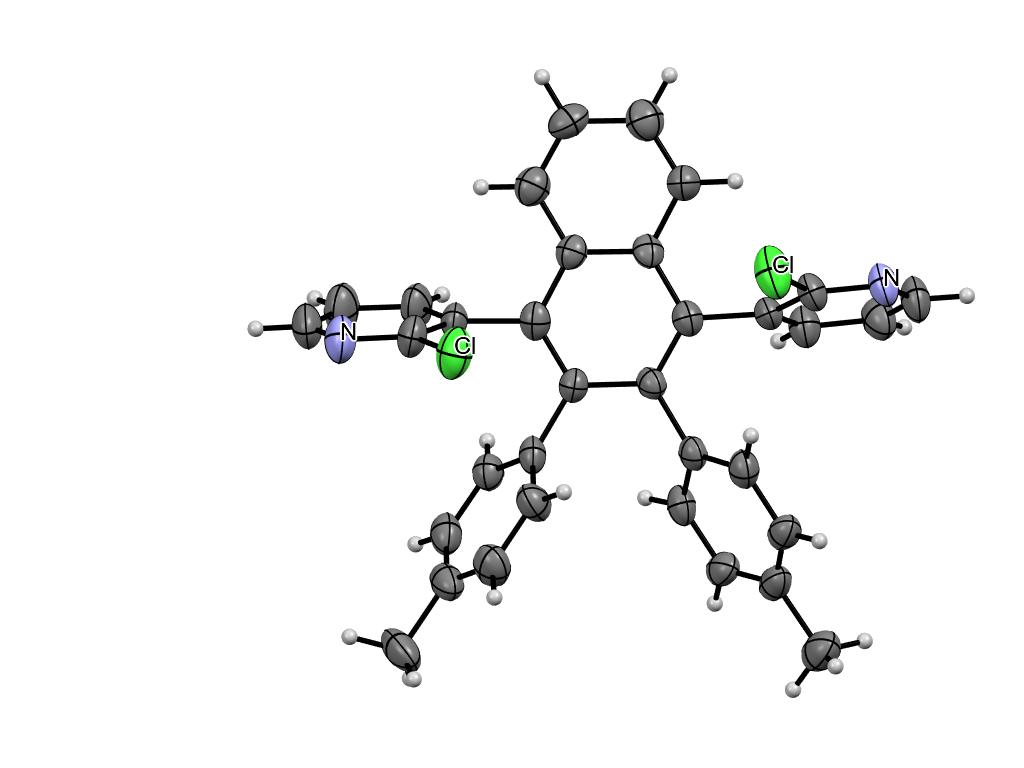


**Figure S73**. ORTEP representation of chloropyridine **1**. Ellipsoid probability level: 50%.

**Table S3**. Data and structure refinement for chloropyridine **1**.

CCDC-number 2486399

Empirical formula C_34_H_24_Cl_2_N_2_

Formula weight 531.45

Temperature 200(2) K

Wavelength 1.54178 Å

Crystal system monoclinic

Space group *P*2_1_/c

Z 4

Unit cell dimensions *a* = 12.3695(5) Å *α* = 90 deg.

*b* = 11.2492(3) Å *β* = 94.066(3) deg.

*c* = 19.6897(8) Å *γ* = 90 deg.

Volume 2732.87(17) Å^3^

Density (calculated) 1.29 g/cm^3^

Absorption coefficient 2.33 mm^-1^

Crystal shape plate

Crystal size 0.057 x 0.030 x 0.014 mm^3^

Crystal colour colourless

Theta range for data collection 3.6 to 61.6 deg.

Index ranges -14≤*h*≤12, -10≤*k*≤12, -16≤*l*≤22

Reflections collected 16730

Independent reflections 4161 (*R(int)* = 0.0846)

Observed reflections 2409 (*I* > 2*σ*(*I*))

Absorption correction Semi-empirical from equivalents

Max. and min. transmission 0.95 and 0.89

Refinement method Full-matrix least-squares on F^2^

Data/restraints/parameters 4161 / 0 / 345

Goodness-of-fit on F^2^ 1.17

Final R indices (I>2sigma(I)) *R*1 = 0.084, *wR*2 = 0.153

Largest diff. peak and hole 0.28 and -0.29 eÅ^-3^

Crystal structure data of *aza*-indeno-*aza*-fluoranthene (**2**).

Crystallized by slow diffusion of *n*-hexane into a dichloromethane solution.


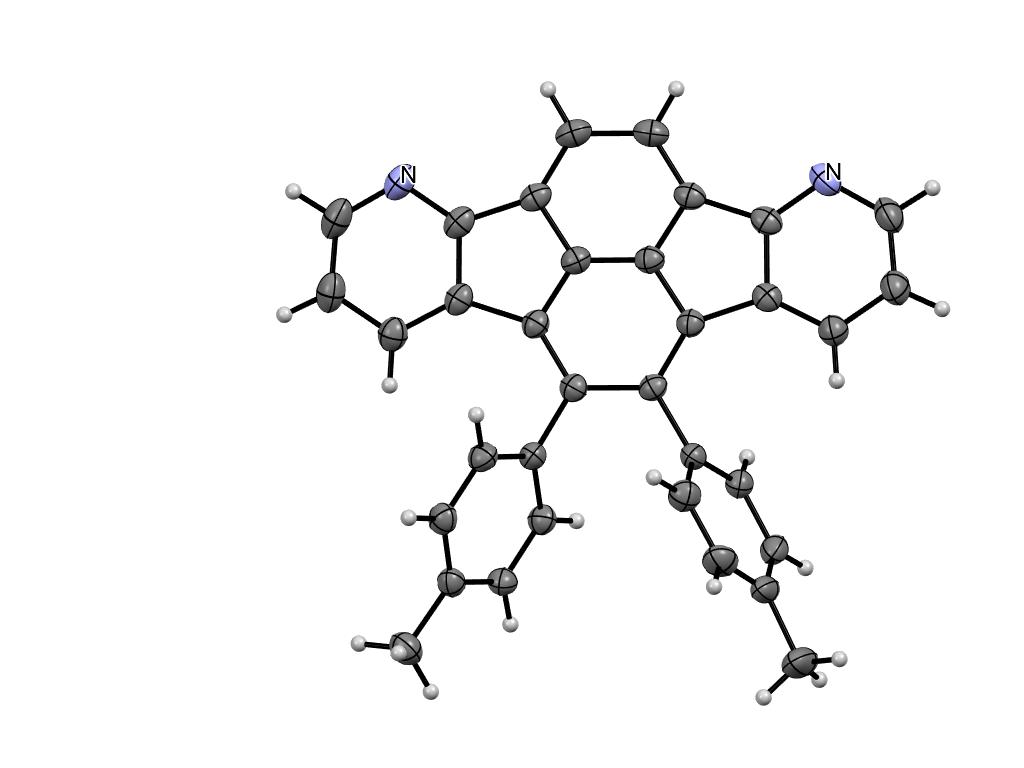


**Figure S74**. ORTEP representation of aza-indeno-aza-fluoranthene (**2**). Ellipsoid probability level: 50%.

**Table S4**. Crystal data and structure refinement for fluoranthene **2**.

CCDC-number 2486400

Empirical formula C_34_H_22_N_2_

Formula weight 458.53

Temperature 200(2) K

Wavelength 0.71073 Å

Crystal system monoclinic

Space group *P*2_1_/c

Z 4

Unit cell dimensions *a* = 8.3401(9) Å *α* = 90 deg.

*b* = 23.432(3) Å *β* = 105.2759(16) deg.

*c* = 12.4217(13) Å *γ* = 90 deg.

Volume 2341.8(4) Å^3^

Density (calculated) 1.30 g/cm^3^

Absorption coefficient 0.08 mm^-1^

Crystal shape column

Crystal size 0.127 x 0.111 x 0.030 mm^3^

Crystal colour orange

Theta range for data collection 2.4 to 29.4 deg.

Index ranges -11≤*h*≤11, -32≤*k*≤31, -17≤*l*≤17

Reflections collected 28459

Independent reflections 6305 (*R(int)* = 0.0739)

Observed reflections 3748 (*I* > 2*σ*(*I*))

Absorption correction Semi-empirical from equivalents

Max. and min. transmission 0.96 and 0.92

Refinement method Full-matrix least-squares on F^2^

Data/restraints/parameters 6305 / 0 / 327

Goodness-of-fit on F^2^ 1.01

Final R indices (I>2sigma(I)) *R*1 = 0.056, *wR*2 = 0.117

Largest diff. peak and hole 0.25 and -0.25 eÅ^-3^

Crystal structure data of compound **3**.

Crystallized by slow diffusion of *n*-hexane into a dichloromethane solution.


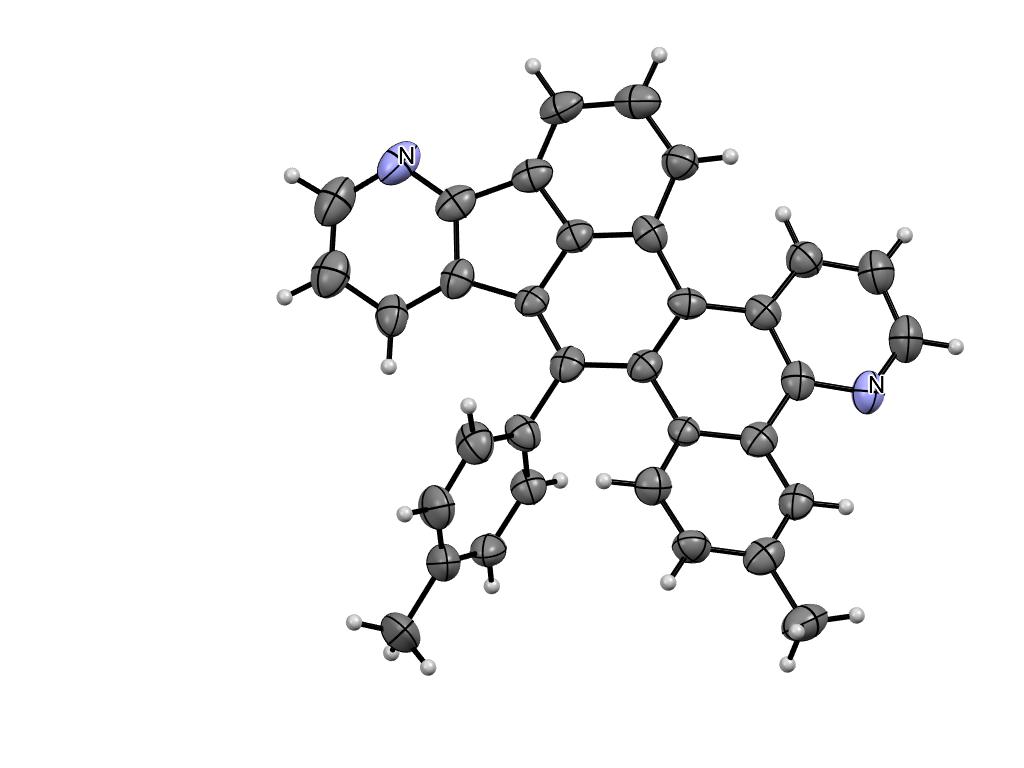


**Figure 75.** ORTEP representation of compound **3**. Ellipsoid probability level: 50%.

**Table S5**. Data and structure refinement for compound **3**.

CCDC-number 2486401

Empirical formula C_34_H_22_N_2_

Formula weight 458.53

Temperature 200(2) K

Wavelength 0.71073 Å

Crystal system monoclinic

Space group *P*2_1_/c

Z 8

Unit cell dimensions *a* = 10.3373(10) Å *α* = 90 deg.

*b* = 19.608(2) Å *β* = 96.886(3) deg.

*c* = 22.789(2) Å *γ* = 90 deg.

Volume 4585.8(8) Å^3^

Density (calculated) 1.33 g/cm^3^

Absorption coefficient 0.08 mm^-1^

Crystal shape irregular

Crystal size 0.114 x 0.053 x 0.045 mm^3^

Crystal colour yellow

Theta range for data collection 1.4 to 21.6 deg.

Index ranges -10≤*h*≤10, -20≤*k*≤20, -23≤*l*≤23

Reflections collected 31113

Independent reflections 5349 (*R(int)* = 0.0825)

Observed reflections 3679 (*I* > 2*σ*(*I*))

Absorption correction Semi-empirical from equivalents

Max. and min. transmission 0.96 and 0.91

Refinement method Full-matrix least-squares on F^2^

Data/restraints/parameters 5349 / 2427 / 916

Goodness-of-fit on F^2^ 1.09

Final R indices (I>2sigma(I)) *R*1 = 0.064, *wR*2 = 0.136

Largest diff. peak and hole 0.31 and -0.18 eÅ^-3^

Crystal structure data of pentahelicene **4**.

Crystallized by slow diffusion of *n*-hexane into a dichloromethane solution


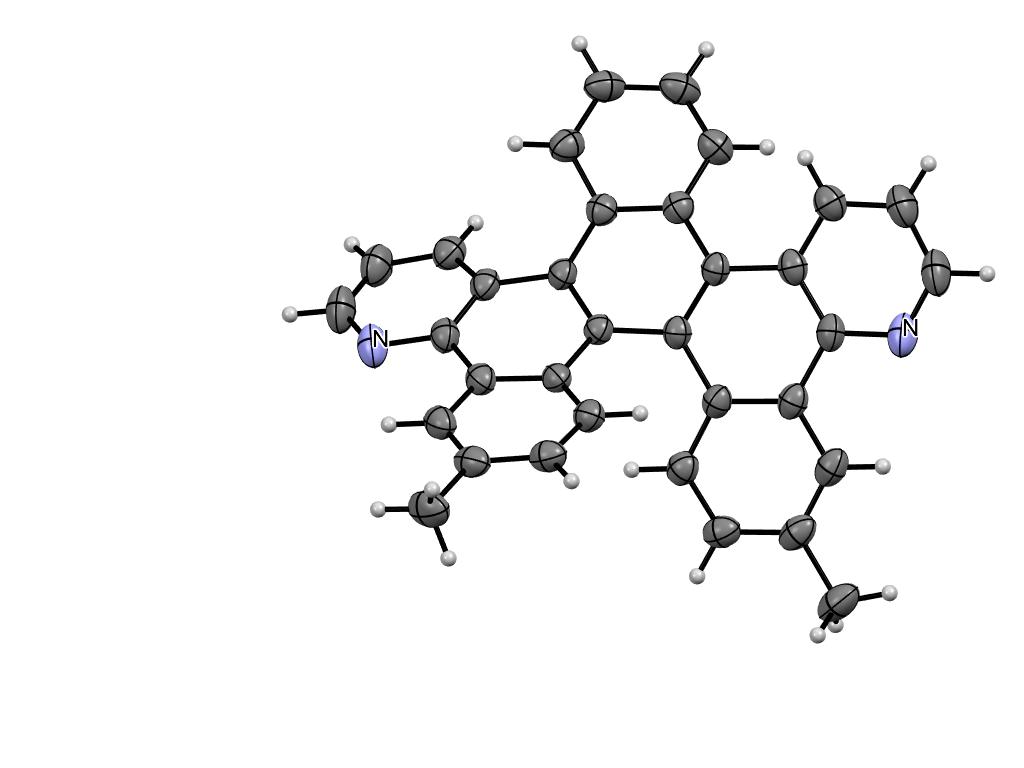


**Figure S76**. ORTEP representation of pentahelicene **4**. Ellipsoid probability level: 50%.

**Table S6**. Crystal data and structure refinement for pentahelicene **4**.

CCDC-number 2486402

Empirical formula C_34_H_22_N_2_

Formula weight 458.53

Temperature 200(2) K

Wavelength 1.54178 Å

Crystal system monoclinic

Space group *C*2/c

Z 4

Unit cell dimensions *a* = 15.7179(6) Å *α* = 90 deg.

*b* = 20.1560(9) Å *β* = 113.063(3) deg.

*c* = 7.6636(3) Å *γ* = 90 deg.

Volume 2233.86(16) Å^3^

Density (calculated) 1.36 g/cm^3^

Absorption coefficient 0.61 mm^-1^

Crystal shape column

Crystal size 0.060 x 0.028 x 0.025 mm^3^

Crystal colour pale yellow

Theta range for data collection 3.8 to 68.5 deg.

Index ranges -18≤*h*≤11, -24≤*k*≤22, -8≤*l*≤9

Reflections collected 11355

Independent reflections 2017 (R(int) = 0.0476)

Observed reflections 1269 (*I* > 2*σ*(*I*))

Absorption correction Semi-empirical from equivalents

Max. and min. transmission 0.99 and 0.86

Refinement method Full-matrix least-squares on F^2^

Data/restraints/parameters 2017 / 0 / 164

Goodness-of-fit on F^2^ 1.02

Final R indices (I>2sigma(I)) *R*1 = 0.042, *wR*2 = 0.093

Largest diff. peak and hole 0.17 and -0.16 eÅ^-3^

# 3 Calculations

## Kohn-Sham Molecular Orbitals


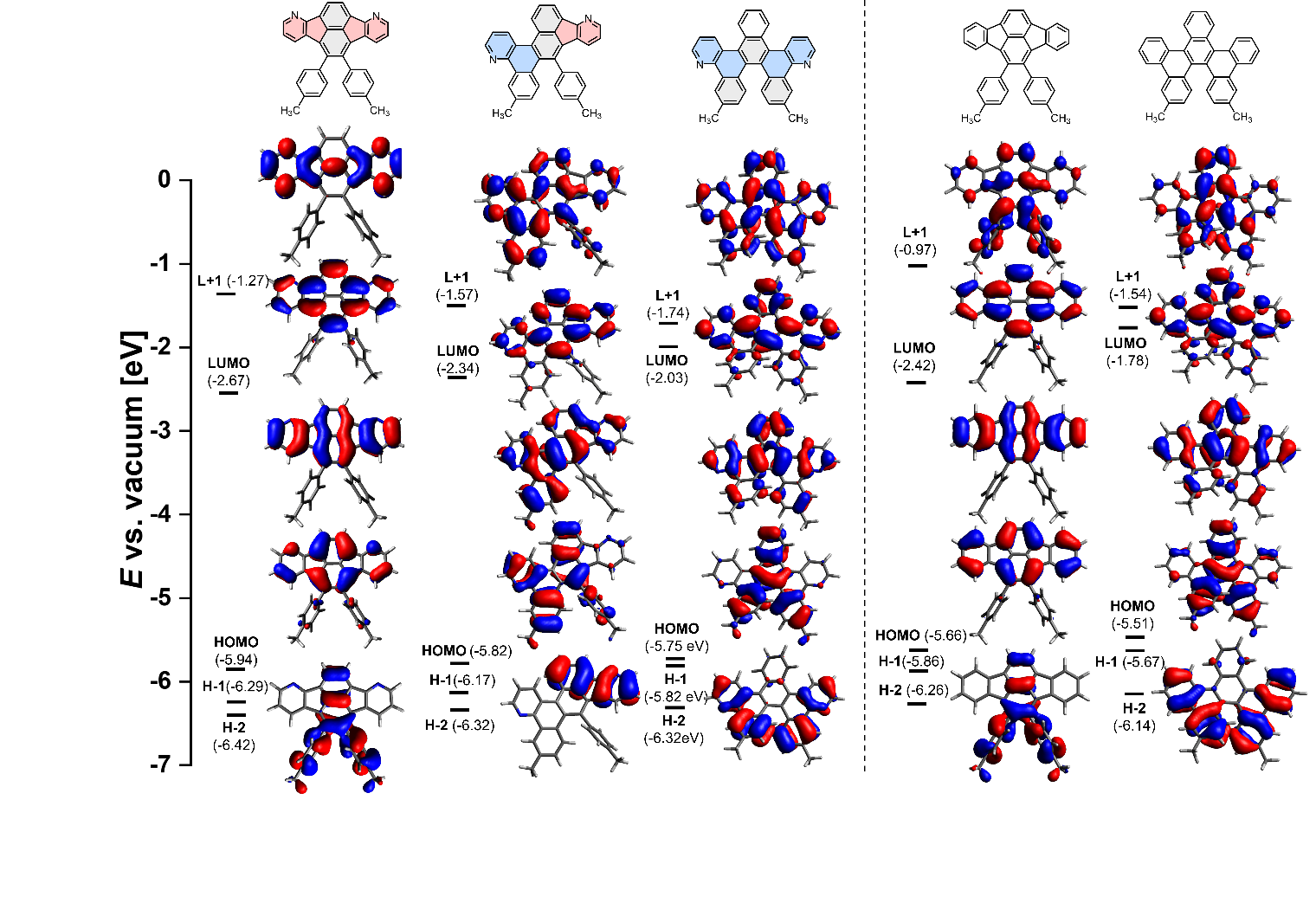


**Figure S77**. a) Kohn-Sham molecular orbitals of **2**-**4** and the all-carbon analogues of **2** (**14**) and **4** (**15**). Isosurface value = 0.026. Level of theory: B3LYP/6-311g(d,p).

## UV/Vis Absorption Spectra (TD-DFT)


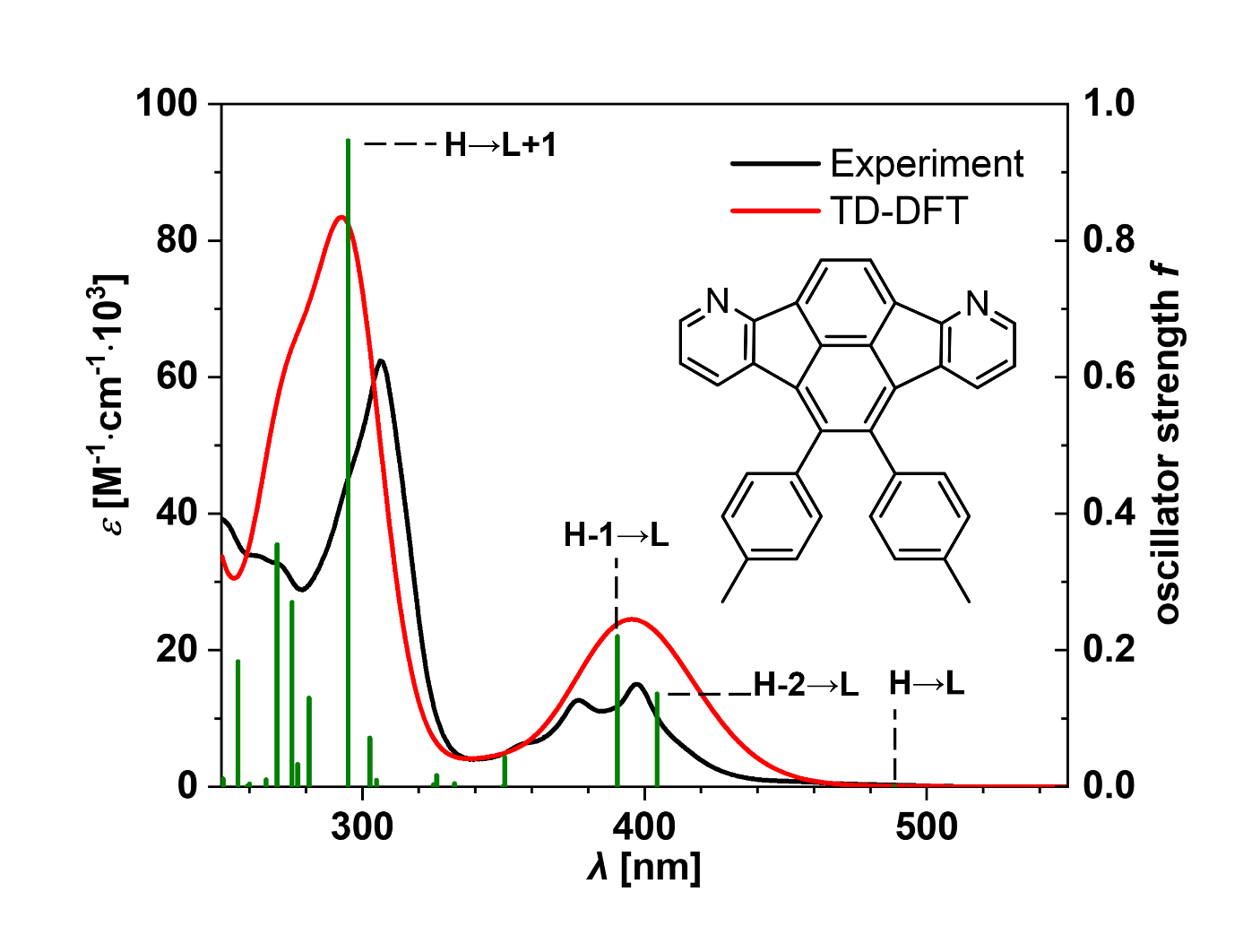
**Figure S78**. TD-DFT-simulated UV/Vis-absorption spectrum of fluoranthene **2** (B3LYP/6-311g(d,p) in CHCl_3_, red) and experimental spectrum (CHCl_3_, 23 °C, black). FWHM: 3000 cm^-1^.

**Table S7**. Gaussian output for the TD-DFT calculated UV/Vis absorption spectrum of fluoranthene **2**.

| No. | *λ* [nm] | *f* | Major Contributions |
| --- | --- | --- | --- |
| S_0_→S_1_ | 488 | 0.0006 | HOMO->LUMO (99%) |
| S_0_→S_2_ | 404 | 0.1361 | H-2->LUMO (97%) |
| S_0_→S_3_ | 390 | 0.2204 | H-1->LUMO (94%), HOMO->L+1 (4%) |
| S_0_→S_4_ | 350 | 0.0436 | H-8->LUMO (11%), H-3->LUMO (84%) |
| S_0_→S_5_ | 349 | 0.0001 | H-4->LUMO (88%), H-5->LUMO (7%), H-1->L+2 (2%) |
| S_0_→S_6_ | 336 | 0.0 | H-7->LUMO (84%), H-5->LUMO (12%), H-1->L+2 (2%) |
| S_0_→S_7_ | 333 | 0.0047 | H-8->LUMO (71%), H-6->LUMO (14%), H-3->LUMO (14%) |
| S_0_→S_8_ | 326 | 0.0168 | H-7->LUMO (13%), H-5->LUMO (80%) |
| S_0_→S_9_ | 325 | 0.0032 | H-8->LUMO (15%), H-6->LUMO (82%) |
| S_0_→S_10_ | 305 | 0.0097 | H-9->LUMO (67%), H-1->L+2 (10%), HOMO->L+2 (17%) |
| S_0_→S_11_ | 303 | 0.0716 | H-9->LUMO (14%), HOMO->L+2 (70%), H-10->LUMO (7%) |
| S_0_→S_12_ | 295 | 0.9465 | HOMO->L+1 (88%), H-11->LUMO (2%), H-2->L+2 (2%), H-1->LUMO (3%) |


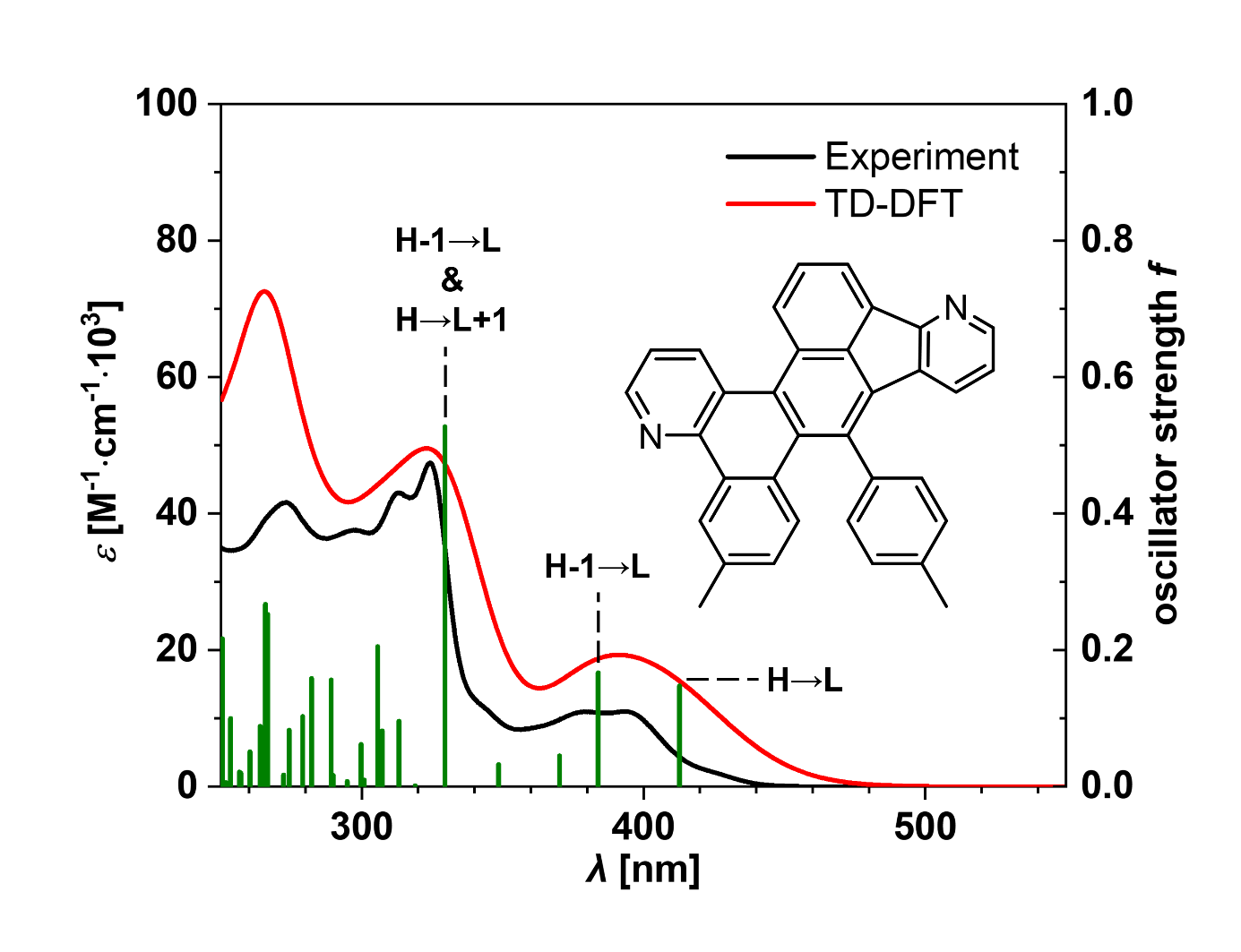


**Figure S79**. TD-DFT-simulated UV/Vis-absorption spectrum of compound **3** (B3LYP/6-311g(d,p) in CHCl_3_, red) and experimental spectrum (CHCl_3_, 23 °C, black). FWHM: 3000 cm^-1^.

**Table S8.** Gaussian output for the TD-DFT calculated UV/Vis absorption spectrum of compound **3**.

| No. | *λ* [nm] | *f* | Major Contributions |
| --- | --- | --- | --- |
| S_0_→S_1_ | 413 | 0.1477 | HOMO->LUMO (89%), H-2->LUMO (6%), H-1->LUMO (2%) |
| S_0_→S_2_ | 384 | 0.167 | H-1->LUMO (80%), HOMO->L+1 (11%), H-2->LUMO (3%) |
| S_0_→S_3_ | 370 | 0.0452 | H-2->LUMO (86%), H-3->LUMO (3%), H-1->LUMO (4%), HOMO->LUMO (4%) |
| S_0_→S_4_ | 348 | 0.0324 | H-3->LUMO (88%), H-2->LUMO (2%), HOMO->L+1 (3%), HOMO->L+2 (2%) |
| S_0_→S_5_ | 329 | 0.5275 | H-1->LUMO (10%), HOMO->L+1 (76%), H-4->LUMO (2%), H-3->LUMO (5%) |
| S_0_→S_6_ | 319 | 0.0015 | H-4->LUMO (39%), HOMO->L+2 (47%), H-7->LUMO (3%), H-6->LUMO (3%), H-1->L+1 (3%) |
| S_0_→S_7_ | 313 | 0.0958 | H-6->LUMO (28%), H-4->LUMO (20%), HOMO->L+2 (32%), H-7->LUMO (8%), H-1->L+1 (2%), HOMO->L+1 (2%) |
| S_0_→S_8_ | 307 | 0.0817 | H-1->L+1 (59%), HOMO->L+3 (21%) H-7->LUMO (6%), H-6->LUMO (3%), H-4->LUMO (2%), H-2->L+1 (2%) |
| S_0_→S_9_ | 305 | 0.2054 | H-7->LUMO (34%), H-4->LUMO (17%), H-1->L+1 (14%), H-6->LUMO (7%), H-5->LUMO (8%), H-2->L+1 (3%), HOMO->L+2 (9%) |
| S_0_→S_10_ | 301 | 0.0099 | H-6->LUMO (35%), H-5->LUMO (32%), H-4->LUMO (14%), H-8->LUMO (3%), H-7->LUMO (2%), H-2->L+1 (6%) |


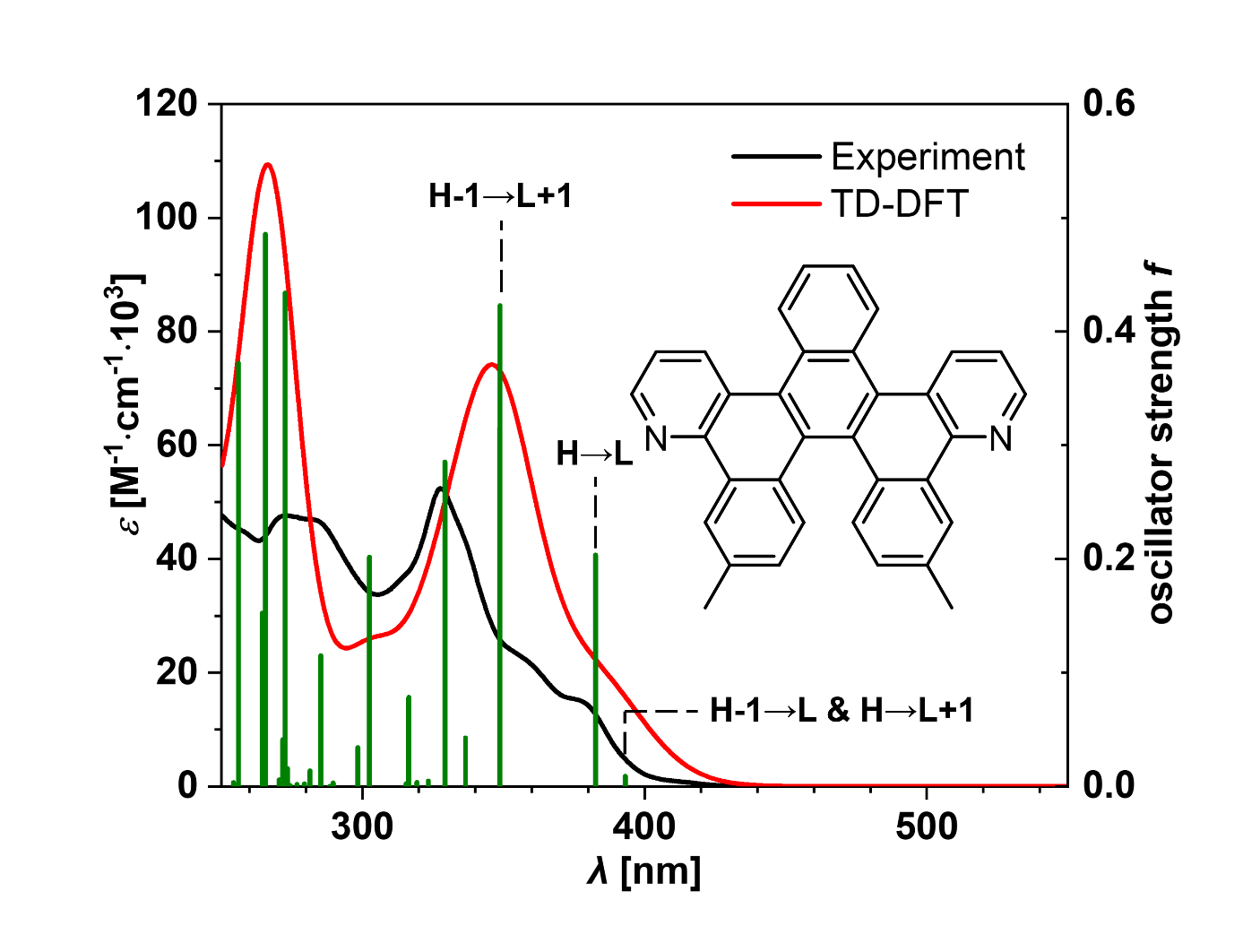


**Figure S80.** TD-DFT-simulated UV/Vis-absorption spectrum of pentahelicene **4** (B3LYP/6-311g(d,p) in CHCl_3_, red) and experimental spectrum (CHCl_3_, 23 °C, black). FWHM: 2600 cm^-1^.

**Table S9.** Gaussian output for the TD-DFT calculated UV/Vis absorption spectrum of pentahelicene **4**.

| No. | *λ* [nm] | *f* | Major Contributions |
| --- | --- | --- | --- |
| S_0_→S_1_ | 393 | 0.009 | H-1->LUMO (66%), HOMO->L+1 (31%) |
| S_0_→S_2_ | 382 | 0.2035 | HOMO->LUMO (90%) |
| S_0_→S_3_ | 349 | 0.4229 | H-1->L+1 (88%) |
| S_0_→S_4_ | 349 | 0.3152 | H-2->LUMO (19%), H-1->LUMO (23%), HOMO->L+1 (45%), H-1->L+3 (5%), HOMO->L+2 (5%) |
| S_0_→S_5_ | 336 | 0.0427 | H-3->LUMO (38%), H-1->L+2 (17%), HOMO->L+3 (33%), H-2->L+1 (8%), H-1->L+1 (2%) |
| S_0_→S_6_ | 329 | 0.2854 | H-2->LUMO (45%), HOMO->L+1 (16%), HOMO->L+2 (23%), H-3->L+1 (3%), H-1->LUMO (7%) |
| S_0_→S_7_ | 323 | 0.005 | H-2->LUMO (30%), HOMO->L+2 (61%), H-3->L+1 (4%), HOMO->L+1 (2%) |
| S_0_→S_8_ | 319 | 0.0037 | H-1->L+2 (70%), HOMO->L+3 (14%), H-3->LUMO (9%), H-2->L+1 (5%) |
| S_0_→S_9_ | 316 | 0.0784 | H-3->LUMO (48%), HOMO->L+3 (47%) |
| S_0_→S_10_ | 315 | 0.0023 | H-3->L+1 (11%), H-1->L+3 (76%) |

## NICS(0) & NICS(1)


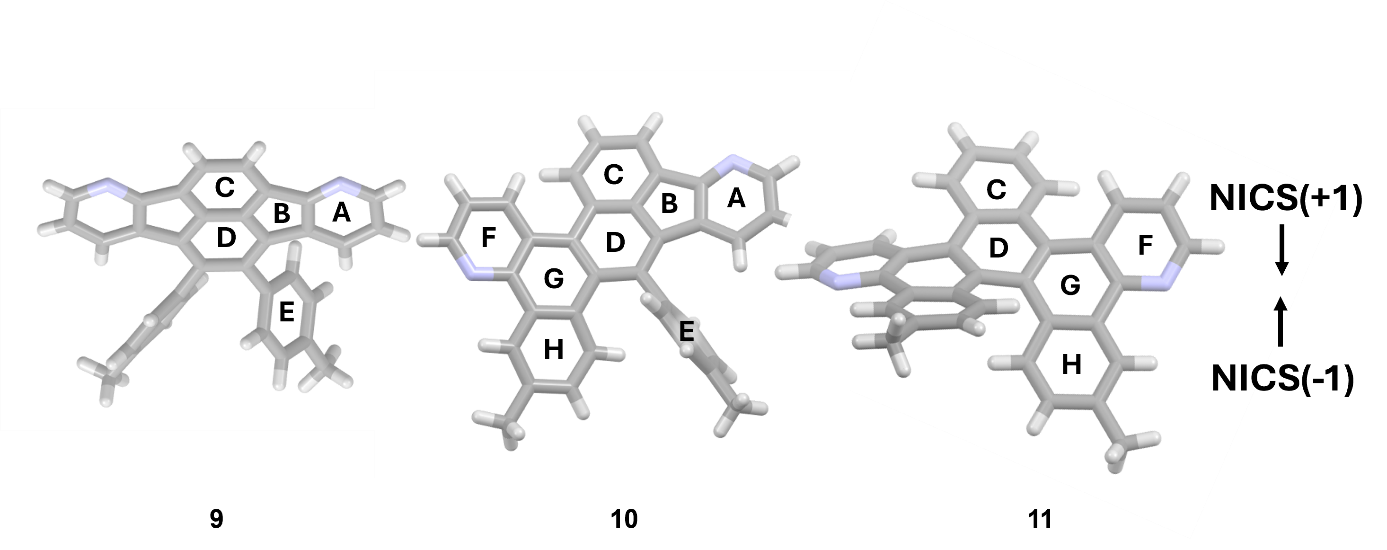


**Figure S81**. Calculated NICS(1)av-values of **2-4** (Level of theory: HF-GIAO/6-311g(d,p)).

**Table S10**. Calculated NICS-values of fluoranthene **2** (Level of theory: HF-GIAO/6-311g(d,p).

| Ring | fluoranthene **2** | | | |
| --- | --- | --- | --- | --- |
|  | **NICS(0)** | **NICS(+1)** | **NICS(-1)** | **NICS(1)_av_** |
| A | -5.6 | -8.9 | -9.2 | -9.1 |
| B | 7.6 | 2.3 | 2.0 | +2.2 |
| C | -6.5 | -8.9 | -8.8 | -8.9 |
| D | -5.3 | -7.4 | -7.3 | -7.4 |
| E | -9.8 | -11.6 | -10.9 | -11.3 |
| F | - | - | - | - |
| G | - | - | - | - |
| H | - | - | - | - |

**Table S11**. Calculated NICS-values of compound **3** (Level of theory: HF-GIAO/6-311g(d,p).

| Ring | Compound **3** | | | |
| --- | --- | --- | --- | --- |
|  | **NICS(0)** | **NICS(+1)** | **NICS(-1)** | **NICS(1)_av_** |
| A | -6.7 | -10.4 | -9.8 | -10.1 |
| B | 3.9 | -1.0 | -0.4 | -0.7 |
| C | -8.8 | -10.2 | -11.6 | -10.9 |
| D | -6.3 | -8.7 | -8.6 | -8.7 |
| E | -8.9 | -10.1 | -10.0 | -10.1 |
| F | -8.1 | -12.2 | -10.3 | -11.3 |
| G | -3.0 | -6.4 | -6.5 | -6.5 |
| H | -10.2 | -11.5 | -12.7 | -12.1 |

**Table S12**. Calculated NICS-values of pentahelicene **4** (Level of theory: HF-GIAO/6-311g(d,p).

| Ring | pentahelicene **4** | | | |
| --- | --- | --- | --- | --- |
|  | **NICS(0)** | **NICS(+1)** | **NICS(-1)** | **NICS(1)_av_** |
| A | - | - | - | - |
| B | - | - | - | - |
| C | -9.3 | -11.3 | -11.3 | -11.3 |
| D | -3.8 | -7.2 | -7.2 | -7.2 |
| E | - | - | - | - |
| F | -8.3 | -10.1 | -12.4 | -11.3 |
| G | -3.3 | -4.8 | -8.6 | -6.7 |
| H | -9.5 | -9.9 | -12.7 | -11.3 |

## Charge-Transfer Integrals

Description of methods used: A supercell of *aza*-indeno-*aza*-fluoranthene (**2**) was constructed based on the experimental crystal structure and contains 20*10*5 molecules arranged along the crystallographic axes. Geometry optimization was performed at the B3LYP/6-311G* level of theory. Force field parameters were obtained from the General AMBER Force Field (GAFF),^[S33]^ with atomic partial charges derived using the restrained electrostatic potential (RESP) fitting method based on calculations at the HF/6-311G* level, as implemented in Gaussian09. The charge transfer Hamiltonian was formulated using a fragment orbital approach, where the matrix elements were computed using the Density Functional Tight Binding (DFTB) method.^[S34-35]^ In this Hamiltonian, the diagonal elements represent the site energies, while the off-diagonal elements correspond to the electronic couplings between molecular sites.

The reorganization energies for hole and electron transport, as calculated by DFTB for fluoranthene **2**, are *λ*_h_ = 150.3 meV and *λ*_e_ = 201.5 meV.

**Table S13**. Transfer Integral values (t) and standard deviation (σ) of **2**-**4** in meV in different directions for hole (h) and electron (e) transport, calculated with DFTB.

| Compound | Direction | *t*_h_ | *σ*_h_ | *t*_e_ | *σ*_e_ |
| --- | --- | --- | --- | --- | --- |
| **2** | d_1_ (π-π) | 14.9 | 83.0 | 33.7 | 22.9 |
| **2** | *d*_2_ (N–H) | 0.8 | 1.2 | 0.6 | 0.6 |
| **2** | *d*_3_ (VdW) | 0.6 | 0.9 | 0.3 | 0.4 |
| **3** | *d*_6_ (π-π) | 13.4 | 11.2 | 25.5 | 17.2 |
| **3** | *d*_7_ (π-π) | 22.5 | 21.8 | 36.3 | 15.6 |
| **4** | *d*_8_ (π-π) | 21.3 | 21.7 | 14.4 | 31.8 |

## Calculation of One-Dimensional Offsets in Crystal-Packing of Fluoranthene 2

The crystal structure was converted to an.xyz-file using Mercury and then processed with Avogadro by aligning the axes along the molecular axes. The processed .xyz-file was opened in Mercury and centroids of the π-scaffold were generated. The coordinates of the centroids were used to calculate the offsets into x-, y-, and z-directions (Figure S82).


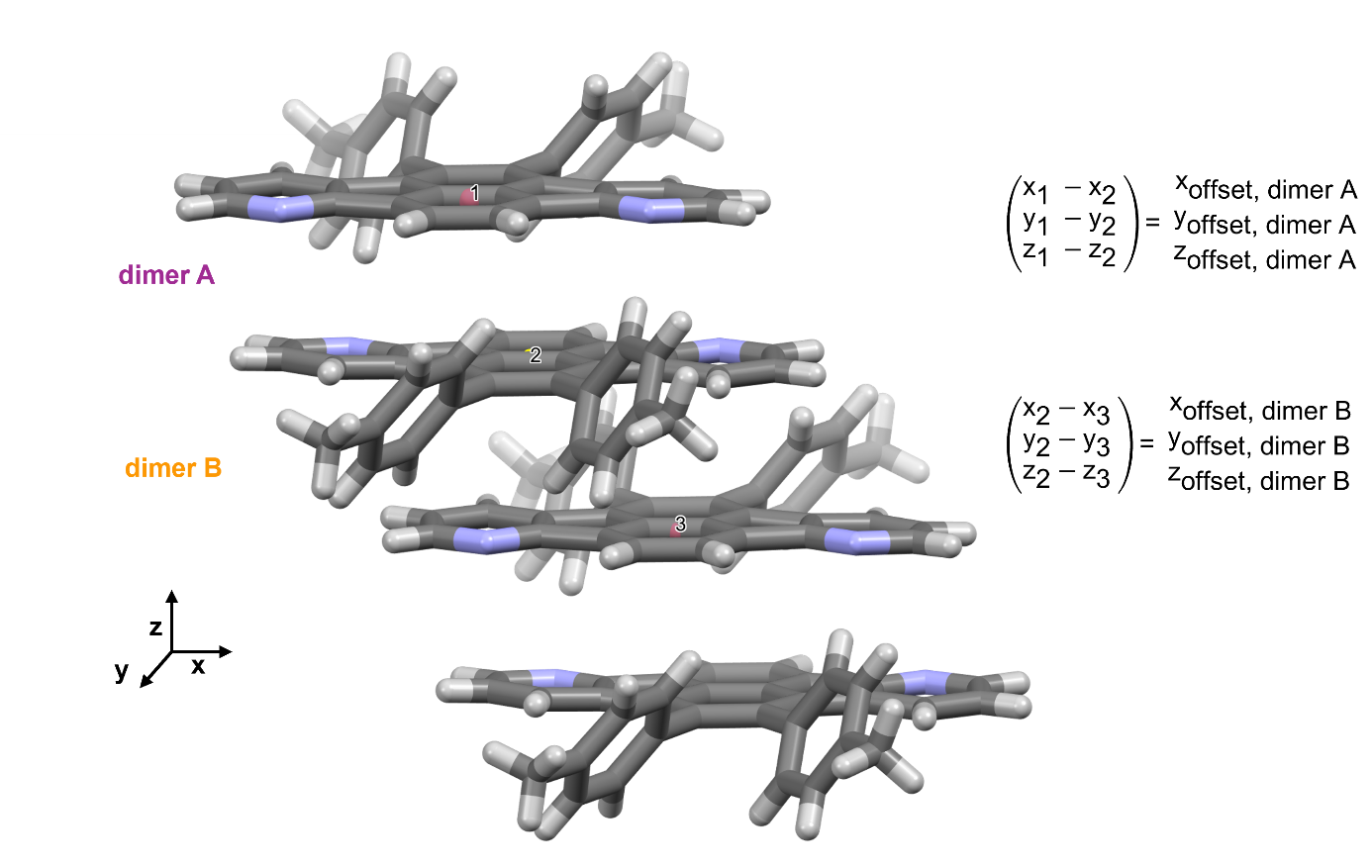


**Figure S82** Calculation of the offset distances in the crystal packing of fluoranthene **2**.

## Reaction Mechanism

Optimized Geometries (L = PMe_3_). No imaginary frequency was found, unless stated otherwise. Integers (e.g. “0 1”) at the beginning of each geometry data set indicate charge (first integer, charge = 0 or -1) and multiplicity (second integer, 1 = singlet).

0 1

C 0.50451700 -2.65730500 0.25116200

C -0.04969300 -1.36476900 0.05094900

C -1.52629300 -1.20964500 0.09163200

C -2.34088800 -1.99115600 -0.73241800

C -3.72534800 -1.84592800 -0.71329800

C -4.34171700 -0.92522000 0.13548500

C -3.52420500 -0.15446800 0.96943000

C -2.14304000 -0.29175500 0.95064200

C -5.84092000 -0.77534600 0.17495400

N -0.24796100 3.32231800 0.20496100

C -0.82886100 3.54034400 -0.97921500

C -0.92529600 2.56318000 -1.96189700

C -0.38557200 1.31002300 -1.69669000

C 0.23079000 1.05436000 -0.47118900

C 0.25135200 2.12689700 0.43149500

C 1.85760600 -2.86153700 0.23209500

C 0.79756200 -0.29004900 -0.17887300

Cl 0.96262700 1.87799100 2.02759500

C 5.00037700 -0.92265300 -0.28080300

C 4.15376100 -1.97478100 -0.03569000

C 2.74944500 -1.78634400 -0.00196700

C 2.21354000 -0.47907900 -0.21842300

C 3.11743000 0.58378100 -0.47856900

C 4.47332800 0.36810300 -0.50725300

H -0.17053500 -3.48309700 0.44680800

H -1.88379700 -2.70363600 -1.41184200

H -4.33450100 -2.45647600 -1.37334900

H -3.97700600 0.56346700 1.64718900

H -1.53557500 0.30675300 1.61888700

H -6.25716300 -1.20221400 1.09338800

H -6.13879100 0.27632200 0.14631700

H -6.31731800 -1.28127000 -0.66721000

H -1.23079200 4.53731200 -1.13270800

H -1.41076000 2.77644400 -2.90696400

0 1

C 1.52016100 2.08201500 -2.03385600

C 0.84545200 1.76645800 -0.82415700

C -0.60661300 2.07231300 -0.78384700

C -1.11971900 2.95298700 0.17290500

C -2.44453300 3.37274200 0.11463400

C -3.30274700 2.91763700 -0.89024700

C -2.78700700 2.02869700 -1.83795400

C -1.46293100 1.61148000 -1.79026700

C -4.75090500 3.33148300 -0.92353500

N -0.31869300 -0.86638900 2.70697200

C -0.01005500 -0.26311600 3.86173800

C 0.69221800 0.93112100 3.92353800

C 1.15989100 1.46412500 2.72602100

C 0.90191300 0.82009000 1.51527900

C 0.08144500 -0.33985300 1.54517600

C 2.87881500 1.98188500 -2.14453700

C 1.56561700 1.29651100 0.26710900

Pd -0.64346900 -1.16182300 -0.15174000

C 5.82477200 1.06759500 -0.05948000

C 5.07046000 1.47808200 -1.12910400

C 3.65822700 1.56629400 -1.03936600

C 2.99967700 1.21855400 0.18390400

C 3.81845800 0.78953200 1.26630500

C 5.18588500 0.72100400 1.15032000

Cl -1.52844800 -1.99607800 -2.30798200

P 1.35529000 -2.25534700 -0.66871300

C 0.95107500 -4.01719300 -0.98407200

C 2.68013800 -2.39414500 0.58882300

C 2.20699400 -1.72198900 -2.19651300

P -2.83543000 -0.89584800 0.70403300

C -3.11302800 -2.43741300 1.65924400

C -3.26487200 0.41705100 1.90929100

C -4.24323400 -0.86918600 -0.46662000

H 0.93081100 2.43874900 -2.87054700

-1 1

Pd -0.52062400 -0.73449700 1.03724900

O -0.61317100 -2.82145400 0.83828900

O 1.30995000 -1.73784800 0.80115900

C 0.71526700 -2.92235400 0.70545200

O 1.30076500 -3.97568400 0.50499100

C 1.90125300 -0.87560500 -2.40067700

C 1.14309500 0.08544200 -1.68524800

C -0.28376100 0.24068400 -2.06773100

C -0.82630300 1.47723600 -2.42194800

C -2.15000300 1.58520400 -2.84239100

C -2.97533000 0.46249300 -2.92075100

C -2.43385000 -0.77380500 -2.55213100

C -1.11604800 -0.88738700 -2.12813000

C -4.42021500 0.57841400 -3.33703600

N -0.73869700 2.10753100 1.85791800

C -0.44726100 3.41314400 1.83108400

C 0.51641000 3.96918800 1.00285300

C 1.22108500 3.10229000 0.16956100

C 0.94509100 1.73861200 0.19248600

C -0.06868600 1.24553100 1.06144300

C 3.21837900 -1.10216900 -2.11707700

C 1.73434900 0.80340200 -0.65650900

C 5.82398600 0.05689800 0.27773900

C 5.22139600 -0.61112100 -0.75888800

C 3.86094800 -0.38528300 -1.08277400

C 3.10812500 0.56995000 -0.33201300

C 3.75617500 1.21977300 0.75182400

C 5.07586200 0.97539600 1.04604900

P -2.70838300 -0.30822800 1.41253400

C -3.57479200 1.10225700 0.62219600

C -3.07595600 -0.05359100 3.19330900

C -3.73290400 -1.75876300 0.94707400

H 1.40937900 -1.43721900 -3.18744600

H -0.20361200 2.36279700 -2.37212200

H -2.54622400 2.56085900 -3.11220000

H -3.05744400 -1.66345900 -2.58276400

H -0.73797700 -1.84697800 -1.79614000

H -4.61747400 1.52845000 -3.84002800

H -4.70928400 -0.22773700 -4.01772700

H -5.09033700 0.52250700 -2.47106200

H -1.02620500 4.04355900 2.50619100

H 0.70830000 5.03776700 1.00551900

H 1.99190300 3.48129900 -0.49697600

H 3.77935400 -1.85105200 -2.66799700

H 6.86447600 -0.13500200 0.52305900

H 5.77581900 -1.34434200 -1.33815200

H 3.18675200 1.90134800 1.36958200

H 5.54195600 1.47838900 1.88825700

H -4.63901200 1.11238100 0.87758600

H -3.10364900 2.02251600 0.96373500

H -3.45329600 1.03448100 -0.45744300

H -4.14057600 0.12255100 3.37857600

H -2.49162500 0.80866500 3.51634100

H -2.74783700 -0.93036900 3.75377400

H -4.75217600 -1.70027600 1.34111900

H -3.76521900 -1.82293700 -0.14142600

H -3.22891500 -2.65576700 1.30872900

Transition state, imaginary frequency = -61 cm^-1^ (corresponds to Pd-C and O-H-C vibration)

-1 1

C -0.81836000 -0.53894100 -2.84588200

C -0.22208600 -1.10447100 -1.69355100

C 1.26067300 -1.18065000 -1.59044200

C 1.90807600 -2.38302100 -1.29105900

C 3.27166200 -2.39966900 -1.01884200

C 4.01838600 -1.21788600 -1.00653300

C 3.36759300 -0.02640900 -1.33946400

C 2.00882600 0.00902300 -1.66836200

C 5.46994200 -1.22064400 -0.59761100

N 1.11916700 -1.31132600 2.45804000

C 0.91789200 -2.52603300 2.98546400

C 0.08156200 -3.47742200 2.41866700

C -0.57047900 -3.14355500 1.23177300

C -0.36284100 -1.89294100 0.66049800

C 0.51464100 -0.97970900 1.30939600

C -2.17711800 -0.41015100 -2.94616100

C -1.01298900 -1.48959100 -0.61529200

C -5.23144300 -0.95039100 -0.87733200

C -4.42715300 -0.63718400 -1.94503900

C -3.02125100 -0.81053000 -1.88328200

C -2.43358900 -1.34521600 -0.69091200

C -3.29628500 -1.63959400 0.40058100

C -4.65434700 -1.44944400 0.31148100

H 1.46736100 1.09116000 -2.01617900

O 0.62364100 2.46173600 -2.28851800

C 0.60146200 3.11508900 -1.19359900

O 1.45182100 2.70384100 -0.20129300

Pd 0.96299600 0.82481900 0.50912500

O -0.15363700 4.08626800 -0.93838400

P -0.68983600 1.83841700 1.64533900

C -0.22474200 3.50758700 2.21054100

C -2.13486100 2.13777000 0.57038200

C -1.36069100 0.95553100 3.10710700

H -0.17189000 -0.17222200 -3.63290600

H 1.33489800 -3.30267900 -1.23518100

H 3.75580700 -3.34195800 -0.77403800

H 3.91563400 0.91171500 -1.33666800

H 5.57563700 -0.99302200 0.46910500

H 6.04234900 -0.46748600 -1.14559200

H 5.93757100 -2.19391400 -0.77216600

H 1.45100700 -2.73813700 3.91126800

H -0.05421500 -4.44829800 2.88487100

H -1.22587700 -3.85888600 0.74171000

H -2.62010300 0.04401700 -3.82707100

H -6.30504700 -0.80001200 -0.93720000

H -4.85652400 -0.22801600 -2.85516400

H -2.86552300 -2.00587500 1.32348600

H -5.28726400 -1.67405200 1.16478300

H 0.67752000 3.46009000 2.82057300

H -0.01626700 4.06788000 1.29253600

H -1.04210100 3.96586900 2.77739100

H -2.94267300 2.60697300 1.14115100

H -1.80140400 2.81815400 -0.21959600

H -2.48738000 1.20212300 0.14037500

H -0.54309800 0.68057000 3.77386800

H -2.08640200 1.57625400 3.64019300

H -1.84592900 0.03706300 2.77473500

-1 1

C -0.05613400 -1.81501400 -2.42885800

C 0.27822800 -1.68839500 -1.05626800

C 1.71123400 -1.49272700 -0.70128500

C 2.66755400 -2.41531800 -1.13611600

C 4.01923600 -2.21826700 -0.86725300

C 4.44009000 -1.08409300 -0.17152100

C 3.47275900 -0.16074100 0.24519800

C 2.10760700 -0.33301700 0.00000000

C 5.89926500 -0.86073500 0.14364000

N 0.37757900 -0.16988400 3.13981700

C 0.06478400 -1.15178600 3.99518300

C -0.48186700 -2.36796700 3.60826100

C -0.73700600 -2.56135400 2.25068100

C -0.42664700 -1.55220900 1.34592400

C 0.15784500 -0.35605400 1.83100700

C -1.35637900 -1.90634800 -2.84793300

C -0.73570400 -1.68525800 -0.10358600

C -4.79416100 -1.77893500 -1.40818900

C -3.77413500 -1.88142100 -2.32136900

C -2.41636600 -1.85831900 -1.91302300

C -2.10345500 -1.74428700 -0.52007500

C -3.18699500 -1.62948900 0.39470500

C -4.49240600 -1.64404600 -0.03449400

H 2.01174300 0.91799000 -2.04054700

O 1.50587200 1.30294700 -2.77066900

C 0.83186100 2.40305000 -2.25191000

O 1.07918200 2.66036000 -1.01530000

Pd 0.64521100 1.04851000 0.50704400

O 0.08726300 3.01163000 -3.00678400

P -1.26806800 2.38936100 0.82217200

C -0.94912400 4.18910000 0.99062600

C -2.32986600 2.30110700 -0.67208600

C -2.43334500 2.00519800 2.19511300

H 0.74771300 -1.77683100 -3.15442900

H 2.35088800 -3.29944100 -1.68419800

H 4.75027000 -2.94913000 -1.20506700

H 3.80104100 0.73102600 0.77464800

H 6.11838500 -1.07640800 1.19592400

H 6.19202400 0.17773400 -0.03709200

H 6.54540300 -1.50123100 -0.46338800

H 0.26655400 -0.94732800 5.04613900

H -0.70321600 -3.13737800 4.34134700

H -1.17746200 -3.48916600 1.89539200

H -1.58921500 -1.96859700 -3.90695900

H -5.82861900 -1.78675200 -1.73816800

H -3.99243300 -1.96549400 -3.38245600

H -2.96934700 -1.51175200 1.44823400

H -5.29769500 -1.53972700 0.68652900

H -0.44071300 4.38616400 1.93674400

H -0.27938800 4.45329100 0.17139100

H -1.86621400 4.78392800 0.93754800

H -3.22688500 2.92062200 -0.57047100

H -1.74507000 2.63861400 -1.53146200

H -2.62571200 1.26412900 -0.84101900

H -1.88863700 2.00774400 3.14075900

H -3.26777000 2.71112300 2.24627400

H -2.82718300 0.99862200 2.04280000

0 1

C -0.75333300 -2.41638400 -0.48494800

C -0.10590300 -1.16968100 -0.23705200

C 1.34579800 -1.09138300 -0.12133100

C 2.17066400 -2.23746300 -0.12083700

C 3.53293500 -2.14199300 0.06483300

C 4.15866800 -0.89560000 0.26012500

C 3.36458100 0.23536400 0.25616900

C 1.97259100 0.16065200 0.06726600

C 5.64869800 -0.81445200 0.46389300

N 1.83555100 2.55027300 0.00155300

C 1.15971100 3.66484800 -0.21219400

C -0.20737500 3.67915500 -0.51426900

C -0.89681300 2.48456800 -0.48864100

C -0.23315400 1.28476800 -0.15455900

C 1.17544900 1.37298700 -0.00808900

C -2.10989500 -2.52407100 -0.52155600

C -0.89297700 -0.01340700 -0.09132800

C -5.11770000 -0.53142500 0.39968100

C -4.33487400 -1.56418400 -0.05821900

C -2.93412700 -1.41734700 -0.18435500

C -2.32074500 -0.15808000 0.09326300

C -3.15064100 0.85699200 0.63618100

C -4.50812900 0.67732000 0.78463200

H -0.15646500 -3.29570000 -0.68530500

H 1.73846800 -3.22165000 -0.24434600

H 4.13392100 -3.04695800 0.06692700

H 3.79888400 1.21828700 0.38799500

H 5.97635000 0.21688500 0.60407400

H 5.96141800 -1.38752800 1.34247900

H 6.19011200 -1.22397400 -0.39477400

H 1.73185700 4.58908900 -0.18014000

H -0.70393100 4.60574700 -0.77929100

H -1.93960600 2.46491200 -0.77142900

H -2.57815800 -3.47500200 -0.75555000

H -6.18991100 -0.65829900 0.50362900

H -4.77888700 -2.52447400 -0.30206900

H -2.70589000 1.77105300 1.00262800

H -5.10758700 1.46861400 1.22230300

-1 1

C -1.14011500 2.76520100 -0.82419800

C -1.44654100 1.55693400 -0.13570200

C -2.76186400 0.92106700 -0.40994800

C -3.95484200 1.61845700 -0.21091900

C -5.18591800 1.02783800 -0.49207700

C -5.26113600 -0.27290300 -0.98854100

C -4.06207200 -0.96389400 -1.20224900

C -2.83485900 -0.38310800 -0.91932900

C -6.58742100 -0.92700700 -1.28466000

N 0.13612500 -2.61923900 1.44688700

C -0.92690400 -2.91346200 2.20521500

C -1.90612100 -1.99320300 2.56379400

C -1.77915500 -0.68976800 2.08908800

C -0.68649800 -0.37174000 1.29143000

C 0.28531300 -1.37036000 0.98832200

C 0.10752000 3.32154600 -0.77462100

C -0.48790900 0.95963000 0.66873800

C 3.40337000 2.70034700 0.87123700

C 2.43360600 3.26715300 0.07318200

C 1.12698400 2.73694600 0.01987000

C 0.81232400 1.56842400 0.78740600

C 1.81286400 1.05837100 1.67550300

C 3.08307000 1.61327700 1.69781200

Pd 1.88483100 -0.73519500 -0.08029200

H -1.90390200 3.20011000 -1.46065100

H -3.91950400 2.62881400 0.18555500

H -6.10042600 1.58873400 -0.31732000

H -4.08981000 -1.97610500 -1.59619500

H -1.91926100 -0.93736100 -1.08842400

H -7.41872200 -0.23469000 -1.13123900

H -6.63686200 -1.28237700 -2.31877900

H -6.75690100 -1.79588500 -0.64003000

H -0.99403900 -3.94610100 2.54677200

H -2.74393500 -2.28838700 3.18777100

H -2.52885300 0.06151400 2.31974900

H 0.33984500 4.20707600 -1.35872900

H 4.41214000 3.09858200 0.86993200

H 2.66963500 4.12702100 -0.54679800

H 1.54684400 0.29809600 2.39662400

H 3.83185100 1.19617100 2.36168300

O 3.51831600 -0.38919300 -1.38337400

O 2.38056600 -2.26629500 -1.36449600

C 3.39620900 -1.58456000 -1.92106100

O 4.09078700 -2.05234500 -2.80893600

-1 1

C -2.42547500 2.78050900 0.55409300

C -2.49905400 1.56909400 -0.14308300

C -3.62914100 0.62511500 0.06557900

C -3.48576100 -0.39697100 1.01538400

C -4.50489300 -1.32593700 1.19180700

C -5.68546400 -1.27047700 0.44175400

C -5.82094900 -0.24596000 -0.49623300

C -4.80490800 0.69142500 -0.68242200

C -6.78411500 -2.28217700 0.65927100

N 0.93657300 -0.87073000 -2.76840400

C 0.24863200 -2.00216300 -3.00524800

C -1.06666800 -2.20583600 -2.60259500

C -1.74316100 -1.19761200 -1.91102600

C -1.04808500 -0.02448400 -1.64476600

C 0.29658600 0.07243000 -2.09058600

C -1.25894700 3.54657600 0.58925200

C -1.39064500 1.20697900 -0.91958100

C 2.28245500 2.76491500 -0.17399500

C 1.24789900 3.59299200 0.12354600

C -0.11665000 3.13413200 -0.11110200

C -0.26303900 2.02353700 -0.93470100

C 0.87572600 1.41764500 -1.70580600

C 2.13683100 1.46051700 -0.84170300

H 1.05339000 1.99616700 -2.63266800

H 3.01670500 1.21313600 -1.43669700

Pd 1.90835100 -0.00254500 0.70115200

O 1.22576400 -1.37360200 2.15643800

O -0.03826900 0.29963600 1.47047600

C 0.03337200 -0.78862300 2.22055000

O -0.90539400 -1.21875400 2.88739300

P 3.83123900 -1.03712000 0.17940300

C 3.62711200 -2.22396500 -1.20668700

C 5.36116800 -0.12633500 -0.29888900

C 4.39459000 -2.10178200 1.56541200

H -3.27315300 3.07534500 1.16560100

H -2.57558700 -0.47187700 1.60687200

H -4.36781200 -2.11469800 1.92701200

H -6.72746400 -0.18134300 -1.09367100

H -4.91626500 1.47302500 -1.42830100

H -6.38991100 -3.30261500 0.68318800

H -7.29787900 -2.11651900 1.61301100

H -7.53743600 -2.23351800 -0.13161600

H 0.78679700 -2.78164100 -3.54074000

H -1.55698700 -3.14927800 -2.82082300

H -2.76441100 -1.33627900 -1.57917100

H -1.20919500 4.41237600 1.24465900

H 3.28467600 3.04313500 0.14692000

H 1.41286400 4.50310700 0.69426900

H 3.23364800 -1.69283100 -2.07456800

H 4.56113600 -2.73759000 -1.45686000

H 2.87416500 -2.95714400 -0.91263200

H 5.17096500 0.46181600 -1.19808400

H 6.19690100 -0.80596700 -0.49061700

H 5.63493700 0.56194700 0.50279800

H 5.15420200 -2.82710500 1.25820300

H 4.79238000 -1.47294100 2.36417100

H 3.51329500 -2.60923000 1.96071500

0 1

C 0.08008800 -2.55758300 0.00656900

C 0.43666000 -1.17205000 0.00187000

C 1.86939300 -0.79829900 0.01786900

C 2.75872400 -1.31091600 -0.93239100

C 4.10469700 -0.96147200 -0.90922600

C 4.61183600 -0.10203200 0.06863400

C 3.72241000 0.40024000 1.02382000

C 2.37539300 0.05959300 1.00123500

C 6.07703600 0.24771600 0.11414600

N -2.55290600 2.81512100 -0.09542400

C -1.63779300 3.79082300 -0.20050800

C -0.26431800 3.56363700 -0.26372400

C 0.22822700 2.25730700 -0.19818000

C -0.69289800 1.22698300 -0.06881900

C -2.07955800 1.57683000 -0.03843600

C -1.22202700 -3.00634500 0.04157600

C -0.59399000 -0.24551100 0.00399100

C -4.60650800 -1.31854700 0.10679900

C -3.68818000 -2.35272000 0.10418400

C -2.29539800 -2.07494800 0.06815000

C -1.93171500 -0.72293600 0.04397800

C -2.86804600 0.33658500 0.03422500

C -4.21204500 0.04423300 0.06847200

H 0.88854200 -3.28102900 0.01088100

H 2.38546200 -1.96838800 -1.71074200

H 4.77171400 -1.36035200 -1.66779900

H 4.09090800 1.06427400 1.80036500

H 1.70490500 0.45276800 1.75753500

H 6.53768700 0.17614500 -0.87351000

H 6.23649400 1.26219100 0.48698100

H 6.62323800 -0.43092000 0.77816200

H -2.02869200 4.80379000 -0.24287600

H 0.41494500 4.40282100 -0.36444000

H 1.29223300 2.06416000 -0.25090800

H -1.42655900 -4.07234900 0.05726700

H -5.66539700 -1.55347500 0.13338100

H -4.03144800 -3.38239300 0.12598600

H -4.95561600 0.83339300 0.06228600

0 1

C 1.41271000 2.86370300 -1.14968200

C 1.65521300 1.74531000 -0.30397300

C 2.93514800 1.00868600 -0.46910000

C 2.94888900 -0.36034200 -0.76473700

C 4.14658500 -1.03908500 -0.93567800

C 5.37643900 -0.38102000 -0.81643600

C 5.36049500 0.98401000 -0.52800000

C 4.16003500 1.67106600 -0.36261300

C 6.67082400 -1.12767100 -1.01353500

N -0.12004700 -2.05170000 1.91282500

C 0.89886000 -2.26199500 2.76246900

C 1.90395000 -1.33191800 2.98392300

C 1.85461900 -0.12778500 2.28444600

C 0.80566900 0.10350900 1.40144200

C -0.18478200 -0.89856200 1.25496500

C 0.20627500 3.50804400 -1.15486500

C 0.67293300 1.33520900 0.58420400

C -3.08009300 3.35815400 0.61328400

C -2.09369500 3.74035200 -0.26654400

C -0.82858700 3.11303900 -0.27035600

C -0.57822000 2.03967800 0.64147400

C -1.59774200 1.71382200 1.59235200

C -2.82013300 2.35995300 1.56459500

Pd -1.72680400 -0.35499100 0.05658200

Cl -3.54435300 0.46463100 -1.32973500

P -1.73584300 -2.42056600 -0.84561900

C -2.66774800 -2.57700900 -2.40747600

C -2.48409800 -3.63461800 0.29181600

C -0.08944500 -3.11406400 -1.24673000

H 2.18971000 3.15852100 -1.84631300

H 2.00900700 -0.89071700 -0.85725300

H 4.12915000 -2.10061900 -1.16645900

H 6.29935700 1.52046700 -0.42739500

H 4.17421600 2.73079200 -0.12828600

H 6.69202900 -2.05282300 -0.43072200

H 6.81203100 -1.40580700 -2.06309000

H 7.53096800 -0.52525400 -0.71496100

H 0.90025100 -3.21944700 3.27682700

H 2.70858100 -1.54478900 3.67846700

H 2.62988700 0.62003500 2.41218300

H 0.02167800 4.32256100 -1.84781800

H -4.05548900 3.82850500 0.57725700

H -2.28394500 4.52586000 -0.99076000

H -1.37865600 1.02606300 2.39868700

H -3.58179600 2.08885500 2.28650800

H -2.62507000 -3.60774900 -2.77011400

H -3.69910500 -2.27164500 -2.24243600

H -2.24694000 -1.90164100 -3.15182700

H -3.52880200 -3.36990900 0.45846500

H -1.94129300 -3.57696900 1.23524200

H -2.42488900 -4.64681700 -0.11671500

H -0.18961600 -4.11663900 -1.67020900

H 0.40791200 -2.46997800 -1.97294300

H 0.51117600 -3.16237600 -0.34055500

0 1

C -3.21795100 2.11347200 -1.45449800

C -3.16129200 0.79106500 -0.98157200

C -4.35038600 0.17795700 -0.34691300

C -4.98844900 0.79501400 0.73397100

C -6.10770800 0.21856700 1.32665900

C -6.63321000 -0.98745700 0.85778800

C -5.99765300 -1.59985500 -0.22772300

C -4.87827900 -1.02939300 -0.82067900

C -7.86362100 -1.59710100 1.47902600

N 0.67858700 -2.39282700 -0.87753100

C 0.14251300 -3.35762400 -0.11150500

C -1.14644600 -3.30244400 0.40875500

C -1.96199700 -2.20841000 0.11727300

C -1.43059200 -1.20107300 -0.68246300

C -0.08918300 -1.34241700 -1.12585400

C -2.11473600 2.76261000 -2.00981800

C -1.94325700 0.10341000 -1.14848500

C 1.54319500 2.02323900 -2.13869200

C 0.40250900 2.69237500 -2.42981900

C -0.89538500 2.08561800 -2.15472800

C -0.88507700 0.74360500 -1.79505800

C 0.31325400 -0.14067100 -1.95510500

C 1.62287000 0.61192700 -1.70180900

Pd 2.33492100 0.34603400 0.28463600

H 0.32757100 -0.47545200 -3.00858800

H 2.41980500 0.14243400 -2.26763000

Cl 3.33582000 -0.15488400 2.53315100

P 3.91244800 -1.24381800 -0.46740600

C 3.85497600 -2.83262200 0.43903500

C 3.93260000 -1.80585500 -2.21729100

C 5.62669600 -0.64981300 -0.19253500

H -4.15545200 2.65127000 -1.35887200

H -4.58784100 1.72421700 1.12585100

H -6.57613200 0.71213700 2.17315100

H -6.38874600 -2.53537100 -0.61738200

H -4.40543900 -1.51898600 -1.66541600

H -8.00218500 -1.26062800 2.50868200

H -8.76489900 -1.32041600 0.92141500

H -7.81143200 -2.68853900 1.48633500

H 0.78045300 -4.21158400 0.09808900

H -1.50924300 -4.11010600 1.03437600

H -2.96939200 -2.14921800 0.50844500

H -2.19154500 3.81071900 -2.28299300

H 2.49298600 2.53982100 -2.24896000

H 0.44060300 3.72703700 -2.75783700

H 4.70245100 -3.46533100 0.16228600

H 3.87313600 -2.62934200 1.50797200

H 2.92520300 -3.33821200 0.18754600

H 2.95314700 -2.22036100 -2.45771000

H 4.69337500 -2.57719400 -2.35903200

H 4.14990700 -0.97959100 -2.89610100

H 5.80602900 0.24569500 -0.79012500

H 5.72996800 -0.38760200 0.86023700

H 6.36373000 -1.41224200 -0.45842200

P 0.85494800 1.75642600 1.49335500

C -0.09346500 0.70373200 2.66051800

C -0.44835700 2.89190700 0.88189800

C 1.81461700 2.89208400 2.57311400

H -0.76481100 0.05734700 2.09206900

H -0.68153000 1.31186500 3.35309100

H 0.60512100 0.07930000 3.21544300

H -1.28594300 2.32812900 0.48091200

H -0.80329700 3.51099500 1.71002000

H -0.05776200 3.53707000 0.09692300

H 1.15884400 3.38374900 3.29674900

H 2.58774600 2.32931500 3.09153200

H 2.29212600 3.65413700 1.95370200

Transition state, imaginary frequency = 365 cm^-1^ (corresponds to concerted Pd-C, Pd-C and H-Cl vibration)

0 1

C 0.14407000 -2.74931300 -1.60723900

C -0.23793600 -1.97560300 -0.48273100

C -1.67843400 -1.65853500 -0.28530100

C -2.60108400 -2.68060000 -0.05797800

C -3.94534600 -2.38593300 0.15159000

C -4.40428500 -1.06641600 0.13184600

C -3.47435500 -0.05428500 -0.12052900

C -2.11624400 -0.31875900 -0.33670500

C -5.85323500 -0.74498700 0.39691800

N -0.57785300 1.27211700 2.54386900

C -0.35643500 0.78198300 3.77349900

C 0.21762100 -0.46171800 3.99674100

C 0.58266500 -1.22786400 2.89033800

C 0.35437100 -0.73719300 1.60692600

C -0.24411100 0.53261100 1.50148000

C 1.46186700 -3.00008800 -1.88569400

C 0.73580200 -1.50380600 0.38964200

C 4.83821300 -2.14606000 -0.55288400

C 3.85709700 -2.67861900 -1.35154800

C 2.48434900 -2.48476600 -1.05348900

C 2.11891300 -1.73251000 0.10800200

C 3.16231800 -1.19486400 0.91059400

C 4.48332400 -1.39245300 0.58812400

H -1.86460100 0.39796400 -1.83972900

Cl -1.53178100 1.27126600 -2.94476500

Pd -0.59911700 1.20941800 -0.33319600

P 1.27385700 2.61108400 -0.27254700

C 1.02904100 4.31116000 -0.92434100

C 2.06456900 2.88021200 1.35795600

C 2.63573400 1.94257800 -1.30648900

H -0.63430600 -3.11766100 -2.26649300

H -2.26339800 -3.71229700 -0.02444900

H -4.64726700 -3.19464200 0.33539100

H -3.82194900 0.97464300 -0.16674500

H -6.14017000 0.20688300 -0.05520300

H -6.04922500 -0.66706500 1.47183700

H -6.51654100 -1.51858400 0.00186600

H -0.65228700 1.41981500 4.60208700

H 0.37470300 -0.82376000 5.00653500

H 1.03820400 -2.20489000 3.01860600

H 1.73759400 -3.57880700 -2.76183400

H 5.88444600 -2.29830400 -0.79593800

H 4.11968600 -3.25388200 -2.23421700

H 2.90525900 -0.61263600 1.78628800

H 5.26072600 -0.96482700 1.21285800

H 0.61255600 4.25321800 -1.93123100

H 0.31391400 4.84107200 -0.29316700

H 1.96610700 4.87337400 -0.95508900

H 2.43010900 1.92532100 1.73726300

H 2.89775300 3.58356000 1.28970700

H 1.32003400 3.25071500 2.06246300

H 2.92787400 0.96271500 -0.92653100

H 2.27971500 1.81409900 -2.32976400

H 3.50692400 2.60311100 -1.30773100

0 1

C -0.56105900 2.61633400 -1.80084400

C -0.26542000 2.05608600 -0.53091400

C 1.14363500 2.06961900 -0.06371700

C 1.87506800 3.26476200 -0.05042800

C 3.19972600 3.28180800 0.36866200

C 3.82545400 2.10272500 0.78613100

C 3.09212400 0.91311000 0.74701400

C 1.76481100 0.87018900 0.31635800

C 5.24668400 2.12202600 1.29073500

N 0.05909800 -0.62674800 3.02797400

C -0.54586700 -0.05760000 4.08106600

C -1.40089900 1.03004200 3.97335100

C -1.64890600 1.54343500 2.70170400

C -1.03048900 0.96609500 1.59521400

C -0.15940000 -0.12662600 1.80710000

C -1.82305100 2.57355600 -2.33203200

C -1.28970800 1.48728500 0.22282400

Pd 0.73562300 -0.91059800 0.13996000

C -5.19938100 1.25232100 -1.46864300

C -4.18580600 1.86508700 -2.16169800

C -2.87903100 1.95832300 -1.61899800

C -2.61258200 1.41313700 -0.32140100

C -3.68785600 0.78104300 0.36222100

C -4.94170200 0.70102100 -0.19378700

P -0.57015500 -2.85953600 0.45606000

C -0.77432500 -4.16993500 -0.82713400

C -2.31259400 -2.53416500 0.93912000

C 0.08230300 -3.79248100 1.89429200

P 1.64044900 -1.21692500 -2.03634300

C 2.32724500 -2.81214300 -2.66036900

C 2.94292700 -0.02899000 -2.55442600

C 0.29615500 -0.86630800 -3.24398700

H 0.25002500 3.06889400 -2.36191800

H 1.39393600 4.19145700 -0.35121400

H 3.74887200 4.21923500 0.38407400

H 3.57873100 -0.00825500 1.05823900

H 5.86476300 2.82948000 0.73098100

H 5.28772900 2.42066800 2.34401500

H 5.71192100 1.13617300 1.21614400

H -0.32936200 -0.49988300 5.05099900

H -1.85842300 1.46496900 4.85523200

H -2.31424900 2.39145800 2.56924200

H -2.02423700 2.99872900 -3.31089000

H -6.19462500 1.18708100 -1.89586600

H -4.36974400 2.28895800 -3.14462200

H -3.50683300 0.34823600 1.33653200

H -5.74088900 0.20774600 0.35010600

H -1.21653600 -3.74296900 -1.72950600

H 0.20085800 -4.58469600 -1.08658500

H -1.41508500 -4.98270600 -0.47523800

H -2.85709100 -3.46209100 1.13285000

H -2.81934900 -1.97272000 0.15344400

H -2.31377700 -1.92188300 1.84046400

H 0.21771700 -3.07748400 2.70817000

H -0.58887500 -4.59774000 2.20484700

H 1.05797200 -4.21334200 1.64384400

H 3.18748500 -3.10122400 -2.05356500

H 2.64269100 -2.73718000 -3.70455100

H 1.57544200 -3.59746700 -2.57801700

H 3.83889500 -0.17875000 -1.95114300

H 3.19230800 -0.14474100 -3.61249900

H 2.59700100 0.98698800 -2.36542700

H 0.65504400 -0.88458600 -4.27674200

H -0.49991100 -1.60410600 -3.13040400

H -0.12751800 0.11487800 -3.02519900

# 4 References

[S1] G. R. Fulmer, A. J. M. Miller, N. H. Sherden, H. E. Gottlieb, A. Nudelman, B. M. Stoltz, J. E. Bercaw, K. I. Goldberg,"NMR Chemical Shifts of Trace Impurities: Common Laboratory Solvents, Organics, and Gases in Deuterated Solvents Relevant to the Organometallic Chemist", *Organometallics* **2010**, *29*, 2176–2179.

[S2] M. J. Frisch, G. W. Trucks, H. B. Schlegel, G. E. Scuseria, M. A. Robb, J. R. Cheeseman, G. Scalmani, V. Barone, G. A. Petersson, H. Nakatsuji, X. Li, M. Caricato, A. V. Marenich, J. Bloino, B. G. Janesko, R. Gomperts, B. Mennucci, H. P. Hratchian, J. V. Ortiz, A. F. Izmaylov, J. L. Sonnenberg, D. Williams-Young, F. Ding, F. Lipparini, F. Egidi, J. Goings, B. Peng, A. Petrone, T. Henderson, D. Ranasinghe, V. G. Zakrzewski, J. Gao, N. Rega, G. Zheng, W. Liang, M. Hada, M. Ehara, K. Toyota, R. Fukuda, J. Hasegawa, M. Ishida, T. Nakajima, Y. Honda, O. Kitao, H. Nakai, T. Vreven, K. Throssell, J. J. A. Montgomery, J. E. Peralta, F. Ogliaro, M. J. Bearpark, J. J. Heyd, E. N. Brothers, K. N. Kudin, V. N. Staroverov, T. A. Keith, R. Kobayashi, J. Normand, K. Raghavachari, A. P. Rendell, J. C. Burant, S. S. Iyengar, J. Tomasi, M. Cossi, J. M. Millam, M. Klene, C. Adamo, R. Cammi, J. W. Ochterski, R. L. Martin, K. Morokuma, O. Farkas, J. B. Foresman, D. J. Fox, *Gaussian 16, Revision C.01*.

[S3] P. Hohenberg, W. Kohn,"Inhomogeneous Electron Gas", *Phys. Rev.* **1964**, *136*, B864-B871.

[S4] W. Kohn, L. J. Sham,"Self-Consistent Equations Including Exchange and Correlation Effects", *Phys. Rev.* **1965**, *140*, A1133-A1138.

[S5] R. G. Parr, W. Yang, *Density-functional theory of atoms and molecules*, Oxford Univ. Press, Oxford, **1989**.

[S6] M. C. Z. Dennis R. Salahub, *The Challenge of d and f Electrons- Theory and Computation, Vol. 394*, ACS, **1989**.

[S7] S. H. Vosko, L. Wilk, M. Nusair,"*Can. J. Phys.* **1980**, *58*, 1200-1211.

[S8] C. Lee, W. Yang, R. G. Parr,"Development of the Colle-Salvetti correlation-energy formula into a functional of the electron density", *Phys. Rev. B: Condens. Matter Mater. Phys.* **1988**, *37*, 785–789.

[S9] A. D. Becke,"Density‐functional thermochemistry. III. The role of exact exchange", *J. Chem. Phys.* **1993**, *98*, 5648–5652.

[S10] P. J. Stephens, F. J. Devlin, C. F. Chabalowski, M. J. Frisch,"Ab Initio Calculation of Vibrational Absorption and Circular Dichroism Spectra Using Density Functional Force Fields", *J. Phys. Chem.* **1994**, *98*, 11623–11627.

[S11] A. D. McLean, G. S. Chandler,"Contracted Gaussian basis sets for molecular calculations. I. Second row atoms, Z=11–18", *J. Chem. Phys.* **1980**, *72*, 5639-5648.

[S12] R. Krishnan, J. S. Binkley, R. Seeger, J. A. Pople,"Self‐consistent molecular orbital methods. XX. A basis set for correlated wave functions", *J. Chem. Phys.* **1980**, *72*, 650-654.

[S13] M. D. Hanwell, D. E. Curtis, D. C. Lonie, T. Vandermeersch, E. Zurek, G. R. Hutchison,"Avogadro: an advanced semantic chemical editor, visualization, and analysis platform", *J. Cheminformatics* **2012**, *4*, 1–17.

[S14] R. Bauernschmitt, R. Ahlrichs,"Treatment of electronic excitations within the adiabatic approximation of time dependent density functional theory", *Chem. Phys. Lett.* **1996**, *256*, 454-464.

[S15] M. E. Casida, C. Jamorski, K. C. Casida, D. R. Salahub,"Molecular excitation energies to high-lying bound states from time-dependent density-functional response theory: Characterization and correction of the time-dependent local density approximation ionization threshold", *J. Chem. Phys.* **1998**, *108*, 4439-4449.

[S16] R. E. Stratmann, G. E. Scuseria, M. J. Frisch,"An efficient implementation of time-dependent density-functional theory for the calculation of excitation energies of large molecules", *J. Chem. Phys.* **1998**, *109*, 8218-8224.

[S17] C. Van Caillie, R. D. Amos,"Geometric derivatives of excitation energies using SCF and DFT", *Chem. Phys. Lett.* **1999**, *308*, 249-255.

[S18] C. Van Caillie, R. D. Amos,"Geometric derivatives of density functional theory excitation energies using gradient-corrected functionals", *Chem. Phys. Lett.* **2000**, *317*, 159-164.

[S19] F. Furche, R. Ahlrichs,"Adiabatic time-dependent density functional methods for excited state properties", *J. Chem. Phys.* **2002**, *117*, 7433-7447.

[S20] G. Scalmani, M. J. Frisch, B. Mennucci, J. Tomasi, R. Cammi, V. Barone,"Geometries and properties of excited states in the gas phase and in solution: Theory and application of a time-dependent density functional theory polarizable continuum model", *J. Chem. Phys.* **2006**, *124*, 094107.

[S21] N. M. O'Boyle, A. L. Tenderholt, K. M. Langner,"cclib: A library for package-independent computational chemistry algorithms", *J. Comp. Chem,* **2008**, *29*, 839–845.

[S22] C. C. J. Roothaan,"New Developments in Molecular Orbital Theory", *Rev. Mod. Phys.* **1951**, *23*, 69-89.

[S23] F. London,"The quantic theory of inter-atomic currents in aromatic combinations", *J. Phys. Radium* **1937**, *8*, 397–409.

[S24] R. McWeeny,"Perturbation Theory for the Fock-Dirac Density Matrix", *Physical Review* **1962**, *126*, 1028-1034.

[S25] R. Ditchfield,"Self-consistent perturbation theory of diamagnetism", *Mol. Phys.* **1974**, *27*, 789-807.

[S26] K. Wolinski, J. F. Hinton, P. Pulay,"Efficient implementation of the gauge-independent atomic orbital method for NMR chemical shift calculations", *J. Am. Chem. Soc.* **1990**, *112*, 8251-8260.

[S27] J. R. Cheeseman, G. W. Trucks, T. A. Keith, M. J. Frisch,"A comparison of models for calculating nuclear magnetic resonance shielding tensors", *J. Chem. Phys.* **1996**, *104*, 5497-5509.

[S28] OpenAI, ChatGPT (Version GPT-4), OpenAI, San Francisco, USA.

[S29] L. Krause, R. Herbst-Irmer, G. M. Sheldrick., D. Stalke.,"Comparison of silver and molybdenum microfocus X-ray sources for single-crystal structure determination", *J. Appl. Crystallogr.* **2015**, *48*, 3-10.

[S30] G. M. Sheldrick,"Crystal structure solution with ShelXT", *Acta Cryst.* **2015**, *A71*, 3-8.

[S31] G. M. Sheldrick,"Crystal structure solution with ShelXT", *Acta Cryst.* **2015**, *C71*, 3-8.

[S32] K. Takai, Y. Inoue, Y. Konishi, A. Suwa, Y. Uruno, H. Matsuda, T. Nakako, M. Sakai, H. Nishikawa, G. Hashimoto, T. Enomoto, A. Kitamura, Y. Uematsu, A. Kiyoshi, T. Sumiyoshi,"Discovery of N-substituted 7-azaindoline derivatives as potent, orally available M1 and M4 muscarinic acetylcholine receptors selective agonists", *Bioorg. Med. Chem. Lett.* **2014**, *24*, 3189-3193.

[S33] W. D. Cornell, P. Cieplak, C. I. Bayly, I. R. Gould, K. M. Merz, D. M. Ferguson, D. C. Spellmeyer, T. Fox, J. W. Caldwell, P. A. Kollman,"A Second Generation Force Field for the Simulation of Proteins, Nucleic Acids, and Organic Molecules", *J. Am. Chem. Soc.* **1995**, *117*, 5179-5197.

[S34] D. Porezag, T. Frauenheim, T. Köhler, G. Seifert, R. Kaschner,"Construction of tight-binding-like potentials on the basis of density-functional theory: Application to carbon", *Phys. Rev. B* **1995**, *51*, 12947-12957.

[S35] M. Elstner, D. Porezag, G. Jungnickel, J. Elsner, M. Haugk, T. Frauenheim, S. Suhai, G. Seifert,"Self-consistent-charge density-functional tight-binding method for simulations of complex materials properties", *Phys. Rev. B* **1998**, *58*, 7260-7268.
