# Supplementary material for: Synthesis of Aza‐indeno‐aza‐fluoranthene and Constitutional Isomers by Ring‐Size Selective C–H Activation
Source: Chemistry. 2025 Nov 11;31(72):e02960. doi: 10.1002/chem.202502960 (PMC12731527; doi:10.1002/chem.202502960)

checkCIF/PLATON report

You have not supplied any structure factors. As a result the full set of tests cannot be run.

THIS REPORT IS FOR GUIDANCE ONLY. IF USED AS PART OF A REVIEW PROCEDURE FOR PUBLICATION, IT SHOULD NOT REPLACE THE EXPERTISE OF AN EXPERIENCED CRYSTALLOGRAPHIC REFEREE.

No syntax errors found. CIF dictionary Interpreting this report

Datablock: keck18

```
Bond precision:   C-C = 0.0025 A          Wavelength=0.71073

Cell:            a=8.3401(9)    b=23.432(3)            c=12.4217(13)
                 alpha=90       beta=105.2759(16)      gamma=90
Temperature:     200 K

                 Calculated                  Reported
Volume           2341.8(5)                   2341.8(4)
Space group      P 21/c                      P 21/c
Hall group       -P 2ybc                     -P 2ybc
Moiety formula   C34 H22 N2                  ?
Sum formula      C34 H22 N2                  C34 H22 N2
Mr               458.54                      458.53
Dx,g cm-3        1.301                       1.301
Z                4                           4
Mu (mm-1)        0.076                       0.076
F000             960.0                       960.0
F000'            960.34
h,k,lmax         11,32,17                    11,32,17
Nref             6481                        6305
Tmin,Tmax        0.990,0.998                 0.921,0.960
Tmin'            0.990

Correction method= # Reported T Limits: Tmin=0.921 Tmax=0.960
AbsCorr = MULTI-SCAN

Data completeness= 0.973          Theta(max)= 29.412

                                                        wR2(reflections)=
R(reflections)= 0.0565( 3748)                           0.1425( 6305)

S = 1.015                 Npar= 327
```

The following ALERTS were generated. Each ALERT has the format
test-name_ALERT_alert-type_alert-level.
Click on the hyperlinks for more details of the test.

Alert level C

ABSTY02_ALERT_1_C An _exptl_absorpt_correction_type has been given without
a literature citation. This should be contained in the
_exptl_absorpt_process_details field.
Absorption correction given as multi-scan
PLAT767_ALERT_4_C INS Embedded LIST 6 Instruction Should be LIST 4 Please Check

Alert level G

PLAT883_ALERT_1_G No Info/Value for _atom_sites_solution_primary . Please Do !
PLAT941_ALERT_3_G Average HKL Measurement Multiplicity .... 4.5 Low

0 **ALERT level A** = Most likely a serious problem - resolve or explain
0 **ALERT level B** = A potentially serious problem, consider carefully
2 **ALERT level C** = Check. Ensure it is not caused by an omission or oversight
2 **ALERT level G** = General information/check it is not something unexpected

2 ALERT type 1 CIF construction/syntax error, inconsistent or missing data
0 ALERT type 2 Indicator that the structure model may be wrong or deficient
1 ALERT type 3 Indicator that the structure quality may be low
1 ALERT type 4 Improvement, methodology, query or suggestion
0 ALERT type 5 Informative message, check

It is advisable to attempt to resolve as many as possible of the alerts in all categories. Often the minor alerts point to easily fixed oversights, errors and omissions in your CIF or refinement strategy, so attention to these fine details can be worthwhile. In order to resolve some of the more serious problems it may be necessary to carry out additional measurements or structure refinements. However, the purpose of your study may justify the reported deviations and the more serious of these should normally be commented upon in the discussion or experimental section of a paper or in the "special_details" fields of the CIF. checkCIF was carefully designed to identify outliers and unusual parameters, but every test has its limitations and alerts that are not important in a particular case may appear. Conversely, the absence of alerts does not guarantee there are no aspects of the results needing attention. It is up to the individual to critically assess their own results and, if necessary, seek expert advice.

Publication of your CIF in IUCr journals

A basic structural check has been run on your CIF. These basic checks will be run on all CIFs submitted for publication in IUCr journals (*Acta Crystallographica*, *Journal of Applied Crystallography*, *Journal of Synchrotron Radiation*); however, if you intend to submit to *Acta Crystallographica Section C* or *E* or *IUCrData*, you should make sure that full publication checks are run on the final version of your CIF prior to submission.

Publication of your CIF in other journals

Please refer to the *Notes for Authors* of the relevant journal for any special instructions relating to CIF submission.

PLATON version of 06/01/2024; check.def file version of 05/01/2024

Datablock keck18 - ellipsoid plot

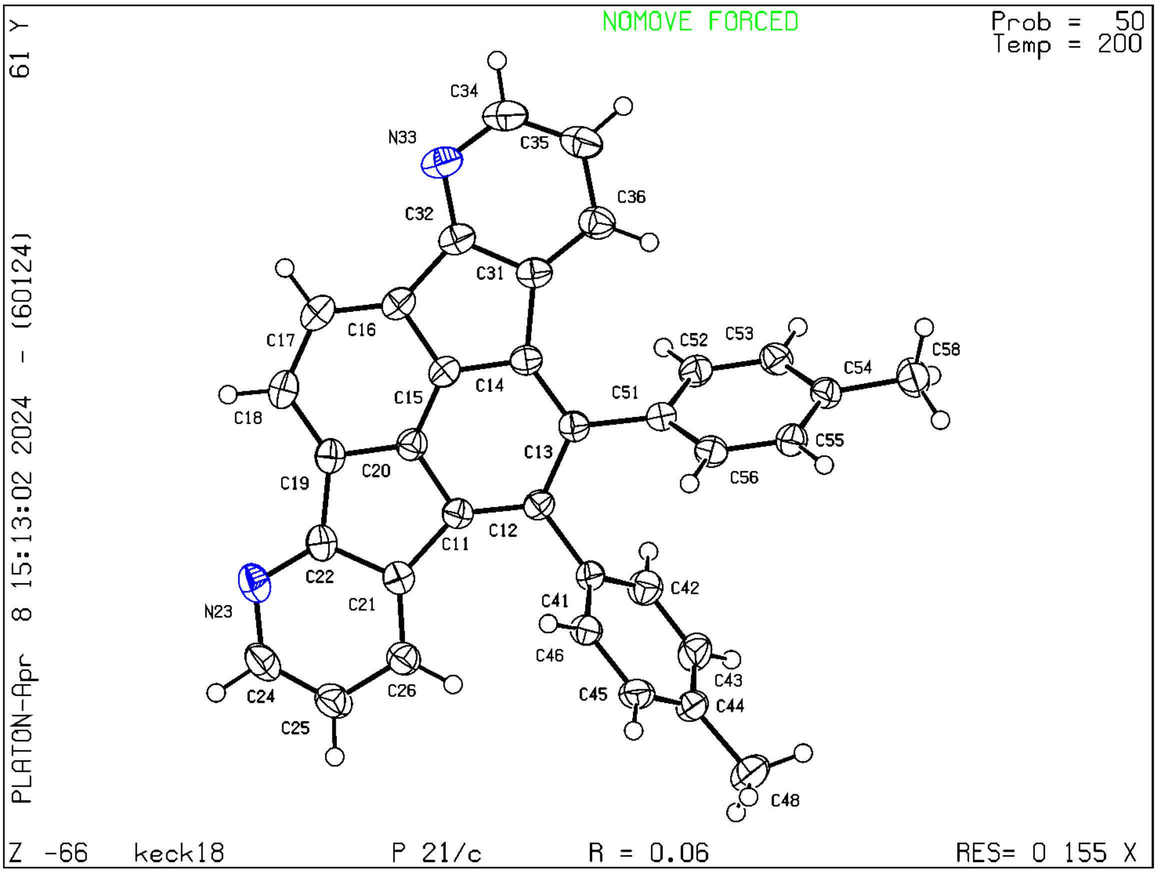

Supplement: Supplementary file 2 — Supporting Information [file CHEM-31-e02960-s002.zip › CCDC_2486400_checkcif.pdf]
