# Supplementary material for: Synthesis of Aza‐indeno‐aza‐fluoranthene and Constitutional Isomers by Ring‐Size Selective C–H Activation
Source: Chemistry. 2025 Nov 11;31(72):e02960. doi: 10.1002/chem.202502960 (PMC12731527; doi:10.1002/chem.202502960)

checkCIF/PLATON report

You have not supplied any structure factors. As a result the full set of tests cannot be run.

THIS REPORT IS FOR GUIDANCE ONLY. IF USED AS PART OF A REVIEW PROCEDURE FOR PUBLICATION, IT SHOULD NOT REPLACE THE EXPERTISE OF AN EXPERIENCED CRYSTALLOGRAPHIC REFEREE.

No syntax errors found.   CIF dictionary   Interpreting this report

Datablock: keck20

Bond precision:	C-C = 0.0030 A		Wavelength=1.54178
Cell:	a=15.7179(6)	b=20.1560(9)	c=7.6636(3)
	alpha=90	beta=113.063(3)	gamma=90
Temperature:	200 K		

	Calculated	Reported
Volume	2233.85(17)	2233.86(16)
Space group	C 2/c	C 2/c
Hall group	-C 2yc	-C 2yc
Moiety formula	C34 H22 N2	?
Sum formula	C34 H22 N2	C34 H22 N2
Mr	458.54	458.53
Dx,g cm-3	1.363	1.363
Z	4	4
Mu (mm-1)	0.612	0.612
F000	960.0	960.0
F000'	962.56	
h,k,lmax	18,24,9	18,24,9
Nref	2059	2017
Tmin,Tmax	0.980,0.985	0.858,0.991
Tmin'	0.964	

Correction method= # Reported T Limits: Tmin=0.858 Tmax=0.991
AbsCorr = MULTI-SCAN

Data completeness= 0.980   Theta(max)= 68.499

R(reflections)= 0.0419(1269)   wR2(reflections)= 0.1115(2017)

S = 1.024   Npar= 164

The following ALERTS were generated. Each ALERT has the format
test-name_ALERT_alert-type_alert-level.
Click on the hyperlinks for more details of the test.

Alert level C

```
ABSTY02_ALERT_1_C  An _exptl_absorpt_correction_type has been given without
            a literature citation. This should be contained in the
            _exptl_absorpt_process_details field.
            Absorption correction given as multi-scan
```

Alert level G

```
PLAT128_ALERT_4_G Alternate Setting for Input Space Group     C2/c       I2/a Note
PLAT883_ALERT_1_G No Info/Value for _atom_sites_solution_primary .     Please Do !
```

0 **ALERT level A** = Most likely a serious problem - resolve or explain
0 **ALERT level B** = A potentially serious problem, consider carefully
1 **ALERT level C** = Check. Ensure it is not caused by an omission or oversight
2 **ALERT level G** = General information/check it is not something unexpected

2 ALERT type 1 CIF construction/syntax error, inconsistent or missing data
0 ALERT type 2 Indicator that the structure model may be wrong or deficient
0 ALERT type 3 Indicator that the structure quality may be low
1 ALERT type 4 Improvement, methodology, query or suggestion
0 ALERT type 5 Informative message, check

It is advisable to attempt to resolve as many as possible of the alerts in all categories. Often the minor alerts point to easily fixed oversights, errors and omissions in your CIF or refinement strategy, so attention to these fine details can be worthwhile. In order to resolve some of the more serious problems it may be necessary to carry out additional measurements or structure refinements. However, the purpose of your study may justify the reported deviations and the more serious of these should normally be commented upon in the discussion or experimental section of a paper or in the "special_details" fields of the CIF. checkCIF was carefully designed to identify outliers and unusual parameters, but every test has its limitations and alerts that are not important in a particular case may appear. Conversely, the absence of alerts does not guarantee there are no aspects of the results needing attention. It is up to the individual to critically assess their own results and, if necessary, seek expert advice.

Publication of your CIF in IUCr journals

A basic structural check has been run on your CIF. These basic checks will be run on all CIFs submitted for publication in IUCr journals (*Acta Crystallographica*, *Journal of Applied Crystallography*, *Journal of Synchrotron Radiation*); however, if you intend to submit to *Acta Crystallographica Section C* or *E* or *IUCrData*, you should make sure that full publication checks are run on the final version of your CIF prior to submission.

Publication of your CIF in other journals

Please refer to the *Notes for Authors* of the relevant journal for any special instructions relating to CIF submission.

PLATON version of 06/01/2024; check.def file version of 05/01/2024

Datablock keck20 - ellipsoid plot

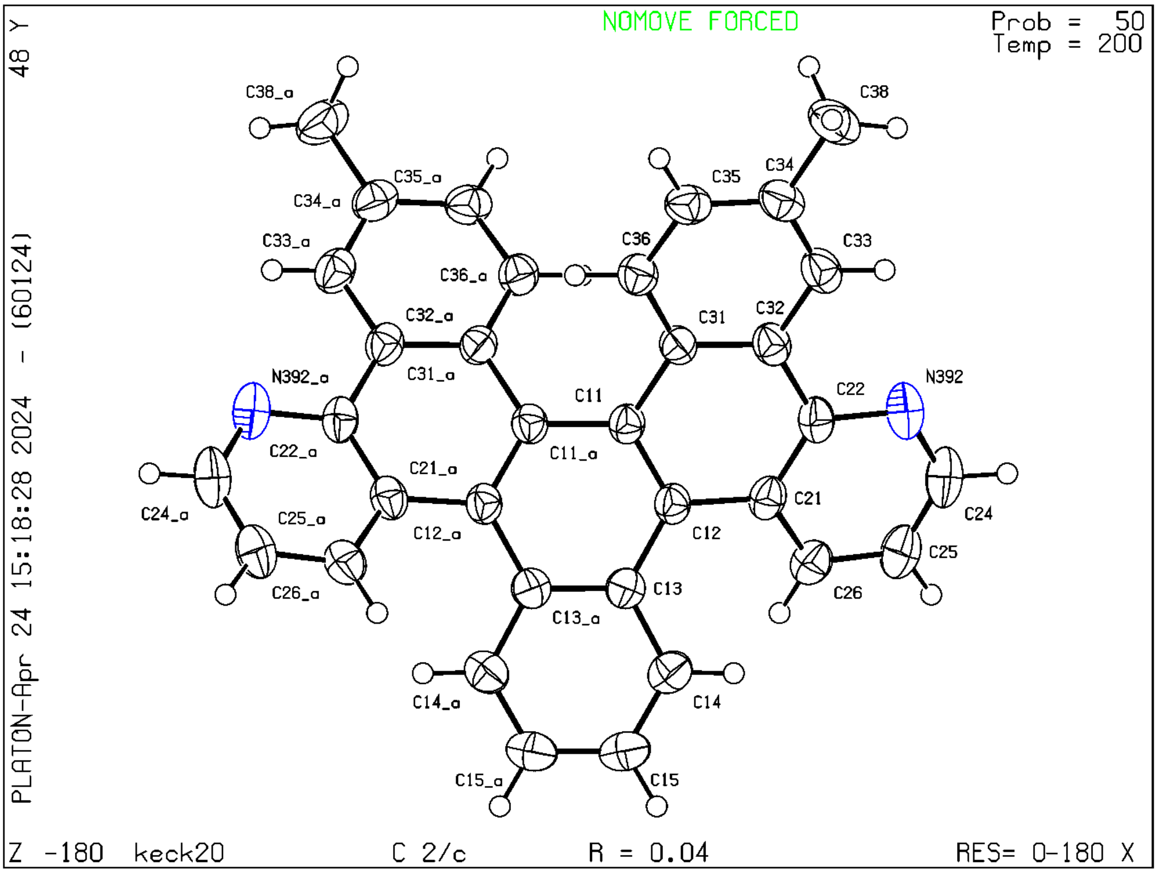

Supplement: Supplementary file 2 — Supporting Information [file CHEM-31-e02960-s002.zip › CCDC_2486402_checkcif.pdf]
