# Supplementary material for: Synthesis of Aza‐indeno‐aza‐fluoranthene and Constitutional Isomers by Ring‐Size Selective C–H Activation
Source: Chemistry. 2025 Nov 11;31(72):e02960. doi: 10.1002/chem.202502960 (PMC12731527; doi:10.1002/chem.202502960)

checkCIF/PLATON report

You have not supplied any structure factors. As a result the full set of tests cannot be run.

THIS REPORT IS FOR GUIDANCE ONLY. IF USED AS PART OF A REVIEW PROCEDURE FOR PUBLICATION, IT SHOULD NOT REPLACE THE EXPERTISE OF AN EXPERIENCED CRYSTALLOGRAPHIC REFEREE.

No syntax errors found.   CIF dictionary   Interpreting this report

Datablock: keck17

```
Bond precision:   C-C = 0.0095 A          Wavelength=1.54178

Cell:             a=12.3695(5)     b=11.2492(3)       c=19.6897(8)
                  alpha=90         beta=94.066(3)     gamma=90
Temperature:      200 K
```

	Calculated	Reported
Volume	2732.87(17)	2732.87(17)
Space group	P 21/c	P 21/c
Hall group	-P 2ybc	-P 2ybc
Moiety formula	C34 H24 Cl2 N2	?
Sum formula	C34 H24 Cl2 N2	C34 H24 Cl2 N2
Mr	531.45	531.45
Dx,g cm-3	1.292	1.292
Z	4	4
Mu (mm-1)	2.327	2.327
F000	1104.0	1104.0
F000'	1109.49	
h,k,lmax	14,12,22	14,12,22
Nref	4246	4161
Tmin,Tmax	0.920,0.968	0.887,0.950
Tmin'	0.876	

```
Correction method= # Reported T Limits: Tmin=0.887 Tmax=0.950
AbsCorr = MULTI-SCAN

Data completeness= 0.980           Theta(max)= 61.624

R(reflections)= 0.0843( 2409)                          wR2(reflections)=
                                                       0.1978( 4161)
S = 1.168                  Npar= 345
```

The following ALERTS were generated. Each ALERT has the format
test-name_ALERT_alert-type_alert-level.
Click on the hyperlinks for more details of the test.

Alert level B

```
THETM01_ALERT_3_B  The value of sine(theta_max)/wavelength is less than 0.575
            Calculated sin(theta_max)/wavelength =    0.5707
```

Alert level C

```
ABSTY02_ALERT_1_C  An _exptl_absorpt_correction_type has been given without
            a literature citation. This should be contained in the
            _exptl_absorpt_process_details field.
            Absorption correction given as multi-scan
PLAT340_ALERT_3_C Low Bond Precision on  C-C Bonds ...............    0.00949 Ang.
```

Alert level G

```
PLAT083_ALERT_2_G SHELXL Second Parameter in WGHT  Unusually Large      10.34 Why ?
PLAT883_ALERT_1_G No Info/Value for _atom_sites_solution_primary .     Please Do !
PLAT941_ALERT_3_G Average HKL Measurement Multiplicity ..........        4.0 Low
```

0 **ALERT level A** = Most likely a serious problem - resolve or explain
1 **ALERT level B** = A potentially serious problem, consider carefully
2 **ALERT level C** = Check. Ensure it is not caused by an omission or oversight
3 **ALERT level G** = General information/check it is not something unexpected

2 ALERT type 1 CIF construction/syntax error, inconsistent or missing data
1 ALERT type 2 Indicator that the structure model may be wrong or deficient
3 ALERT type 3 Indicator that the structure quality may be low
0 ALERT type 4 Improvement, methodology, query or suggestion
0 ALERT type 5 Informative message, check

It is advisable to attempt to resolve as many as possible of the alerts in all categories. Often the minor alerts point to easily fixed oversights, errors and omissions in your CIF or refinement strategy, so attention to these fine details can be worthwhile. In order to resolve some of the more serious problems it may be necessary to carry out additional measurements or structure refinements. However, the purpose of your study may justify the reported deviations and the more serious of these should normally be commented upon in the discussion or experimental section of a paper or in the "special_details" fields of the CIF. checkCIF was carefully designed to identify outliers and unusual parameters, but every test has its limitations and alerts that are not important in a particular case may appear. Conversely, the absence of alerts does not guarantee there are no aspects of the results needing attention. It is up to the individual to critically assess their own results and, if necessary, seek expert advice.

Publication of your CIF in IUCr journals

A basic structural check has been run on your CIF. These basic checks will be run on all CIFs submitted for publication in IUCr journals (*Acta Crystallographica*, *Journal of Applied Crystallography*, *Journal of Synchrotron Radiation*); however, if you intend to submit to *Acta Crystallographica Section C* or *E* or *IUCrData*, you should make sure that full publication checks are run on the final version of your CIF prior to submission.

Publication of your CIF in other journals

Please refer to the *Notes for Authors* of the relevant journal for any special instructions relating to CIF submission.

PLATON version of 06/01/2024; check.def file version of 05/01/2024

Datablock keck17 - ellipsoid plot

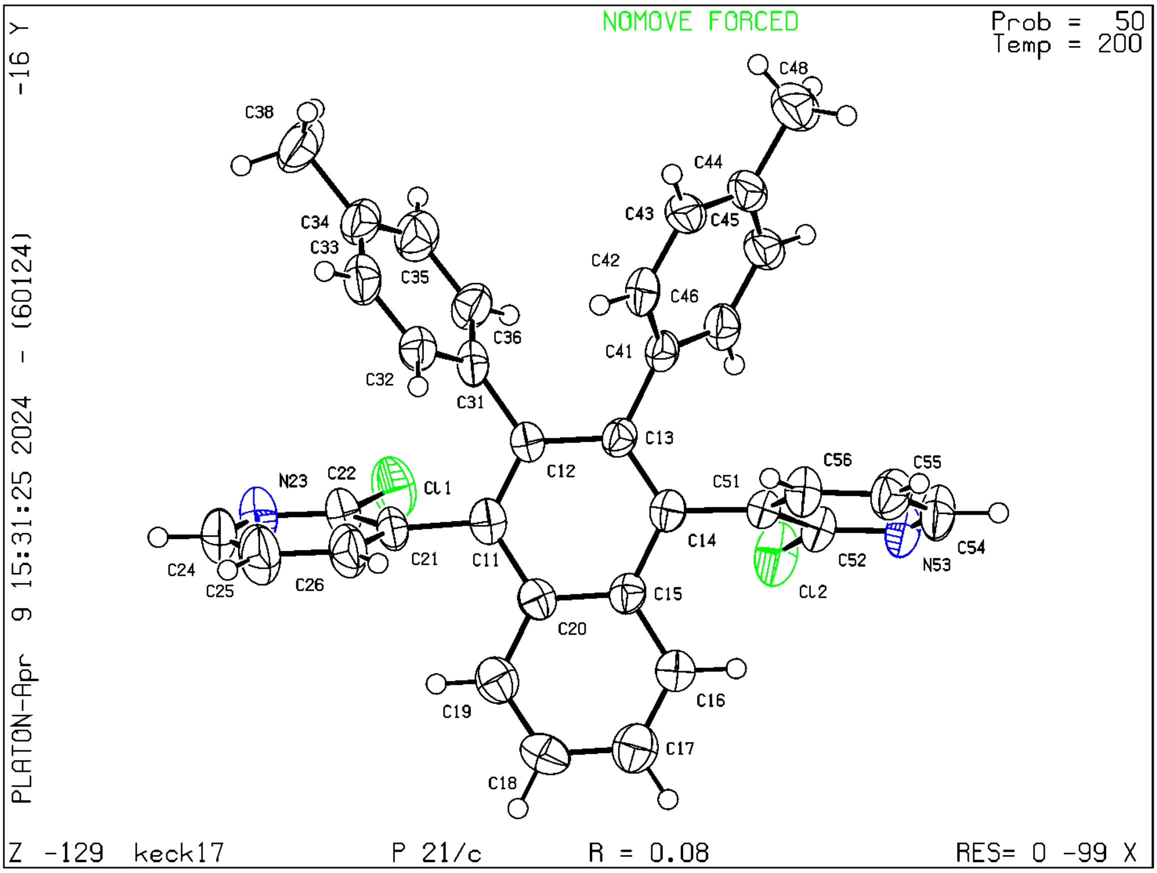

Supplement: Supplementary file 2 — Supporting Information [file CHEM-31-e02960-s002.zip › CCDC_2486399_checkcif.pdf]
