# Supplementary material for: Synthesis of Aza‐indeno‐aza‐fluoranthene and Constitutional Isomers by Ring‐Size Selective C–H Activation
Source: Chemistry. 2025 Nov 11;31(72):e02960. doi: 10.1002/chem.202502960 (PMC12731527; doi:10.1002/chem.202502960)

checkCIF/PLATON report

You have not supplied any structure factors. As a result the full set of tests cannot be run.

THIS REPORT IS FOR GUIDANCE ONLY. IF USED AS PART OF A REVIEW PROCEDURE FOR PUBLICATION, IT SHOULD NOT REPLACE THE EXPERTISE OF AN EXPERIENCED CRYSTALLOGRAPHIC REFEREE.

No syntax errors found. CIF dictionary Interpreting this report

Datablock: keck19

Bond precision:	C-C = 0.0063 A		Wavelength=0.71073
Cell:	a=10.3373(10)	b=19.608(2)	c=22.789(2)
	alpha=90	beta=96.886(3)	gamma=90
Temperature:	200 K		

	Calculated	Reported
Volume	4585.9(8)	4585.8(8)
Space group	P 21/c	P 21/c
Hall group	-P 2ybc	-P 2ybc
Moiety formula	C34 H22 N2	?
Sum formula	C34 H22 N2	C34 H22 N2
Mr	458.54	458.53
Dx,g cm-3	1.328	1.328
Z	8	8
Mu (mm-1)	0.077	0.077
F000	1920.0	1920.0
F000’	1920.68	
h,k,lmax	10,20,23	10,20,23
Nref	5370	5349
Tmin,Tmax	0.995,0.997	0.908,0.956
Tmin’	0.991	

Correction method= # Reported T Limits: Tmin=0.908 Tmax=0.956
AbsCorr = MULTI-SCAN

Data completeness= 0.996 Theta(max)= 21.644

R(reflections)= 0.0645(3679) wR2(reflections)= 0.1526(5349)

S = 1.091 Npar= 916

The following ALERTS were generated. Each ALERT has the format
test-name_ALERT_alert-type_alert-level.
Click on the hyperlinks for more details of the test.

Alert level A

```
THETM01_ALERT_3_A  The value of sine(theta_max)/wavelength is less than 0.550
            Calculated sin(theta_max)/wavelength =    0.5190
```

Author Response: We have cut the dataset at a resolution of 0.96 A due to I/sigma and R(int) criteria. Despite of using a high intnsity source and long irradiation times this was the best achievable resolution. The data to parameter ratio is weak. In order to compensate for the lack of information we used all possible local symmetry restraints (SHELX SAME command) and rigid bond restraints (SHELX RIGU command) for all atoms.

```
PLAT088_ALERT_3_A Poor Data / Parameter Ratio ...................       5.86 Note
```

Author Response: We have cut the dataset at a resolution of 0.96 A due to I/sigma and R(int) criteria. Despite of using a high intnsity source and long irradiation times this was the best achievable resolution. The data to parameter ratio is weak. In order to compensate for the lack of information we used all possible local symmetry restraints (SHELX SAME command) and rigid bond restraints (SHELX RIGU command) for all atoms.

Alert level C

```
ABSTY02_ALERT_1_C  An _exptl_absorpt_correction_type has been given without
            a literature citation. This should be contained in the
            _exptl_absorpt_process_details field.
            Absorption correction given as multi-scan
PLAT220_ALERT_2_C NonSolvent   Resd 1  C   Ueq(max)/Ueq(min) Range        3.1 Ratio
PLAT340_ALERT_3_C Low Bond Precision on  C-C Bonds ..............     0.0063 Ang.
PLAT410_ALERT_2_C Short Intra H...H Contact  H26_1    ..H35_1    .       1.94 Ang.
                                                      x,y,z  =      1_555 Check
```

Alert level G

```
PLAT002_ALERT_2_G Number of Distance or Angle Restraints on AtSite        101 Note
PLAT003_ALERT_2_G Number of Uiso or Uij Restrained non-H Atoms ...         65 Report
PLAT083_ALERT_2_G SHELXL Second Parameter in WGHT  Unusually Large       6.86 Why ?
PLAT175_ALERT_4_G The CIF-Embedded .res File Contains SAME Records          2 Report
PLAT178_ALERT_4_G The CIF-Embedded .res File Contains SIMU Records          1 Report
PLAT187_ALERT_4_G The CIF-Embedded .res File Contains RIGU Records          1 Report
PLAT301_ALERT_3_G Main Residue  Disorder .............(Resd    1)         81% Note
PLAT413_ALERT_2_G Short Inter XH3 .. XHn     H18C_1    ..H15B_2    .       2.11 Ang.
                                            -x,-1/2+y,1/2-z  =      2_545 Check
PLAT413_ALERT_2_G Short Inter XH3 .. XHn     H58B_1    ..H18F_2    .       1.95 Ang.
                                            -x,-1/2+y,1/2-z  =      2_545 Check
```

```
PLAT432_ALERT_2_G Short Inter X...Y Contact  C25_1    ..C45B_2   .       3.18 Ang.
                                              x,3/2-y,-1/2+z  =      4_575 Check
PLAT432_ALERT_2_G Short Inter X...Y Contact  C54_2    ..C24B_2   .       3.09 Ang.
                                              x,3/2-y,1/2+z  =      4_576 Check
PLAT432_ALERT_2_G Short Inter X...Y Contact  C55_2    ..C24B_2   .       3.14 Ang.
                                              x,3/2-y,1/2+z  =      4_576 Check
PLAT722_ALERT_1_G Angle   Calc      120.00, Rep      118.90 Dev...       1.10 Degree
              C25B_2  -C26B_2  -H26B_2     1_555  1_555  1_555     #  273 Check
PLAT811_ALERT_5_G No ADDSYM Analysis: Too Many Excluded Atoms ....          ! Info
PLAT860_ALERT_3_G Number of Least-Squares Restraints ............       2427 Note
PLAT883_ALERT_1_G No Info/Value for _atom_sites_solution_primary .     Please Do !
```

```
   2 ALERT level A = Most likely a serious problem - resolve or explain
   0 ALERT level B = A potentially serious problem, consider carefully
   4 ALERT level C = Check. Ensure it is not caused by an omission or oversight
  16 ALERT level G = General information/check it is not something unexpected

   3 ALERT type 1 CIF construction/syntax error, inconsistent or missing data
  10 ALERT type 2 Indicator that the structure model may be wrong or deficient
   5 ALERT type 3 Indicator that the structure quality may be low
   3 ALERT type 4 Improvement, methodology, query or suggestion
   1 ALERT type 5 Informative message, check
```

It is advisable to attempt to resolve as many as possible of the alerts in all categories. Often the minor alerts point to easily fixed oversights, errors and omissions in your CIF or refinement strategy, so attention to these fine details can be worthwhile. In order to resolve some of the more serious problems it may be necessary to carry out additional measurements or structure refinements. However, the purpose of your study may justify the reported deviations and the more serious of these should normally be commented upon in the discussion or experimental section of a paper or in the "special_details" fields of the CIF. checkCIF was carefully designed to identify outliers and unusual parameters, but every test has its limitations and alerts that are not important in a particular case may appear. Conversely, the absence of alerts does not guarantee there are no aspects of the results needing attention. It is up to the individual to critically assess their own results and, if necessary, seek expert advice.

Publication of your CIF in IUCr journals

A basic structural check has been run on your CIF. These basic checks will be run on all CIFs submitted for publication in IUCr journals (*Acta Crystallographica*, *Journal of Applied Crystallography*, *Journal of Synchrotron Radiation*); however, if you intend to submit to *Acta Crystallographica Section C* or *E* or *IUCrData*, you should make sure that full publication checks are run on the final version of your CIF prior to submission.

Publication of your CIF in other journals

Please refer to the *Notes for Authors* of the relevant journal for any special instructions relating to CIF submission.

PLATON version of 06/01/2024; check.def file version of 05/01/2024

Datablock keck19 - ellipsoid plot

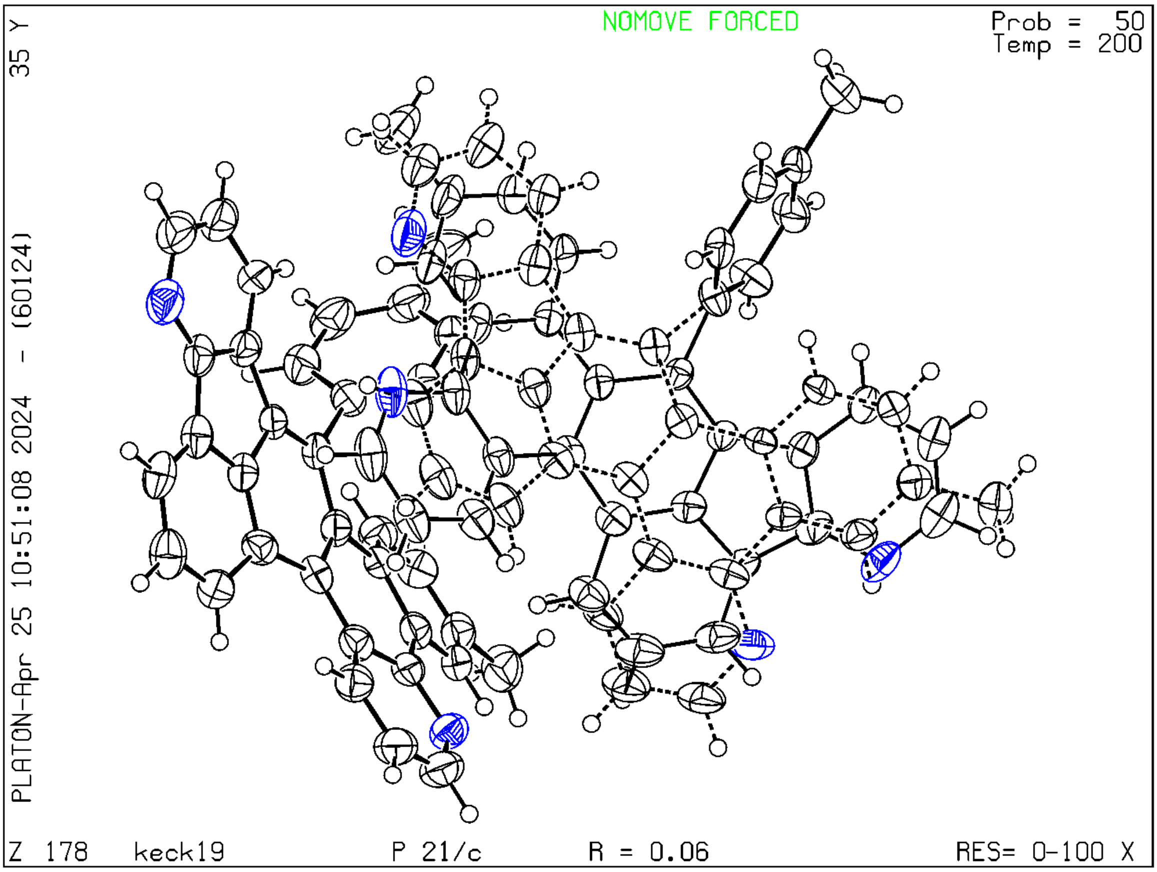

Supplement: Supplementary file 2 — Supporting Information [file CCDC_2486401_checkcif.pdf › CCDC_2486401_checkcif.pdf]
